
JOURNAL INFORMATION
==============================
NLM Title Abbreviation: Mol Neurodegener
Journal ID: Mol Neurodegener
NLM Journal ID: 101266600

Molecular Neurodegeneration

EISSN: 1750-1326
Publisher: BMC

ARTICLE INFORMATION
==============================
PMCID: PMC6685143
PMID: 31391110
DOI: 10.1186/s13024-019-0328-2
Article ID: 328
Article version: 1
Subjects: Meeting Abstracts

The 5th International Conference on Molecular Neurodegeneration: Overlapping Pathologies and Common Mechanisms

Stockholm, Sweden. 11-13 June 2018

Electronic publication date: 2019 Aug 7
Publication date: 2019
Volume: 14
Issue: Suppl 1
Issue sponsor: This publication was not supported by sponsorship.
Electronic Location ID: 30
Copyright: © The Author(s). 2019
License: Open AccessThis article is distributed under the terms of the Creative Commons Attribution 4.0 International License (http://creativecommons.org/licenses/by/4.0/), which permits unrestricted use, distribution, and reproduction in any medium, provided you give appropriate credit to the original author(s) and the source, provide a link to the Creative Commons license, and indicate if changes were made. The Creative Commons Public Domain Dedication waiver (http://creativecommons.org/publicdomain/zero/1.0/) applies to the data made available in this article, unless otherwise stated.
License URL: https://creativecommons.org/licenses/by/4.0/

Conference name: The 5th International Conference on Molecular Neurodegeneration
Conference Acronym: ICMN 2018
Conference location: Stockholm, Sweden
Conference date: 11-13 June 2018
issue-copyright-statement: © The Author(s) 2019

==============================

## INVITED TALKS

### O1 THE BIOLOGY OF PROTEOSTASIS IN AGING AND DISEASE

Richard I. Morimoto
Department of Molecular Biosciences, Rice Institute for Biomedical Research, Northwestern University, Evanston, IL 60208, USA
*Molecular Neurodegeneration* 2019, **14(Suppl 1):**INVITED

Protein quality control mechanisms regulated by the proteostasis network (PN), the heat shock response (HSR) and the unfolded protein responses of the endoplasmic reticulum and mitochondria are essential for all aspects of proteome quality control, cellular health and lifespan. Failure of these systems, for example during aging, stress or upon expression of mutant or damaged proteins leads to increased risk for neurodegeneration, metabolic diseases, cancer, and muscle wasting diseases. A question is whether it is possible to detect the cellular events of proteostasis failure, and to develop therapeutic strategies to arrest, reverse, or slow this decline. To understand how aging contributes to the challenge of protein conformation, we have combined a combination of approaches from physical biochemistry and supersaturation to discern the contribution of at-risk metastability together with genetic, cellular, molecular, genomic and systems approaches using C. elegans for an organismal view of proteostasis. Towards this, we have identified an event that occurs early in adulthood, at the transition of reproductive maturity, in which germ line stem cells prevent induction of the HSR in other somatic tissues. This germ line signal results in reduced expression of the jumonji demethylase in somatic tissues leading to elevated H3K27me3 repressive marks at the promoter regions for HS genes and other stress responsive components of the PN. The inability of HSF1 to bind in vivo results in a dramatic decline in the HSR and initiates proteostasis collapse that further amplifies misfolding of metastable proteins, resulting in the loss of cellular stress resistance, and a decline in cellular health and lifespan. To address whether these genetically regulated events of organismal proteostasis in aging can be reversed, we have taken genetic approaches to discovery modifier genes to reset proteostasis. For example, failure in the PN can be reversed by systemic overexpression of the jumonji demethylase, inhibition of germ line signaling, or induction of mild mitochondrial stress with the result that the HSR and other cell stress responses are not inhibited and persist throughout most of adult life. These results suggest that there are a number of cell autonomous and cell non-autonomous pathways that can be re-engaged to restore organismal proteostasis and to promote cellular healthspan.

### O2 THE COMPLEXITIES OF ALZHEIMER DISEASE HETEROGENEITY

Bradley T Hyman and Simon DuJardin
Alzheimer Disease Research Center, Massachusetts General Hospital, Boston MA USA
*Molecular Neurodegeneration* 2019, **14(Suppl 1):**INVITED

**Background**

Rate of progression of patients with Alzheimer's disease varies enormously. The inability to predict which patients will proceed with fast or slow rates of progression impact predictions, and compromise clinical care. Importantly, this heterogeneity also impacts clinical study design because studies to detect a difference in rate of progression must overcome this "noise" – needing thousands of patients followed for ~2 years to detect signal. We reasoned that the rate of tau propagation might be an important cofactor of rate of progression, since worsening symptoms are associated with more widespread tau accumulations.

**Materials and Methods**

We used a FRET based tau bioassay to determine Tau seeding ability form 32 brains of patients who had had AD with various rates of progression ante-mortem.

**Results**

Cases varied by ~2 fold or more in tau seeding properties; the rank order of the extent of tau seeding measured correlated with the rank order of rates of progression of the patients, as assessed by analysis of their premorbid assessments in a research protocol.

**Discussion**

Tau seeding bioactivity seems to be a factor that contributes to rate of progression in AD. The biophysical basis of the differences among cases in tau seeding ability is unknown.

**Conclusions**

If tau seeding from CSF corresponds also to rate of progression, it could represent an in vivo biomarker that might be useful clinically and in the context of clinical trials.

### O3 PROTEOPATHIC SEEDS IN NEURODEGENERATIVE DISEASES

Mathias Jucker
Department of Cellular Neurology, Hertie Institute for Clinical Brain Research, University of Tübingen, and German Center for Neurodegenerative Diseases (DZNE), D-72076 Tübingen, Germany
*Molecular Neurodegeneration* 2019, **14(Suppl 1):**INVITED

The commonality of many neurodegenerative disorders is the predictable temporal occurrence and progression of specific aggregated proteins in the brain. The hallmark proteopathy is Alzheimer's disease in which aggregated amyloid-β peptide (Aβ) is deposited in the brain

BMC

parenchyma (amyloid plaques), and aggregated Tau protein forms neuronal inclusions (neurofibrillary tangles). Multiple evidence suggests that Aβ and Tau aggregates can spread within and among brain regions and act as corruptive templates (seeds) that induce a chain-reaction of misfolding and aggregation of cognate proteins. The same appears true for α-synuclein aggregates in α-synucleinopathies. The insight that the prion paradigm also applies to Alzheimer´s disease and other age-related neurodegenerative diseases suggests new directions in search of biomarkers and novel therapeutic strategies.

## O4 COMMON MECHANISMS IN BRAIN AMYLOIDOSIS

Annemieke JM Rozemuller[1,2], Wim van Hecke[2], Baayla Boon[1], Jeroen JM Hoozemans[1]
[1]Department of Pathology VU Medical Centre, Amsterdam, the Netherlands; [2]Department of Pathology University Medical Centre, Utrecht, the Netherlands
*Molecular Neurodegeneration* 2019, **14(Suppl 1):**INVITED

**Correspondence:** Annemieke JM Rozemuller

**Background**

In the brain several forms of amyloidosis are known that originate from neuronal proteins: Amyloid Beta (Aβ) is found in senile plaques in aging and Alzheimer's disease and in cerebral amyloid angiopathy 1,2. Prion amyloid is seen in prion disorders with longer duration (> 1 year) also as plaques or CAA 3, 4. Amyloid molecule Abri is found in plaques and CAA in Familial British Dementia (BRI2 gene), and a partly similar molecule, ADAN has been described in a Familial Danish Dementia 4. The role of neuroinflammation in these neurodegenerative disorders is unknown. We have investigated the presence of complement, microglia, tau and ubiquitin in relation to the Amyloid beta plaques, Abeta CAA, prion plaques and prion CAA and studied the literature.

**Methods**

We studied 20 AD brains (several subtypes), 5 cases with CAA type1 including dyshoric amyloid beta deposits, 7 prion amyloidosis including 3 vCJD and 1 case with prion-CAA. Frozen tissue and or paraffin tissue was used for immunohistochemical studies, using antibodies against complement factors C3d and C4d, microglia markers (IBA1, CD68, HLADR/DP/DQ) and neurodegeneration (TAU (mab AT8), ubiquitin).

**Results**

Both Amyloid beta deposits and large, cortical prion amyloid deposits show complement- and microglia activation. Prion plaques in GSS can mimic Abeta plaques. Complement activation is seen in both Abeta- and in prion-CAA. Tau is present in AD as dystrophic neurites, threads and tangles in AD but this can also be found in young cases of GSS with large prion amyloid plaques. Dystrophic neurites are seen around dyshoric vessels that show microglia and complement activation. Literature shows the same for ABri and Adan4.

**Discussion**

Fibrillary amyloid deposits of different neuronal molecules show complement activation, microglia activation which lead in a later stage to neurodegeneration.

**Conclusions**

Common mechanism are found in different neurodegenerative disorders with brain amyloidosis.

## O5 A NETWORK-BASED PIPELINE FOR TARGET DISCOVERY AND VALIDATION: FROM THE AGING HUMAN CORTEX TO PRIORITIZED PROTEINS IN AMP-AD

Sara Mostafavi[1,2], Chris Gaiteri[3], Lei Yu[3], Charles C. White[5], Shinya Tasaki[3], Tracy L. Young-Pearse[4,5], David A. Bennett[3], Philip L. De Jager[5,6]
[1]University of British Columbia, Vancouver, British Columbia, Canada; [2]Canadian Institute for Advanced Research, Toronto, Canada; [3]Rush Alzheimer's Disease Center, Rush University Medical Center, Chicago, Illinois, USA; [4]Departments of Neurology, Brigham and Women's Hospital, Boston, Massachusetts, USA; [5]Broad Institute, Cambridge, Massachusetts, USA; 6 Center for Translational & Computational Immunology, Department of Neurology, Columbia University Medical Center, New York, New York, USA
*Molecular Neurodegeneration* 2019, **14(Suppl 1):**INVITED

**Correspondence:** Philip L. De Jager

**Background**

Through the National Institute of Aging's Accelerating Medicines Partnership for Alzheimer's disease (AMP-AD) program, we have established an analytic and experimental pipeline to identify and validate new targets for therapeutic development in AD.

**Methods**

We used RNAse data derived from the dorsolateral prefrontal cortex of participants in two prospective studies of aging to derive our network map. We used shRNA-mediated knockdown in primary human astrocytes and iPSC-derived neurons in functional validation studies, using amyloid beta1-42 in the supernatant as the outcome measure. We also used SRM proteomics to measure the level of specific proteins in these brains.

**Results**

Our first-generation network map of the aging human cortex leveraged transcriptomic and epigenetic data from more than 500 human subjects and identified several modules of co-expressed genes that relate to amyloid deposition, tau tangles, and/or cognitive decline. In one application, a subset of genes within module 109, were prioritized for in vitro functional and brain proteomic validation. These efforts have uncovered the role of PLXNB1 as a mediator of module 109's effect on amyloid deposition while IGFBP5 and other proteins capture a distinct effect of module 109 on cognitive decline not explained by AD pathologies. In a second example, we use our network map to dissect the immune component of the network and identify a role for a module 5, a transcriptional program related to microglial activation, in worsening cognitive decline through an acceleration of Tau deposition.

**Discussion**

Our network therefore has identified new target proteins affecting known biology such as amyloid and unknown biologies that contribute to aging-related cognitive decline.

**Conclusions**

A network approach to the aging brain has yielded concrete lead proteins and transcriptional programs (modules) that now deserve further investigation as candidate therapeutic targets.

1. De Jager et al. A multi-omic atlas of the human frontal cortex for aging and Alzheimer's disease research bioRxiv 251967

Mostafavi et al. A molecular network of the aging human brain provides insights into the pathology and cognitive decline of Alzheimer's disease. (2018) Nat Neuro (in press)
2. Patrick et al. A cortical immune network map identifies a subset of human microglia involved in Tau pathology bioRxiv 234351

## O6 MINING DATA FROM THE ACCELERATING MEDICINES PARTNERSHIP-ALZHEIMER'S DISEASE (AMP-AD) PROJECT TO IDENTIFY NEW THERAPEUTIC TARGETS

Todd E. Golde[1], Cara L. Croft[1], Hunter Futch[1], Brenda Moore[1], Benoit Giasson[1], Kevin Strang[1], Pedro Cruz[1], Yona Levites[1], Jada Lewis[1], David R. Borchelt[1], Paramita Chakrabarty[1], Marshall Goodwin[1], Yong Ran[1], Christian Lessard[1], Thomas Ladd[1], Daniel H. Ryu[1], Emily J. Koller[1], Mariet Allen[2], Minerva Carassquillo[2], Dennis Dickson[2], Michael DeTure[2], Wenlang Lin[2], Cory Funk[3], Hongdong Li[3,4], Duc Duong[5], Eric B. Dammer[6], Nicholas T. Seyfried[5], James J. Lah[6], Allan I. Levey[6], Nathan Price[3], Nilufer Ertekin-Taner[2]

[1]McKnight Brain Institute, Center for Translational Research in Neurodegenerative Disease, Department of Neuroscience and Neurology, University of Florida, Gainesville, FL, USA; [2]Department of Neuroscience, Mayo Clinic, Jacksonville, FL, USA; [3]Institute for Systems Biology, Seattle, WA 98109, USA; [4]Center for Bioinformatics, School of Information Science and Engineering, Central South University, Changsha, P.R. China; [5] Department of Biochemistry, Emory University, Atlanta, GA, USA; [6]Department of Neurology, Emory University, Atlanta, GA, USA

*Molecular Neurodegeneration* 2019, **14(Suppl 1):**INVITED

**Correspondence:** Todd E. Golde

**Background**

Amyloid and tau pathology are key, and likely essential components, of a complex neurodegenerative cascade that leads to symptomatic Alzheimer's disease (AD). However, there is a need to better understand the sequence of molecular events leading to symptomatic AD. Multi-omics data and new preclinical tools can help provide a better systems level understanding of the molecular changes driving AD and help identify novel therapeutic targets.

**Methods**

The Accelerating Medicines Partnership-Alzheimer's Disease (AMP-AD) program has generated and continues to generate large amounts of publically available multi-omic data from both human samples and preclinical models (https://www.synapse.org/#!Synapse:-syn2580853/wiki/409840). Vignettes about how our group is using this data and newly developed preclinical tools to nominate new targets for the treatment of AD will be presented.

**Results**

A very large number of perturbed networks are present in AD1,2. Whether these system level changes are consequences of disease or play an upstream pathophysiologic role has, typically, not been determined. Our comparative analyses of control, pathologic aging, AD and progressive supranuclear palsy and preclinical model data provides a framework to explore therapeutic targets that play a role in the transition to different disease stages of AD. Such data has enabled us to nominate new targets and we have developed new tools to validate those targets. The data can also be used to support the further evaluation of new targets arising from other lines of research.

**Discussion**

These studies can potentially help to identify precise therapeutic targets, provide biologic insight into the mechanism of action of the proposed targets, and inform on the direction of change needed for therapeutic benefit.

**Conclusions**

The multi-omics data generated by the AMP-AD initiative and new tools for preclinical target validation can be used to both nominate and rapidly evaluate new targets arising form the AMP-AD initiative.

## O7 INTEGRATING MOUSE GENETICS AND SYSTEMS BIOLOGY TO DISSECT HUNTINGTON'S DISEASE PATHOGENESIS

X. William Yang[1,2]

[1]Center for Neurobehavioral Genetics, Semel Institute for Neuroscience & Human Behavior; [2]Department of Psychiatry & Biobehavioral Sciences, David Geffen School of Medicine, University of California at Los Angeles (UCLA), Los Angeles, CA 90095, USA

*Molecular Neurodegeneration* 2019, **14(Suppl 1):**INVITED

**Background**

Huntington's disease (HD) is the most common dominantly inherited neurodegenerative disorder, and is characterized by motor, cognitive, and psychiatric symptoms, as well as a relentless and lethal disease progression. HD is caused by a CAG repeat expansion in the Huntingtin gene, however the molecular mechanisms underlying the pathogenesis of selective neurodegeneration and clinical symptoms remain poorly understood. Because HD is a monogenic disorder, it has the advantage that all HD patients are likely to share tractable disease mechanisms, and therapeutics based on such mechanisms should benefit most, if not all, HD patients.

**Methods**

To uncover critical HD molecular pathogenic mechanisms in an unbiased manner, we performed over 1000 RNA-sequencing and proteomics analyses of various brain samples from an allelic series of knockin mice expressing mutant Huntingtin with increasing CAG repeat length. Moreover, we also employed a mouse germline genetic perturbation approach to evaluate the candidate Huntingtin molecular network genes for their role in disease modification.

**Results**

We have defined Huntingtin CAG-length dependent differentially expressed (DE) genes and coexpression gene modules for multiple brain regions and the liver. We also are in the process of systematically evaluating the Huntingtin molecular network hub genes as modifiers of disease pathogenesis in vivo.

**Discussion**

Compared to the other brain regions, the striatum shows by far the largest number of Huntingtin CAG-length dependent DE genes and gene modules. Hence, the Huntingtin CAG-length dependent transcriptional dysregulation appears to reflect and may contribute to the underlying selective neuronal vulnerability in the disease.

**Conclusions**

Integrative genetics and systems biology is a powerful approach to identify novel molecular networks dysregulated in HD. Moreover, the germline genetic validation of such top disease network genes may provide novel mechanistic insights into the Huntingtin CAG-length driven and striatum-selective pathogenic process in HD.

## O8 TRIOS BASED IDENTIFICATION OF DE NOVO MUTATIONS AND FUNCTIONAL ANALYSIS OF THEIR PATHOGENIC ROLES IN ALS

Seung Hyun Kim[1], Su Min Lim[1], Young-Eun Kim[2], Ki-Wook Oh[1], Ki-Hyung Park[3], Chang-Seok Ki[4]
[1]Department of Neurology, Hanyang University College of Medicine, Seoul, Republic of Korea; [2]Department of Diagnositc Lab, Hanyang University College of Medicine, Seoul, Republic of Korea; [3]Department of Neurology, Gacheon University, College of Medicine, Incheon, Republic of Korea; [4]Department of Laboratory Medicine and Genetics, Samsung Medical Center, Sungkyunkwan University School of Medicine, Seoul, Republic of Korea
*Molecular Neurodegeneration* 2019, **14(Suppl 1):**INVITED

**Correspondence:** Seung Hyun Kim

**Background**

Amyotrophic lateral sclerosis (ALS) has been known as fatal and rapidly progressing neurodegenerative disease affecting both upper and lower motor neuron systems. The recent identification of novel genes linking ALS with FTD and the emerging concept of multisystem proteinopathies have changed the previous concept that ALS affects only the motor neuron system. And, diverse heterogeneous clinical presentations shown in ALS patients might be related with underlying genetic susceptibilities including de novo mutations or oligogenic or polygenic genetic roles in unresolved sporadic ALS. TRIO design [case-unaffected-parents Whole Exome Sequencing (WES)] can be a reliable study to delineate the underlying genetic roles in most young age onset sporadic ALS patients independent of our existing knowledge of ALS genes. Moreover, reliable cell modeling systems are crucible to hunt for evidence that newly identified variants have functional role in disease or motor neuron cell deaths before declaring that it is pathogenic.

**Methods**

TRIO study using WES was conducted in 30 young aged onset sporadic ALS patients and their unaffected parents (n=90). De novo variants were defined when both parents are homozygous and the proband is heterozygous. Rare variant was define as allele frequency less than 0.01 in dbSNP147 database, 1000 Genome Project, and gnomAD. Exome data from 100 ethnically-matched subjects and in-house disease control(exome data from 300 non-ALS patients) were used for validation. Induced neuron derived from patients skin fibroblast and animal models were used for validation.

**Results & Conclusion**

Based on TRIO study in Korean ALS population, numerous novel variants were found. After prioritization of 19 de novo variants, following genes including FUS, CLEC4C, ATP1A3, RabGef26 was selected for functional study using patient's fibroblast derived induced neuron and animal models. In this session, we will present candidates of de novo mutations found in young age onset sporadic ALS patients and their functional study data will be presented. These approach will be a reliable method to validate the pathophysiological roles of de novo mutations in ALS and which will be crucial step toward the precision medicine.

## O9 ALZHEIMER'S DISEASE SEQUENCING PROJECTS

Rudolph E. Tanzi
Department of Neurology, Massachusetts General Hospital, Harvard Medical School, Charlestown, MA, USA
*Molecular Neurodegeneration* 2019, **14(Suppl 1):**INVITED

Alzheimer's disease (AD) is the most common form of dementia affecting the elderly and is characterized by global cognitive decline. AD is strongly influenced by both genetic factors and lifestyle. While certain rare gene mutations, e.g. in the APP, PSEN1 and PSEN2 genes guarantee onset of AD before 60 years old, most cases of AD (>97%) involve genetic susceptibility factors, e.g. APOE, and lifestyle, e.g. diet, exercise, sleep, intellectual and social engagement, stress levels, and brain trauma. Most recently we have found that low-grade infections, e.g. bacterial, fungal, viral, in the brain may also play a role by rapidly nucleating beta-amyloid deposition as an antimicrobial protection response of the brain's innate immune system. Genetic susceptibility factors have been elucidated over the past decade using genome-wide association studies (GWAS) and more recently by follow up with whole genome sequencing (WGS) and whole exome sequencing (WES). We have carried out GWAS using approximately 50 million single nucleotide variants (SNV) from WGS and WES (whole genome sequencing association studies; WGSAS). As AD-linked/associated functional SNVs are identified in these studies, they are being tested in our 3D human stem cell-derived neural-glial culture models of AD, in which we have shown beta-amyloid directly drives tangle formation and neuroinflammation. Many of the more recently identified AD genes are involved in innate immunity, e.g. CD33, which we first reported in our family-based GWAS in 2008 (along with ADAM10 and ATXN1). To study CD33 and other innate immune-related AD genes, we have incorporated microglia into our 3D neural cultures while also utilizing classic transgenic mouse models. Aspects of all these studies will be covered.

## O10 TARGETING Aβ PROTOFIBRILS - FROM MUTATION TO POTENTIAL THERAPY IN ALZHEIMER'S DISEASE

Lars Lannfelt[1,2]
[1]Department of Public Health/Geriatrics, Uppsala University; [2]BioArctic AB, Warfvinges väg 35, 112 51 Stockholm, Sweden
*Molecular Neurodegeneration* 2019, **14(Suppl 1):**INVITED

**Background**

Disease modifying clinical trials in Alzheimer's disease (AD) targeting amyloid-β (Aβ) are actively being pursued. In recent years there has been an increasing interest in aggregated soluble Aβ species as being pathogenic1. Different types of soluble Aβ oligomers/protofibrils have been described. The Arctic mutation (Aβ E22G) causes AD due to an enhanced propensity to form protofibrils2. Soluble Aβ protofibrils are toxic, reduce synapse formation and neuronal survival, and represents an attractive therapeutic target.

**Methods**

The size of in vitro generated Aβ protofibrils was determined to be approximately 100-500 kDa. We used Aβ protofibrils as antigen and immunized mice, and generated hybridomas. Using inhibition ELISA for selection, protofibril selectiv antibodies were identified, one of them being mAb1583**.**

**Results**

The ability of mAb158 to inhibit protofibril-induced neurotoxicity was evaluated in vitro, and the therapeutic efficacy was analyzed in vivo using transgenic mouse models. mAb158 reduced levels of protofibrils in the brain and in CSF in transgenic mice4. mAb158 had an at least 500-fold stronger binding to Aβ protofibrils than to Aβ monomers and 10-15 stronger binding to protofibrils than to Aβ fibrils5, properties considered important for clinical efficacy and avoiding adverse event like brain edema.

**Discussion**

Several clinical trials in AD targeting Aβ have recently been unsuccessful. The explanation for these failures are probably too late intervention, too low doses, partly misdiagnosed patients and aiming at the wrong forms of Aβ. The humanized version of mAb158, BAN2401, is now in clinical development6. Targeting Aβ protofibrils is considered promising, as these Aβ species are toxic. A phase 2b study is being conducted in 856 patients with BAN2401. The 18 months results are expected to be ready this year.

## O11 GENETICS OF THE SYNUCLEINOPATHIES

Owen A. Ross
Department of Neuroscience, Mayo Clinic, Jacksonville, FL, USA
*Molecular Neurodegeneration* 2019, **14(Suppl 1):**INVITED

### Background
Synucleinopathies represent a distinct group of neurodegenerative disorders characterized by the presence of α-synuclein immunopositive aggregates. Clinically they present as the parkinsonian disorders; Parkinson's disease with or without dementia, dementia with Lewy bodies and multiple system atrophy with symptomatic heterogeneity observed across the spectrum. Genetic studies of Parkinson's disease have played a critical role in elucidating the underlying disease etiology and for generating disease in vitro/in vivo models.

### Methods
Genome-wide association studies, whole-genome and exome sequencing, family- and population-based studies, functional validation and induced-pluripotent stem cells.

### Results
Genome-wide association studies in Parkinson's disease have now identified 41 loci as containing susceptibility-altering variants and with the genes identified through familial studies they nominate key cellular pathways including autophagy, mitophagy and lysosomal function1. Genome-wide association studies have identified significant disease loci for dementia with Lewy bodies with an interesting overlap with both Parkinson's and Alzheimer's disease with the key loci being SNCA, GBA and APOE2. No significant hits for multiple system atrophy were identified in the recent genome-wide association study but some candidates were observed just below the threshold that are overlapping with Parkinson's disease including the MAPT locus3.

### Discussion
The genetic heterogeneity of the synucleinopathies mirrors the clinical presentation and can be exploited to define specific genetic subtypes that may in the long term determine the participants of future targeted clinical trials.

### Conclusion
The next wave of genes we await will likely be identified through the large-scale population based sequencing projects, coupled with functional validation, that are already underway4.

### References
1. Chang D, Nalls MA, Hallgrimsdottir IB, et al: A meta-analysis of genome-wide association studies identifies 17 new Parkinson's disease risk loci. Nat Genet 2017; 49: 1511-1516.
2. Guerreiro R, Ross OA, Kun-Rodrigues C, et al: Investigating the genetic architecture of dementia with Lewy bodies: a two-stage genome-wide association study. Lancet Neurol 2018; 17: 64-74.
3. Sailer A, Scholz SW, Nalls MA, et al: A genome-wide association study in multiple system atrophy. Neurology 2016; 87: 1591-1598.
4. Jansen IE, Ye H, Heetveld S, et al: Discovery and functional prioritization of Parkinson's disease candidate genes from large-scale whole exome sequencing. Genome Biol 2017; 18: 22.

## O12 MODULATION OF AMYLOID DEPOSITION AND NEUROINFLAMMATION BY THE MICROBIOME

Hemraj Dodiya[1], Shabana Shaik[1], Myles Minter[1], Martin Zhang[3], Eugene Chang[2], Rudolph Tanzi[3], Sangram Sisodia[3]
[1]Department of Neurobiology; [2]Medicine, University of Chicago, Chicago Il USA; [3]Department of Neurology, Harvard Medical School, Charlestown, MA USA
*Molecular Neurodegeneration* 2019, **14(Suppl 1):**INVITED

**Correspondence:** Sangram Sisodia

### Objectives
Animal models of Alzheimer's disease (AD) recapitulate the severe amyloidosis and neuroinflammation that is evident in the human disease. It is now well established that inflammation associated with amyloid deposition reflects the activation of astrocytes and microglia in response to injury, but the role of peripheral tissues and more importantly, the microbiota in regulating innate immunity that in turn leads to CNS dysfunction has not, to date been defined. We have tested the hypothesis that the composition of the intestinal microbiome plays a key role in modulating neuro-inflammation that will ultimately influence amyloid deposition in two established mouse models of β-amyloidosis.

### Methods
We orally administered a combination of antibiotics to induce rapid and sustained alterations in gut microbial populations. The antibiotic cocktail was administered either postnatally or throughout the lifetime of the animal prior to cull and we employed IHC, biochemical and molecular assays to evaluate amyloid deposition and neuroinflammation in the mouse models.

### Results
Our studies indicate that alterations in the microbiome parallel changes in plasma cytokines and chemokines, reductions in amyloid deposition and modulation of morphological and transcriptional landscapes of microglia.

### Conclusions
Our studies reveal an unexpected, but significant alteration in amyloid deposition and microglial phenotypes in the brains of transgenic mice upon treatment with orally administered antibiotics.

### Acknowledgments
This work was supported by Cure Alzheimer's Fund (CAF) the Edward H. Levi Fund and the Adler Foundation. SSS is a paid Consultant of Eisai Research Labs Inc. and AZTherapies Inc.

## O13 EFFECTS OF APOE IN TAU-MEDIATED NEURODEGENERATION

Yang Shi and David M. Holtzman
Department of Neurology, Hope Center for Neurological Disorders, Knight ADRC, Washington University, St. Louis, MO, USA
*Molecular Neurodegeneration* 2019, **14(Suppl 1):**INVITED

**Correspondence:** David M. Holtzman

### Background
APOE genotype is the strongest genetic risk factor for Alzheimer's disease (AD). There is strong evidence that a large part of the effect of ApoE on AD pathogenesis is via its ability to influence the onset and accumulation of aggregated forms of Abeta in the brain parenchyma through its regulation of Abeta aggregation and clearance. There is emerging evidence that ApoE may also mediate some of its effects in AD and other tauopathies via influencing both tau as well as the brain's innate immune response.

**Methods**

We crossed P301S Tau transgenic (Tg) mice, a mouse model of tauopathy, to human ApoE knockin mice and to ApoE knockout mice and then assessed the effects of ApoE on tau, tau-mediated neurodegeneration, and the innate immune response.

**Results**

We found that ApoE strongly enhances neurodegeneration in P301S mice with ApoE4 having the greatest effects. Very little to no neurodegeneration was seen in the absence of ApoE. The enhanced neurodegeneration seen with ApoE was accompanied by a strong neurodegenerative type glial response and similar responses could be observed in cell culture. In preliminary experiments, we have crossed P301S Tau Tg mice with mice transgenic mice that overexpress the low density lipoprotein receptor (LDLR) in the brain. So far, we have noted that at 9 months of age, P301S Tau Tg mice have significant tauopathy and brain atrophy. In P301S Tau/LDLR Tg mice, ApoE levels are markedly lowered and there is significantly less brain atrophy.

**Discussion**

Understanding the mechanism as to how ApoE contributes to tau-mediated neurodegeneration may provide avenues to development of new therapeutic approaches.

**Conclusions**

ApoE contributes to neurodegeneration in a mouse model of primary tauopathy. This effect appears to be mediated in part via ApoE's effect on the innate immune response.

**Funding**

NIH NS090934, AG047644, and the JPB Foundation

## O14 RTN3 mediates formation of dystrophic neurites in Alzheimer's brains

Riqiang Yan, Md. Golam Sharoar
Department of Neurosciences, University of Connecticut School of Medicine, Farmington, CT 06030, USA
*Molecular Neurodegeneration* 2019, **14(Suppl 1):**INVITED

**Correspondence:** Riqiang Yan

Alzheimer's disease (AD) is the most common age-dependent neurodegenerative disease, and presence of dystrophic neurites is one of the typical features in AD brains. The presence of dystrophic neurites is correlated with impaired synaptic functions. It remains to understand what drives the formation of dystrophic neurites and their molecular natures. We discovered that tubular endoplasmic reticulum (ER) protein reticulon-3 (RTN3) was abundantly accumulated in the dystrophic neurites in brains of AD patients and mainly in the form of clustered tubular ER. Transgenic mice overexpressing RTN3 develop similar tubular ER-enriched dystrophic neurites. However, dystrophic neurites in AD brains are also shown to enrich multivesicle bodies. The relationship between different populations of dystrophic neurites are intriguing. In our recent studies, we aimed to understand how RIDNs are developed in AD mouse brains (5xFAD and APP/PS1ΔE9 mice) and how they are related to or differed from dystrophic neurites that are enriched with other proteins by analyzing 2D and 3D electron microscopic images. We showed that RTN3 appears to mediate the early formation of dystrophic neurites and deposition of amyloid peptides could induce growth of dystrophic neurites by impairing autophagy, ubiquitin proteasome system and normal ER distribution in the axons.

## O15 ROLE OF BRAIN VASCULAR CHANGES IN ALZHEIMER'S DISEASE PATHOGENESIS

Ji-Young Choi, Sangmee A. Jo
Department of Nanobiomedical Sciences & BK21 PLUS NBM Global Research Center for Regenerative Medicine, Dankook University Cheonan-si, Choongnam, Korea.
*Molecular Neurodegeneration* 2019, **14(Suppl 1):**INVITED

**Correspondence:** Sangmee A. Jo

**Background**

Cerebrovascular inflammation and blood-brain barrier dysfunction (BBB) contribute to pathogenesis of vascular-based neurodegenerative brain disorders such as Alzheimer's disease. A typical outcome of BBB dysfunction is leukocyte transmigration from the vessel to the brain, which could be a key event promoting brain inflammation and neuronal injury. Here, we investigated the molecular and cellular mechanism of how vascular inflammation results in leukocyte transmigration and in particular, influences on AD pathogenesis. For this purpose, we examined the effect of tumor necrosis factor alpha (TNF-α) on expression of ICAM1, a vascular adhesion protein involved in leukocyte adhesion to the vessels and the epigenetic regulatory mechanism involved in TNF-induced ICAM1 induction in human microvascular endothelial cells (HBMVECs). In addition, we investigated the role of ICAM1 in expression of neprilysin, an amyloid degrading enzyme.

**Methods**

HBMVECs were cultured, treated with TNF-α (10 ng/ml), and analyzed for Western blotting, RT-PCR, ChIPI, neutrophil adhesion assay, leukocyte transmigration assay, and immunofluorescence staining. For in vivo study C57BL/6 mice were injected with TNF-α (9 μg/kg) with or without drugs and the brain was analyzed for neutrophil infiltration. In addition, APP Swedish/PS1-E9 deletion mice were used for examining the ICAM1 expression pattern.

**Results**

TNF-α dramatically increased ICAM1 mRNA and protein levels in HBMVECs and mouse brain microvessels. Experiments including ChIPI revealed that TNF-α reduced methylation of histone H3 at lysines 9 (H3K9), a well-known residue involved in gene suppression, and KDM4B, a histone demethylase targeting H3K9me2 was involved in TNF-α-induced ICAM1 upregulation and neutrophil transmigration. Interestingly, knock-down of ICAM1 significantly increased the neprilysin protein level with a concomitant reduction of the amyloid level. ICAM1 expression in AD mice brain increased at the very earlier stage pathogenesis (3~4 month old age) with reduction of the neprilysin protein.

**Conclusions**

Collectively, we demonstrated that modification of H3K9me2 by G9a and KDM4B regulate the expression of vascular adhesion proteins and that inhibition of adhesion proteins or KDM4B reduces inflammation-induced leukocyte extravasation and amyloid pathology. Thus, blocking ICAM1 or KDM4B could offer a novel therapeutic opportunity treating brain diseases.

**Acknowledgements**

This work was supported by the research grant of the National Research Foundation of Korea (2017R1A2B4002861).

O16 NOVEL STRATEGIES FOR FACILITATION OF AMYLOID CLEARANCE IN THE BRAIN

**Background**
Amyloid-β peptide (Aβ) and tau are major components of senile plaques and neurofibrillary tangles, respectively, deposited in the brains of Alzheimer disease (AD) patients. Several lines of evidence suggest that accumulation of Aβ by increased production or decreased clearance induces the aggregated tau pathology, which spreads through neuronal circuits and finally leads to neurodegeneration in the AD brain. Thus, these amyloidogenic proteins play a critical role in the pathogenesis of AD. To date, antibodies against these amyloidogenic proteins have been tested in clinical trials, and some antibodies accelerate the clearance of amyloid proteins. However, because of limited brain penetration efficacy of the antibodies, the development of novel approaches to facilitate the amyloid clearance has been required.
**Methods and Results**
We recently identified novel photooxygenation catalysts that specifically bind to the cross-β-sheet structure of target proteins, such as Aβ (Ni et al., Chem 2018). Photooxygenation reaction under near-infrared (NIR) light irradiation attenuated the aggregation and deposition of amyloidogenic proteins in vitro as well as in vivo.
**Discussion**
Since amyloid aggregates commonly preserve the cross-β-sheet structure, the NIR photoactivatable catalysts should be effective to degrade several amyloidogenic proteins (e.g., tau, α-synuclein, TDP-43, amylin). Thus, our strategy would provide a novel therapeutic strategy against both systemic and organ-specific amyloidosis. Moreover, as photoactivatable approach enables us to diminish amyloids in spatiotemporal manner, these catalysts can be utilized in the analysis of amyloid pathology spreading in situ.
**Conclusion**
Artificial photooxygenation catalyst would be a potential therapeutic strategy against amyloid diseases.

**Reference**
1. Ni J, Taniguchi A, Kuninobu Y, Ozawa S, Hori Y, Kuninobu Y, Saito T, Saido TC, Tomita T, Sohma Y, Kanai M: Near-infrared photoactivatable oxygenation catalysts of amyloid protein. Chem in press.

O17 CELLULAR AND MOLECULAR MECHANISMS OF PARKINSON'S DISEASE

Jae-Hyeon Park, Marion Delenclos, Pamela J. McLean
Department of Neuroscience Research, Mayo Clinic, Jacksonville, FL, USA
*Molecular Neurodegeneration* 2019, **14(Suppl 1):**INVITED

**Correspondence:** Pamela J. McLean
**Background**
Despite its predominant localization in the cytosol, alpha-synuclein (αsyn) is found localized to mitochondria in post-mortem PD brains (1). Within the mitochondria, αsyn accumulation impairs complex I and IV function, decreases mitochondria membrane potential, increases levels of mitochondrial ROS, and increases mitochondrial-dependent apoptosis associated with cytochrome c release from the mitochondria (2,3). Maintaining mitochondrial health is essential to prevent neuronal cell death in the brain. Sirtuin 3 (SIRT3) is a NAD+-dependent protein deacetylase exclusively localized to the mitochondria where it regulates mitochondrial processes such as protein deacetylation (4). SIRT3 is expressed at high levels in the brain and other nervous system tissues (5,6), and can act as a pro-survival factor, playing an essential role in protecting neurons under conditions of excitotoxicity (7) and rescuing neuronal loss in models of neurodegeneration (8).
**Methods**
A stable cell line expressing αsyn oligomers, a rat AAV model of PD, and patient fibroblasts from PD patients and healthy controls were assessed for changes in markers of mitochondrial biogenesis, SIRT3 protein levels, and SIRT3 activity using established assays.
**Results**
Overexpression of αsyn oligomers in the mitochondria of cultured cells and rat nigral neurons resulted in decreased mitochondrial SIRT3 protein levels, decreased SIRT3 activity, and decreased mitochondrial biogenesis that could be rescued with AMPK activator AICAR. Patient fibroblasts harboring a triplication of SNCA gene locus had significantly reduced SIRT3 activity and decreased mitochondrial biogenesis compared to healthy controls.
**Discussion**
These data support a hypothesis whereby accumulation of mitochondrial αsyn results in increased mitochondrial ROS via an AMPK/PGC1α/SIRT3-mediated pathway.
**Conclusion**
Upregulation of SIRT3 and/or increased SIRT3 activity are potential targets for novel PD therapeutics.

**References**
1. Devi, L., Raghavendran, V., Prabhu, B. M., Avadhani, N. G., and Anandatheerthavarada, H. K. (2008) Mitochondrial import and accumulation of alpha-synuclein impair complex I in human dopaminergic neuronal cultures and Parkinson disease brain. J Biol Chem 283, 9089-9100
2. Tanaka, Y., Engelender, S., Igarashi, S., Rao, R. K., Wanner, T., Tanzi, R. E., Sawa, A., V, L. D., Dawson, T. M., and Ross, C. A. (2001) Inducible expression of mutant alpha-synuclein decreases proteasome activity and increases sensitivity to mitochondria-dependent apoptosis. Hum Mol Genet 10, 919-926
3. Vekrellis, K., Xilouri, M., Emmanouilidou, E., and Stefanis, L. (2009) Inducible over-expression of wild type alpha-synuclein in human neuronal cells leads to caspase-dependent non-apoptotic death. J Neurochem 109, 1348-1362
4. Lombard, D. B., Alt, F. W., Cheng, H. L., Bunkenborg, J., Streeper, R. S., Mostoslavsky, R., Kim, J., Yancopoulos, G., Valenzuela, D., Murphy, A., Yang, Y., Chen, Y., Hirschey, M. D., Bronson, R. T., Haigis, M., Guarente, L. P., Farese, R. V., Jr., Weissman, S., Verdin, E., and Schwer, B. (2007) Mammalian Sir2 homolog SIRT3 regulates global mitochondrial lysine acetylation. Mol Cell Biol 27, 8807-8814
5. Sidorova-Darmos, E., Wither, R. G., Shulyakova, N., Fisher, C., Ratnam, M., Aarts, M., Lilge, L., Monnier, P. P., and Eubanks, J. H. (2014) Differential expression of sirtuin family members in the developing, adult, and aged rat brain. Front Aging Neurosci 6, 333
6. Zeng, L., Yang, Y., Hu, Y., Sun, Y., Du, Z., Xie, Z., Zhou, T., and Kong, W. (2014) Age-related decrease in the mitochondrial sirtuin deacetylase Sirt3 expression associated with ROS accumulation in the auditory cortex of the mimetic aging rat model. PLoS One 9, e88019
7. Kim, S. H., Lu, H. F., and Alano, C. C. (2011) Neuronal Sirt3 protects against excitotoxic injury in mouse cortical neuron culture. PLoS One 6, e14731
8. Anamika, Khanna, A., Acharjee, P., Acharjee, A., and Trigun, S. K. (2017) Mitochondrial SIRT3 and neurodegenerative brain disorders. J Chem Neuroanat S0891-0618 doi: 10.1016/j.jchemneu.2017.11.009.

O18 MECHANISM OF CELL-TO-CELL PROPAGATION OF ALPHA-SYNUCLEIN

Seung-Jae Lee
Deaprtment of Biomedical Sciences, Seoul National University College of Medicine, Seoul, Korea
*Molecular Neurodegeneration* 2019, **14(Suppl 1):**INVITED

Synucleinopathies are neurological disorders, characterized by neuronal and glial deposition of α-synuclein aggregates. These disorders include Parkinson's disease (PD), dementia with Lewy bodies, and multiple system atrophy. Cell-to-cell propagation of these aggregates are thought to be the underlying mechanism of aggregate spreading in patients' brain and perhaps of clinical progression. Interfering with the aggregate transmission can thus be a potential strategy for halting the disease progression. However, the mechanism by which α-synuclein aggregates spread remains undefined. Here, I present the results showing the identification of receptors that mediate the propagation process. My lab identified toll-like receptor 2 (TLR2), an innate immune receptor, as the receptor for neuron-released α-synuclein oligomers in microglia. I will show evidence that TLR2 plays an important role in cell-to-cell propagation of α-synuclein aggregates, regulating both secretion and uptake of the aggregates. Furthermore, administration of a neutralizing antibody for TLR2 interferes with the intercellular propagation of α-synuclein and thus, alleviates synucleinopathy lesions and neuroinflammation. I propose the anti-TLR2 treatment as a therapeutic strategy for Parkinson's disease and related synucleinopathies.

## O19 LRRK2 IN AUTOPHAGY AND PARKINSON'S DISEASE

Jie Shen
Department of Neurology, Brigham and Women's Hospital, Program in Neuroscience, Harvard Medical School, USA
*Molecular Neurodegeneration* 2019, **14(Suppl 1):**INVITED

**Background**
LRRK2 mutations are the most common genetic cause of Parkinson's disease. Previous analysis of LRRK2-deficient mice identified no detectable phenotype in the brain but remarkable PD-like changes in the aged kidney, including striking α-synuclein accumulation and aggregation, impairment of the autophagy-lysosomal pathway and increases in apoptosis.
**Methods**
We generated LRRK-deficient mice, in which LRRK2 and its functional homologue LRRK1 are inactivated, as LRRK1, which is relatively abundant in the brain, may compensate for the loss of LRRK2 in LRRK2-/- brains.
**Results/Conclusions/Discussion**
LRRK1/2 double knockout (LRRK DKO) mice exhibit earlier mortality at ~16 months of age with marked reduction of body weight but largely normal brain weight. Interestingly, LRRK DKO mice, but not LRRK1 or LRRK2 single KO mice, develop age-dependent DA neurodegeneration, as shown by age-dependent reduction of DA neurons in the SNpc at 14-15 months but not at younger ages. The cerebral cortex and cerebellum, however, are unaffected, though noradrenergic neurons in the locus coeruleus and medium spiny neurons of the striatum are also reduced in LRRK DKO mice at 15 months. The selective, age-dependent neurodegeneration is accompanied with increases in apoptotic cell death, increased levels of α-synuclein, and impaired autophagy-lysosomal pathway. Quantitative electron microscopy (EM) analysis further revealed dramatic increases of autophagic vacuoles in the SNpc of LRRK DKO mice at 10 months, before the onset of DA neuron loss and increases of apoptosis. These results demonstrate that LRRK is required for age-dependent survival of DA neurons, and plays an essential role in the regulation of the autophagy-lysosomal pathway in the aging brain.

**References**
1 Tong Y, Yamaguchi H, Giaime E, Boyle S, Kopan R, Kelleher RJ, Shen J. Loss of leucine-rich repeat kinase 2 causes impairment of protein degradation pathways, accumulation of a-synuclein, and apoptotic cell death in aged mice. Proc Natl Acad Sci USA 2010, 107:9879-84.
2 Tong Y, Giaime E, Yamaguchi H, Ichimura T, Liu Y, Si H, Cai H, Bonventre JV, Shen J. Loss of leucine-rich repeat kinase 2 causes age-dependent biphasic alterations of the autophagy pathway. Mol Neurodegener 2012, 7:2.
3 Tong Y, Shen J. Genetic analysis of Parkinson's disease-linked leucine-rich repeat kinase 2. Biochem Soc Trans 2012, 40:1042-6.
4 Giaime E, Tong Y, Wagner LK, Yuan Y, Huang G, Shen J. Age-dependent dopaminergic neurodegeneration and impairment of the autophagy-lysosomal pathway in LRRK-deficient mice. Neuron 2017, 96(4):796-807.

## O20 C9Orf72 AND ALS

Leonard Petrucelli
Department of Neuroscience, Mayo Clinic, Jacksonville, FL, USA
*Molecular Neurodegeneration* 2019, **14(Suppl 1):**INVITED

The major genetic cause of frontotemporal dementia (FTD) and amyotrophic lateral sclerosis (ALS) is a C9orf72 G4C2 repeat expansion. Proposed mechanisms by which the expansion causes c9FTD/ALS include toxicity from repeat-containing RNA and from dipeptide repeat (DPR) proteins translated from these transcripts. To investigate the contribution of poly(GR) DPRs to c9FTD/ALS pathogenesis in a mammalian in vivo model, we generated mice that expressed GFP-(GR)100 in the brain. GFP-(GR)100 mice developed age-dependent neurodegeneration, brain atrophy, as well as motor and memory deficits through the accumulation of diffuse, cytoplasmic poly(GR). Poly(GR) colocalized with ribosomal subunits and the translation initiation factor eIF3η in GFP-(GR)100 mice and, of importance, in c9FTD/ALS patients and in a c9orf72 repeat AAV mouse model. Combined with the differential expression of ribosome-associated genes in GFP-(GR)100 mice, these findings demonstrate poly(GR)-mediated ribosomal distress. Indeed, poly(GR) inhibited canonical and non-canonical protein translation in HEK293T cells, and also induced the formation of stress granules and delayed their disassembly. These data suggest that poly(GR) contributes to c9FTD/ALS by impairing protein translation and stress granule dynamics consequently causing chronic cellular stress and preventing cells from mounting an effective stress response.

## O21 UNRAVELING THE ROLE OF INNATE IMMUNE PATHWAYS IN NEURODEGENERATION

Bruce T. Lamb[1], Adrian Oblak[1], Shane Bemiller[1], Taylor Jay[3], Lynn Bekris[2], James Leverenz[2], Gary Landreth[1]
[1]Indiana University School of Medicine; [2]The Cleveland Clinic; [3]Case Western Reserve University
*Molecular Neurodegeneration* 2019, **14(Suppl 1):**INVITED

**Correspondence:** Bruce T. Lamb
**Background**
Increasing evidence suggests that neuroinflammation is an important contributor to Alzheimer's disease (AD) pathogenesis, as underscored by the identification of immune-related genetic risk factors for AD, including coding variants in the gene TREM2 (triggering receptor expressed on myeloid cells 2) amongst others. Understanding TREM2 function promises to provide important insights into how neuroinflammation contributes to AD pathology. Notably, recent studies suggest that development of the various AD pathologies (amyloid and tau) occurs across several decades.
**Hypothesis**
The central hypothesis of these studies is that TREM2 and other immune pathways play both pathology-dependent and stage-dependent roles in Alzheimer's disease that will be critical to understand in order to ultimately target these pathways therapeutically.
**Methods**
These studies utilize mouse models of AD and models that contain genetic alterations in innate immune pathway genes as well as analysis of human AD samples.
**Results/Conclusions**
Notably, we provide evidence that TREM2 plays different roles at different stages of disese progression in a transgenic mouse model of AD that develop robust amyloid pathology. Further, TREM2 deficiency seems to play a different role in the modulation of tau pathology. Finally, human studies also support that TREM2 may play a unique role at different stages of disease progression. Taken together, these results suggest that TREM2 and other innate immune pathways implicated in AD, may play distinct functional roles that are both stage- and pathology-dependent. We will also provide an update on the Model Organism Development and Evaluation for Late-onset AD (MODEL-AD) Consortium, which focuses on developing, characterizing and distributing more accurate animal models of AD, that includes a strong focus on innate immune pathways.

O22 DAMPENING THE MICROGLIAL RESPONSE TO AMYLOID PLAQUES IS NEUROPROTECTIVE IN AGED AD-LIKE MICE

Qiaoqiao Shi and Cynthia A. Lemere
Ann Romney Center for Neurologic Diseases, Brigham and Women's Hospital, Harvard Medical School, Boston, MA, USA.
*Molecular Neurodegeneration* 2019, **14(Suppl 1):**INVITED

**Correspondence:** Cynthia A. Lemere

**Background**

Complement has been shown to be involved in microglia-mediated synaptic pruning during brain development [1] and in the response to amyloid-β (Aβ) oligomers in early pre-plaque stages of Alzheimer's disease (AD) [2] as well as aging [3]. We investigated the role of complement C3, a central molecule in the pathway, in Aβ deposition and synapse loss at later, plaque-rich stages of AD [4].

**Methods**

APPswe/PS1dE9 Tg mice were crossed with complement C3 knockout (C3 KO) mice and aged to 16 months. Male APP/PS1;C3 KO mice, wildtype (WT), APP/PS1 and C3 KO mice were compared for cognitive flexibility (Water T Maze, WTM) and anxiety (Elevated Plus Maze, EPM). Aβ plaque load, gliosis, hippocampal synaptic changes and neuron number were evaluated. We also generated an inducible C3 KO mouse model (C3fl/fl;UBC-Cre-ERT2) (C3iKO) in which tamoxifen treatment leads to global knockdown of C3.

**Results**

C3-deficient APP/PS1 mice had significantly better cognitive flexibility and were less anxious (EPM) than APP/PS1 mice, despite having more Aβ plaque deposition. While the number of hippocampal glia did not change, microglia appeared to be less activated in the C3-deficient APP/PS1 mice and fewer glia moved into the plaque center. Several pro-inflammatory cytokines were reduced in the APP/PS1;C3 KO mice. Hippocampal synapses and neuron numbers were rescued by C3-deficiency in APP/PS1 mice. In agreement, male C3iKO mice treated with tamoxifen at 9 months resulted in reduced C3 protein levels in plasma, an increase in synaptic puncta, and significantly higher LTP in hippocampal slices at 12 months of age. Discussion: Our new C3iKO model will allow us to determine whether C3 lowering is protective in early stage neurodegenerative diseases and other health conditions.

**Conclusions**

Complement C3 and/or downstream complement activation fragments appear to play a key role neuronal health and function in the aging brain and AD.

O23 PROFILE OF CNP520, A BACE-1 INHIBITOR FOR PREVENTION STUDIES IN ALZHEIMER'S DISEASE

**Correspondence:** Ulf Neumann

**Background**

Stopping amyloid-β (Aβ) deposition by BACE-1 inhibition appears to be a promising strategy to treat Alzheimer's disease (AD), but treatment in established dementia stages was unsuccessful. We hypothesize that BACE-1 inhibitor treatment needs to start in early stage Aβ deposition and before the onset of significant neurodegeneration. Prevention treatment puts high hurdles on the safety and tolerability, to be addressed already in the drug design and selection process.

**Methods**

CNP520 was designed and profiled in vitro, using animal pharmacological, pharmacokinetic and metabolism studies and underwent toxicological profiling with oral studies up to 39 weeks duration Clinical Phase I and Phase IIa studies in healthy elderly volunteers established its safety, tolerability, and active dose range.

**Results**

CNP520 is a potent and selective BACE-1 inhibitor in vitro. Due to its high brain penetration and plasma protein binding, free compound levels in the periphery are low. Significant Aβ reduction was observed in animals. Results of toxicology studies have not raised major safety concerns. No effects on myelin, muscle spindles, retina, pigmented organs were observed. Humans Phase I studies showed a dose- and time-dependent reduction of CSF Aβ, and a pharmacokinetic profile suitable for once-daily dosing. A 3-months study showed that CNP520 is safe and tolerated in a dosing range that result in 90% reduction of CSF Aβ.

**Discussion**

The profile of CNP520 supports its use in prevention studies of AD. Generation Study 1 and 2 have been initiated, which aim to test CNP520 at 15 or 50 mg in a population of enhanced risk to develop AD, patients are being included based on their age, APOE4 genotype and Aβ positivity.

**Conclusions**

Properties of CNP520 make it suited for the use in prevention trials of AD, the ongoing clinical studies will allow to test the concept of prevention treatment in AD.

O24 UPDATE ON BIOMARKERS FOR ALZHEIMER'S DISEASE

Henrik Zetterberg
University of Gothenburg, Sweden
*Molecular Neurodegeneration* 2019, **14(Suppl 1):**INVITED

Neurodegenerative diseases are (to a varying degree) characterized by accumulation of misfolded proteins, microglial and astrocytic activation, as well as synaptic and neuronal degeneration. The presentation will review the growing list of fluid biomarkers that reflect such degenerative pathologies across dementia disorders and, whenever possible, in relation to attempted therapeutic intervention. Successful and less successful attempts to transform cerebrospinal fluid biomarkers to simple blood tests will also be detailed, along with a discussion on novel promising biomarker candidates

## O25 INTERACTIVE PATHOLOGICAL PROCESSES AND THEIR TIME COURSE IN ALZHEIMER'S DISEASE AS REVEALED BY PET IMAGING

Agneta Nordberg
Department of Neurobiology, Care Sciences and Society, Division of Clinical Geriatrics, Center for Alzheimer Research, Karolinska Institutet, Stockholm, Sweden
*Molecular Neurodegeneration* 2019, **14(Suppl 1):**INVITED

**Background**
Several neurodegenerative diseases are characterized by proteinopathies. Alzheimer's disease (AD) is the most common dementia disease and despite intensive research there is still no cure. The disease processes are most probably initiated decades before clinical symptoms occur which underlines the importance of understanding the time courses of various pathophysiological processes in the brain, to develop early diagnostic markers and allowing secondary prevention and disease modifying therapy.
Positron emission tomography (PET) provides important knowledge of in vivo pathology at different stages of brain diseases in comparison to post-mortem pathology observations at final stage of the disease. PET provides new tools and avenues for understanding of in vivo pathology and open up new windows for early detection and diagnosis of AD and other proteinopathies.
**Methods**
By performing PET studies using different PET tracers in a multitracer paradigm we can measure pathological and functional changes including amyloid plaques, tau deposition, inflammatory changes such as astrocytosis and cerebral glucose metabolism in brain in the same patient. This allow a further understanding of the time course and relationship between the different brain processes and their relationship also with cognition, biomarkers in cerebrospinal fluid (CSF) as well as structural changes measured by magnet resonance imaging (MRI).
**Results and Discussion**
Amyloid starts to accumulate very early already at presymptomatic stages of AD and reaches almost a plateau at early symptomatic stages. Emerging evidences underline the importance of neuroinflammation in AD and its active role in AD pathology which strongly motivates more deeply understand the involvement and time course of early inflammatory processes and their possible causal role in AD progression to unravel the relationship and coupling between astrogliosis and different proteinopathies in relation to synaptic functions and cognition. The prominent initially high and declining astrocytosis in AD during disease progression, contrasting with the increasing amyloid plaque deposition as measured by PET, suggesting that astrocyte activation might be participating in the initiation of AD pathology prior tau deposition and cerebral glucose metabolism. There is presently a rapid development of different PET tracers for visualizing tau pathology in brain. Tau PET demonstrates regional deposition of tau that follows the progression of AD but there is a heterogeneous propagation of tau pathology in brain seen in different patients with clinical symptoms. Although a positive correlation can be observed between Tau PET and cognition it seem that the regional glucose metabolism better reflecting the cognitive decline in AD being a mediator of taupathology on cognition.

## O26 PROPAGATION AND MATURATION OF ALZHEIMER'S DISEASE-RELATED PATHOLOGY

Dietmar R. Thal[1, 2, 3], Ajeet Rijal Upadhaya[3], Janina Gerth[4], Moritz Hecht[3], Lara Krämer[3], Sathish Kumar[4], Markus Otto[5], Christine von Arnim[5], Jochen Walter[4]
[1]Laboratory for Neuropathology – Department of Neuroscience, KU-Leuven, Leuven, Belgium; [2]Department of Pathology, UZ Leuven, Leuven, Belgium; [3]Laboratory for Neuropathology – Institute of Pathology, University of Ulm, Ulm, Germany; [4] Department of Neurology, University of Bonn, Bonn, Germany; [5] Department of Neurology, University of Ulm, Ulm, Germany
*Molecular Neurodegeneration* 2019, **14(Suppl 1):**INVITED

**Correspondence:** Dietmar R. Thal
**Background**
The extracellular deposition of the amyloid β-protein (Aβ) in Aβ plaques and the intracellular accumulation of abnormal phosphorylated τ-protein in neurofibrillary tangles represent the pathological hallmark lesions of Alzheimer's disease (AD). Vascular aggregates of Aβ are hallmark lesions of cerebral amyloid angiopathy (CAA) and associated with AD.
**Methods**
We studied 284 autopsy cases with and without clinical symptoms of AD. The degree of dementia (clinical dementia rating (CDR) score) was assessed. The anatomical expansion of Aβ and τ lesions, including CAA was analyzed based on anti-Aβ and anti-abnormal τ protein stained sections. In a subset of 74 cases we immunostained for AβN3pE and AβpS8. The presence of infarct lesions and cardiovascular risk factors was also assessed.
**Results**
The anatomical spreading of Aβ plaque pathology was accompanied by a stepwise accumulation of modified and non-modified forms of Aβ (B-Aβ plaque stages). The same sequence of Aβ aggregate maturation events was observed in CAA affected vessels (B-CAA stages). In addition to Aβ and τ-related factors influencing cognitive function, we found an association of the CDR score with hippocampal microinfarcts. The hippocampal microinfarcts were, thereby, associated with capillary CAA. The balance between parenchymal (B-Aβ plaque stage) and vascular Aβ maturation (B-CAA stage) was influenced by arterial hypertension.
**Conclusions**
AD is not only related to the anatomical spreading of its hallmark lesions and CAA but also with changes in the composition of the Aβ aggregates, i.e. Aβ aggregate maturation. The predominant maturation of Aβ aggregates in CAA is, thereby, presumably linked with arterial hypertension. Capillary CAA is associated with the development of hippocampal microinfarcts that may have impact on the cognitive status of AD patients presumably pointing to a vascular component of AD.
**Support**
Alzheimer Forschung Initiative (AFI) #10810; FWO-Odysseus program.

O27 MAPPING A PHENOTYPE TO APOE4 IN HUMANS

Henrietta M Nielsen
Department of Neurochemistry/Biochemistry and Biophysics, Stockholm University, Sweden

*Molecular Neurodegeneration* 2019, **14(Suppl 1):**INVITED

Individuals carrying the APOEε4 allele are at increased risk of developing Alzheimer's disease (AD) and dementia with Lewy bodies (DLB), however laboratory tools are lacking to identifty those APOEε4-carriers that will indeed develop disease. Carriers of the APOEε4 variant tend to exhibit low plasma apolipoprotein E (apoE) levels and previous studies have shown that low plasma levels of apoE were linked to higher risk of AD and all dementia. Furthermore, we have demonstrated that an increased relative ratio between the apoE4 and apoE3 isoforms in plasma of healthy APOE ε3/ε4 carriers was linked to gray matter volume reductions and glucose hypometabolism in several brain areas normally affected in AD. The CSF levels of apoE in APOEε4-carriers we found to be unaltered. Given the peripheral phenotype of low plasma apoE levels and that the liver is the main production site of apoE in the periphery we are focusing on detailing an APOEε4 peripheral phenotype. By use of data from liver biopsy RNA seq analyses, primary human hepatocytes and a large 42k antigen array screening for autoreactivities in the blood we are aiming to find phenotypical traits that can help to identify those APOEε4 carriers that will develop disease and those that will not Early identification of individuals that will develop sporadic AD/DLB based on their APOEε4 status is crucial for disease prevention studies.

O28 PATHOGENIC AND THERAPEUTIC PATHWAYS IN A MOUSE MODEL OF RETINAL ISCHEMIA/REPERFUSION INJURY

Abbot F. Clark, Byung-Jin Kim, Sean Silverman, Iok-Hou Pang
North Texas Eye Research Institute, U. North Texas Health Science Center, Ft. Worth, TX USA

*Molecular Neurodegeneration* 2019, **14(Suppl 1):**INVITED

**Correspondence:** Abbot F. Clark

**Background**

Retinal ischemia plays important roles in diabetic retinopathy, retinal vein/artery occlusion, and glaucoma. Glaucoma is a leading neurodegenerative disease affecting more than 70 million individuals worldwide. Ischemia/reperfusion (I/R) injury to the inner retina causes both morphological and functional damage to inner retinal neurons, including retinal ganglion cells (RGCs).

**Methods**

Retinal I/R was induced in C57BL/6J mice by unilateral elevation of intraocular pressure to 120 mmHg for 60 minutes, followed by reperfusion. Retinal damage was measured and quantified in vivo by SD-optical coherence tomography and electroretinography (ERG). Retinal morphology and protein expression were examined by H&E staining and immunofluorescence, respectively. Retinal reactive oxygen species (ROS) were detected in vivo using a new imaging technique using the chemiluminescent probe L-012. Systemic administration of SP600125 or apocynin evaluated the pathogenic roles of JNK activation and ROS in retinal damage, while C1q deficient mice examined the role of the complement system.

**Results**

Oxidative damage, JNK activation, and activation of the complement system play key roles in the pathogenesis of retinal I/R injury. Therapeutic inhibition of each of these pathways partially or totally protected the inner retina by preventing loss of neurons in the inner retina, including RGCs. These therapies also functionally protected the retina as determined by maintenance of ERG b-wave amplitudes and pattern ERG amplitudes.

**Discussion**

Inhibition of JNK activation by systemic administration of SP600125 or inhibition of ROS production by systemic administration of apocynin totally protected the retina from I/R injury indicating that these are the major pathogenic pathways causing retinal I/R injury.

**Conclusion**

The retina is an extension of the CNS that is easily accessible for analysis. A better understanding of the molecular pathogenesis of retinal I/R injury has identified promising new therapeutic opportunities to prevent or inhibit I/R damage to the retina and possibly other CNS neurons.

O29 BLOOD-BRAIN-BARRIER: STRUCTURE, FUNCTION AND PATHOLOGY

Berislav Zlokovic
University of Southern California, USA

*Molecular Neurodegeneration* 2019, **14(Suppl 1):**INVITED

Abstract not available

O30 THE PATHOPHYSIOLOGY OF DOMINANTELY INHERITED ALZHEIMER'S DISEASE AND BIOMARKER FINDINGS IN DIAN-TU PREVENTION TRIALS

Randall J. Bateman, Tammie Benzinger, Guoqiao Wang, Alison Goate, Martin Farlow, Steve Salloway, Susan Mills, Anna Santacruz, Chengjie Xiong, Clifford Jack, Robert Koeppe, Eric McDade, David B. Clifford for the DIAN-TU APT (http://DIAN.wustl.edu )

*Molecular Neurodegeneration* 2019, **14(Suppl 1):**INVITED

**Correspondence:** Randall J. Bateman

**Background**

Autosomal Dominant Alzheimer's Disease is a rare form of AD caused by mutations in APP, PSEN1, or PSEN2. The discovery of these mutations led to a molecular biology revolution for AD, enabling models to be developed and drugs targeting the earliest changes in AD. The Dominantly Inherited Alzheimer Network (DIAN) was established across leading AD centers to collaborate to better understand the clinical, cognitive and biomarker changes which occur in AD and to enable interventional trials. DIAN findings indicate that the AD process begins at least 15 to 20 years before symptom onset, providing a window of opportunity for secondary prevention efforts.

**Methods**

The Dominantly Inherited Alzheimer Network Trials Unit (DIAN-TU) launched an AD prevention trial in 2012 in a genetically defined population of dominantly inherited AD mutation carriers who are destined to get the disease with near 100% penetrance. Recruitment was completed in 2015 into two parallel drug arms in the DIAN-TU adaptive prevention trial (DIAN-TU APT) platform.

Baseline demographic, imaging biomarkers including MRI, PIB PET, AV45 PET, AV1451 PET were analyzed according to protocol. Measures were compared to prior findings in the DIAN observational study.

**Results**

The DIAN-TU APT has excellent completion rates of an extensive battery of biomarkers. A broad sample of baseline data collected to the highest standards available enable characterization of patients prior to and in the early phase of cognitive decline due to autosomal dominant AD with a multifaceted analysis including cognitive function as well as brain structure and brain imaging parameters relevant to AD. This data is available to qualified researchers to probe additional aspects of this condition. Findings of amyloid and tau PET as well as baseline characteristics will be reviewed.

**Conclusions**

Clinical prevention trials in the rare population of ADAD are feasible with extensive collections of biomarkers. Availability of comprehensive biomarker evaluations during trials enables quantification of the impact of interventions and establishment of surrogate and other biomarkers to accelerate development of highly effective interventions for AD.
https://dian.wustl.edu/our-research/clinical-trial/study-sites/

O31 APOE4-BASED THERAPY FOR ALZHEIMER'S DISEASE
Chengzhong Wang, Ramsey Najm, Yadong Huang
Gladstone Institute of Neurological Disease, San Francisco, CA 94158, USA; Biomedical Sciences Graduate Program and Departments of Neurology and Pathology, University of California, San Francisco, CA 94143, USA
*Molecular Neurodegeneration* 2019, **14(Suppl 1):**INVITED

**Correspondence:** Yadong Huang
**Background**
Efforts to develop drugs for Alzheimer's disease (AD) have shown promise in animal studies, only to fail in human trials, suggesting a pressing need to study AD in human model systems.
**Methods**
Using human neurons derived from induced pluripotent stem cells carrying the major genetic risk factor apolipoprotein E4 (apoE4) and gene-edited isogenic lines to study AD pathogenesis and screen for small-molecule compounds targeting apoE4's detrimental effects.
**Results**
We demonstrate that apoE4 neurons have higher levels of tau phosphorylation unrelated to their increased Aβ production and displayed GABAergic neuron degeneration. ApoE4 increased Aβ production in human, but not in mouse, neurons. Converting apoE4 to apoE3 by gene editing rescued these phenotypes, indicating the specific effects of apoE4. Neurons lacking apoE behaved like those expressing apoE3, and introducing apoE4 expression recapitulated the pathological phenotypes, suggesting a gain of toxic effects from apoE4. Interaction between the carboxyl-terminal and the amino-terminal domains, called domain interaction, is a unique biophysical property of apoE4. Domain interaction has been suggested to be a molecular basis for apoE4's detrimental effects in AD pathogenesis, and consequently has been pursued as a drug target to identify small molecule structure correctors capable of converting apoE4 to apoE3 both structurally and functionally. Treating apoE4 neurons with a small-molecule structure corrector ameliorated AD-related detrimental effects, providing a proof of concept that correcting the pathogenic conformation of apoE4 is a viable therapeutic approach for apoE4-related AD.
**Conclusions**
Our data indicate that apoE4 induces AD-related pathological phenotypes, due to a gain of toxic effects, specifically in human neurons, which can be dramatically ameliorated by treatment with a small-molecule apoE4 structure corrector. These findings warrant further development of apoE4 structure correctors and, ultimately, testing in clinical trials.

O32 ANTI-ABETA IMMUNOTHERAPY
Roger Nitsch
University of Zurich, Switzerland
*Molecular Neurodegeneration* 2019, **14(Suppl 1):**INVITED

Abstract not avaiable

## SHORT
## TALKS

S1 MOLECULAR CHARACTERIZATION OF GENDER DIFFERENCES IN AD PATHOGENESIS
Dongming Cai[1,2], Jiqing Cao[1], Lei Guo[3], Li Zhu[1,2], Minghui Wang[3], Vahram Harountunian[2,4], Bin Zhang[3]
[1]Department of Neurology, Icahn School of Medicine at Mount Sinai, New York, NY, USA; [2]Research & Development, James J Peters VA Medical Center, Bronx, NY, USA; [3]Department of Genetics and Genomic Sciences, Institute of Genomics and Multiscale Biology, Icahn School of Medicine at Mount Sinai, New York, NY, USA; [4]Department of Psychiatry, Icahn School of Medicine at Mount Sinai, New York, NY, USA
*Molecular Neurodegeneration* 2019, **14(Suppl 1):**SHORT

**Correspondence:** Dongming Cai
**Background**
The burden of Alzheimer's disease (AD) at the patient level falls disproportionately on females, as many studies find that age-matched females have a higher proportion of AD cases [1-4]. Surprisingly, few if any studies have focused on the genetic and gene expression mechanisms that mediate the apparent gender differences in AD presentation.
**Methods**
To address this gap, we gathered currently available gene expression study of 1400+ postmortem human brain samples, and performed an extensive quality control and covariate correction of the assembled data to ensure that gender status is annotated correctly and that covariates such as age do not confound our analyses. We then utilized this cleaned and aggregated data set to construct gender-specific multiscale networks of AD [5].
**Results**
We systematically searched for genes and pathways that differentiate AD progression between females and males, as well as ApoE4 carriers and non-carriers. We then generalized the analysis to the network level [5-7], which allows us to detect higher-order trends and identify target genes that drive major differences in AD progression between females and males. After integrating large-scale molecular data and known gene regulatory relationships, we prioritized 24 genes for further characterization. We then validated the differences in expression levels of selected key drivers using additional postmortem human brain samples to confirm the biological relevance of the findings.
**Discussion**
We will next investigate functional roles of validated key targets in AD pathogenesis using gene manipulation methods in male and female AD mouse models.
**Conclusions**
Our studies will lead to a better understanding of AD biology working towards a comprehensive understanding of the molecular mechanisms underlying sex differences in AD pathogenesis. It will also pave a path towards distinct targeted drug discovery efforts for AD in females and males.

**References**
1. Plassman, B. L. et al. Prevalence of dementia in the United States: the aging, demographics, and memory study. Neuroepidemiology (2007) 29, 125-132.
2. Hebert, L. E., Scherr, P. A., McCann, J. J., Beckett, L. A. & Evans, D. A. Is the risk of developing Alzheimer's disease greater for women than for men? American journal of epidemiology (2001) 153, 132-136.
3. Miech, R. A. et al. Incidence of AD may decline in the early 90s for men, later for women: The Cache County study. Neurology (2002) 58, 209-218.

S2 EVIDENCE FOR OLIGOGENIC INHERITANCE IN PARKINSON DISEASE DUE TO HETEROZYGOUS MUTATIONS IN MULTIPLE AUTOSOMAL RECESSIVE GENES ASSOCIATED WITH PARKINSON DISEASE AND RELATED DISORDERS
Stefanie Smolders[1,2], David Crosiers[1,2,3], Patrick Cras[2,3], Christine Van Broeckhoven[1,2], on behalf of the BELNEU Consortium
[1]VIB Center for Molecular Neurology, University of Antwerp, Antwerp, Belgium; [2]Institute Born-Bunge, University of Antwerp, Antwerp, Belgium; [3]Department of Neurology, Antwerp University Hospital, Edegem, Belgium
*Molecular Neurodegeneration* 2019, **14(Suppl 1):**SHORT

**Correspondence:** Stefanie Smolders
**Background**
To date, 4 genes are known to cause autosomal recessive Parkinson Disease (PD): PARK2, PINK1, DJ-1 and VPS13C. Notably, a high prevalence of rare single heterozygous mutations in autosomal recessive PD genes and the presence of compound mutations in two different autosomal recessive PD genes are frequently reported in PD patients. These observations imply a role for several genetic factors in disease etiology.
**Methods**
37 PD patients with a single rare heterozygous mutation in an autosomal recessive PD gene were subjected to whole exome sequencing (WES) to identify additional rare variants explaining the observed phenotype. Heterozygous variants in autosomal recessive genes associated with PD, atypical parkinsonian syndromes and related movement/neurodegenerative brain disorders were prioritized based on quality, frequency in public databases and impact (splice site and non-synonymous variants with Combined Annotation Dependent Depletion (CADD) score >20 [1]).

**Results**

WES data analysis revealed the presence of oligogenic inheritance through known pathogenic and rare novel heterozygous mutations in multiple associated genes. We identified 2 PD patients with compound missense variants in PARK2/PINK1 and 2 PD patients with compound missense variants in PARK2/VPS13C. Additionally, we found one carrier of PARK2 p.Q34Rfs*4, VPS13C p.I3726V/p.R2482H and the pathogenic GBA variant p.L434P, one carrier of PARK2 p.P437L and SLC17A2 p.Y306*, and one carrier of compound frameshift mutations in PARK2/POLR3B.

**Discussion**

Oligogenic inheritance of autosomal recessive PD genes could be explained by their essential roles in common mitochondrial quality control and oxidative stress pathways. The identification of pathogenic mutations in GBA and SLC17A2 highlights the importance of lysosomal mechanisms in PD pathogenesis and confirms overlapping pathomechanisms between PD and lysosomal storage disorders. Moreover, compound frameshift mutations in PARK2/POLR3B could imply a common pathway in leukodystrophy and PD pathogenesis.

**Conclusions**

Our results underpin the potential oligogenic complexity of Mendelian genes in PD etiology.

## S4 GWAS-BASED MOUSE MODELS EXHIBIT DISTINCT TRANSCRIPTOMIC SIGNATURES OF LATE-ONSET ALZHEIMER'A DISEASE

Ravi S. Pandey[1], Leah Graham[1], Asli Uyar[1], Kristen D. Onos[1], Christoph Preuss[1], Harriet M. Williams[1], Cai John[1], Kelly J. Keezer[1], Casey J. Acklin[1], Michael Sasner[1], Benjamin Logsdon[1], Gareth R. Howell[1], Gregory W. Carter[1]
[1]The Jackson Laboratory, Bar Harbor, ME, USA; [2]Sage Bionewtorks, Seattle, WA, USA
*Molecular Neurodegeneration* 2019, **14(Suppl 1):**SHORT

**Correspondence:** Gregory W. Carter

**Background:** Genome-wide molecular assays such as RNA-seq are enabling detailed characterization of Alzheimer's disease (AD) pathology in clinical cohorts. In parallel, advances in human genetics and rapid genetic engineering technology are facilitating creation of the next generation of animal models. We compared transcriptomes from human studies and mouse models to identify the subpathologies captured by each mouse model.

**Methods**

RNA-seq was performed on a panel of transgenic and knockout mouse models of Alzheimer's disease in a range of genetic backgrounds, created in the Model Organism Development and Evaluation for Late-onset AD (MODEL-AD) consortium. These include alleles of APOE, TREM2, APP, and other AD-associated genes. Unsupervised data clustering was performed with weighted gene correlation network analysis (WGCNA) to identify modules of co-expressed genes.

**Results**

Gene modules were associated with distinct mouse models. Neuroinflammation, neurometabolism, synaptic signaling, and protein maintenance were among the enriched functions of these modules, matching existing gene modules similarly derived in the Accelerating Medicines Partnership for AD (AMP-AD) consortium.

**Discussion**

Distinct mouse models recapitulate specific endophenotypic pathologies observed in AD populations. This provides evidence that specific genetic perturbations lead to alterations in key biological pathways and processes. We highlight how models based on early and late onset AD risk genes differentially reflect early- and late-onset AD human data.

**Conclusions:** Our results specify which mouse models are appropriate for studying pathway-level alterations in AD patients, guiding research efforts and preclinical testing in optimal models. Furthermore, the controlled genetic perturbations allow for causal hypotheses of which pathways are altered by specific genetic variants and, in turn, suggest how they drive late-onset AD.

## S5 PLASTICITY OF THE ENTORHINAL-HIPPOCAMPAL CIRCUIT AS A VULNERABILITY IN ALZHEIMER'S DISEASE

Stacy D. Grunke[1], Anand K. Singh[1], Rong Zhao[1], Konstantinos Sousounis[1], Kyung-Won Park[1], Joanna L. Jankowsky[1, 2, 3, 4, 5]
-Departments of Neuroscience; [2]Neurology; [3]Neurosurgery; [4]Molecular and Cellular Biology; [5]Huffington Center on Aging, Baylor College of Medicine, Houston, TX 77030
*Molecular Neurodegeneration* 2019, **14(Suppl 1):**SHORT

**Correspondence:** Joanna L. Jankowsky

**Background**

Layer 2 neurons in the entorhinal cortex are among the first cells to degenerate in Alzheimer's disease, but the basis for their vulnerability is completely unknown. These cells form the main cortical input to the hippocampal tri-synaptic loop responsible for lifelong memory consolidation. This function requires an unusual degree of ongoing plasticity that may bestow susceptibility unique to this circuit.

**Methods**

We used a chemogenetic system to perturb circuit homeostasis in three neural populations affected by neurodegenerative disease (entorhinal layer 2, nigral dopaminergic neurons, and cerebellar Purkinje cells). In each population, a subset of neurons was electrically inactivated by systemic administration of a synthetic ligand gating a transgenically-expressed ion channel. At varying times after neural silencing, animals were harvested to evaluate structural changes evoked by acute imbalance of circuit function.

**Results**

Using this model, we unexpectedly found that entorhinal neurons appear to be highly sensitive to inactivity. Unlike neural populations affected in other neurodegenerative diseases, entorhinal neurons underwent cell death following even acute bouts of experimentally-induced electrical arrest. In the days and weeks immediately following chemogenetic silencing, entorhinal axons retracted from the dentate gyrus, activated caspase appeared in the soma, neighboring microglia became reactive, and 30-50% of the silenced neurons ultimately disappeared.

**Discussion**

This patterned degeneration in the adult cortex is reminiscent of activity-dependent process used by the developing brain to sculpt emerging circuits. While it was long believed that the critical period for wholesale structural remodeling closed during early postnatal life, we propose that the entorhinal cortex is among a handful of areas which maintain the potential for substantial modification throughout adulthood.

**Conclusions**

Based on our findings, we hypothesize the ongoing plasticity required to support learning and memory throughout life also renders neurons in the entorhinal-hippocampal circuit vulnerable to activity dependent competition for survival in the adult.

7. Tuo QZ, Lei P, Jackman KA, Li XL, Xiong H, Li XL, Liuyang ZY, Roisman L, Zhang ST, Ayton S, et al: Tau-mediated iron export prevents ferroptotic damage after ischemic stroke. Mol Psychiatry 2017, 22:1520-1530.

## S6 THE INVOLVEMENT OF IRON IN THE PATHOGENESIS OF ALZHEIMER'S DISEASE

Peng Lei[1], Scott Ayton[2], Qing-zhang Tuo[1], Ashley Bush[2]
[1]State Key Laboratory of Biotherapy, West China School of Basic Medical Sciences & Forensic Medicine, Sichuan University, Sichuan 610041, China; [2]Florey Institute of Neuroscience & Mental Health, the University of Melbourne, Parkville 3052, Australia
*Molecular Neurodegeneration* 2019, **14(Suppl 1):**SHORT

**Correspondence:** Peng Lei

**Background**

No disease-modifying drug currently exists for neurodegenerative disorders such as Alzheimer's (AD) and Parkinson's diseases (PD). While iron has been implicated in neurodegeneration for many years, the extent to which iron elevation contributes to pathogenesis, and the origin of its elevation, had remained unknown.

**Methods**

A range of cell culture and animal experiments were conducted under appropriate regulations. The use of human brain tissues and data were approved by various ethics committee.

**Results**

We demonstrated that the AD-implicated amyloid precursor protein (APP) binds to the iron exporting protein, ferroportin, tethering it to the membrane for efficient iron efflux [1]. We also showed that the ADimplicated tau protein maintains neuronal iron homeostasis by facilitating APP trafficking to the cell surface [2,3], and the ferroxidase ceruloplasmin, recruited from astrocytes, is involved in neuronal iron release[4]. We also quantified the contribution of iron on progression of AD, and revealed that the iron burden of the brain has a similar magnitude impact on longitudinal (7 years) outcomes of AD (cognition, brain atrophy) compared to more established factors in the disease (e.g. CSF tau and Aβ)[5, 6]. By using ischemia-related cognitive impairment model, we found that unilateral, transient middle cerebral artery occlusion (MCAO) suppressed hemispheric tau and increased iron levels in young (3 month old) mice and rats, and such cognitive impairment were protected by iron-targeted interventions: ceruloplasmin (Cp), amyloid precursor protein ectodomain (APPec), as well as ferroptosis inhibitors[7].

**Conclusions**

Pre-clinical and clinical studies demonstrate the potential of iron to contribute to disease progression and iron presents as an unexplored prognostic, and tractable therapeutic target of neurological disorders.

## S7 DYSREGULATION OF PROTEIN PRENYLATION AND RAS SIGNALING IN ALZHEIMER'S DISEASE

Angela Jeong[1], David A. Bennett[2], Mark Distefano[3], Ling Li[1]
[1]Department of Experimental and Clinical Pharmacology, University of Minnesota, Minneapolis, MN, USA; [2]Rush Alzheimer's Disease Center, Rush University, Chicago, IL, USA; [3]Department of Chemistry, University of Minnesota, Minneapolis, MN, USA
*Molecular Neurodegeneration* 2019, **14(Suppl 1):**SHORT

**Correspondence:** Ling Li

**Background**

Alzheimer's disease (AD) is the most prevalent neurodegenerative disorder and its pathogenesis is not fully understood. Emerging evidence indicates that protein prenylation, a posttranslational lipid modification process catalyzed by farnesyl and geranylgeranyl transferases, may play an important role in AD [1-3]. Many proteins, including the Ras superfamily of small GTPases, undergo prenylation [4]. These small GTPases serve as molecular switches in signal transduction pathways, regulating diverse cellular processes and functions [5]. Modulation of protein prenylation influences multiple aspects of neuropathology of AD [1-3]. Recently, we have demonstrated that downregulation of protein prenylation reduces neuropathology in a transgenic mouse model of AD, although only farnesyltransferase haplodeficiency rescues cognitive function [6]. The present study aims to determine the dynamic changes of prenylated proteins and related signaling molecules in human brains and to evaluate whether these changes are associated with cognition and AD-neuropathology.

**Methods**

Postmortem frozen tissue from the dorsolateral prefrontal cortex was obtained from participants in the Religious Orders Study, which included human subjects with a spectrum of cognition from normal, mild cognitive impairment (MCI), to AD dementia. The brain tissue samples were subjected to subcellular fractionation and immunoblotting analysis.

**Results**

We found that the level of membrane-associated H-Ras, an exclusively farnesylated protein, was significantly increased in the brains from individuals with MCI and AD compared with individuals with normal cognition. Further, the level of farnesylated H-Ras correlated with tangle pathology and the activation of ERK, a major downstream effector of H-Ras. Consistent with the elevation of membrane-associated H-Ras, the level of farnesyltransferase was increased in AD brains.

**Conclusions**

These findings suggest that upregulation of protein farnesylation is an early event with primary importance in the pathogenic cascade of AD and activation of downstream signaling pathways contributes to the development of cognitive impairment and neuropathology in AD.

S8 VIRAL BRAIN EXPRESSION OF APP C-TERMINAL FRAGMENT PRESERVES MEMORY IN ALZHEIMER'S DISEASE MOUSE MODELS

**Correspondence:** Angèle T. Parent

**Background**

Our study explores the amyloid precursor protein (APP) as a whole cellular component that might influence neuronal function and impact the course of AD [1]. We previously reported that the in vitro accumulation of APP C-terminal fragment (CTF) caused by overexpression of membrane-tethered APP intracellular domain (mAICD) favors axodendritic outgrowth as a result of direct coupling with GαS and subsequent activation of adenylate cyclase and CREB signaling [2, 3]. PKA-dependent and associated CREB signaling strongly correlate with synaptic enhancement and memory consolidation. As a proof of concept, we tested if recombinant adeno-associated virus (rAAV)-mediated expression of mAICD could alleviate memory deficits in an AD mouse model.

**Methods**

We generated mAICD construct and mAICD variant lacking the GαS interaction site. We used neonatal rAAV brain delivery to achieve high and prolonged in vivo brain expression of mAICD in the 5XFAD transgenic mouse model of amyloidosis. We subjected 5XFAD mice to memory behavior tasks at 6-months and analyzed the Aβ burden.

**Results**

Our results show that mAICD expression affects spatial working memory as depicted by an improvement in novel object recognition and spontaneous alternation in 5XFAD mice. The expression of mAICD construct lacking the GαS-protein interaction site did not have this outcome. Immunohistochemical analysis revealed that mAICD expression produced a significant decrease of Aβ deposition in the hippocampus. Our findings also indicate that G-protein signaling mediated by mAICD facilitates axodendritic outgrowth and the accumulation of APP at the cell surface.

**Discussion**

We have identified the significance of APP-CTF and its associated signaling partners in contributing to cognitive function and amyloidogenic cascade. Our results demonstrate that mAICD-mediated signaling events could alter APP processing, reduce Aβ burden, and enhance memory process in AD mouse models.

**Conclusions**

mAICD expression favors non-amyloidogenic processing of APP and rescues cognitive deficit in AD mice.

S9 MUSCLEBLIND IS A NOVEL MODIFIER OF FUS-MEDIATED NEURODEGENERATION IN VIVO

**Correspondence:** Udai Bhan Pandey

**Background**

Amyotrophic lateral Sclerosis (ALS) is a devastating motor neuron disease involving the progressive loss of neurons in the brain and spinal cord. Mutations of the gene Fused in Sarcoma (FUS), which codes for the protein FUS, have been linked with ALS pathogenesis. FUS is a DNA/RNA-binding protein that plays critical roles in RNA metabolism including RNA trafficking and alternative splicing.

**Methods**

To identify dominant modifiers of FUS-associated neurodegeneration, we performed an unbiased genetic screen in our fly model of ALS followed by in-depth validation in mammalian neuronal models and FUS patient-derived iPSC motor neurons.

**Results**

Unexpectedly, we identified muscleblind (mbl), the Drosophila homolog of human muscleblind-like (MBNL), as a novel suppressor of FUS-mediated neurodegeneration in vivo. RNAi-mediated knockdown of endogenous Drosophila mbl rescues neurodegenerative phenotypes such as retinal degeneration, reduced life span and neuromuscular junction defects caused by pathogenic mutations in FUS. Ectopic expression of muscleblind strongly enhanced FUS toxicity in vivo. We observed that FUS and muscleblind physically interact in mammalian cells. Muscleblind is a strong suppressor of dendritic morphological defects and toxicity in mammalian neurons. Interestingly, muscleblind is a component of cytoplasmic stress granules in mammalian neuronal cells. We observed that muscleblind protects against FUS toxicity by promoting degradation of stalled cytoplasmic stress granules in mammalian neurons and

ALS patient motor neurons. To understand the molecular mechanisms of mbl mediated suppression, we performed RNA sequencing using Drosophila brains expressing WT or mutant FUS with or without mbl. We identified several predominantly nuclear genes whose expression is altered by FUS expression, and subsequently returned to almost normal following knockdown of endogenous mbl.

**Conclusions**

Our data suggests an unexpected function of mbl in FUS-mediated neurodegeneration and demonstrates that muscleblind is a regulator of toxicity associated with FUS in Drosophila, primary mammalian neurons and patient motor neurons.

S10 UNRAVELING AMYLOID TOXICITY: FROM STRUCTURE TO FUNCTION

Karen Marshall, Devkee Vadukul, Saskia Pollack, Luca Biasetti, Lenzie Ford, Mahmoud Bukar Maina, Youssra Al-Hilaly, Louise Serpell
School of Life Sciences, University of Sussex, Falmer, Brighton, BN1 9QG, UK
*Molecular Neurodegeneration* 2019, **14(Suppl 1):**SHORT

**Correspondence:** Louise Serpell

**Background**

Many proteins and peptides with different primary sequences share the ability to self-assemble to form amyloid fibrils. A large number of these have been implicated in protein misfolding diseases but many perform functional roles in living organisms [1]. Aβand tau are key proteins that have the ability to selfassemble and are deposited in brains of patients with Alzheimer's disease.

**Methods**

Using a unique combination of molecular biophysics, structural biology and cell biology, we have explored the relationship between sequence, amyloidogenicity and toxicity.

**Results**

A variant of the Alzheimer's Aβ peptide has been designed in order to examine the specific structural variations that lead to Aβ toxicity [2]. Key differences have been identified in the aggregation propensity, which are closely linked to cellular uptake and functional effects including memory loss in a model organism. Oxidative stress results in the formation of dityrosine cross-linked Aβ and tau related to the neurodegeneration observed in Alzheimer's disease, highlighting a key role for tyrosine in amyloidogenic proteins [3]. A truncated form of tau is able to self-assemble to form bona-fide paired helical filament, providing an ideal model system for toxicity studies [4].

**Discussion**

Here we will describe how these striking observations have led to insights into the mechanism of Aβ and tau toxicity.

**Conclusions**

These studies are providing a platform to better understand deleterious effects of oligomeric proteins in disease and how amyloid fibrils are controlled for functional, non-toxic roles.

S11 EPIGENETIC MICROGLIAL MEMORY OF PERIPHERAL INFLAMMATION SHAPES NEUROLOGICAL DISEASE

**Correspondence:** Ann-Christin Wendeln

**Background**

Innate immune memory is a vital mechanism of myeloid cell plasticity that occurs in response to environmental stimuli and alters subsequent immune responses. Two types of immunological imprinting can be distinguished, training and tolerance, which are mediated by epigenetic mechanisms and enhance or suppress subsequent inflammation, respectively. Whether immune memory occurs in tissue-resident macrophages in vivo and how it may affect pathology remains largely unknown.

**Methods**

Peripheral immune stimulation with lipopolysaccharides was applied to mouse models of cerebral β-amyloidosis and stroke. Brain tissue was subsequently analyzed histologically and biochemically. Microglia were isolated from immune stimulated mice and analyzed by RNA-sequencing and ChIP-sequencing to assess their transcriptome and enhancer repertoire.

**Results**

We demonstrate that peripherally applied inflammatory stimuli induce acute immune training and tolerance in the brain. Strikingly, in a mouse model of Alzheimer's pathology, immune training exacerbates while tolerance alleviates cerebral β-amyloidosis; similarly, peripheral immune stimulation modifies pathological features after stroke. Training and tolerance lead to differential epigenetic reprogramming of brain-resident macrophages, microglia, that persists for at least six months and impacts microglial gene expression and function.

**Discussion**

Our results provide first evidence for innate immune memory in tissue-resident macrophages. Long-lasting functional changes of microglia based on epigenetic alterations in response to peripheral immune stimuli impact pathological features of cerebral β-

amyloidosis and stroke, indicating epigenetic microglial reprogramming as an important modifier of brain pathology.

**Conclusions**

Peripheral inflammatory stimuli induce innate immune memory effects in the brain and lead to long-lasting epigenetic reprogramming of microglia, thereby modulating later occurring neuropathology.

## S12 THE INVESTIGATIONAL STEREOPURE ANTISENSE OLIGONUCLEOTIDE WVE-3972-01 PREFERENTIALLY REDUCES G4C2 REPEAT-CONTAINING C9ORF72 TRANSCRIPTS: A POTENTIAL THERAPEUTIC APPROACH FOR THE TREATMENT OF AMYOTROPHIC LATERAL SCLEROSIS (ALS) AND FRONTOTEMPORAL DEMENTIA (FTD)

Yuanjing Liu1, Jean-Cosme Dodart1, Helene Tran2, Shaunna Berkovitch1, Michael Byrne1, Ann Durbin1, Naoki Iwamoto1, Hyun Gyung Jang1, Pachamuthu Kandasamy1, Susovan Mohapatra1, Hailin Yang[1], Yuan Yin[1], Jason Zhang[1], Zhong Zhong[1], Chandra Vargeese[1], Robert H. Brown, Jr.[2]
[1]Wave Life Sciences, Cambridge, MA, USA; [2]Department of Neurology, University of Massachusetts, Worcester, MA USA
*Molecular Neurodegeneration* 2019, **14(Suppl 1):**SHORT

**Correspondence:** Zhong Zhong

**Background**

A large hexanucleotide GGGGCC (G4C2) repeat expansion in the first intronic region of the C9ORF72 gene is the most common genetic cause of ALS and FTD. The G4C2 repeat expansion reduces the normal expression of the C9ORF72 gene, causing the production of mutant repeat-containing transcripts that form pathogenic nuclear RNA foci and dipeptide repeat (DPR) proteins.

**Methods**

Using a luciferase reporter assay and patient-derived induced pluripotent stem cell (iPSC) neurons, antisense oligonucleotide (ASO) sequences that preferentially reduced repeatcontaining transcripts compared to the total transcripts were identified. In vitro activity of the lead candidate, WVE-3972-01, was then confirmed under transfection conditions in patient-derived fibroblasts and under gymnotic conditions in neuronal cultures. In vivo efficacy and distribution studies were performed in transgenic mice carrying a human bacterial artificial chromosome (BAC) construct containing the human C9ORF72 gene with a repeat expansion, referred to as C9BAC mice.

**Results**

Optimization of ASO chemistry and the chirality of the phosphorothioate backbone resulted in potent stereopure ASOs, with sub-nanomolar activity in the reporter assay, and nanomolar activity in C9-ALS patient-derived fibroblasts and neurons, as well as in primary neurons from C9BAC mice. Stereopure ASOs, including the lead candidate WVE-3972-01, demonstrated improved in vitro metabolic stability compared to stereorandom ASOs in mouse brain homogenates. Intracerebroventricular injection of WVE-3972-01 into C9BAC mice resulted in substantial and sustained reduction of repeat-containing C9orf72 transcripts, RNA foci, and DPR proteins, without altering total C9orf72 protein levels.

**Discussion**

WVE-3972-01 specifically targets transcripts that contain the G4C2 repeat expansion in the first intronic region of the C9ORF72 gene, thereby preventing production of toxic RNA foci and DPR proteins with minimal alteration of total C9orf72 protein levels.

**Conclusions**

Results suggest that preferential targeting of repeat-containing transcripts using stereopure ASOs may be a viable therapeutic approach for the treatment of ALS and FTD.

## S13 TYPE I INTERFERON-MEDIATED NEUROINFLAMMATORY PROGRAM IN ALZHEIMER'S DISEASE

Ethan Roy, Baiping Wang, Hui Zheng, Wei Cao
Huffington Center on Aging, Department of Molecular and Human Genetics, Baylor College of Medicine, Houston, Texas, USA
*Molecular Neurodegeneration* 2019, **14(Suppl 1):**SHORT

**Correspondence:** Wei Cao

**Background**

The cytokine family type I interferon (IFN) is a major innate immune mediator extensively studied in the peripheral immune responses but largely under-investigated in AD. Previously, we established that innate immune cells readily produce IFN in response to amyloid fibrils containing nucleic acids as cofactor (1, 2). Here, we investigated whether IFN pathway is associated with amyloidosis in AD brain and contributes to neuroinflammation.

**Methods**

We systemically characterized neuroinflammation in multiple murine AD models by gene expression analysis and explored an archived human AD brain database. Hippocampal slice culture was stimulated with different forms of amyloid fibrils and analyzed for inflammatory responses. In addition, stereotaxic injections were performed to evaluate the IFN-mediated neuroinflammation and effects of an IFN receptor neutralizing antibody in vivo.

**Results**

We established a comprehensive core AD neuroinflammation profile that includes several key proinflammatory cytokine families, among which IFN pathway is consistently activated. Nucleic acid containing amyloid fibrils, but not heparin-containing fibrils, potently stimulated comprehensive neuroinflammation in hippocampal slice culture. Furthermore, IFNβ induced an immune response in the brain of wild type mice analogous to the core neuroinflammatory profile associated with Aβ pathology; whereas IFN receptor blockade significantly blunted the ongoing reactive gliosis and complement C3 expression in APP knock-in mice. Moreover, genes stimulated by IFN are grossly upregulated in human AD brains, where coregulation of IFN pathway and C3 is evident.

**Conclusions and Discussion**

We have demonstrated that type I interferon constitutes a major pathway within the neuroinflammatory network of AD. Given its observed detrimental effects on CNS functions (3-6), IFN may represent a molecular target to restrain the pathogenic inflammatory response associated with AD.

## S14 PERIPHERAL APOE IMPACTS CNS COGNITION AND ALZHEIMER'S PATHWAYS

Chia-Chen Liu[1], Yuanxin Chen, Na Zhao[1], Yuan Fu[1], Yingxue Ren2, Wenhui Qiao[1], Na Wang[1], Justin Rogers1, Jing Zhao[1], Cynthia Mencia Linares[1], Joshua Knight[1], Lindsey Felton[1], Aishe Kurti[1], John Fryer[1], Yan W. Asmann[2], Betty Kim[1], Guojun Bu[1]
[1]Department of Neuroscience, Mayo Clinic, Jacksonville, FL, USA;
[2]Division of Biomedical Statistics and Informatics, Department of Health Sciences Research, Mayo Clinic, Jacksonville, FL, USA
*Molecular Neurodegeneration* 2019, **14(Suppl 1):**SHORT

**Correspondence:** Chia-Chen Liu

**Background**

The ε4 allele of the apolipoprotein E (APOE) gene is the strongest genetic risk factor for lateonset Alzheimer's disease (AD) [1]. ApoE4 promotes Aβ aggregation and deposition and is associated with impaired brain lipid homeostasis, glucose metabolism, vascular functions and increased neuroinflammation [2,3]. Thus, understanding the pathobiology of apoE4 represents a great opportunity to both uncover mechanisms underlying AD risk and also explore new strategies for AD therapy. ApoE is abundantly expressed in the brain and in periphery. In fact, apoE concentration in plasma is about 10 times higher than that in the cerebral spinal fluid [4]. As peripheral apoE, produced mainly by the liver, is separated from brain apoE by the blood-brain barrier (BBB) [5, 6], it is not clear whether and how peripheral apoE affects the function of the central nervous system (CNS) and AD pathogenesis.

**Methods**

We have developed novel mouse models expressing human apoE3 or apoE4 in an inducible, cell type-specific manner. After breeding to albumin-Cre (Alb-Cre) mice which drive apoE expression specifically in the liver, we have generated human apoE3 and apoE4 liver-specific mouse models in the background of murine Apoe-KO in the presence or absence of amyloid pathology.

**Results**

We found that expression of apoE3 in the periphery in the absence of brain apoE enhanced synaptic activity and cognition, whereas expression of apoE4 did the opposite. In addition, we found that peripherally expressed apoE isoforms differentially impacted neuroinflammation and amyloid pathology. Gene expression profiling also identified molecular pathways contributing to potential mechanisms.

**Discussion**

Our results for the first time provide clear evidence that apoE4 in the periphery also impacts brain functions and AD pathologies, raising the possibility that enhanced AD risk associated with APOE4 likely involves contribution from apoE4 protein in both brain and periphery.

**Conclusions**

Our findings demonstrate that peripheral apoE isoforms have differential effects on CNS functions and AD-related pathways.

## S15 CEREBROSPINAL FLUID NEUROGRANIN CONCENTRATION IN RELATION TO ALZHEIMER'S DISEASE AND OTHER NEURODEGENERATIVE PATHOLOGIES

Erik Portelius[1, 2], Bob Olsson[1, 2], Kina Höglund[1, 2], Hlin Kvartsberg[1], Ulf Andreasson[1, 2], Henrik Zetterberg[1, 2, 3, 4], Åsa Sandelius1, Leslie M. Shaw[5], Virginia M.Y. Lee5, David J. Irwin6, Murray Grossman6, Daniel Weintraub[7,8], Alice Chen-Plotkin[6], David A. Wolk[6], Leo McCluskey[6], Lauren Elman[6], Jennifer McBride[5], Jon B. Toledo[5,9], John Q. Trojanowski[5], Kaj Blennow[1, 2]
[1] Institute of Neuroscience and Physiology, Department of Psychiatry and Neurochemistry, the Sahlgrenska Academy at the University of Gothenburg, Mölndal, Sweden; [2] Clinical Neurochemistry Laboratory, Sahlgrenska University Hospital, Mölndal, Sweden; [3] Department of Molecular Neuroscience, UCL Institute of Neurology, Queen Square, WC1N London UK; [4] UK Dementia Research Institute, WC1N London UK; [5] Department of Pathology and Laboratory Medicine, Institute on Aging, Center for Neurodegenerative Disease Research, University of Pennsylvania School of Medicine, Philadelphia, PA, USA; [6] Department of Neurology, University of Pennsylvania School of Medicine, Philadelphia, PA; [7] Department of Psychiatry, University of Pennsylvania School of Medicine, Philadelphia, PA; [8] Parkinson's Disease and Mental Illness Research, Education and Clinical Centers (PADRECC and MIRECC), Philadelphia Veterans Affairs Medical Center, Philadelphia, PA; [9] Department of Neurology, Houston Methodist Hospital, Houston, TX
*Molecular Neurodegeneration* 2019, **14(Suppl 1):**SHORT

**Correspondence:** Erik Portelius

**Background**

The post-synaptic protein neurogranin (Ng) is expressed in specific brain regions, including hippocampus and amygdala. Recent studies have shown that the concentration of Ng is increased in cerebrospinal fluid (CSF) from Alzheimer's disease (AD) patients, but no study has evaluated CSF Ng in neuropathologyconfirmed cases. Further, the relation to amount of plaque and tangle load has not been investigated.

**Methods**

An enzyme-linked immunosorbent assay for quantification of CSF Ng was developed and the performance of Ng as a marker was investigated in 915 patients with different neurodegenerative diseases. Of these 915 patients, 115 had a neuropathologically confirmed definitive diagnosis. The relation of CSF Ng to neuropathology was evaluated across several different diseases.

**Results**

The CSF Ng concentration was significantly increased in AD compared to e.g., Parkinson's disease, frontotemporal dementia and amyotrophic lateral sclerosis. Neuropathology data showed that a greater plaque and tangle load was positively associated with higher CSF Ng concentrations. In contrast, in the hippocampus and amygdala, the concentration of CSF Ng was not associated with α-synuclein or TAR DNA-binding protein 43 pathology load.

**Discussion**

CSF Ng concentrations were significantly higher in AD than in several other neurodegenerative disorders, also in neuropathologically confirmed cases. Further, higher CSF Ng concentrations correlated with higher amounts of plaques in the hippocampus and amygdala, which may reflect synaptotoxic effects of aggregated Aβ.

**Conclusions**

The data presented shows that CSF Ng is increased specifically in AD and that high CSF Ng concentration likely reflects synaptic dysfunction associated with plaque pathology.

S16 PET RADIOLIGANDS BASED ON BISPECIFIC ANTIBODIES FOR IMAGING OF AMYLOID-BETA PATHOLOGY

Dag Sehlin1, Greta Hultqvist[1,2], Xiaotian Fang[1], Silvio Meier[1], Gunnar Antoni[3], Lars Lannfelt[1,4], Stina Syvänen[1]
[1]Department of Public Health and Caring Sciences, Uppsala University, Uppsala, Sweden; [2]Department of Pharmaceutical Biosciences, Uppsala University, Uppsala, Sweden; [3]PET Centre, Uppsala University Hospital, Uppsala, Sweden; [4]BioArctic AB, Stockholm, Sweden
*Molecular Neurodegeneration* 2019, **14(Suppl 1):**SHORT

**Correspondence:** Dag Sehlin

**Background**

Traditionally PET radioligands for brain imaging are based on small drug-like molecules. Antibodies are large molecules with low and slow brain distribution and have therefore not been used for brain PET. However, antibodies can be designed for more efficient passage across the blood-brain barrier (BBB) into the brain. Thus, the aim of the present study was to develop antibody-based radioligands, of different formats and sizes, for PET imaging of soluble amyloid-beta (Aβ) protofibrils, which are suggested to cause neurodegeneration in Alzheimer's disease (AD) [1].

**Methods**

Protofibril selective antibody mAb158 [2] was fused with either transferrin receptor (TfR) antibody 8D3 [3] or fragments of 8D3 resulting in three different bispecific antibodies. Binding to TfR via the 8D3 binding domain enabled receptor mediated transcytosis across the BBB while the mAb158-moiety bound to Aβ in the brain parenchyma. The bispecific antibodies were then labeled with iodine-124 (124I) and used for PET imaging in tg-ArcSwe and wild-type mice of different ages.

**Results**

All three bispecific antibodies bound both Aβ protofibrils and TfR, and displayed up to 80-fold better BBB transport compared to unmodified mAb158. There was a clear difference between PET images obtained in tg-ArcSwe and wild-type mice at three days post injection. The PET signal correlated closely with levels of Aβ protofibrils measured in brain homogenate [4]. Compared with [11C]PIB, antibody based PET imaging detected Aβ pathology at an earlier stage and with a larger dynamic range.

**Discussion and Conclusions**

Protofibrils were visualized in vivo with PET. To our knowledge this is the first time antibody-based ligands have been successfully used for imaging of a target inside the brain. In a longer perspective, the use of bispecific antibodies as PET ligands may enable imaging of proteins involved in diseases of the brain for which imaging agents are lacking today.

S17 TIME-RESOLVED THIOFLAVIN T FLUORESCENCE INTENSITY FLUCTUATION ANALYSIS AND TIRF MICROSCOPY FOR ULTRASENSITIVE CHARACTERIZATION OF AMYLOIDOGENIC NANO-PLAQUES

**Correspondence:** Jüri Jarvet

**Background**

Fluorescence correlation spectroscopy (FCS) is a time-resolved spectroscopic technique that can measure the concentration and size of fluorescently labeled single particles. Thioflavin T (ThT) can be used to fluorescently label amyloid structures. The FCS method benefits from the fact that many ThT molecules bind to a single amyloid aggregate. By using FCS we were able to detect with single-molecule sensitivity very low levels of small structured amyloidogenic nano-plaque particles and to monitor their propagation in time using time-resolved studies of the Amyloid β (Aβ) peptide in vitro. We could identify Aβ aggregates of different
sizes with molecular weight from 260 kDa to more than 1 × 106 kDa and revealed the hitherto unobserved kinetic turnover of intermediates during the aggregation process [1].

**Methods**

Time-resolved detection of ThT fluorescence intensity fluctuations in a sub-femtoliter sized observation volume element, allowed us to monitor ThT active nano-plaques with single-particle sensitivity. Total Internal Reflection Fluorescence (TIRF) microscopy measures fluorescence in a very thin layer (100 nm above the cover glass) of the sample.

**Results**

The results show that unlike bulk fluorescence ThT spectroscopy, time-resolved ThT fluorescence intensity fluctuation analysis allows direct detection, quantification and size determination of small structured amyloidogenic nano-plaques in solution. In addition, TIRF microscopy could be used to visualize the nanoplaque aggregates. TIRF was also used to follow the elongation of amyloid fibrils during Aβ amyloid formation in vitro.

**Discussion**

FCS and TIRF microscopy allow observation of very low concentrations of ThT-labeled amyloid particles in different solvents. The single particle observations give rise to very strong background suppression.

**Conclusions**

We can measure very low levels of the ThT active nano-plaques that are related to the actual amyloid states present in fibrils found in brain tissue.

## POSTER

## PRESENTATIONS

P1-A01 ADAM10 DEPENDENT NUCLEAR LOCALIZATION OF THE APP-BINDING PROTEIN FE65 IS ATTENUATED IN NEURONALLY DIFFERENTIATED SH-SY5Y CELLS

Niina A. Koistinen, Preeti M. Menon, Anna-Lena Ström
Department of Biochemistry and Biophysics, Stockholm University, Sweden
*Molecular Neurodegeneration* 2019, **14(Suppl 1):**POSTER

**Correspondence:** Niina A. Koistinen

**Background**

Fe65 is a brain enriched adaptor protein involved in various cellular processes [1]. Fe65 interacts with the amyloid-β precursor protein (APP) [2], and has been proposed to be involved in APP/AICD (APP intracellular domain)-dependent transcriptional activity [3]. The mechanisms behind Fe65 and APP/AICD nuclear localization are not completely understood. We have previously shown that Fe65 nuclear localization is dependent on α-secretase processing [4]. Here we further investigated the role of α-secretase processing in Fe65 nuclear localization during neuronal differentiation.

**Methods**

SH-SY5Y cells differentiated for 6 days with RA or PMA, and treated with α- or γ- secretase inhibitors (GI254023X, Batimastat or DAPT) were subject for subcellular fractionation and western blot analysis.

**Results**

The inhibition of α-secretases by Batimastat (a broad-spectrum metalloproteinase inhibitor), or γ-secretase with DAPT, decreased Fe65 nuclear levels to the same extent in both undifferentiated and differentiated SH-SY5Y cells. However, specific inhibition of ADAM10 with GI254023X was shown to have a more prominent effect on blocking Fe65 nuclear localization in undifferentiated SH-SY5Y cells.

**Discussion**

α-secretase processing seems to play a key role in promoting Fe65 nuclear localization. However, Fe65 nuclear localization appears to be regulated by different α-secretases. Hence, ADAM10 had a more prominent role in undifferentiated SH-SY5Y cells, whereas other α-secretases, in addition to ADAM10, were involved in Fe65 nuclear localization in differentiated SH-SY5Y cells. Moreover, transcriptionally active AICD has been suggested to be generated through the β-secretase pathway [5, 6]. Since α-secretases have several other substrates than APP, our results indicate that Fe65 nuclear function is not exclusively dependent on its interaction with APP/AICD.

**Conclusions**

α-secretase processing seems to have a prominent role in regulating Fe65 nuclear localization, and different α-secretases are involved in regulating Fe65 nuclear entry in non-differentiated and differentiated cells. Moreover, Fe65 nuclear function is not exclusively dependent on APP.

## P1-A03 AMYLOID β-PEPTIDES 1-40 AND 1-42 FORM OLIGOMERS WITH MIXED β-SHEETS

Maurizio Baldassarre, Cesare M. Baronio, Andreas Barth
Department of Biochemistry and Biophysics, Stockholm University, Sweden

*Molecular Neurodegeneration* 2019, **14(Suppl 1):**POSTER

**Correspondence:** Andreas Barth

**Background**

Two main amyloid-β peptides of different length (Aβ40 and Aβ42) are involved in Alzheimer's disease. Their relative abundance is decisive for the severity of the disease and mixed oligomers may contribute to the toxic species. However, little is know about the extent of mixing in oligomers. Other proteins have also been suspected to coaggregate with Aβ.

**Methods**

We used Fourier transform infrared spectroscopy in combination with 13C-labeling and spectrum calculation to study whether Aβ40 and Aβ42 co-aggregate. Mixtures of monomeric labeled Aβ40 and unlabeled Aβ42 (and vice versa) were co-incubated for ~20 min and their infrared spectra recorded.

**Results**

The spectra of the 1:1 mixtures were different from the average spectra of the labeled and unlabeled peptides, indicating that the vibrational coupling between amide oscillators was affected by mixing. The position of the main 13Camide I' band shifted to higher wavenumbers with increasing admixture of 12C-peptide due to the presence of 12C-amides in the vicinity of 13C-amides. The effect could be reproduced in spectrum calculations [1].

**Discussion**

The experimental results indicate a largely random distribution of Aβ40 and Aβ42 in the β-sheets of the mixed aggregates. Spectrum calculations are consistent with structural models in which each peptide contributes at least two adjacent β-strands (hairpin) to the β-sheets of the oligomers.

**Conclusions**

This work highlights the relevance of heterogeneous aggregates for Alzheimer's and other neurodegenerative diseases.

1. Baldassarre M, Baronio CM, Morozova-Roche LA, Barth A: Amyloid ß-peptides 1-40 and 1-42 form oligomers

## P1-A05 INTERNAL STRUCTURE OF AMYLOID BETA PEPTIDE OLIGOMERS

C.M. Baronio, A. Barth
Stockholm University, Department of Biochemistry and Biophysics, Arrhenius Laboratories, 10691 Stockholm, Sweden

*Molecular Neurodegeneration* 2019, **14(Suppl 1):**POSTER

**Correspondence:** C.M. Baronio

**Background**

The aggregation of peptides and proteins is a biological phenomenon which can cause the appearance of many neuropathological diseases as Alzheimer’s disease. Here, the protein of interest is the amyloid beta (Aβ) peptide, the aggregates of which are toxic, because they cause synaptic cell dysfunctions and/or death. The amyloid fibrils consist of parallel β-sheets, whereas the β-sheet structure of the amyloid oligomers is antiparallel. In our experimental studies [1] with infrared spectroscopy on mixtures of Aβ40 and Aβ42, one of the peptides was 13C-labelled. They showed that the absorption of the C=O bond in the backbone (amide I band) shifts to lower wavenumbers when the percentage of labeled peptide increases. Our aim is to simulate spectra, to reproduce experimental behavior and to elucidate the structure of individual peptides within Aβ oligomers.

**Methods**

A Matlab program [2] was used in order to simulate the amide I infrared spectra of antiparallel β-sheets and β-barrels, which consist of mixtures of labeled and unlabeled peptides. Normal coordinate analysis is used to predict infrared absorbance spectra of peptides. The force constant matrix, calculated according to the transition dipole coupling theory and coupling constants from density functional theory, describes the interactions between the peptide groups. Statistical distribution of labeled and unlabeled strands is examined using 3000 structures, each one with a different isotopic composition, for each experimental 12C/13C ratio.

**Results**

The results confirm the downshift of the absorbance band at increasing labeling ratios.

**Discussion**

This shift towards lower wavenumbers is smaller when individual peptides contribute a hairpin or a 3-stranded block instead of a single strand to the structure.

**Conclusions**

Ongoing calculations and comparison with the experiments will reveal which one of the three structural units is the building block of the oligomers

## P1-A06 APP Ser675 PHOSPHORYLATION AFFECTS α-SECRETASE PROCESSING RESULTING IN DECREASED SECRETION OF NEUROPROTETIVE ECTODOMAIN sAPPα

Preeti K. Menon, Niina A. Koistinen, Anna-Lena Ström
Department of Biochemistry and Biophysics, Stockholm University, Sweden
*Molecular Neurodegeneration* 2019, **14(Suppl 1):**POSTER

**Correspondence:** Preeti K. Menon

**Background**

Alzheimer's disease (AD) is a neurodegenerative disease characterized by abnormal deposition of the amyloid-β (Aβ) peptide. Aβ is produced after amyloidogenic (β-secretase) processing of the transmembrane amyloid precursor protein (APP) [1]. However, APP can also be processed by α-secretases, instead resulting in release of neuroprotective sAPPα. Growing evidence indicate that aberrant post-translational modifications of APP may play a pivotal role in AD pathogenesis by dysregulating APP processing (reviewed in [2]). APP Ser675 phosphorylation occurs in AD brains [3] and in this study we therefore investigated the effect of this modification on APP processing.

**Methods**

SK-N-AS cells expressing APPwt, APP-S675A or APP-S675E were treated with the γ-secretase inhibitor DAPT, together with an α-secretase inhibitor (GI254023X or Batimastat), and the release of sAPPα and APP CTFs levels analyzed by western blotting. In addition, the APP-FE65 interaction and the cell surface localization of APP was analyzed by a TAP-tag pull down and a biotinylation assay, respectively.

**Results**

We show that mimicking APP Ser675 phosphorylation (APP-S675E) decreases the release of sAPPα, while the level of an alternative APP-C83-CTF fragment was increased. Moreover, we found that while APP-Ser675E increased the APPFe65 interaction, the level of APP-Ser675E at the plasma membrane were unaltered.

**Discussion**

Taken together these results suggest that APP Ser675 phosphorylation alters the α-secretase processing of APP at the plasma membrane.

**Conclusions**

As α-secretase processing of APP is an essential step in decreasing the generation of Aβ, our results suggest that Ser675 phosphorylation could contribute to AD pathology.

## P1-A07 VASCULAR LOCALIZATION OF THE C-TERMINALLY TRUNCATED AMYLOID β-PEPTIDE SPECIES Aβ34

Tunahan Kirabali[1], Filip Liebsch[2], Roger M. Nitsch[1], Gerhard Multhaup[2], Luka Kulic[1]
[1]Institute for Regenerative Medicine, University of Zurich, Zurich, Switzerland; [2]Department of Pharmacology & Therapeutics, McGill University, Montreal, Canada
*Molecular Neurodegeneration* 2019, **14(Suppl 1):**POSTER

**Correspondence:** Tunahan Kirabali

**Background**

The C-terminally truncated amyloid β-peptide (Aβ) isoform, Aβ34, has been identified as an important intermediate product of enzymatic Aβ degradation[1]. It can be detected in cerebrospinal fluid, plasma and cell culture supernatants and has therefore the potential to be used as a promising novel biomarker of amyloid clearance in Alzheimer's Disease (AD)[2, 3] . In this study, we aimed to identify the distribution of Aβ34 in human brain and its possible role in Aβ clearance pathways.

**Methods**

Human post-mortem cortex and hippocampus samples categorized according to Braak stages, CERAD scores, ApoE genotype and disease status (AD or non-AD) were analyzed by immunohistochemistry using a highly specific novel monoclonal antibody directed against Aβ34. Human primary pericyte cultures were used to study Aβ34 metabolism in vitro. In addition, microvessels isolated from human cortex were assessed for the presence of Aβ34 along perivascular drainage pathways.

**Results**

Aβ34 immunoreactivity was observed exclusively in small capillaries and post-capillary venules during early Braak stages, in which it co-localized with the pericyte marker PDGFR-β. In late Braak stages, however, a significant loss of Aβ34 immunoreactivity and redistribution to larger congophilic amyloid angiopathy(CAA)-laden vessels was observed. Non-demented controls showed significantly higher Aβ34 immunoreactivity compared to AD patients. Aβ34 immunoreactivity was also detected in isolated human microvessels and in cultured human primary pericytes.

**Conclusion**

Early detection of Aβ34 in the vasculature and its association with pericytes point to the existence of a yet unknown novel Aβ degradation pathway along vascular clearance routes. A failure of this degradation pathway might contribute to increased Aβ accumulation and amyloid deposition in early AD pathogenesis.

## P1-A08 AN AMYLOID PRECURSOR C-TERMINAL FRAGMENT OF APPROXIMATELY 23 KILODALTON IS HIGHLY ABUNDANT IN HUMAN BUT NOT IN MOUSE AND RAT BRAIN

Susanne Frykman[1], Hazal Haytural[1], Jolanta L. Lundgren[1], Tomàs Jordà Siquier[2], Mohammed Seed Ahmed[1,3,4], Bengt Winblad[1], Dag Årsland[1,5], Caroline Graff[1], Gaël Barthet[2], Lars O. Tjernberg[1]
1Department of Neurobiology, Care Science and Society, Karolinska Institutet, Sweden, 2Interdisciplinary Institute of Neuroscience, Université de Bordeaux, France, 3Department of Learning, Informatics, Management and Ethics, Karolinska Institutet, Sweden, 4Department of Physiology, University of Khartoum, Sudan, 5Department of Old Age Psychiatry, King´s College London, UK
*Molecular Neurodegeneration* 2019, **14(Suppl 1):**POSTER

**Correspondence:** Susanne Frykman

**Background**

The amyloid β-peptide plays an important role in Alzheimer Disease (AD) and is derived from the amyloid precursor protein (APP) by sequential processing by β- and γ-secretase. Recently, a novel synaptotoxic APP fragment, Aα-η was identified that is derived from an alternative C-terminal fragment APPCTF-η [1]. However, the levels of the different CTFs in human brain has, to our knowledge, not been thoroughly investigated.

**Methods**

We used brain material from AD and control humans and human embryo; rat; adult and embryonic and transgenic mouse; guinea pig and macaque and determined the presence of APP c-terminal fragments using a number of c-terminal antibodies, western blotting, mass spectrometry and immunoprecipitation.

**Results**

We found that a 23 kDa band - possibly corresponding to APP-CTF-η - was present at much higher levels in human than in rat brain. The identity of the 23 kDa band was confirmed by siRNA silencing and mass spectrometry. In adult brain, the 23 kDa band was abundant in guinea pig and macaque but neither in rat, wildtype nor APP transgenic mice. Opposite to adult, the CTF pattern was similar in human and mouse embryonic brain, with low levels of all CTFs and no detectable levels of the 23 kDa CTF. No significant differences were detected between AD and control brain.

**Discussion**

The relative abundance of the 23 kDa band in guinea pig and macaque brain can possibly explain why these species develop amyloid plaques whereas mice and rats don't. The absence of the 23 kDa band in human APP transgenic mice indicates that it is the environment in the human brain, rather than the human APP gene, that influences the differential processing.

**Conclusions**

We detect important differences in APP processing between mice and rats and humans which should be taken into consideration when translating animal model studies to clinical trials.

## P1-A09 NEURONAL Aβ42 IS PRESENT AT THE PRESYNAPTIC SIDE OF THE SYNAPSE

Yang Yu[1], Daniel C. Jans[2], Bengt Winblad[1], Lars Tjernberg[1], Sophia Schedin-Weiss[1]
[1]Karolinska Institutet, Department of NVS, Center for Alzheimer Research, Karolinska Institutet, Division of Neurogeriatrics, 141 57 Huddinge, Sweden; [2]Science for Life Laboratory, Department of Applied Physics, Royal Institute of Technology, 171 65, Stockholm, Sweden
*Molecular Neurodegeneration* 2019, **14(Suppl 1):**POSTER

**Correspondence:** Yang Yu

**Background**

The amyloid β-peptide containing 42 amino acids (Aβ42) is neurotoxic and believed to be acausative agent in Alzheimer disease (AD), but the molecular details behind its role in the initiating eventsleading to clinical AD are elusive. It has previously been shown that the Aβ generating enzyme γ-secretase isenriched at both sides of the synapse [1]. However, since γ-secretase has a multitude of substrates besides theAβ precursor protein (APP), the question of where Aβ is produced is still open.

**Methods**

Mouse primary hippocampal neurons cultured for 21 days in vitro were imaged with super-resolution microscopy (STED, STORM and three-dimensional STED). To visualize the location of Aβ42, we used a C-terminal specific Aβ42 antibody (anti-AβC42), the presynaptic marker synaptophysin or postsynaptic marker PSD95, combined with a confocal channel for actin staining.

**Results**

We focused on the neurites, and found that STED made it possible to distinguish between different Aβ42 structures. A large fraction of Aβ42 was present in small vesicles found in the presynapse, opposite to PSD95 clusters in mushroom, thin and stubby spines, as well as in immature synapses. Interestingly, the majority of these vesicles were not stained by synaptophysin, suggesting that Aβ42 is present in a different type of vesicle at the presynapse. We also quantified the relative presence of Aβ42 in the pre- and postsynaptic sides of the synapse, showing that 97% of the presynapses, but only 5.2% of the dendritic spine heads, contained Aβ42.

**Discussion**

Aβ vesicles are highly abundant at the presynapse in vesicles that lack synaptophysin. Other studies have in line with our results concluded that Aβ42 is secreted at the synapse, and that the major pool is independent on synaptic activity [2].

**Conclusions**

Aβ42 is present at the presynaptic side of the synapse in vesicles lacking synaptophysin.

## P1-A10 THIOREDOXIN-80 PROTECTS AGAINST AD-LIKE PATHOLOGY THROUGH AUTOPHAGY-LYSOSOMAL PATHWAY REGULATION

Gorka Gerenu[1,2], Torbjörn Persson†[1], Julen Goikolea[1], Javier Calvo-Garrido[1], Philipp Pottmeier[1], Helen Poska[1], Jenny Presto[1], Angel Cedazo-Minguez[1]
[1]Karolinska Institutet; Department of Neurobiology, Care Sciences and Society; Center for Alzheimer Research, Division for Neurogeriatrics, 141 57 Huddinge, Sweden; [2]Biodonostia Health Research Institute, Neuroscience Area, Donostia-San Sebastián, Gipuzkoa, 20014, Spain
*Molecular Neurodegeneration* 2019, **14(Suppl 1):**POSTER

**Correspondence:** Gorka Gerenu

**Background**

Aggregation and accumulation of amyloid-beta (Aβ) is believed to be of great importance in the pathogenesis of Alzheimer disease (AD). We recently reported that Thioredoxin-80 (Trx80), a truncated form of Thioredoxin-1, prevents the toxic effects of Aβ and inhibits its aggregation in vitro. Trx80 is present in human brain and cerebrospinal fluid, with dramatically reduced levels in AD patients. In this study, we investigated the effect of Trx80 in in vivo and in vitro models of Aβ pathology.

**Methods**

We developed transgenic models of Drosophila Melanogaster that overexpresses human Trx80, human Aβ42 or both; exclusively in the central nervous system. Longevity and Locomotor tests were assessed and the results were confirmaed by further molecular studies. SHSY-5Y neuroblastoma cell line was used to confirm and enhance the power of our results.

**Results**

We found that Trx80 prevents Aβ accumulation in the brain and rescues the reduction in lifespan and locomotor impairment seen in Aβ42 expressing flies. We showed that Trx80 induces autophagolysosome formation and reverse the inhibition of Atg4B-Atg8a/b pathway caused byAβ42. These effects were confirmed in human neuroblastoma cells with an effect of Trx80 on reducing Aβ42 levels and activating the autophagic machinery.

**Conclusions**

These results give insight about the function of Trx80 in vivo and suggest that Trx80 has an effect on the formation of autophagolysosomes, which results in enhanced Aβ42 degradation. In addition, it adds support to the view that Trx80 can be part of a novel therapeutic treatment for AD.

P1-A11 A ROLE OF ALZHEIMER'S DISEASE ASSOCIATED γ-SECRETASE ACTIVITY IN LIPID METABOLISM AND LIPID DROPLET FORMATION

Esteban Gutierrez[1], Dieter Lütjohann[2], Lars Kürschner[3], Christoph Thiele[3], Jochen Walter[1]
[1]Department of Neurology, University Hospital Bonn, Germany; [2]Institute for Clinical Chemistry and Clinical Pharmacology; University Hospital Bonn, Bonn, Germany; [3]Life and Medical Sciences (LIMES),, Membrane Biology & Biochemistry. Unit, University of Bonn, Bonn, Germany
*Molecular Neurodegeneration* 2019, **14(Suppl 1):**POSTER

**Correspondence:** Jochen Walter

**Background**

Mutations in the presenilins (PS) or the amyloid precursor protein (APP) are a major cause of familial Alzheimer's disease (AD). PS proteins are the catalytically active components of the γ-secretase complex that cleavages C-terminal fragments (CTF) of APP to generate the amyloid β peptide (Aβ). While the involvement of PS and APP in AD is well recognized in the production of Aβ, additional mechanisms potentially linking these proteins to AD pathogenesis are not comprehensively understood. It has been shown that PS proteins are also involved in cellular cholesterol metabolism (1–3). This study aimed to further elucidate the role of PS proteins and the APP CTF on cellular metabolismof sterols, triglycerides and lipid droplets (LD).

**Materials and Methods**

γ-Secretase activity was modulated pharmacologically or genetically in human astroglioma H4 or mouse embryonic fibroblasts. LD content was analyzed by fluorescence microscopy using the dye LD540. Sterol levels and esterification ratios where analyzed by gas-liquid chromatography-mass spectrometry (GLCMS) and the Amplex red cholesterol assay. The interaction between APP CTF and cholesterol was studied by fluorescence microscopy on cell models overexpressing an APP C99-GFP fusion protein, combined with cholesterol staining using Filipin.

**Results**

Genetic deletion of PS or pharmacological inhibition of γ-secretase activity lead to significantly increased amounts of lipid droplets and triglycerides, together with significantly higher levels of the cholesterol precursors lathosterol and desmosterol, and significantly lower sterol esterification ratios. Moreover, following pharmacological inhibition of γ-secretase, overexpressed APP C99-GFP accumulated in cholesterol positive intracellular membrane structures. Increased expression and accumulation of C99-GFP was shown to correlate with an increased number of cellular LDs. These effects were associated with increased activity of the liver X receptor and upregulation of target genes that regulate cellular lipid metabolism.

**Discussion and Conclusions**

Our findings support an important role of PS and γ-secretase activity in cellular lipid metabolism. We identified alterations relevant to cellular sterol and lipid homeostasis upon loss of γ-secretase activity. Furthermore, the observed association of C99-GFP with cholesterol in cell-based assays, together with the correlation between C99-GFP accumulation and increased LD formation, indicates that this γ-secretase substrate represents a potential link between PS activity and lipid metabolism.

P1-A13 INHIBITION OF AMYLOID β PEPTIDE 1-40 (Aβ40) AGGREGATION BY ANTI-PROTOFIBRIL ANTIBODY STUDIED BY FLUORESCENCE CORRELATION SPECTROSCOPY AND DUAL-COLOR FLUORESCENCE CROSS-CORRELATION SPECTROSCOPY

**Correspondence:** Ann Tiiman

**Background**

Alzheimer's disease (AD) is a progressive neurodegenerative disorder that is characterized by the self-assembly of amyloid β (Aβ) peptides and their deposition into plaques. Conformation-sensitive antibodies are able to bind to early Aβ aggregates and inhibit amyloid formation, but the detailed kinetic mechanisms underlying this complex process are not fully understood. We have recently shown that timeresolved methods with single-molecule sensitivity can characterize the turnover of intermediates during Aβ aggregation [1], and use here this novel approach to analyze the effect of the 5C9.A2 antibody on the time course of Aβ aggregation.

**Methods**

Fluorescence Correlation Spectroscopy (FCS), dual-color Fluorescence Cross-Correlation spectroscopy (FCCS) and Confocal Laser Scanning Microscopy (CLSM) were used to characterize in vitro the effects of the 5C9.A2 antibody on the time course of Aβ 1-40 (Aβ40) aggregation.

Materials include: recombinant unlabeled Aβ40peptide, fluorescently tagged Aβ40-Alexa488, 5C9.A2 antibody, fluorescently tagged 5C9.A2-DyLight633 antibody and Thioflavin T.

**Results**

Our study shows that the 5C9.A2 antibody inhibits Aβ aggregation in solution in a concentration dependent manner and can totally abolish this process at higher concentrations, for 10 μM Aβ40 the required antibody concentration was 120 ± 60 ng/μl. The 5C9.A2 antibody does not bind to the functionally active Aβ40 monomers, but selectively interacts with early intermediates formed during the aggregation process.

**Discussion**

It is generally accepted that oligomers are the most toxic species in the Aβ aggregation pathway and their early detection and clearance are crucial for successful AD therapy and prevention [2]. The newly developed 5C9.A2 antibody binds to early intermediates and can be used to detect amyloid formation in tissue sections and reduce toxicity in vivo.

**Conclusions**

Inhibition of Aβ aggregation by the 5C9.A2 antibody occurs through its interactions with early intermediates in the Aβ aggregation process, not with Aβ monomers.

## P1-A14 γ-SECRETASE INHIBITION INDUCES LIPID DROPLET ACCUMULATION VIA APP-CTF ACCUMULATION

Naoto Oikawa[1], Ulrike C. Müller[2], Jochen Walter[1]
[1]Department of Neurology, University of Bonn, Bonn, Germany; [2]Institute for Pharmacy and Molecular Biotechnology, University of Heidelberg, Heidelberg, Germany
*Molecular Neurodegeneration* 2019, **14(Suppl 1):**POSTER

**Correspondence:** Naoto Oikawa

**Background**

Abnormal cholesterol metabolism is suspected as one of the factors contributing to Alzheimer disease (AD) pathogenesis. We and others have previously shown that γ-secretase dysfunction, which appears to be a main consequence caused by clinical presenilin mutations relevant to familial AD, increases cholesterol level in non-neuronal cells [1, 2]. Additionally, we proposed that increase of one of the γ-secretase substrates, amyloid precursor protein C-terminal fragments (APP-CTFs), is a possible mediator of the cholesterol increase [2]. In this study, we examined the involvement of APP-CTFs in the metabolism of cholesterol and lipid droplets [3] in neuronally differentiated SH-SY5Y (nSY5Y) cells and in mouse embryonic fibroblasts lacking APP expression (MEFs-APPKO).

**Methods**

nSY5Y cells differentiated by retinoic acid or MEFs-wild type (MEFs-WT) or MEFs-APPKO were treated with a γ-secretase inhibitor, N-[N-(3,5-Difluorophenacetyl)-L-alanyl]-Sphenylglycine t-butyl ester (DAPT). To suppress APP-CTF accumulation in nSY5Y cells upon DAPT treatment, cells were co-treated with inhibitors of α-secretase or β-secretase. Levels of lipid droplets and cholesterol were measured by oil red-O staining and enzymatic assay, respectively.

**Results**

γ-Secretase inhibition in nSY5Y cells by DAPT significantly increased levels of lipid droplet and cholesterol and affected the expression profile of the proteins involved in cholesterol metabolism, such as ABCA1, NPC1, sterol regulatory element-binding protein 2, and LDLR. Suppression of the DAPT-induced APP-CTFs accumulation completely rescued lipid droplet accumulation; however, cholesterol accumulation and abnormal expression profile of the proteins were not rescued by suppression of the APP-CTFs accumulation. Additionally, γ-secretase inhibition induced lipid droplet accumulation only in MEFs-WT but not in MEFs-APPKO in contrast to cholesterol accumulation, which was detected in both of them upon DAPT treatment.

**Discussion/Conclusions**

These results indicate that γ-secretase inhibition has complex effects on cellular lipid metabolism in neuronal and non-neuronal cells, partly involving accumulated APP-CTFs.

## P1-A15 ULTRASTRUCTURAL ANALYSIS OF VESICLE KINETICS IN APPSwe/Ind MICE

Luca Biasetti[1], Stephanie A. Rey[1], Catherine A. Smith[1], Milena W. Fowler[1], Sarah L. King[1], Mariana Vargas-Caballero[2], Louise C. Serpell[1], Kevin Staras[1]
[1]Dementia Research Group, Sussex Neuroscience, School of Life Sciences, University of Sussex, Falmer, UK; [2]Institute for Life Sciences, University of Southampton, Southampton, UK
*Molecular Neurodegeneration* 2019, **14(Suppl 1):**POSTER

**Correspondence:** Luca Biasetti

**Background**

Alzheimer's disease (AD) is characterized by neurological pathology that causes progressive deterioration of the brain and its functions. The majority of AD cases are sporadic, although there are some hereditary forms which are early onset. It is known that normal synaptic function and information transmission are impaired in the disease; however, the specific cellular targets and underlying mechanisms are yet to be fully elucidated. An important candidate substrate is the population of neurotransmitter-containing vesicles located in the presynaptic terminals, known to be critical determinants of synaptic efficacy.

**Methods**

Here we investigate the properties of pre-synaptic vesicles that are recruited by synaptic activity in CA3-CA1 hippocampal synapses from acute brain slices obtained from 3-month-old transgenic APPswe/Ind mice [1]. Specifically, we couple electrophysiological stimulation with FM1-43FX dye labelling and then photoconvert fluorescently-labelled vesicles for subsequent ultrastructural analysis [2].

**Results**

Our results show a robust and significant increase in the total number of vesicles recruited by a saturating loading simulus hippocampal CA1 synapses versus matched controls. Furthermore, we observe differences in the physical organization of pools; in particular, functional vesicles located at sites closer to the active zone in the AD model versus controls.

**Discussion**

The significantly higher percentage of functional vesicles in APPswe/Ind mice compared to controls suggests a change in the vesicle exocytosis and retrieval processes; one hypothesis is that the efficient mechanisms of recycling that normally permit a substantial re-use of retrieved vesicles in a maintained stimulus train are defective in AD mice. The increase in the total vesicle recycling pool size would thus represent a compensatory mechanism, similar to pool rescaling observed in forms of homeostatic silencing.

**Conclusions**

Our findings suggest that changes and defects in vesicle turnover and clearance in the presynaptic terminal may play an important role in the network dysfunction seen in Alzheimer's disease.

## P1-A16 THE NEURONAL S100B PROTEIN IS A CALCIUM-TUNED SUPPRESSOR OF AMYLOID-β AGGREGATION

Joana S. Cristóvão[1], Vanessa K. Morris[2,3], Isabel Cardoso[4,5], Sónia S. Leal[1], Javier Martinez[1], Hugo M. Botelho[1], Christoph Göbl[2,3], Rodrigo David[1], Katrin Kierdorf[7,8], Mobina Alemi[4,5,6], Tobias Madl[2,3,9], Günter Fritz[7], Bernd Reif[2,3], Cláudio M. Gomes[1]
[1]Biosystems and Integrative Sciences Institute, Faculdade de Ciências, Universidade de Lisboa, 1749-016 Lisboa, Portugal and Departamento de Química e Bioquímica, Universidade de Lisboa, Portugal; [2]Center for Integrated Protein Science Munich at Department of Chemistry, Technische Universität München, Germany; [3]Helmholtz Zentrum München, Deutsches Forschungszentrum für Gesundheit und Umwelt, Germany; [4]IBMC - Instituto de Biologia Molecular e Celular, Universidade do Porto, Portugal; [5]i3S - Instituto de Investigação e Inovação em Saúde, Universidade do Porto, Portugal; [6]Faculdade de Medicina, Universidade do Porto, Portugal; [7]Department of Neuropathology, University of Freiburg, Germany; [8]MRC Centre for Molecular Bacteriology and Infection and Department of Life Sciences, Imperial College London, UK; [9]Institute of Molecular Biology & Biochemistry, Center of Molecular Medicine, Medical University of Graz, Austria
*Molecular Neurodegeneration* 2019, **14(Suppl 1):**POSTER

**Correspondence:** Cláudio M. Gomes

**Background**

Neuroinflammation and deposits of insoluble amyloid β1-42 (Aβ42) in the synaptic cleft are features in Alzheimer's disease (AD) and strong candidates for the initiation of the neurodegeneration process. S100B is an abundant pro-inflammatory calcium-binding protein chronically up-regulated in AD and associated with senile plaques.

**Methods**

Our approach combines complementary molecular, cellular and biophysical methods.

**Results**

We demonstrate a novel role for the neuronal S100B protein as a chaperone-like molecule that interacts with Aβ42, inhibiting its aggregation and decreasing Aβ42 toxicity. This involves a dynamic interaction of Aβ42 with an interfacial cleft within the S100B dimer which is favored by calcium-binding to S100B. Further, we establish that the aggregation-suppressing activity is influenced by calcium-binding to S100B, as different microscopic steps in Aβ42 aggregation (primary and secondary nucleation) are differentially affected, depending on whether calcium is bound to S100B or not. Our results also show that S100B protects cells from Aβ42-mediated toxicity, rescuing cell viability and decreasing apoptosis induced by Aβ42 in cell cultures

**Discussion**

The fact that S100B is one of the most abundant proteins in the brain (0.5% of total protein) which is further augmented in the ageing brain, upon traumatic brain injury and in AD itself, suggests that our findings of this novel regulatory role of S100B over Aβ42 aggregation may be a very relevant process in the context of AD physiopathology.

**Conclusions**

Our work support previous evidence for roles of S100 proteins in neurodegeneration, and establish a novel view for a relationship between inflammation and protein deposition, by implicating the proinflammatory S100B protein in a novel chaperone-like function as regulator of Aβ42 aggregation and toxicity. Also, our findings suggest that molecular targeting of S100B could be translated into the development of novel approaches to ameliorate AD neurodegeneration.

## P1-A17 SOMATOSTATIN RECEPTOR SUBTYPES 1 AND 4 REDUNDANTLY REGULATE THE MAJOR Aβ-DEGRADING ENZYME NEPRILYSIN

Per Nilsson[1], Karin Sörgjerd[2], Naomasa Kakiya[2], Hiroki Sasaguri[1]†, Zhulin Zhou[1], Hayato Isshiki[2], Misaki Sekiguchi[2], Aline Petrish3, Stefan Schulz[3], Takashi Saito[2], Bengt Winblad[1], Takaomi C. Saido[2]
[1]Department of Neurobiology, Care Sciences and Society, Division for Neurogeriatrics, Karolinska Institutet, Huddinge, Sweden; [2]Laboratory for Proteolytic Neuroscience, RIKEN Brain Science Institute, Wako, Saitama, Japan; [3]Institute of Pharmacology and Toxicology, Jena University Hospital, Friedrich-Schiller University, 07747, Jena, Germany
*Molecular Neurodegeneration* 2019, **14(Suppl 1):**POSTER

**Correspondence:** Per Nilsson

**Background**

Abnormally altered Aβ metabolism leads to development of Alzheimer's disease (AD). Especially in sporadic AD, which lacks robust genetic components, a decrease in the degradation of Aβ may contribute to the increased Aβ levels. In previous work, we identified neprilysin as the major Aβ-degrading enzyme. We subsequently found that neprilysin activity is regulated in the neurons by somatostatin, a neuropeptide decreased with aging and in AD possibly due to death of somatostatinergic interneurons. Here we have identified the somatostatin receptor subtypes responsible for the regulation of neprilysin. Here we will present the identity of the two SSTR subtypes involved in the regulation of neprilysin.

**Methods**

The importance of the SSTR subtypes in neprilysin regulation was studied by doubly knocking out (KO) in mouse SSTR subtypes expressed in cortex and hippocampus in a combinatorial manner. SSTR1 x 4 double KO mice was then crossed with a novel App-knockin mouse model of AD exhibiting robust Aβ pathology.

**Results**

A simultaneous knockout of SSTR1 and SSTR4 led to specific down-regulation of neprilysin in dentate gyrus. In addition, genetic deficiency of these two receptors led to increased Aβ levels and impaired memory in the mice, indicating an important role of these receptors in cognitive function. SSTR1x4 double KO mice crossed with App-knockin mice exhibited increased Aβ pathology in hippocampus.

**Discussion**

These findings indicate presence of functional redundancy among the receptor subtypes in the regulation of neprilysin. SSTR1 and 4 redundantly regulate neprilysin and subsequently Aβ pathology.

**Conclusions**

SSTR1 and 4 regulate neprilysin and could be targets to lower Aβ levels in AD.

## P1-A18 BIOPHYSICAL STUDIES OF METAL ION INTERACTIONS WITH THE AMYLOID-β PEPTIDE

Cecilia Wallin[1], Axel Abelein[2], Sebastian Wärmländer[1], Astrid Gräslund[1]
[1]Department of Biochemistry and Biophysics, Stockholm University, Stockholm, Sweden; -Department of Neurobiology, Care Sciences and Society, Center for Alzheimer Research, Division of Neurogeriatrics, Karolinska Institutet, Sweden
*Molecular Neurodegeneration* 2019, **14(Suppl 1):**POSTER

**Correspondence:** Cecilia Wallin

**Background**

Senile plaques consist mainly of insoluble amyloid-β (Aβ) peptides aggregated into amyloid fibrils, and various small molecules such as metal ions [1]. The self-assembly of Aβ peptides is suggested as a central process in Alzheimer's disease (AD) [2]. Here we study in detail how metal ions affect the Aβ aggregation properties and self-assembly.

**Methods**

In these studies we used a combination of spectroscopic methods such as nuclear magnetic resonance (NMR), circular dichroism (CD) and fluorescence spectroscopy, as well as aggregation kinetics and atomic force microscopy (AFM) imaging.

**Results**

Ongoing studies show changed Aβ properties and aggregation patterns upon metal binding. The monomeric Aβ peptide binds metal ions such as Zn(II), Mn(II), Pb(IV), Hg(II), and Ag(I) ions specifically at the N-terminus in the milli-to micromolar range, both located in a membrane mimicking environment and as a free monomer in buffer solutions. The metal ion interaction is transient and pulse field gradient translational diffusion experiments reveal a more compact structure in the presence of a metal ion. The compact structure suggests an induced fold of the N-terminal part of the Aβ peptide around the metal ion to coordinate the ligands. How the affinity for the Aβ/metal complex changes during the aggregation pathways are still unclear.

Preliminary results show modulation of the aggregation process in a metal ion concentration-dependent manner.

**Discussion**

From the physiological perspective, metal binding to the Aβ peptide might influence 1) the chemical properties of the Aβ peptide and hence its aggregation propensities and the expression of AβPP, 2) oxidative stress from induction of reactive oxygen species and 3) mis-localized essential metal ions and both gain of toxicity and loss of function. The similarities and differences in how different metal ion species affect the amyloid aggregation properties in relation to biological relevance are subject to further investigation.

**Conclusions**

Coordinating metal ligands in the peptide are typically located in the N-terminus. A specific metal binding mode affects the structure of the Aβ peptide and hence its properties.

## P1-A19 PEPTIDE CONSTRUCTS WITH SIGNAL SEQUENCE MOTIFS EFFICIENTLY MODIFIES THE AMYLOID-β PEPTIDE AGGREGATION KINETICS IN VITRO

Sylwia Król[1], Cecilia Wallin[1], Jüri Jarvet[1], Sebastian Wärmländer[1], Anja Henning-Knechtel[2], Mazin Magzoub[2], Astrid Gräslund[1]

[1] Department of Biochemistry and Biophysics, Stockholm University, Stockholm, Sweden; [2] Biology Program, Division of Science, New York University Abu Dhabi, Abu Dhabi, UAE

*Molecular Neurodegeneration* 2019, **14(Suppl 1):**POSTER

**Correspondence:** Sylwia Król

**Background**

Alzheimer's disease (AD) is characterized by abnormal deposition of neurotoxic amyloid-β (Aβ) peptides. Up to date a variety of potential inhibitors of aggregation of Aβ have been studied, but in most cases their mechanism of action is still not fully understood. In this study we present results on how the Aβ peptide self-assembly is modified by two novel peptide constructs; NCAM-Prion and NCAM-Aβ. The peptides exhibit properties of cell penetrating peptides (CPP) and were originally designed to reduce prion propagation [1]. They consist of two segments, one hydrophobic signal sequence from the Neuronal cell adhesion molecule-1 (NCAM1-19), which is followed by a prion protein (1-6) sequence (KKRPKP), or the Aβ (16-20) sequence plus one extra K (KKLVFF).

**Methods**

Effects of NCAM-Prion and NCAM-Aβ on the Aβ fibril formation were studied by Thioflavin T (ThT) fluorescence kinetic experiments, while potential secondary structure changes and conformational rearrangements were investigated by circular dichroism (CD) and Nuclear magnetic resonance (NMR). Atomic force microscopy (AFM) was used for imaging of the fibril formation upon addition of NCAMPrion and NCAM-Aβ peptides.

**Results and discussion**

Our experiments revealed that NCAM-Prion and NCAM-Aβ affect the Aβ amyloid aggregation process in a concentration-dependent manner. 1D and 2D NMR experiments indicated an interaction between the Aβ peptide and the NCAM-Prion peptide. The CD spectra confirmed concentration and time dependent structural changes of secondary structures of both Aβ peptide in the presence of the NCAMPrion or NCAM-Aβ peptides and of the NCAM-Prion and NCAM-Aβ peptides alone. Upon incorporation of an additional sample purification step, size exclusion chromatography (SEC), it was also revealed that the effect from NCAM peptides on Aβ fibril formation differed depending on the starting material of Aβ.

**Conclusion**

The outcome of the Aβ amyloid formation process is dependent on the presence of the two peptide constructs. Such peptides may be considered as starting points for developing therapies against AD.

## P1-A20 MERCURY AND ALZHEIMER'S DISEASE: Hg(II) IONS DISPLAY SPECIFIC BINDING TO THE AMYLOID-BETA (Aβ) PEPTIDE

Sebastian Wärmländer, Cecilia Wallin, Jüri Jarvet, Astrid Gräslund

Department of Biochemistry and Biophysics, Arrhenius Laboratories, Stockholm University, 106 91, Stockholm, Sweden

*Molecular Neurodegeneration* 2019, **14(Suppl 1):**POSTER

**Correspondence:** Sebastian Wärmländer

**Background**

Growing evidence links Alzheimer's disease (AD) to metal exposure. The role of metals in AD pathogenesis is however unresolved. AD pathology involves deposition inside the brain of amyloid plaques, consisting mainly of aggregated amyloid-β (Aβ) peptides. The plaques in AD patients contain numerous metals, most prominently Ca, Cu, Fe, and Zn, but also smaller amounts of other transition metals and heavy metals. Epidemiologic studies have implicated mercury as a risk factor in AD, but no links between Hg and Aβ aggregation and plaque formation have been observed at a molecular level.

**Methods**

We study the interaction between Aβ(1-40) peptides and Hg(II) ions in vitro, using biophysical methods of spectroscopy and imaging such as AFM, NMR, CD, and fluorescence spectroscopy.

**Results**

We show that Hg(II) ions display relatively strong and specific binding to the N-terminal part of the Aβ peptide. When bound, the Hg(II) ions change the Aβ aggregation properties in a concentration-dependent manner.

**Discussion**

This is the first time a molecular mechanism has been observed that connects Hg ions to the Aβ aggregation that underlies Alzheimer's disease.

**Conclusions**

Our in vitro results showing specific interactions between Hg(II) ions and the Aβ(1-40) peptide provides evidence at a molecular level for the previously suggested connection between AD and mercury exposure.

3. Schrötter, A., Mastalski, T., Nensa, F. M., Neumann, M., Loosse, C., Pfeiffer, K., Magraoui, F. E., Platta, H. W., Erdmann, R., Theiss, C., Uszkoreit, J., Eisenacher, M., Meyer, H. E., Marcus, K., and Müller, T. (2013) FE65 regulates and interacts with the Bloom syndrome protein in dynamic nuclear spheres - potential relevance to Alzheimer's disease. J Cell Sci 126, 2480-2492

## P1-A21 AMYLOID PRECURSOR PROTEIN SIGNALS TO THE NUCLEUS GENERATING p53-CONTAINING SPHERICAL COMPLEX

Hassan Bukhari[1], Katharina Kolbe[1], David Marks[1], Sophia Meermeyer[1], Verian Bader[2], Anne-Mette Bjerg Petersen[1], Katharina Heß[3], Werner Paulus[3], Michael T Heneka[4], Konstanze Winklhofer[2], Thomas Günther-Pomorski[5], Thorsten Müller[1]

[1]Cell Signaling, Molecular Biochemistry, Ruhr- University Bochum, Germany; [2]Molecular Cell Biology, Institute of Biochemistry and Pathobiochemistry, Ruhr University Bochum, 44801 Bochum, Germany; [3]Institute of Neuropathology, University Hospital Münster, Münster, Germany; [4]German Center for Neurodegenerative Diseases (DZNE), Sigmund Freud Str. 27, 53127, Bonn, Germany; [5]Department of Molecular Biochemistry, Faculty of Chemistry and Biochemistry, Ruhr University Bochum, Universitätsstrasse 150, 44780, Bochum, Germany

*Molecular Neurodegeneration* 2019, **14(Suppl 1):**POSTER

**Correspondence:** Thorsten Müller

**Background**

Amyloid precursor protein has been suggested to signal to the nucleus via its cytosolic domain (1). Cleavage of APP as well as its phosphorylation are thought to modulate this pathway (2) – mechanisms that are known to be involved in the pathology of Alzheimer's disease (AD).

**Methods**

By the use of confocal imaging, fluorescence-lifetime imaging, photoactivated localization microscopy, and electron microscopy the nuclear APP-dependent complex has been studied in cell culture and human brain hippocampal slices. Confocal live cell imaging has been used to study nuclear complex dynamics.

**Results**

APP signaling causes the generation of a nuclear complex consisting of FE65, TIP60, BLM (3), p53 and other proteins. The APP c-terminal domain is not a prerequisite for genesis of the complex in the nucleus, but regulates its formation via its interaction to the FE65 adapter protein, which depends on APP cleavage and/or phosphorylation. Upon genesis, the spherical complex, which does not contain a membrane coating, is highly dynamic and single complexes fuse to larger structures. Initial data point to a high toxicity of the complex, especially in neuronal cells.

**Discussion**

Derived from the nuclear complex composition, including DNA helicases like BLM, the histone acetyl transferase TIP60, and the tumor suppressor p53, this APP signaling pathway might play a role in DNA replication or repair.

**Conclusions**

Early stages of Alzheimer's disease are characterized by neurons that are positive for cell cycle re-entry markers indicating that adult neurons got a signal to induce mitosis. Oxidative damage including accumulation of mutations in mitochondrial DNA are currently discussed to occur in early stage AD. These immunostaining derived observations might be the consequence of APP signaling to the cellular nucleus. Thus, the APP-derived generation of nuclear aggregates might be a relevant pathway in AD.

## P1-A22 AMYLOID PRECURSOR PROTEIN FAMILY MEMBERS FUNCTION AS SYNAPTIC CELL ADHESION MOLECULES

Simone Eggert[1], Sandra Schilling[1], Susann Ludewig[2], Alexander August[1], Paul Strecker[1], Jonathan Stephan[3], Martin Korte[2], Ulrike Müller[4] and Stefan Kins[1]

[1]Department of Human Biology and Human Genetics, University of Kaiserslautern, 67663 Kaiserslautern, Germany; [2]Department of Cellular Neurobiology, TU Braunschweig, Zoological Institute, 38106 Braunschweig, Germany; [3]Department of Animal Physiology, University of Kaiserslautern, 67663 Kaiserslautern, Germany; [4]Institute for Pharmacy and Molecular Biotechnology, University of Heidelberg, 69120 Heidelberg, Germany

*Molecular Neurodegeneration* 2019, **14(Suppl 1):**POSTER

**Correspondence:** Stefan Kins

**Background**

The amyloid precursor protein (APP) plays a pivotal role in synapse formation and synaptic plasticity. In part, these functions are mediated by the secreted ectodomain, sAPPalpha. However, accumulating evidence also suggests an essential function of membrane tethered full-length APP and its homologues APLP1 and APLP2 at the synapse.

**Methods**

We used different biochemical studies, including ITC, in vitro bead aggregation assayand Co-IPs to analyze trans-dimerization properties of APP/APLPs. The mixed co-culture assay was used to analyze APP/APLPs synaptogenic activity. For analysis of APLP1 and Fe65/Fe65L1 knockout mice different immunochemical, electrophysiological and behavioral studies were performed.

**Results**

We observed pre- and postsynaptic localization of all APP family members and could show that they form trans-directed dimers [1] , modulated by metal (copper/zinc) and heparin binding [2]. Further, heterologous expression of APP/APLPs in non-neuronal cells induces presynaptic differentiation in contacting axons of co-cultured neurons, similar to other synaptic adhesion molecules (SAMs). Finally, we show that Fe65/Fe65L1 knockouts have spatial learning and memory deficits and severe motor impairments, hippocampal LTP deficits and neuromuscular junction (NMJ) abnormalities. Notably, NMJ deficits were aggravated in APLP2/FE65-DKO and APLP2/FE65L1-DKO mice when compared to single FE65- and FE65L1-KO mice [3].

**Discussion**

Our data demonstrate that APP/APLPs form trans-directed dimers, possibly organizing in interplay with copper, zinc and heparin the pre- and postsynaptic site, essential for proper synapse formation/maintenance. Finally, as the Fe65/Fe65L1 knockout mice deficits resemble those of mutant APP mice, lacking the Fe65 binding site, our data suggest a central role for Fe65 proteins at the synapse possibly downstream of APP/APLPs.

**Conclusions**

Together, our results demonstrate that t he APP family members have typical features of SAMs, likely involved in interplay with Fe65 in synapse formation and maintenance.

binding to the growth factor-like domain. J Neurosci 2014, 34:11159 -11172. 3. Strecker P, Ludewig S, Rust M, Mundinger TA, Gorlich A, Krachan EG, Mehrfeld C, Herz J, Korte M, Guenette SY, Kins S: FE65 and FE65L1 share common synaptic functions and genetically interact with the APP family in neuromuscular junction formation. Sci Rep 2016, 6:2565

## P1-A24 STRUCTURAL EVALUATION OF THE EFFECT OF GLYCATION ON ABETA AGGREGATION

Giulia Milordini[1], Alessandro Emendato[2], Elsa Zacco[1], Annalisa Pastore[1], Delia Picone[2]
[1]Maurice Wohl Clinical Neuroscience Institute, King's College London, London, UK; [2]Department of Chemistry, University of Naples, via Cinthia, Napoli, Italy
*Molecular Neurodegeneration* 2019, **14(Suppl 1):**POSTER

**Correspondence:** Giulia Milordini

**Background**

Glycation, non-enzymatic addition of sugars to proteins, can be a pathological process leading to misfolding of the Abeta peptide into amyloids, hallmark of Alzheimer's disease (AD). [1,2] We aim to define the effects of glycation on the aggregation propensity of the peptides Abeta40 and Abeta42. These results could help understanding the high incidence of AD in Type 2 Diabetes (T2D) patients.

**Methods**

Methylglyoxal (MGO) was used as glycating agent. Circular dichroism illustrated the secondary structure variation upon glycation. Fluorescence-based assays and mass spectroscopy elucidated glycation and aggregation kinetics. High-resolution structural studies via atomic force microscopy (AFM) evaluated the morphological effects of glycation on amyloid fibrils.

**Results**

Abeta40 glycation allows the protein to retain its random coil conformation, while the non-glycated peptide forms amyloids after 24 hours. No significant variation of the secondary structure was observed upon glycation of Abeta42. However, the aggregation-associated fluorescence of both glycated Abeta40 and Abeta42 compared to the nonglycated versions shows a decrease in the final amount of total aggregates and a slower kinetics of aggregation with in MGO-concentration dependent fashion. AFM illustrates that the fibril formation is decelerated, and the peptides are found for a longer time in an oligomeric state.

**Discussion**

The glycation of Abeta 40 and Abeta42 has a strong impact on their structural stability and behavior [3]. The slower aggregation kinetics upon glycation translates into the stabilization of oligomeric forms, considered more toxic than the amyloids. These results could explain the higher incidence of AD in T2D patients.

**Conclusions**

Post-translational modifications can affect the structural behavior of aggregation-prone proteins and might be used to interfere with the development of AD and T2D.

## P1-A25 ZINC AND COPPER DIFFERENTIALLY MODULATE AMYLOID PRECURSOR PROTEIN PROCESSING BY γ-SECRETASE AND AMYLOID-β PEPTIDE PRODUCTION

Hermeto Gerber[1,2,3], Fang Wu[2,4], Mitko Dimitrov[2], Guillermo M. Garcia Osuna[2], and Patrick C. Fraering[1,2]
[1]Foundation Eclosion, Plan-Les-Ouates & Campus Biotech Innovation Park, Geneva, Switzerland; [2]Brain Mind Institute and School of Life Sciences, Swiss Federal Institute of Technology (EPFL), Lausanne, Switzerland; [3]Department of Biology, University of Fribourg, Switzerland; [4]Key Laboratory of Systems Biomedicine, Ministry of Education, Shanghai Center for Systems Biomedicine, Shanghai Jiao Tong University, Shanghai, China
*Molecular Neurodegeneration* 2019, **14(Suppl 1):**POSTER

**Correspondence:** Hermeto Gerber

**Background**

Recent evidence suggests involvement of biometal homeostasis in the pathological mechanisms of Alzheimer's disease (AD). Cu2+ and Zn2+ bind Aβ peptides and the amyloid precursor protein (APP) with high affinity (1-3), and are involved in Aβ oligomerization (1,4). However, little is known about whether these biometals modulate Aβ production.

**Methods**

Cell-free and cell-based assays were used to demonstrate that zinc and copper regulate Aβ production by affecting the γ-secretase processing of its Aβ precursor protein substrate APP-C99.

**Results**

We found that Zn2+ induces APP-C99 dimerization, which prevents its cleavage by γ-secretase and Aβ production, with an IC50 value of 15 μM. Importantly, at this concentration, Zn2+ also drastically raised the production of the aggregation-prone Aβ43 found in the senile plaques of AD brains (5). The APP-C99 histidine residues H6/H13/H14 control the Zn2+-dependent APP-C99 dimerization and inhibition of Aβ production, while the increased Aβ43:Aβ40 ratio is involves the known Zn2+-binding lysine K28 residue that orientates the APP-C99 transmembrane domain within the lipid bilayer. Unlike zinc, copper inhibited Aβ production by directly targeting the subunits presenilin and nicastrin in the γ-secretase complex. Discussion The human brain concentrations of bioavailable Cu2+ and Zn2+ (70 μM and 350 μM, respectively) are compatible with a physiological biometal-dependent modulation of Aβ production. Importantly, inhibition of the processing of protein receptors involved in synaptic activity, such as Neurexin or Cadherin, could also contribute to the pathogenesis of AD.

**Conclusions**

Altogether, our data demonstrate that zinc and copper differentially modulate Aβ production. They further suggest that dimerization of APP-C99, or the specific targeting of individual residues regulating the production of the long, toxic Aβ species, offer two therapeutic strategies for preventing AD.

P1-B01 MICROGLIAL MODIFICATION AS THE THERAPEUTIC STRATEGY FOR NEURODEGENERATIVE DISEASES

**Correspondence:** Min-Young Noh

**Background**

Microglia are the innate immune cells of the CNS and play important roles in neuroinflammation related with neurological disorders. Yet, studying human microglia is challenging because of the rarity and difficulty in acquiring primary cells from human fetal or adult CNS tissue. We aimed to develop and characterize the microglia cell model reflecting adult microglial character from peripheral blood mononuclear cells, and to study their function by disease progression in Amyotrophic lateral sclerosis (ALS).

**Methods**

In this study, the functional characteristics of slow ALS (ALS (S)) and ALS (ALS (R)) were analyzed after establishment of microglia-like cells (iMG) model in human mononuclear cells. Induced microglial cells (iMG) were generated that the adherent cells (monocytes) isolated from peripheral blood mononuclear cells were culture with GM-CSF and IL-34 for 21 days.

**Results**

We find that iMG express appropriate markers and function as primary human microglia. Functional assessment of iMG reveals that they secrete cytokines in response to inflammatory stimuli, migrate, and robustly phagocytosis function. Furthermore, whole-transcriptome analysis demonstrates that they are highly similar to cultured adult human microglia. iMG from rapidly progressing patients (Rapid-iMG) shows dysmorphic morphology and severe impaired phagocytosis function. Transcriptome analysis exhibits that low NCKAP1 expression is associated with impaired phagocytosis in Rapid-iMG. Finally, overexpression of NCKAP1 gene in ALS(R) iMG restored dysfunctional phagocytosis and knockdown of NCKAP1 gene in ALS(S) iMG decreased phagocytosis.

**Conclusions**

Taken together, these findings demonstrate that iMG can be used to study microglial function, providing important new insight into human neurological disease and microglia modification by NCKAP1 gene may be a potentially useful therapeutic strategy for Neurodegenerative diseases.

P1-B02 THE ROLE OF MICROGLIAL ALTERATIONS IN THE EARLY-LIFE STRESS-INDUCED MODULATION OF ALZHEIMER'S DISEASE PATHOLOGY IN MICE

**Correspondence:** Janssen Kotah

**Background**

Early-life stress (ES) is proposed to increase risk for aging-related dementias like Alzheimer's disease (AD). However, neurobiological substrates of such effects are not well-understood. We recently described ES to age-dependently alter microglial marker expression in wildtype (WT) mice, as well as progression of and response to amyloid-β (Aβ) pathology in an AD model. To further investigate the role of microglia in ES-induced vulnerability to AD, we investigated how ES alters the microglial response to i) acute LPS challenges in vitro, ii) chronic challenges in the form of amyloid accumulation in transgenic AD mice and iii) aging in WT mice.

**Methods**

We exposed WT C57BL/6 and APPswe/PS1dE9 mice to the limited nesting and bedding model from postnatal days (PND) 2-9. We measured gene expression in i) microglia isolated from PND9 WT mice exposed to LPS in vitro; ii) APP/PS1 mice at 10 months; and iii) WT mice followed up to 4, 10, and 20 months. We also assessed internalization of coated beads by isolated microglia, as well as microglial proliferation and clustering around Aβ plaques in the APP/PS1 tissue.

**Results**

ES-derived microglia increased pro-inflammatory cytokine expression in response to LPS, and internalized more latex, but not Aβ, beads. ES dampened the APP/PS1-induced increase in HMGB1, NLRP3, and CCL2 expression at 10 months, as well as decreased microglial proliferation (Iba1+/BrdU+ cells) but not clustering (Iba1+ cells) around Aβ plaques. ES did not alter aging-induced changes in microglial priming gene expression in WT mice.

**Discussion**

Overall, ES seems to alter microglial response to acute and chronic challenges in an agedependent manner, but not aging-induced changes in inflammatory genes. In vivo LPS studies are ongoing.

**Conclusions**

Insights on how ES alters microglial responses to different immune challenges will be key to understanding how it might modulate progression of diseases like AD.

P1-B03 INHIBITION OF APOPTOSIS SIGNAL-REGULATING KINASE 1 REDUCES GLIOVASCULAR INFLAMMASOME AFTER ISCHEMIC INJURY

**Correspondence:** So Yeong Cheon

**Background**

Ischemic injury triggers inflammatory mediator production, which is one of the main factors in pathology of ischemic stroke [1, 2]. Inflammasomes are innate immune complex, are involved in pathogenesis of diseases, such as atherosclerosis, and Alzheimer's disease [3]. The previous studies have demonstrated that endothelial cells and astorcytes are closely assotiated with nucleotide binding oligomerization domain (NOD)-like receptor (NLR) protein (NLRP) inflammasome [4, 5]. Apoptosis signal-regulating kinase 1 (ASK1) is closely related to the inflammatory response and is involved in productions of inflammation-related mediators [6].

**Methods**

Therefore, in this study, we investigated whether ASK1 affects inflammasomes in astrocytes and endothelial cells under ischemic condition which is performed in vivo study by using middle cerebral artery occlusion/reperfusion model in C57BL6 mice. Alteration of inflammasome-associated components was confirmed by real-time PCR.

**Results**

Our data showed that ICAM-1, endothelial cell activation marker, and GFAP, reactive astrocyte maker, were upregulated after ischemic injury. Also, NLRP2 and NLRP3 were increased in the ischemic cortex and striatum respectively. Inflammasome components, such as ASC and caspase-1, are efficiently upregulated after ischemic injury. Also, inflammatory cytokines, including IL-1β, and IL-18 were increased in the brain lesion. However, inhibition of ASK1 by small interfering RNA significantly decreased the levels of NLRP2, NLRP3, ASC, and caspase-1, thereby reducing IL-1β, and IL-18 levels in the cerebral cortex and striatum respectively.

## Conclusions

Collectively, inhibition of ASK1 may be novel strategy for reduction of inflammasomes after ischemic stroke.

## P1-B04 DIFFERENT CO-LOCALIZATION OF NEUROINFLAMMATORY MARKERS IN PLAQUES OF ATYPICAL COMPARED TO TYPICAL ALZHEIMER'S DISEASE

Baayla DC Boon[1, 2], Jeroen JM Hoozemans [2], Boaz Lupohaä[2], Kristel N Eigenhuis[2], Philip Scheltens[1], Annemieke JM Rozemuller[2], Femke H Bouwman[1]

[1]Department of Neurology, Alzheimer Center, Neuroscience Campus Amsterdam, VU University Medical Center; [2]Department of Pathology, Neuroscience Campus Amsterdam, VU University Medical Center; [3]Department of Anatomy and Neurosciences, Neuroscience Campus Amsterdam, VU University Medical Center

*Molecular Neurodegeneration* 2019, **14(Suppl 1):**POSTER

**Correspondence:** Baayla DC Boon

## Background

An atypical presentation of Alzheimer's disease (AD) is seen in 30% of patients with early disease onset [1]. This atypical presentation is reflected by a different spreading of neurofibrillary tangles (NFT) than originally proposed by Braak and Braak [2,3,4]. Here we aimed to explore whether neuroinflammation is associated with an atypical spreading of pathology in AD.

## Methods

Typical and atypical AD cases were selected based on both NFT distribution and amnestic or non-amnestic clinical presentation. Immunohistochemistry was performed on the temporal pole and superior parietal lobe of 10 typical and 9 atypical AD cases for the presence of amyloid-beta (Nterminal; IC16), pTau (AT8), microglia (Iba1, CD68, and HLA-DP/DQ/DR), as well as complement (C3d and C4b) and was quantified by image analysis. To study co-localization of neuroinflammatory markers with amyloid(-beta), immunofluorescence triple-stainings were performed on a subset of typical and atypical AD cases.

## Results

The distribution of pTau, activated microglia (CD68 and HLA-DP/DQ/DR), and complement (C3d, and C4b) differed between AD variants. Typical AD cases showed a temporal dominant distribution of these markers, whereas atypical AD cases showed a parietal dominant distribution. We found a temporal dominant distribution for amyloid-beta and Iba1 positive microglia in both typical and atypical AD. Interestingly, the morphology of amyloid-beta plaques differed between the two subtypes. Compared to typical AD cases, atypical AD cases showed a more coarse plaque type in the parietal cortex. In addition, these atypical plaques showed an altered localization of activated microglia and complement compared to classical plaques in typical AD cases.

## Conclusions

Our data indicate that neuroinflammation is related to a different atypical plaque morphology and occurs alongside the atypical distribution of tau pathology in atypical AD, suggesting that neuroinflammation might be a crucial link between amyloid-beta deposits, tau pathology, and clinical symptoms.

## P1-B06 ENRICHMENT-DEPENDENT DEFICITS IN HIPPOCAMPAL NEUROGENESIS MEDIATED BY FAMILIAL ALZHEIMER'S DISEASE-LINKED PS1 VARIANTS IS RESCUED BY MICROGLIAL DEPLETION

Sylvia Ortega-Martinez, Nisha Palla, Xiaoqiong Zhang, Myles R. Minter, Pablo Oyler-Castrillo, Sangram S. Sisodia

Department of Neurobiology, The University of Chicago, Chicago, IL, 60637, USA

*Molecular Neurodegeneration* 2019, **14(Suppl 1):**POSTER

**Correspondence:** Sylvia Ortega-Martinez

## Background

Presenilin 1 (PS1) plays a critical role in neurogenesis. Hippocampal neurogenesis is required for the normal cognitive and emotional function of the brain, and constitutes a key target in Alzheimer Disease (AD). We have demonstrated that ubiquitous expression of familial, early-onset Alzheimer's disease (AD)-linked PS1 (FAD-PS1) mutants impairs environmental enrichment (EE)-induced proliferation and neurogenesis of adult hippocampal progenitor cells (AHNPCs) in a non-cell autonomous manner (1, 2, 3). These impairments are, at least in part, due to alterations in the levels of specific chemokines and growth factors secreted from microglia expressing FAD-PS1 variants.

## Methods

1 month-old male mice expressing PrP promoter-driven human wild-type PS1, M146L and ΔE9 were fed with PLX5562 for 7 days, then subject to Standard Housing or EE conditions for 1 month. PLX5622 is a CSF1 receptor antagonist, used to deplete microglia in adult brain. Animals were injected with a single bolus of BrdU, and sacrificed after 24 hours or 2 weeks. Baseline anxiety behavior was tested using Marble Burying and Dark/light test. Brain immunostaining was used to assess proliferation, neurogenic cell density, differentiation and survival of hippocampal progenitors. Quantification was performed using unbiased stereological methods (2, 3).

## Results

Compared with mice expressing human WT PS1, mice expressing FAD-PS1 linked mutations exhibit lower rates of proliferation, neural stem cells and GFAP+ cells in the hippocampus following EE. These deficits were correlated with higher rates of baseline anxiety behaviors. PLX5622-mediated depletion of microglia in mice expressing FAD-PS1 linked variants rescued the deficits in AHNPC proliferation and differentiation and aberrant baseline anxiety.

## Conclusions

PLX5622-mediated depletion of microglia in mice expressing FAD-PS1 linked variants rescues deficits in AHNPC proliferation and baseline anxiety of those mice. These findings reinforce the important role of microglia in the regulation of neurogenesis in FAD-PS1 models.

**Acknowledgments**
Supported by NIH AG027854, Cure Alzheimer's Fund (CAF), and the Edward H. Levi Fund SSS is a paid Consultant of Eisai Research Labs Inc., AZTherapies Inc.

## P1-B07 NLRP3/CASPASE-8 INFLAMMASOME DRIVES PRIMARY PROGRESSIVE MULTIPLE SCLEROSIS

Federica Piancone[1], Marina Saresella[1], Ivana Marventano[1], Francesca La Rosa[1], Maria Antonia Santangelo[2], Domenico Caputo[3], Laura Mendozzi[3], Marco Rovaris[3], Mario Clerici[1,4]
[1]Laboratory of Molecular Medicine and Biotechnology, Don Gnocchi Foundation, IRCCS, Milan, Italy; [2]Laboratory of Clinical Analysis, Don Gnocchi Foundation, IRCCS, Milan, Italy; [3]Department of Neurology, Don Gnocchi Foundation, IRCCS, Milan, Italy; [4]Department of Physiopathology and Transplants, University of Milano, Milan, Italy
*Molecular Neurodegeneration* 2019, **14(Suppl 1):**POSTER

**Correspondence:** Federica Piancone

**Background**

Inflammasome-driven neuroinflammation is postulated to play a role in Multiple Sclerosis (MS), but there is no direct evidence that the nod-like receptor protein 3 (NLRP3) inflammasome is involved in MS pathogenesis. Uric acid was shown to be one of the 'danger' signals involved in the activation of NLRP3 inflammasome; notably, the concentration of uric acid is increased in the serum and in the CSF of MS individuals.

**Methods**

PBMCs from 41 MS individuals with a diagnosis of primary progressive MS (PPMS), stable relapsing-remitting MS (SMS), acute relapsing-remitting MS (AMS), or benign MS (BMS) and from 10 age and sex matched healthy controls (HC) were primed with LPS and stimulated with Monosodium Urate Crystals (MSU). The expression of the NLRP3, ASC, caspase-1, caspase-8, IL-1β and IL-18 inflammasome genes was evaluated by RT-PCR whereas production of the pro-inflammatory cytokines IL-1β and IL-18 was measured by ELISA in supernatants.

**Results**

Results showed that: 1) uric acid serum concentration was significantly increased in PPMS alone compared to all other groups; 2) mRNA for NLRP3, ASC and IL-18 was up regulated in PPMS and in AMS patients, as well, but caspase-8 mRNA was up regulated only in PPMS; 3) IL-18 production was significantly increased in PPMS alone, in whom a direct correlation between hyperuricemia and caspase-8 was detected.

**Discussion**

Taken together our results suggest that in PPMS patients a possibly prolonged and chronic stimulation would results in the up-regulation of mRNA expression of NLRP3, ASC, caspase-8 and IL-18 genes and of IL-18 pro-inflammatory cytokine; this could be justified by the observation that hyperuricemia is present in PPMS patients.

**Conclusion**

The NLRP3/caspase-8 inflammasome pathway is activated in PPMS, possibly as a consequence of hyperuricemia. Therapeutic strategies reducing NLRP3 activation and/or lowering hyperuricemia could be useful in the therapy of PPMS.

Grant by Italian Ministry of Health : Ricerca Corrente 2016-17

## P1-C01 CSF GROWTH ASSOCIATED PROTEIN-43 IS A PRESYNAPTIC ALZHEIMER'S DISEASE SPECIFIC BIOMARKER ASSOCIATED WITH TAU AND AMYLOID PATHOLOGY

Åsa Sandelius[1], Erik Portelius[1, 2], Åsa Källén[2], Henrik Zetterberg[1, 2, 3, 4], Uros Rot5, Bob Olsson[1, 2], Jon B Toledo[6,7], Leslie M. Shaw [6], Virginia M.Y. Lee[6], David J. Irwin[6], Murray Grossman[8], Daniel Weintraub[9,10], Alice Chen-Plotkin[8], David A. Wolk[8], Leo McCluskey[8], Lauren Elman8, Jennifer McBride6, John Q. Trojanowski[6], Kaj Blennow[1, 2]
[1] Institute of Neuroscience and Physiology, Department of Psychiatry and Neurochemistry, the Sahlgrenska Academy at the University of Gothenburg, Mölndal, Sweden; [2] Clinical Neurochemistry Laboratory, Sahlgrenska University Hospital, Mölndal, Sweden; [3] UK Dementia Research Institute, WC1N London UK; [4] Department of Molecular Neuroscience, UCL Institute of Neurology, Queen Square, WC1N London UK; [5] Department of Neurology, University Medical Centre, Ljubljana, Slovenia; [6] Department of Pathology and Laboratory Medicine, Institute on Aging, Center for Neurodegenerative Disease Research, University of Pennsylvania School of Medicine, Philadelphia, PA, USA; [7] Department of Neurology, Houston Methodist Hospital, Houston, TX, USA; [8] Department of Neurology, University of Pennsylvania School of Medicine, Philadelphia, PA; [9] Departments of Psychiatry and Neurology, University of Pennsylvania School of Medicine, Philadelphia, PA USA; [10]Parkinson's Disease and Mental Illness Research, Education and Clinical Centers (PADRECC and MIRECC), Philadelphia Veterans Affairs Medical Center, Philadelphia, PA, USA
*Molecular Neurodegeneration* 2019, **14(Suppl 1):**POSTER

**Correspondence:** Åsa Sandelius

**Background**

Synaptic dysfunction is a core feature of Alzheimer's disease (AD) with a strong correlation to cognitive dysfunction and affected early in the disease. Novel treatments specifically targeting AD pathology are currently under development and likely to be most effective early. This increases the need for early and reliable disease detection. Here we evaluated the biomarker potential of the presynaptic protein Growth-Associated Protein 43 (GAP-43) and its associations to neurodegenerative disease pathology.

**Methods**

We developed and characterized a novel in house enzyme-linked immunosorbent assay (ELISAs) for GAP-43 quantification in CSF and measured a pilot cohort and a large cohort of neurologically normal controls (n = 43), AD patients (n = 275) and other neurodegenerative disorders (n= 348), such as mild cognitive impairment (MCI), Parkinson's disease (PD), dementia with Lewy Bodies, frontotemporal dementia (FTD) and amyotrophic lateral sclerosis (ALS). In a subpopulation (n = 93), neuropathological analysis of Aβ plaques, tau neurofibrillary tangle, α-synuclein and TDP-43 was performed.

**Results**

CSF GAP-43 was significantly increased in AD compared to controls and MCI patients. Furthermore, CSF GAP-43 concentrations were significantly increased in AD compared to ALS, FTD, PD and atypical parkinsonian diseases.

There was no difference in GAP-43 concentrations between AD and primary progressive aphasia or corticobasal syndrome patients, which often co-occur with AD pathology. CSF GAP-43 concentration correlated with Mini Mental State Exam and the magnitude of neurofibrillary tangles and Aβ plaques in the hippocampus, amygdala and cortex. No associations could be detected to α-synuclein or TDP-43 pathology.

**Discussion**

The results suggest a specific association between GAP-43, tau and amyloid pathology in AD, however, the biological connection between the three is yet to be demonstrated.

**Conclusions**

CSF GAP-43 concentration may serve as a pathology response marker that would be interesting to evaluate in disease-modifying drug trials against AD.

P1-C02 APPLICATION OF AFFIBODY ZSYM73, A NOVEL, NON-ANTIBODY PROTEIN, FOR STUDYING PATTERNS OF Aβ PEPTIDES IN HUMAN CEREBROSPINAL FLUID

M. Rozga[1], K. Dillner Bergstedt[2], F. Frejd[2], E. Portelius[1,3], K. Blennow[1,3], H. Zetteberg[1,3], K. Höglund[1,3]
[1]Institute of Neuroscience and Physiology, Department of Psychiatry and Neurochemistry, Mölndal, Sweden; [2]Affibody AB, Affibody AB, Stockholm, Sweden; [3]Sahlgrenska University Hospital, Clinical Neurochemistry Laboratory, Mölndal, Sweden
*Molecular Neurodegeneration* 2019, **14(Suppl 1):**POSTER

**Correspondence:** M. Rozga

**Background**
Aβ peptides are centrally involved in the pathogenesis of AD pathology. Proteomic analysis of brain and cerebrospinal fluid (CSF) revealed that Aβ is de facto a heterogeneous mixture of more than 40 peptides possessing different chain length and post-translational modifications [1]. The ZSYM73 Affibody represents a novel class, non-antibody affinity protein, designed for selective and tight binding of the Aβ peptide [2]. The aim of this study was to assess the binding profile of ZSYM73 and evaluate the potential of ZSYM73 as a convenient and robust tool for mapping endogenous Aβ peptides in biological samples.

**Methods**
Immunoprecipitation (IP) of Aβ from CSF and brain tissue using ZSYM73 and anti-Aβ antibodies 6E10 and 4G8, followed by matrix assisted laser desorption ionization time-of-flight mass spectrometry (MALDI-TOF MS) analyses.

**Results**
Immunoprecipitation of human CSF with ZSYM73 allowed measurement of nine Aβ peptides starting at amino acid position 1, 5 or 11 and ending at position from 37 up to 43. A total number of Aβ peptides detected in insoluble brain fraction was sixteen: three beginning at amino acid position 1 and thirteen cleaved at seven different positions within the N-terminal part of the peptide chain. In addition to monomeric Aβ, ZSYM73 recognizes and binds Aβ dimers and trimers.

**Discussion**
The epitope for ZSYM73 is located outside the first eleven N-terminal amino acids of the Aβ sequence. The last 5-7 residues of the Aβ C-tail are crucially involved in the interaction between Aβ and ZSYM73 and possibly stabilization of the Aβ-ZSYM73 complex. ZSYM73 offers improved IP yield comparing to 6E10 or 4G8 antibodies.

**Conclusions**
Our data demonstrate the utility and robustness of ZSYM73 as a novel, non-antibody affinity protein to detect and measure Aβ peptides. The combination of ZSYM73 with anti-Aβ antibodies allows for a more detailed and complete mapping of Aβ isoforms in human samples.

**References**
1. Mansson JE, Nilsson J et al: An online nano-LC-ESI-FTICR-MS method for comprehensive characterization of endogenous fragments from amyloid beta and amyloid precursor protein in human and cat cerebrospinal fluid. J Mass Spectrom 2012, 47(5):591-603.
2. Lindberg H, Hard T, Lofblom J, Stahl S: A truncated and dimeric format of an Affibody library on bacteria enables FACS-mediated isolation of amyloid-beta aggregation inhibitors with subnanomolar affinity. Biotechnol J 2015, 10(11):1707-1718.

P1-C03 DETECTION OF NEUROGRANIN COMPLEXES OF ~38 kD AND ~12 kD IN CSF

Bruno Becker, Elena Camporesi, Henrik Zetterberg, and Kaj Blennow
Institute of Neuroscience and Physiology, Department of Psychiatry and Neurochemistry, The Sahlgrenska Academy at University of Gothenburg, S-405 30 Gothenburg, Sweden
*Molecular Neurodegeneration* 2019, **14(Suppl 1):**POSTER

**Correspondence:** Bruno Becker

**Background**
The 7.5 kD postsynaptic protein neurogranin (Ng) is being evaluated as a biomarker in CSF for early and advanced stages of Alzheimer disease (AD). Although its name implies granular aggregates of the protein, complexes of Ng have so far not been detected in CSF.

**Methods**
Neurogranin was immunoprecipitated from CSF using monoclonal antibody NG36 immobilized on magnetic beads. After washing, the beads were extracted in non-reducing and reducing SDS sample buffer and analyzed via western blots. The detection antibody was biotinylated NG2 monoclonal antibody.

**Results**
Complexes of an apparent size of approximately 38 kD and monomeric Ng (about 12 kD) were detected in Ng immunoprecipitated from CSF on Western blots under non-reducing conditions. This indicates the presence of either trimeric to tetrameric Ng or of complexes of similar size with other proteins in the samples. The complex was sensitive to reduction, therefore disulfide bridges are likely to be involved in complex formation.

**Discussion**
For quantitative assays of Ng it is important to be aware of the molecular species present. Judging by the apparent size of the complex, a Ng trimer seems most likely, although a Ng tetramer or the presence of other proteins cannot be ruled out. Furthermore, artefactual complex formation via intermolecular disulfide bridge formation upon oxidation, for example during sample preparation or SDS PAGE, is possible. Therefore, we intend to perform MS analysis of the excised complex, and use SDS-PAGE independent methods for complex detection, such as homogeneous ELISA (same capture and detection antibody).

**Conclusions**
Western blot data from immunoprecipitated samples of CSF indicates the presence of higher molecular weight complexes of Ng. It will be of interest to analyze the nature of the complex and to see whether the extent of complex formation differs between samples from AD patients and controls.

P1-C04 THE PLASMA PROTEIN AND ACTIVITY OF CHOLINE ACETHYLTRANSFERASE IN PATIENTS WITH ALZHEIMER'S DISEASE COMPARED TO CONTROLS

Azadeh Karami[1, 3], Taher Darreh-Shori[1], Henrik Zetterberg[2,4,5,6], Kaj Blennow[2,4,5,6], Maria Eriksdotter[3,7]
[1]Center for Alzheimer Research, Division of Translational Alzheimer Neurobiology, Department of Neurobiology, Care Sciences and Society, Karolinska Institutet, Stockholm, Sweden; [2]Clinical Neurochemistry Laboratory, Sahlgrenska University Hospital, Mölndal, Sweden; [3]Center for Alzheimer Research, Division of Clinical Geriatrics, Department of Neurobiology, Care Sciences and Society, Karolinska Institutet, Stockholm, Sweden; [4]Department of Psychiatry and Neurochemistry, Institute of Neuroscience and Physiology, the Sahlgrenska Academy at the University of Gothenburg, Mölndal, Sweden; [5]Department of Molecular Neuroscience, UCL Institute of Neurology, Queen Square, London, UK; [6]UK Dementia Research Institute at UCL, London, UK; [7]Theme Aging, Karolinska University Hospital, Huddinge, Sweden
*Molecular Neurodegeneration* 2019, **14(Suppl 1):**POSTER

**Correspondence:** Azadeh Karami

**Background**
One of the most prevalent forms of dementia is Alzheimer's disease (AD), the symptoms of which are deterioration of memory loss and cognitive functions brought about partly by a decrease in cholinergic neurotransmission.
Being incurable, treatment with cholinesterase inhibitors (ChEI) brings symptoms relief. Choline acetyltransferase (ChAT), the enzyme responsible for biosynthesis of acetylcholine (ACh), has recently been found in both the cerebrospinal fluid and plasma. Given that extracellular ChAT may regulate extrasynaptic levels of ACh, it may prove to be crucial in AD patients' prognosis and cognitive performance.

**Methods**
A recently developed integrated sandwich ELISA protocol was used to measure the activity and protein concentration of ChAT in plasma samples. Totally 181 samples were collected from 32 AD, 30 MCI and

22 SCI patients at the time of diagnosis at a memory clinic and 97 samples obtained from a group of age-matched healthy controls (HC) from the Gothenburg biobank.

**Discussion**

Among patients with MCI and AD, ChAT activity and protein were the highest, while the values were lower for SCI-diagnosed patients, and lowest for HC [ChAT protein concentration: p<0.0016 for AD vs. MCI, p < 0.0002 for AD vs. SCI, p < 0.0001 for AD vs. HC, MCI vs HC p< 0.0001, SCI vs HC p< 0.0001 and ChAT activity: p< 0.0448 for AD vs. SCI].

**Conclusions**

This study shows for the first time, that plasma ChAT activity and protein levels differ in AD and MCI vs SCI patients and healthy controls, making this an interesting marker to further explore in the quest for biomarkers which can distinguish patients in different cognitive stages.

## P1-C05 BASELINE LEVELS OF CSF TAU FRAGMENTS ENDING AT AMINO ACID 123 AND 224 ARE ASSOCIATED WITH RATE OF COGNITIVE DECLINE IN ALZHEIMER'S DISEASE

Claudia Cicognola[1], Erik Portelius[1,2], Lucilla Parnetti[3], Kaj Blennow[1], Kina Höglund[1,2,4]
[1]Institute of Neuroscience and Physiology, Department of Psychiatry and Neurochemistry, The Sahlgrenska Academy at University of Gothenburg, Mölndal, Sweden; [2]Clinical Neurochemistry Laboratory, Sahlgrenska University Hospital, Mölndal, Sweden; [3]Department of Medicine, Neurology Clinic, University of Perugia, Ospedale Santa Maria della Misericordia, Perugia, Perugia, Italy; [4]Department of Neurobiology, Care Sciences and Society, Center for Alzheimer Research, Division for Neurogeriatrics, Karolinska Institutet, Novum, Huddinge, Stockholm, Sweden
*Molecular Neurodegeneration* 2019, **14(Suppl 1):**POSTER

**Correspondence:** Claudia Cicognola

**Background**

Tau pathology is a hallmark of Alzheimer's disease (AD). Recent data suggest that tau is present in the cerebrospinal fluid (CSF) as a series of fragments. Through immunoprecipitation-mass spectrometry (IPMS), we have identified two abundant CSF pools of tau ending at amino acid (aa) 123 and 224. Our aim was to assess if baseline CSF concentrations of these fragments is associated to cognitive decline in AD.

**Methods**

CSF samples from 16 AD, 38 mild cognitive impairment (MCI), 20 MCI due to AD (MCI-AD) and 21 other neurological diseases (OND) subjects were collected at baseline. Subjects were clinically diagnosed, with AD CSF biomarkers (Abeta42, total tau, phosphorylated tau) used as support. Cognitive assessment at follow-up was performed through Mini Mental State Examination (MMSE) every 6 months for ~2.3 years. Tau 123 and 224 fragments were measured with in-house ELISA and single-molecule array (Simoa) assays, respectively. Subjects were classified into quartiles based on the baseline concentrations and tested for association to disease progression, as examined by longitudinal change in MMSE.

**Results**

Baseline levels of tau 123 and 224 were significantly higher in AD vs. OND (p=0.03 and 0.0017, respectively), MCI-AD vs. MCI (p=0.0061; p= 0.0018) and MCI-AD vs. OND (p=0.0011; p<0.0001). MMSE score decreased significantly over years from baseline for the highest quartile (Q4) for both fragments (p <0.0001).

**Discussion**

Through IP-MS, we identified CSF pools of tau fragments ending at aa 123 and 224. Baseline CSF levels were successful in discriminating AD from OND, MCI-AD from MCI and MCI-AD from OND. Longitudinal deterioration in cognitive performance, as measured by MMSE, was associated to highest baseline levels of tau 123 and tau 224.

**Conclusions**

Baseline levels of CSF tau fragments ending at aa 123 and aa 224 might represent a prognostic biomarker for rate of cognitive decline in AD.

## P1-C06 PROTEOMICS ANALYSIS IDENTIFIES NEW MARKERS ASSOCIATED WITH CAPILLARY CEREBRAL AMYLOID ANGIOPATHY IN ALZHEIMER'S DISEASE

David C Hondius[1,2], Kristel N Eigenhuis[2], Tjado HJ Morrema[1], Roel C van der Schors[2], Pim van Nierop[2], Marianna Bugiani[1], Ka Wan Li[2], Jeroen JM Hoozemans[1], August B Smit[2†], Annemieke JM Rozemuller[1†]
[1]Department of Pathology, Amsterdam Neuroscience, VU University Medical Center, The Netherlands; [2]Department of Molecular and Cellular Neurobiology, Center for Neurogenomics and Cognitive Research, Amsterdam Neuroscience, VU University Amsterdam, The Netherlands
*Molecular Neurodegeneration* 2019, **14(Suppl 1):**POSTER

**Correspondence:** David C Hondius
[†]Authors with equal contribution

**Background**

In Alzheimer's disease (AD) amyloid beta (Aβ) aggregates in parenchymal plaques or around the brain vasculature, which is known as cerebral amyloid angiopathy (CAA). In CAA type 1 Aβ accumulates in both capillaries and larger vessels. CAA type 1 occurs in approximately 40% of all AD patients, contributes to the symptomatic manifestation of AD, and could even result in rapidly progressive dementia. The pathophysiology of CAA is elusive and biomarkers for CAA are warranted for stratification of patients involved in clinical trials and future therapy. The objective of this study is to identify proteins selectively involved in CAA by laser dissection assisted mass spectrometry analysis on postmortem human brain tissue.

**Methods**

For this study we selected postmortem human brain tissue of AD cases with severe plaque pathology (n=7), cases with severe CAA type 1 (n=7) and cognitively healthy control cases (n=6). Grey matter of the occipital cortex areas with high pathological burden were visualized with Aβ immunostaining and subsequently isolated by laser microdissection. Proteins were quantified using Orbitrap LC-MS/MS and MaxQuant software.

**Results**

Initial data analysis shows a clear distinction in the proteome between AD patients with and without CAA. By contrasting the experimental groups we were able to identify individual proteins that are specific for CAA pathology. Data obtained by mass spectrometry was confirmed using immunohistochemistry and immunoblotting.

**Conclusions**

The distinct changes identified in the proteome of CAA pathology provides insight in the biology of CAA and yields potentially valuable data on CAA biomarkers.

## P1-C07 TESTING NEUROFILAMENT LIGHT (NF-L) CHAIN IN BLOOD SAMPLES TO MONITOR NEURODEGENERATIVE DISEASES AND SUPPORT CLINICAL DRUG DEVELOPMENT: QUALIFICATION OF AN ULTRASENSITIVE IMMUNO-ASSAY USING CLINICAL SERUM AND PLASMA SAMPLES

Christine Bain, Marion Emeric-Bourgogne, Renaud Jasnowski, Cendrine Josson, Marc Essodaigui, Nicolas Anfossi
ABL, 321 avenue Jean Jaurès, 69007 Lyon, France
*Molecular Neurodegeneration* 2019, **14(Suppl 1):**POSTER

**Correspondence:** Christine Bain

**Background**

NF-L has been recently reported as a potential biomarker of neuronal damage in numerous neuroinflammatory disorders, including Alzheimer's disease (AD) [1], multiple sclerosis (MS) [2] or traumatic brain injury (TBI) [3]. Furthermore, NF-L levels in cerebrospinal fluid and blood have been reported as normalized following effective MS therapy [4]. In that context, we aimed at assessing the performance of an ultrasensitive method to quantify NF-L in human serum or plasma and evaluating its routine use to support clinical drug development.

**Methods**

Trueness, precision, parallelism, dilution linearity and lower limit of quantification (LLOQ) of the Simoa kit (QuanterixTM) have been assessed in serum and plasma samples from healthy donors and patients with neuroinflammatory disorders.

**Results**

Dosing endogenous NF-L levels demonstrated good precision of the method, when tested at the minimal required dilution with intra- and inter-run variability ranging from respectively 1.6% and 19.9% and 5.2 and 19.9% depending on the concentration level. The parallelism study shows that test samples can be serially diluted without impacting measured NFL concentration. Finally, based on these results, LLOQ could be set at 0.6 pg/mL in diluted blood matrix. Finally, levels of endogenous NF-L measured from 10 healthy donors did not differ significantly between paired serum (4.8-13.9 pg/mL) and plasma (4.4-10.9 pg/mL).

**Discussion**

Our results related to high performance and sensitivity of the Simoa-based method to dose NFL are in accordance with recent data [5], strengthening the benefit to dose circulating NF-L in clinical samples using this technology.

**Conclusions**

NF-L is now ready to be tested with accuracy and high sensitivity in both human serum and plasma samples. As these matrices are easy to collect and store frozen in the context of clinical trials, NF-L testing can now support clinical drug development in many neurodegenerative pathologies, either in prospective or retrospective settings.

## P1-C08 BRAIN CREATINE KINASE (CKB): A MARKER FOR OXIDATIVE STRESS IN EARLY ALZHEIMER'S DISEASE

Rebecca Sjöberg[1], Jinar Rostami[2], David Kotol[3], Nicholas Mitsios[1], Fredrik Edfors[3], Anna Erlandsson[2], Mathias Uhlén[1,3], Jan Mulder[1]

[1]Department of Neuroscience, Karolinska Institute, Stockholm, Sweden; [2]Department of Public Health and Caring Science, Uppsala University, Uppsala, Sweden; [3]Department of Proteomics and Nanobiotechnology, Royal Institute of Technology, Stockholm, Sweden

*Molecular Neurodegeneration* 2019, **14(Suppl 1):**POSTER

**Correspondence:** Rebecca Sjöberg

**Background**

Alzheimer's disease (AD) is an age-related neurodegenerative disease without any treatment or cure. Available therapeutics only aim to improve cognitive functions and delay disease progression. It is reported that the onset of pathological changes, including oxidative stress, precede clinical presentation of symptoms, this poses a need for diagnostic markers that enable early detection of alterations associated with AD [1, 2]. Brain creatine kinase (CKB) is an enzyme that regulates available adenosine triphosphate (ATP) levels in the brain. It has been previously reported that CKB is sensitive to oxidation and reduced CKB activity is observed in the AD brain [3]. This could make CKB a potential marker for oxidative stress in AD.

**Methods**

Human post-mortem brain samples (frontal and temporal cortex) from patients with AD, Lewy body dementia (DLB), non-demented age-matched controls and nondemented controls aged 49-60 were used to quantify CKB on transcript level and protein level (using immunofluorescence, western blot and targeted mass spectrometry).

**Results**

We found CKB to be only expressed in astrocytes. CKB was up-regulated on transcript level but down-regulated on protein level in AD when compared to age-matched non-demented controls. In DLB and control subjects protein levels of CKB varied depending on degree of amyloid plaque load. No relationship between CKB levels and tangles or Lewy bodies was observed.

**Discussion**

Presence of amyloid beta peptides (especially 1-42) have shown to induce oxidative stress [4] and since CKB is sensitive to oxidation this consequently leading to loss of function and protein degradation. This explains the down-regulation on protein level of CKB when amyloid plaques are present.

**Conclusions**

CKB protein is down-regulated in AD likely due to protein oxidation. Diagnostics based on measuring reduced CKB activity or quantifying post-translational modifications of CKB could provide insights in the oxidative stress in the pre-clinical phase of disease.

## P1-C09 HIGH DETECTION SENSITIVITY WITH ANTIBODY-BASED PET RADIOLIGAND FOR AMYLOID BETA IN THE BRAIN

Xiaotian T. Fang[1], Greta Hultqvist[1,2], Silvio R. Meier[1], Gunnar Antoni[3,4], Dag Sehlin[1], Stina Syvänen[1]

[1]Department of Public Health and Caring Sciences / Geriatrics, Uppsala University, Uppsala, Sweden; [2]Department of Pharmaceutical Biosciences, Uppsala University, Uppsala, Sweden; [3]Department of Medicinal Chemistry, Preclinical PET Platform, Uppsala University, Uppsala, Sweden; [4]PET Centre, Uppsala University Hospital, Uppsala, Sweden

*Molecular Neurodegeneration* 2019, **14(Suppl 1):**POSTER

**Correspondence:** Xiaotian T. Fang

**Background**

Amyloid-beta (Aβ) PET has become an important aid in Alzheimer's disease diagnosis, and an inclusion criterion for enrolment of patients into clinical trials of new anti-Aβ treatments. All available Aβ PET radioligands bind to insoluble fibrils, i.e. Aβ plaques. Levels of prefibrillar Aβ forms, e.g. soluble oligomers and protofibrils, correlate better than plaques with disease severity, and these soluble forms are neurotoxic. The aim was to create an antibody-based radioligand, recognizing fibrillary Aβ, and also smaller, soluble aggregates. We designed and expressed a small recombinant bispecific antibody construct, di-scFv 3D6-8D3 targeting the Aβ N-terminus and the transferrin receptor (TfR).

**Methods**

Two mouse models were used in this study: tg-Swe (KM670/671NL) harbouring the Swedish AβPP, and tg-ArcSwe which has the Swedish and the Arctic (E693G) mutations. 3D6-8D3 was 124Ilabeled using

Chloramine-T. Animals were injected intraveneously with [124I]3D6-8D3 (6.0±2.2 MBq), then scanned 14, 24 and/or 72h post-injection.
**Results**
TfR, expressed at the blood-brain barrier, functioned as a shuttle between blood-brain. 3D6-8D3 bound to Aβ1-40 (Kd: 1.22 nM) and to TfR (Kd: 12.19 nM) pre- and post- radiolabeling with iodine-124. [124I]3D6-8D3 was retained in transgenic animals brain while it was cleared from the wild-type brain. This was observed from 24h onwards, and at 72h, 18 m.o. transgenes displayed SUVRbrain/cer of 2.2-3.5 while wildtype mice showed ratios close to unity. In a subset of mice scanned with [11C]PIB, wt mice displayed ratios of unity while transgenes showed slightly elevated SUVR of 1.2, indicating improved sensitivity with [124I]3D6- 8D3 compared with [11C]PIB. Brain concentrations of [124I]3D6-8D3 correlated with soluble Aβ ($p < 0.0001$), not with total Aβ, i.e. plaque load ($p = 0.34$).
**Discussion**
[124I]3D6-8D3 displayed better sensitivity than [11C]PIB, and brain concentrations correlated with soluble neurotoxic forms of Aβ.
**Conclusions**
We created a small bispecific antibody-based radioligand capable of crossing the BBB, subsequently binding to and visualizing intrabrain Aβ.

## P1-C10 LEVELS OF UNMODIFIED AND TOTAL IAPP IN RETINA AND BRAIN OF ALZHEIMER'S DISEASE PATIENTS

Nina Schultz[1], Elin Byman[1], the Netherlands Brain Bank[2], Malin Wennström [1]
[1]Department of Clinical Sciences Malmö, Clinical Memory Research Unit, Lund University, Malmö, Sweden, [2]Netherlands Institute for Neuroscience, Amsterdam, the Netherlands
*Molecular Neurodegeneration* 2019, **14(Suppl 1):**POSTER

**Correspondence:** Nina Schultz
**Background**
Recent studies show accumulation of islet amyloid polypeptide (IAPP) in the brain of Alzheimer's disease (AD) patients, suggesting an additional amyloid peptide, beside amyloid beta, to be implicated in AD [1, 2]. We have in a previous study shown a toxic impact of the peptide on brain pericytes [3]. In the current study we investigate whether IAPP deposits also in the retina, if it affects the retinal pericyte population and if retinal levels correlate with hippocampal IAPP levels.
**Methods**
Soluble and insoluble fractions of retinal and brain homogenates from AD patients (n=12) and non-demented controls (NC) (n=8) were generated by either formic acid dissolvement or ultracentrifugation/guanine hydrochloride treatment. ELISA was used to analyse levels of unmodified IAPP and total (unmodified and modified) IAPP. The retinal and hippocampal tissue were immunostained against the pericyte marker NG2 in order to analyse the number of pericytes, the vessel length and number of pericytes per vessel.
**Results**
We found reduced levels of unmodified IAPP and unaltered total IAPP in the insoluble retina fraction and reduced number of retinal pericytes in AD patients. Moreover, unmodified retinal IAPP levels as well as pericyte numbers correlated with the corresponding hippocampal variable and levels of unmodified IAPP correlated positively with number of retinal pericytes.
**Discussions**
Our studies suggest that IAPP can accumulate in the retina and that modifications of the peptide are associated with pericyte alterations in AD patients. Moreover, the accumulation of IAPP in the hippocampus correlates with IAPP levels in retina, suggesting that retina can function as a type of window for IAPP induced hippocampal changes.
**Conclusions**
Our studies demonstrate that IAPP levels are altered in the AD retina and suggest that modifications of IAPP may play a role in AD vasculopathy.

**References**
1. Jackson K, Barisone GA, Diaz E, Jin LW, DeCarli C, Despa F: Amylin deposition in the brain: A second amyloid in Alzheimer disease? Ann Neurol 2013, 74:517-526.
2. Oskarsson ME, Paulsson JF, Schultz SW, Ingelsson M, Westermark P, Westermark GT: In vivo seeding and cross-seeding of localized amyloidosis: a molecular link between type 2 diabetes and Alzheimer disease. The American journal of pathology 2015, 185:834-846.
3. Schultz N, Byman E, Fex M, Wennstrom M: Amylin alters human brain pericyte viability and NG2 expression. Journal of cerebral blood flow and metabolism : official journal of the International Society of Cerebral Blood Flow and Metabolism 2016.

## P1-C11 SPECT IMAGING AND BRAIN RETENTION OF RECOMBINANT BISPECIFIC AND UNMODIFIED ANTIBODIES IN AN AMYLOID BETA MOUSE MODEL

Tobias Gustavsson, Stina Syvänen, Dag Sehlin
Department of Public Health and Caring Sciences, Uppsala University, Uppsala, Sweden
*Molecular Neurodegeneration* 2019, **14(Suppl 1):**POSTER

**Correspondence:** Tobias Gustavsson
**Background**
Multiple immunotherapeutic agents have been evaluated as treatment in Alzheimer's disease, but with limited success. This can, at least partly, be attributed to the blood-brain barrier (BBB) which drastically reduces brain entry of large molecules such as antibodies. In this project, we have evaluated a bispecific antibody targeting Aβ protofibrils and the transferrin receptor, RmAb158-scFv8D3, in comparison with unmodified RmAb158.
**Methods**
RmAb158-scFv8D3 and RmAb158 were labeled with 125I and injected into tg-ArcSwe and WT mice, aged 18-24 months. Blood pharmacokinetics were evaluated over a period of 27 days and SPECT scans were performed at 6, 14 and 27 days. Brain was isolated following SPECT scanning and radioactivity was measured ex vivo. Autoradiography was performed on 20 μm brain sections to investigate antibody intrabrain distribution while Aβ pathology was examined with Aβ immunohistochemistry.
**Results**
RmAb158-ScFv8D3 showed a faster blood clearance compared to RmAb158. SPECT showed a higher uptake and more uniform distribution of RmAb158-scFv8D3 compared to RmAb158. Antibody brain retention, expressed as percent of injected dose (%ID), was 0.8±0.25% and 0.3% at 3 days; 0.3±0.13% and 0.15% at 14 days; and 0.12±0.03% and 0.05±0.008% at 27 days for RmAb158-scFv8D3 and RmAb158, respectively. Ex vivo autoradiography of RmAb158-scFv8D3 and RmAb158 injected mice revealed that while RmAb158-scFv8D3 was uniformly distributed throughout the brain, coinciding with Aβ pathology, RmAb158 was confined to central brain areas and a few high intensity hotspots in the brain parenchyma.
**Conclusion**
The bispecific antibody RmAb158-scFv8D3 showed higher brain concentrations than unmodified RmAb158 at all studied time points after administration demonstrating the feasibility of TfR mediated transcytosis. In addition, the global distribution pattern in the brain parenchyma was fundamentally different between the two types of antibodies; RmAb158-scFv8D3 was detected throughout the brain in line with the abundant brain Aβ pathology while RmAb158 appeared in a more scattered pattern.

## P1-C12 INCREASED LEVELS OF EXTRACELLULAR MATRIX PROTEINS IN CEREBROSPINAL FLUID FROM PATIENTS WITH ALZHEIMER'S DISEASE

Karolina Minta[1], Kaj Blennow[1,2], Henrik Zetterberg[1,2,3,4], Ulf Andreasson[1,2]
[1]Institute of Neuroscience and Physiology, Department of Psychiatry and Neurochemistry, the Sahlgrenska Academy at the University of Gothenburg, Sweden; [2]Clinical Neurochemistry Laboratory, Sahlgrenska University Hospital, Mölndal, Sweden; [3]Department of Molecular Neuroscience, UCL Institute of Neurology, London, UK; [4]UK Dementia Research Institute at UCL, London, UK
*Molecular Neurodegeneration* 2019, **14(Suppl 1):**POSTER

**Correspondence:** Karolina Minta

**Background**

Brevican, neurocan, tenascin-C and tenascin-R are extracellular matrix (ECM) proteins expressed in the brain. Brevican and neurocan belong to chondroitin sulfate proteoglycan family, the most abundant proteoglycans in CNS. Together with tenascins and hyaluronic acid they form perineuronal nets that are responsible for synaptic stabilization in the brain. They play important roles in proliferation, migration and differentiation of neurons and other cell types in the brain. They are also expressed in various pathological conditions being the major inhibitory component of glial scars. The aim of the study was to investigate if ECM protein concentrations in CSF are linked to the neurodegenerative process in Alzheimer's disease (AD).

**Methods**

Lumbar CSF samples from a non-AD control group (n=28) and a neurochemically diagnosed AD group (n=33), matched for age and gender, were analyzed using commercially available ELISAs. The AD patients had abnormal core AD CSF biomarker (Aβ42, t-tau and p-tau) levels, while controls had normal levels. Non-parametric Mann-Whitney U test was used to examine group differences, while Spearman's rho nonparametric test was used for correlations.

**Results**

Brevican, neurocan and tenascin-R levels were significantly higher in AD group compared to controls (p=0.0002, p=0.002, p=0.0017, respectively). There was no significant difference in CSF tenascin-C concentration between AD patients and controls. Brevican, neurocan and tenascin-R concentrations correlated with tau and p-tau levels (r=0.6-0.7, p<0.0001). Tenascin-C only correlated with tau (r=0.3, p= 0.04)

**Conclusions**

The study shows that increased CSF levels of brevican, neurocan and tenascin-R are associated with AD, indicating that these ECM proteins might represent novel biomarkers for AD. The correlations with tau and p-tau levels further support that these proteins are related to AD-type neurodegeneration.

## P1-C13 CEREBROSPINAL FLUID α-SYNUCLEIN LEVELS ARE ASSOCIATED WITH PIB-PET RETENTION AND ESTIMATED YEARS TO SYMPTOM ONSET IN PRECLINICAL AUTOSOMAL DOMINANT ALZHEIMER'S DISEASE

Daniel Twohig[1], Elena Rodriguez-Vieitez[2], Kalicharan Patra[1], Anne Fagan[3], Randall J. Bateman[3], John C. Morris[3], Agneta Nordberg[2], Henrietta M. Nielsen[1], for the Dominantly Inherited Alzheimer Network (DIAN)
[1]Stockholm University, Department of Neurochemistry, Stockholm, Sweden; [2]Karolinska Institutet, Department of Neurobiology, Care Sciences and Society, Stockholm, Sweden; [3]Washington University School of Medicine, Department of Neurology, St Louis, USA
*Molecular Neurodegeneration* 2019, **14(Suppl 1):**POSTER

**Correspondence:** Daniel Twohig

**Background**

Accumulating evidence of increased cerebrospinal fluid (CSF) α-synuclein (aSyn) levels and aSyn pathology in the brains of Alzheimer's disease (AD) patients suggests that aSyn is involved in AD pathogenesis1–3. To investigate whether CSF aSyn alterations occur during the preclinical phase of AD we assessed CSF aSyn levels in a cross- sectional sample from the Dominantly Inherited Alzheimer Network (DIAN) including asymptomatic and symptomatic participants carrying autosomal dominant AD gene mutations in the amyloid precursor protein (APP), presenilin-1 (PSEN1), or presenilin-2 (PSEN2) genes, and their non-mutation carrying relatives.

**Methods**

A total of n=142 participants were analyzed. Specifically, n=92 participants with autosomal dominant AD mutations including n=24 APP, n=50 PSEN1, and n=18 PSEN2 mutation carriers (MCs), along with n= 50 genetically related non- mutation carrying control participants (NCs). Quantification of CSF aSyn was performed using a commercially available sandwich enzyme-linked immunosorbent assay (ELISA) (Anaspec). A total of n=133 participants underwent 11C- Pittsburgh Compound B (PiB) positron emission tomography (PET) imaging.

**Results**

We found an increase in CSF aSyn levels in symptomatic MCs versus NCs (p=0.03), and CSF aSyn was positively correlated to the estimated years to symptom onset (EYO) (p=0.05) across all MCs. Importantly, in asymptomatic MCs higher CSF aSyn levels were related to higher PiB-PET retention in several brain areas. This relationship was inversed once the MCs had developed clinical symptoms whereby lower CSF aSyn levels were correlated to higher PiB-PET retention in selected brain regions.

**Conclusions**

Elevated CSF aSyn levels are linked to the development of dementia symptoms in autosomal dominant AD, with increasing CSF aSyn levels being positively correlated to amyloid deposition during the preclinical stages of disease. Future studies detailing the molecular links between altered CSF, brain parenchymal aSyn levels, amyloid pathology and the development of AD symptoms are needed in order to reveal the role of aSyn in the pathogenesis of AD.

## P1-C14 HIGH BLOOD GLUCOSE LEVELS WITH APOE ε3 RELATED TO WORSE COGNITIVE FUNCTION IN COMMUNITY ELDERS

Qiu Qi, Lin Xiang, Sun Lin, Min-jie Zhu, TaoWang, Jing-hua Wang, Guanjun Li, Shi-fu Xiao, Xia Li
Department of Psychogeriatrics, Shanghai Mental Health Center, Shanghai JiaoTong University School of Medicine, Shanghai, China
*Molecular Neurodegeneration* 2019, **14(Suppl 1):**POSTER

**Correspondence:** Shi-fu Xiao, Xia Li

**Background**

Few studies investigated the effects of blood glucose (BG) on cognitive function in community dwelling elderly with Apolipoprotein E (APOE) ε3. Objective To explore the effect of high BG levels in APOE ε3 carriers on cognitive function in non-demented community older adults, compared to counterparts with APOE ε4 or APOE ε2 genotype.

**Methods**

Within the China Longitudinal Ageing Study, we recruited 282 elderly adults without dementia. Data collected included demographic information, psychological measures, laboratory tests including BG and plasma lipid levles, and APOE genotypes. We divided the groups as the APOE ε2(ε2/ ε2, ε2/ ε3), ε3(ε3/ ε3), ε4(ε3/ε4, ε4/ ε4) groups. The partial correlation analyses and multivariate linear regression analyses were utilized to assess the cognitive function and laboratory data like BG and lipid data in different APOE carriers. Furthermore, white matter hyperintensity (WMH) was measured on MRI in 77 participants.

**Results**

With adjustment for age, education and sex, higher BG in non-demented communities' older adults was associated with cognitive decline on immediate memory and verbal fluency. In APOE ε3 group, elevated BG was associated with cognitive decline in immediate memory, verbal fluency, and perceptual reasoning. In APOE ε4 group, higher glucose was also correlated with visual spatial reasoning decline. A trend association was showed that higher BG had severer WMHs.

**Conclusion**

Higher BG was correlated with different cognitive domains decline in APOE ε3 carriers in non-dementia older adults.

## P1-C15 NEUROFILAMEN CHANGES IN SERUM AND CEREBROSPINAL FLUID AFTER ACUTE ISCHEMIC STROKE

Fani Pujol-Calderón[1], Erik Portelius[1,2], Henrik Zetterberg[1,2,3,4], Kaj Blennow[1,2], Lars E. Rosengren[5], Kina Höglund[1,2]
[1] Department of Psychiatry & Neurochemistry, University of Gothenburg, Sweden; [2] Clinical Neurochemistry Laboratory, Sahlgrenska University Hospital, Mölndal, Sweden; [3] Department of Molecular Neuroscience, UCL Institute of Neurology, Queen Square, London, UK; [4] UK Dementia Research Institute at UCL, London, UK; [5] Department of Neurology, University of Gothenburg, Sweden
*Molecular Neurodegeneration* 2019, **14(Suppl 1):**POSTER

**Correspondence:** Fani Pujol-Calderón

**Background**

Neurofilament light (NFL) is a well-validated biomarker for neuronal injury and neurodegeneration. It shows increased cerebrospinal fluid (CSF) levels after stroke [1], as well as in patients with a broad range of neurodegenerative and neuroinflammatory diseases [2]. Neurofilament heavy, (NFH) belongs to the same family of structural proteins but it is less extensively studied [3]. The role of phosphorylated NFH (pNFH) as a marker and in disease progression is unknown. In this pilot study, we aimed to examine the temporal pattern evolution of NFL and pNFH concentrations in serum and CSF after acute ischemic stroke.

**Methods**

A quantitative Enzyme Linked ImmunoSorbent Assay (ELISA) for pNFH was developed and tested on CSF and serum samples. NFL and pNFH were analysed in serum and CSF of acute ischemic stroke patients, who were followed over time (Day 0-1, Day 2-3, Day 7-9, 3 weeks, 3-5 months after stroke).

**Results**

NFL and pNFH concentrations in serum and CSF increased after stroke, peaked during the 3rd week, and then decreased back to almost baseline levels after 3-5 months. CSF-NFL and serum-NFL correlated to the outcome measured by Barthel Index after 3-5 months, whilst no such association was seen for pNFH.

**Discussion**

These findings suggest that NFL and pNFH both in CSF and serum reflect the temporal pattern of the post ischemic Wallerian degeneration of axons and that this process does not seem to progress after 3-5 months.

**Conclusion**

Further studies in larger populations are needed to fully understand the progression of the neuronal damage after acute ischemic stroke and to evaluate if these biomarkers can provide additive information and how they relate to outcome.

## P1-D01 NEW INSIGHTS INTO THE AGGREGATION OF α-SYNUCLEIN AT LOW CONCENTRATIONS

Kseniia Afitska [1], Anna Fucikova [2], Volodymyr V. Shvadchak [1], Dmytro A. Yushchenko [1]
[1] Institute of Organic Chemistry and Biochemistry, Academy of Sciences of the Czech Republic, Prague, Czech Republic; [2] Department of Chemical Physics and Optics, Faculty of Mathematics and Physics, Charles University, Prague, Czech Republic
*Molecular Neurodegeneration* 2019, **14(Suppl 1):**POSTER

**Correspondence:** Kseniia Afitska

**Background**

α-Synuclein is an intrinsically disordered protein that is expressed in neurons. Misfolding of αSyn in amyloid fibrils is one of hallmarks of Parkinson's disease, the second most common neurodegenerative disorder [1]. Although there were several attempts to find critical concentration of α- synuclein fibrillization [2,3], aggregation of α-synuclein at low concentrations has not been characterized and fibril dissociation constant Kd has not been determined.

**Methods**

We studied kinetics of α-synuclein aggregation at protein concentrations in the range 0.11-20 μM. Aggregation was monitored using CD-spectroscopy and Thioflavine T (ThT), a dye which increases its fluorescence upon binding to β-sheet-rich protein aggregates. The formed aggregates were characterized by atomic force microscopy (AFM).

**Results**

The samples of α-synuclein with concentrations 5-20 μM demonstrated typical first-order aggregation kinetics according to ThT and formation of fibrils visualized by AFM. The samples of 0.4-5 μM α-synuclein showed atypical kinetics with saturation before complete monomer depletion. Although according to CD spectra the β-sheet content in these samples increased during the aggregation, the structure of the formed aggregates was different from amyloid fibrils that was shown by AFM microscopy. The samples of 0.11-0.4 μM α-synuclein did not aggregate. From the kinetic curves we calculated Kd of α-synuclein fibrils and found that it is in the range of 0.4 μM.

**Discussion**

Our data suggest that the typical α-synuclein amyloid fibrils are formed at the protein concentrations higher than 5 μM, whereas at low μM concentrations α- synuclein forms β-sheet-rich aggregates. Similar structures were also observed at the initial stages of aggregation of 45 μM α-synuclein [4]. Calculated Kd of α-synuclein fibrils is similar to Kd of another amyloid protein Aβ [5].

**Conclusions**

We show that aggregation of α-synuclein occurs at much lower concentrations than it was reported earlier. Also for the first time we calculated Kd of α-synuclein fibrils.

properties of aggregates during amyloid fibrillization. Angew Chem Int Ed Engl 2015, 54: 2462- 2466.
5. O'Nuallain, B., Shivaprasad, S., Kheterpal, I., and Wetzel, R. Thermodynamics of A beta(1-40) amyloid fibril elongation. Biochemistry 2005, 44: 12709-12718.

## P1-D02 ACCUMULATION OF AGGREGATED ALPHA-SYNUCLEIN IN HUMAN ASTROCYTES AFFECTS THE AUTOPHAGOSOMAL PATHWAY

Jinar Rostami[1], Staffan Holmqvist[2], Martin Ingelsson[1], Joakim Bergström[1], Laurent Roybon[2], Anna Erlandsson[1]
[1]Department of Public Health and Caring Science, Uppsala University, Sweden; [2]Department of Experimental Medical Science, Lund University, Sweden
*Molecular Neurodegeneration* 2019, **14(Suppl 1):**POSTER

**Correspondence:** Jinar Rostami

**Background**

Parkinson's disease (PD) is characterized by intracellular neuronal inclusions called Lewy bodies, which are mainly composed of aggregated alpha-synuclein [1,2]. In addition to the neuronal pathology, alpha-synuclein inclusions also appear frequently in astrocytes. Being the most abundant glial cell type in the brain, astrocytes have great impact on tissue homeostasis [3], but their role in PD remains elusive. The aim of this study was to clarify the effect of alpha-synuclein aggregates on the autophagosomal pathway in astrocytes.

**Methods**

Human astrocytes were treated with Cy3-labelled alpha-synuclein aggregates for 24 h, after which the cells were thoroughly washed and incubated for additional 0, 3 or 6 days in alpha-synuclein free medium.

**Results and discussion**

The astrocytes engulfed large amounts of alpha-synuclein aggregates, which were stored in the trans-Golgi network region rather than degraded resulting in mitochondrial damage and ER swelling. Immunostainings for the autophagosomal marker, LC3BII, suggested increased autophagosome formation at the earliest time points after alpha-synuclein exposure, but declined to control levels at the latest time point. To study the fusion and turnover of autophagosomes to autolysosomes, LC3BII/I ratios were measured by Western blot analysis. The alpha-synuclein exposed cells displayed significantly higher levels of LC3BII/I, indicating that the alpha-synuclein accumulation affects the autophagosome turnover. This result was further verified using a transfection method including the LC3B-RFP-GFP gene where the GFP protein is sensitive to low pH resulting in only RFP appearance. Accordingly, 3 days following alpha-synuclein exposure, most cells displayed RFP labeled vesicles whereas at day 6 following alpha-synuclein exposure, most cells had RFP/GFP labeled vesicles, suggesting altered autophagosome-lysosome fusion.

**Conclusion**

In summary, our results demonstrate that accumulation of alpha-synuclein aggregates in human astrocytes affect the autophagosomal pathway, which may influence the supporting function of astrocytes to neurons, consequently leading to exacerbated neuronal damage.

## P1-D03 IN SITU PROXIMITY LIGATION ASSAY REVEALS CO-LOCALIZATION OF ALPHA-SYNUCLEIN AND SNARE PROTEINS IN MURINE PRIMARY NEURONS

Emma Persson, Leire Almandoz-Gil,Veronica Lindström, Martin Ingelsson, Anna Erlandsson, Joakim Bergström
Department of Public Health and Caring Sciences / Molecular Geriatrics, Uppsala University, Sweden
*Molecular Neurodegeneration* 2019, **14(Suppl 1):**POSTER

**Correspondence:** Emma Persson

**Background**

The aggregation of alpha-synuclein (αSyn) is the pathological hallmark of Parkinson's disease. However, the physiological function of the protein and how it relates to its pathological effects remain poorly understood. One of the proposed roles of αSyn is to promote the soluble N- ethylmaleimide-sensitive factor activating protein receptor (SNARE) complex assembly by binding to VAMP-2 [1]. The objective of this study was to visualize the co-localization between αSyn and the SNARE proteins (VAMP-2, SNAP-25 and syntaxin- 1) using in situ proximity ligation assay (PLA) [2].

**Methods**

Cortical primary neurons were cultured from E14 non-transgenic or transgenic mice expressing human A30P αSyn. The neurons were analyzed with sandwich ELISA, immunofluorescence and PLA.

**Results**

With an αSyn antibody, a PLA signal indicating close proximity between αSyn and the three SNARE proteins was observed both in the soma and throughout the processes. No differences in the extent of PLA signals were seen between non-transgenic and transgenic neurons. ELISA analysis detected 600 pM of human αSyn in A30P neurons but no difference in total levels of αSyn. Immunofluorescence images indicated 13 % of A30P neurons to be human αSyn positive. With an antibody specific against human αSyn, the PLA signal was seen to a lesser degree, mostly located to the soma.

**Discussion**

PLA have previously been used to study interactions between αSyn and other synaptic proteins [3- 6]. This is the first time co-localization have been visualized between αSyn and the SNARE proteins using in situ PLA in primary neurons. The PLA puncta were abundant in the processes which could indicate localization in the synaptic boutons.

**Conclusions**

In situ PLA is a method that can be used to investigate the co-localization of αSyn and the SNARE proteins in primary neuronal cultures and could potentially uncover pathological changes in protein levels and/or distribution.

P1-D04 ORGANOTYPIC HIPPOCAMPAL SLICE CULTURES IN MODELLING α-SYNUCLEINOPATHIES

**Correspondence:** Melanie Barth

**Background**

Synucleinopathies including Parkinson's disease (PD) or Lewy body dementia (LBD) are neurodegenerative diseases characterised by the abnormal accumulation of α- synuclein (αS) in the perikarya or processes of neurons, the so-called Lewy bodies (LB) and Lewy neurites (LN), respectively. It is not clear how these αS aggregates are formed and propagate within the brain of diseased individuals but accumulating evidence suggests a prion-like mechanism [1]. In line with this, several in vitro and in vivo studies that use insoluble αS from human brains with PD or LBD, synthetic pre-formed fibrils (αS pff) or transgenic mouse brain homogenate as seeds have reported the induction of αS pathology in wildtype (wt) as well as transgenic (tg) experimental models [2,3].

**Methods**

Here we aimed to establish a hippocampal slice culture (HSC) model to study prion-like aggregation and propagation of αS inclusions.

**Results**

We were able to induce LB- and LN-like αS inclusions in HSC from tg as well as wt mice by only one-time treatment with αS seeds (either brain-derived or pff) in a time- and concentration-dependent manner.

**Conclusion**

This work illustrates that HSC have the potential to become a potential promising tool to study αS lesions and gain further insights into its seeding and spreading patterns.

P1-D05 AUXILIN PROTECTS AGAINST ALPHA-SYNUCLEIN AGGREGATION, CELL DEATH AND IMPAIRMENT OF ENDOCYTOSIS

**Correspondence:** Christian Hansen

**Background**

A hallmark of Parkinson's disease (PD) is the formation of Lewy bodies (LB's) in neurons. LB's are cytoplasmic protein rich aggregates, in which α-synuclein (α- syn) is the most abundant protein. Recently, recessive mutations were discovered in the auxilin (DNAJC6) encoding gene, that were linked to a juvenile form of PD. Auxilin is a DNAJ co-chaperone of the HSP70 chaperones, which are important for protein folding and homeostasis in the cell.

**Methods**

To address our questions, we have used HEK293 and N2a cell culture, KO of auxilin by Crispr/CAS9 method, produced recombinant proteins and performed α-syn based thioflavin T assays of α-syn aggregation. Endocytosis has been measured using fluorescently labeled transferrin and cell death using tryphan blue.

**Results**

We observed that auxilin KO cells, which overexpressed α-syn-Dsred, contained more α-syn aggregates than did the parental control cells. Moreover, we observed an increased cell death in KO cells that overexpressed α-syn-Dsred. The increased cell death in α-syn- Dsred Auxillin KO cells could be prevented by re-introducing Auxilin into these cells. With use of recombinant proteins, we found that auxilin inhibits α-syn aggregation as well in vitro. As both α-syn and auxilin, are important for endocytosis and exocytosis in neurons, we explored how these dynamics were affected in HEK293T cells. We observed that endocytosis was impaired in cells that had KO of auxilin and overexpressed α- syn-Dsred, but not in parental control cells.**Discussion**

Our results suggest, that increased aggregation of α-syn-Dsred can impair endocytosis in the absence of auxilin which ultimately affects cell survival.

**Conclusions**

These results links a cellular role of auxilin in preventing vulnerability to α-syn aggregates, and this provides a possible explanation for how recessive mutations in the auxillin gene could be linked to PD.

P1-D06 CELL AND ANIMAL MODELS WITH MUTATION IN THE SMALL GTPase RAB39B SHOW KEY PATHOLOGICAL FEATURES OF PARKINSON'S DISEASE

Y Gao[1,2], GR Wilson[1,2], SEM Stephenson[1,2], K Bozaoglu[1,2], M Dottori[3], PQ Thomas[4], PJ Lockhart[1,2]
[1]Bruce Lefroy Centre for Genetic Health Research, Murdoch Children's Research Institute, Melbourne, Australia; [2]Department of Paediatrics, University of Melbourne, Melbourne, Australia; [3]Illawarra Health and Medical Research Institute, Centre for Molecular and Medical Bioscience, University of Wollongong, New South Wales, Australia; [4]School of Biological Sciences, The University of Adelaide, Adelaide, Australia
*Molecular Neurodegeneration* 2019, **14(Suppl 1):**POSTER

**Correspondence:** Y Gao

**Background**

Parkinson's disease (PD) is a neurodegenerative disorder characterized by loss of dopaminergic neurons in the substantia nigra and presence of α-synuclein (αSN) aggregates. We previously demonstrated loss of function mutations in Ras Analog in Brain 39B (RAB39B) cause early onset PD, with dysregulation of αSN observed in vitro and in vivo [1]. Our aim is to investigate the role of RAB39B in PD pathology.

**Methods**

We utilised CRISPR/CAS9 genome editing to generate isogenic pluripotent stem cell (PSC) lines with deletion/knockout of RAB39B, in addition to PSC from RAB39B deletion patient fibroblasts. We also utilised CRISPR/CAS9 to generate knockout of Rab39b in C57BL/6 mouse.

**Results**

For the stem cell models, we successfully differentiated cortical and dopaminergic neurons from the isogenic and patient stem cell lines. By western blot analysis, we observed a significant increase in steady-state levels of αSN in the mature RAB39B knockout neurons compared to controls. For the mouse models, behavioral analysis of a knockout cohort at three time points (11, 18 and 22 months) identified motor deficits. On the balance beam, knockout mice performed significantly worse than littermate controls, slipping more than five times as often at 22 months (Wildtype 2.3±1.0, n=6; Knockout 12.8±

3.3, n=6, p=0.006). Similarly, there was a significant deficit recorded by accelerating rotarod at 22 months (Wildtype 169.2±17.6, n=6; Knockout 83.2±21.4, n=6, p=0.010).

**Discussion**

Disease models with knockout of RAB39B show key pathological features of PD, including increased aSN levels and deficits in fine motor control. This suggests shared underlying pathological mechanisms between RAB39B- mediated PD and other forms of PD.

**Conclusions**

We have generated unique models that recapitulate aspects of the human disease; these will be useful tools to determine the neuropathological mechanisms underlying RAB39B-mediated PD, its role in the regulation of aSN homeostasis, and the therapeutic potential of RAB39B.

## P1-D07 CHARACTERIZATION OF ALPHA-SYNUCLEIN PATHOLOGY PROGRESSION IN h[A30P]-ALPHA-SYNUCLEIN TRANSGENIC MICE FOLLOWING INTRAMUSCULAR INJECTION OF SONICATED PRE-FORMED ALPHA-SYNUCLEIN FIBRILS

Fredrik Eriksson[1], Adeline Rachalski[1], Wen Li[2], Jessica Sigvardsson[1], Christer Möller[1], Jia Yi Li[2], Eva Nordström[1]

[1]BioArctic AB, Stockholm, Sweden; [2]Department of Experimental Medical Science, Lund University, Lund, Sweden

*Molecular Neurodegeneration* 2019, **14(Suppl 1):**POSTER

**Correspondence:** Fredrik Eriksson

**Background**

Onset of alpha-synuclein pathology and motor phenotype following intramuscular injection of sonicated pre-formed alpha-synuclein fibrils (PFF) in A53T and WT alpha-synuclein transgenic mice was recently published [1] and later replicated in A30P transgenic mice. The aim of this study was to further characterize the model with regards to progression of alpha-synuclein pathology in h[A30P] alpha- synuclein transgenic mice.

**Methods**

8-10 weeks old h[A30P]alphaSYN tg mice were unilaterally injected in the right gastrocnemius muscle with 1 μg of PFF. At pre-defined time-points post injection and at terminal disease, mice were sacrificed and tissues collected and evaluated for alpha-synuclein pathology.

**Results**

A time dependent progression of alpha-synuclein pathology was observed. At 1 week post injection all mice exhibited pSer129-alpha-synuclein positive neurites in the lumbar sections of the spinal cord which progressed to the thoracal and cervical parts at 2 weeks post injection. Terminal animals, i.e. mice exhibiting severe motor deficits at time of sacrifice, showed pathology throughout the CNS including brain. The progression of pathology co-incided with an increase of neurofilament light chain (NfL) in both CSF and plasma, suggesting that NfL can serve as a marker of disease progression in this model.

**Discussion**

As described for the A53T transgenic mice, intramuscular PFF injection results in a synchronized onset of alpha-synuclein pathology also in A30P mice. The approach will significantly improve the use of this otherwise highly heterogenic mouse strain in pre-clinical evaluation of alpha- synuclein targeted therapies.

**Conclusions**

Intramuscular injection of PFF results in a time dependent progression of alpha-synuclein pathology in A30P transgenic mice.

## P1-E01 GENETIC ANALYSIS OF THE GENES ASSOCIATED WITH ALZHEIMER'S DISEASE AND FRONTOTEMPORAL LOBAR DEGENERATION IN EARLY-ONSET DEMENTIA PATIENTS

Laura Luukkainen[1], Johanna Krüger[1,2], Seppo Helisalmi[3], Laura Kytövuori[1,2], Riitta Ahmasalo[4], Eino Solje[3,5], Mikko Hiltunen[6], Anne M Remes[1,2]

[1]Research Unit of Clinical Neuroscience, Neurology, University of Oulu, Oulu, Finland; [2]Medical Research Center, Oulu University Hospital, Oulu, Finland; [3]Institute of Clinical Medicine - Neurology, University of Eastern Finland, Kuopio, Finland; [4]Neurology, Lapland Central Hospital, Rovaniemi, Finland; [5]Neuro Center, Kuopio University Hospital, Kuopio, Finland; [6]Institute of Biomedicine, University of Eastern Finland, Kuopio, Finland

*Molecular Neurodegeneration* 2019, **14(Suppl 1):**POSTER

**Correspondence:** Laura Luukkainen

**Background**

Amyloid precursor protein (APP), presenilin-1 (PSEN1) and presenilin-2 (PSEN2) mutations account for under 10% of early-onset Alzheimer´s disease (EOAD) cases [1]. To date, over 280 mutations in these genes have been identified [1]. Mutations in C9orf72, microtubule associated protein tau (MAPT) and progranulin (GRN) genes account for 60% of inherited frontotemporal lobar degeneration (FTLD) cases [2]. The aim of this study was to evaluate the contributions of the mutations associated with AD and FTLD in a cohort of early-onset dementia (EOD).

**Methods**

The study population consisted of 39 patients (mean age of onset 54.8±6.3 years) with EOD diagnosed at two memory outpatient clinics in Finland. The patients had early-onset disease and one or two dementia patients in family or atypical or rapidly progressive clinical picture. The patients carrying the APOE ε4 allele or the C9ORF72 expansion were excluded. Mutations were identified by NGS- based exome sequencing and confirmed by Sanger sequencing.

**Results**

We identified two pathogenic mutations; PSEN1 p.His163Arg and MAPT p.Arg406Trp. The patient with PSEN1 p.His163Arg had rapidly evolving amnesia, clumsiness, myoclonic jerks and upper limb tremor. Later she became euphoric and got facial dyskinesia and vocal tic. The patient with MAPT p.Arg406Trp mutation had familial EOD with mood symptoms that evolved to psychosis. No pathogenic APP or GRN mutations were identified.

**Discussion**

The phenotype of the patients carrying PSEN1 p.His163Arg mutation has been recognized to include aphasia, apraxia, amnestic and depression symptoms, visual hallucinations, rigid-bradykinetic syndrome and multifocal myoclonus [3-5]. Progression of euphoria and vocal tic might also be part of the neurodegenerative process. MAPT p.Arg406Trp mutation has been reported to cause both EOAD and FTD [6]. Rapidly progressing disorder with psychosis has been reported in a Japanese patient [7].

**Conclusions**

Autosomal dominant mutations MAPT p.Arg406Trp and PSEN1 p.His163Arg were identified in a Finnish EOD cohort.

## P1-E04 EMPLOYING GENETIC CODE EXPANSION TO STUDY PROCESSING OF AMYLOID PRECURSOR PROTEIN

Lea S. van Husen[1], Simon J. Elsässer[2], Thomas P. Sakmar[1,3], Manija A. Kazmi[3], Bengt Winblad[1], Sophia Schedin-Weiss[1] , Lars O. Tjernberg[1]
[1] Department NVS, Center for Alzheimer Research, Division of Neurogeriatrics, Karolinska Institutet, 141 57, Huddinge, Sweden; [2] Science for Life Laboratory, Department of Medical Biochemistry and Biophysics, Karolinska Institutet, Stockholm, Sweden; [3] Laboratory of Chemical Biology and Signal Transduction, The Rockefeller University, 1230 York Avenue, New York, NY 10065, USA
*Molecular Neurodegeneration* 2019, **14(Suppl 1):**POSTER

**Correspondence:** Lea S. van Husen

**Background**

The amyloid precursor protein (APP) is critical in the pathophysiology of Alzheimer disease (AD), since two- step proteolytic processing of APP generates the neurotoxic amyloid β- peptide (Aβ). Trafficking and processing of APP has been widely studied [1][2] but a major challenge is the labeling of Aβ in living cells. To solve this problem, we used a genetic code expansion approach by inserting an amber stop codon at specific positions in APP to enable incorporation of an unnatural amino acid (UAA) that can be fluorescently tagged [3].

**Methods**

We individually mutated ten different codons to amber codons in the Aβ region and added a SNAP-tag at the C-terminus of APP. HEK293T cells were transfected with plasmids containing one modified APP and an amber codon suppression system (the orthogonal Pyrrolysyl tRNA synthetase/ tRNA pair) and grown in the presence of a lysine- derived UAA. We validated the processing of the APP variants with ELISA and immuno-blotting. The UAA was fluorescently labeled with silicon rhodamine-tetrazine and the SNAP-tag with TMR-Star, followed by analysis using confocal microscopy.

**Results**

We were able to incorporate different lysine- derivatives into all mutated APP variants using amber codon suppression. We identified several APP mutants that showed similar cleavage efficiency as wild-type APP. Moreover, we showed that dual labeling of the UAA and the SNAPtag of APP is possible.

**Discussion**

Our data show that the incorporation of the lysine-derivatives lead to minimal changes in APP processing. Thus, amber codon suppression is a suitable method to study APP cell biology and biochemistry. Dual labeling further enables us to monitor Aβ and the C-terminal cleavage products simultaneously.

**Conclusions**

This method provides a powerful tool to study the processing of APP with minimal changes in the protein itself. Understanding the pathway of APP processing may enable novel strategies for treatment of AD.

## P1-E05 GABA CATABOLISM INVOLVEMENT IN ALZHEIMER'S DISEASE: A CASE-CONTROL STUDY OF SNPs INALDH5A1 GENE

Giovanna Menduti [1], Luisa Benussi[2], Roberta Ghidoni[2], Rosanna Squitti[2], Mariacristina Siotto[3], Giuliano Binetti[2,4], Concetta Capo[1], Bianca M. Ciminelli[1], Patrizia Malaspina[1], Luisa Rossi[1]
[1]Department of Biology, University of Rome Tor Vergata,Rome, Italy; [2]Molecular Markers Laboratory, IRCCS Istituto Centro San Giovanni di Dio-Fatebenefratelli, Brescia, Italy; [3]Don Carlo Gnocchi ONLUS Foundation, Milan, Italy [4]MAC Centro per la Memoria, IRCCS Istituto Centro San Giovanni di Dio - Fatebenefratelli
*Molecular Neurodegeneration* 2019, **14(Suppl 1):**POSTER

**Correspondence:** Giovanna Menduti

Alzheimer's disease (AD) etiology involves polygenic and multifactorial causes, along with environmental risk factors. An interesting relationship between the catabolism of GABA and AD progression was suggested [1,2]. Succinic semialdehyde dehydrogenase (SSADH) is the enzyme responsible for the conversion of succinic semialdehyde (SSA), a GABA catabolite. Moreover, SSA can be directed to a minor route, generating gammahydroxybutyrate (GHB). Different missense mutations of SSADH gene (ALDH5A1), leading to enzyme failure, cause a rare metabolic disorder known as SSADH deficiency. Furthermore, common polymorphisms have been identified which affect SSADH activity when in vitro expressed [3,4]. A link between SSADH and cognitive impairment may be envisaged, since SSADH polymorphism c.538C>T was found to affect survival and cognitive performance in the elderly [5] and an increase of a GHB catabolite was reported in AD patients [6]. Therefore, the present study was aimed to search a possible association between ALDH5A1 SNPs and AD. We performed a case- control study by genotyping a population of 300 AD patients and 300 matched controls for c.538C>T and c.545C>T SNPs of the ALDH5A1 gene by TaqMan assays. DNA samples were obtained by the Biobanca of IRCCS Fatebenefratelli (Brescia). AD patients have been selected according to NINCDS–ADRDA criteria and MMSE ≤25. All individuals (cases and controls) have been genotyped for the APOE ε2, ε3 and ε4 polymorphism.

Our results show that the T allele of the c.538C>T ALDH5A1 SNP is more frequent in samples with AD than in controls. Furthermore, when considering both SNPs, we identified one haplotype with higher frequency in AD patients than in controls.

This case-control study suggests that ALDH5A1 SNPs may be related to AD onset/progression, reinforcing the hypothesis that GABA catabolism alterations might be involved in AD.

P1-E06 MUTATION ANALYSES OF DISEASE CAUSING GENES IN PATIENTS WITH EARLY ONSET FORMS OF NEURODEGENERATIVE DISORDERS

**Correspondence:** María Pagnon

**Background**

Most forms of dementia have a clear genetic background and several disease genes have been identified. Mutations in the tau gene lead to frontotemporal dementia, whereas mutations in the genes for amyloid precursor protein (APP) and the presenilins (PSEN1, PSEN2) cause early- onset, dominantly inherited forms of Alzheimer's disease (AD). In Parkinson's disease (PD) several genes resulting in autosomal dominant (α-synuclein and LRRK2) or autosomal recessive disease (Parkin, PINK1, DJ1, and ATP13A2) have been described.

**Methods**

DNA samples from 79 patients with early onset forms of AD, frontotemporal dementia, dementia with Lewy bodies or PD were analyzed during two rounds of analyses. Genes with known pathogenic mutations causing familial early onset disease as well as genes associated with disease in large genome wide association studies were included. All coding exons of selected genes were amplified using sequence enrichment technology, followed by high throughput sequencing.

**Results**

We have identified three AD patients carrying known disease causing mutations in the PSEN1 gene, leading to Pro264Leu and Met146Val amino acid substitution. We also discovered a new APP mutation in two siblings, suffering from an aggressive early onset form of Alzheimer's disease. The presence of this mutation has been mapped in the extensive family and we have excluded the possibility that it is a rare polymorphism by screening 500 other subjects with and without AD. Furthermore, in several of the PD patients we have found mutations in the PINK1 and GBA genes, which might be related to the disease development.

**Conclusions**

Even though familial forms of dementia are uncommon, it is important to identify disease-causing mutations. Our findings have helped us to confirm the clinical diagnoses. We also hope that the identification of new pathogenic mutations will enable us to better understand the underlying disease mechanisms.

P1-E07 ASSOCIATION OF POLYMORPHISM rs934945 GENE PER2 WITH SLEEP DISORDER IN THE MALE POPULATION IN RUSSIA/SIBERIA

**Correspondence:** Eldar Krymov

**Background**

We aimed to study the prevalence and associations of gene PER2 with sleep disorders in the male population aged 25-44 years in Russia/Siberia (Novosibirsk)

**Methods**

**Results**

The most common genotype of rs934945 PER2 gene was genotype G/G - 65,36%. A/G was in 30.17% and genotype A/A was in 4,47% of men. Those carriers with the A/A genotype has a increasing tendency of anxious dreams during sleep compared to carriers of other genotypes. Carriers of genotype A/A often wake up during the night. Sleep was the strongest in men with A/G and G/G genotype. Lack of sleep (5 hours or less) is also more common in persons who had genotype with homozygous allele A was presented in.

**Conclusions**

The gene PER2 (rs934945) in our population has a relationship with various chronotype that plays an important role in understanding the basics of sleep disorders.

P1-E09 SYSTEMS GENETICS IDENTIFIES MODIFIERS OF ALZHEIMER'S DISEASE RISK AND RESILIENCE

**Correspondence:** Sarah M. Neuner, Catherine C. Kaczorowski

**Background**

Recent studies of familial Alzheimer's disease (AD) cases suggest genetic modifiers may delay the onset and progression of AD symptoms by decades. Although modifiers promoting resilience may provide key targets for the prevention of AD, they remain largely unidentified in human populations. To address this significant need, our lab recently developed a novel panel of genetically diverse AD mice.

**Methods**

Female B6-5XFAD mice were bred to males from the BXD genetic reference panel. F1 offspring were phenotyped across the lifespan. Genetic interval mapping was utilized to identify areas of the genome containing variants that modify the observed variation in AD-relevant phenotypes.

**Results**

We have found that genetic background has a profound effect on the expressivity of the 5XFAD transgene. We identified multiple genomic regions associated with resilience or susceptibility to AD, including the APOE locus and two novel loci. We validated positional candidate Trpc3 as a modifier of AD symptoms with translational relevance in human populations.

**Discussion**

Our results demonstrate the incorporation of genetic diversity into animal models of AD is a critical step toward identifying translationally relevant mechanisms underlying disease. As each BXD line is fully inbred, each F1 AD-BXD line studied here can be replicated across time and laboratories, maximizing rigor and reproducibility. This approach is of broad interest to the neurodegenerative field, as it can be used with a variety of transgenic models to identify common and unique mechanisms across diseases.

### Conclusions
Here we use a variety of techniques to both confirm known genetic associations with AD and identify novel variants (e.g. Trpc3) that may play a role in disease onset and progression. Overall, results here significantly advance our understanding of how individual genetic variation provides protection to AD, and demonstrate the utility of our panel as an important resource for the study of AD.

## P1-E10 NOVEL ARPP21 MUTATIONS ARE IDENTIFIED IN AMYOTROPHIC LATERAL SCLEROSIS (ALS) PATIENTS

Chun Hao Wong[1], Simon D Topp [1], Youn-Bok Lee[1], Sarah Mueller[1], Graham Cocks[1], Bradley N Smith[1], Nicola Ticozzi[2], John Landers[3], Christopher E Shaw[1]
[1]Maurice Wohl Clinical Neuroscience Institute, Institute of Psychiatry, Psychology and Neuroscience, King's College London, UK; [2]Department of Neurology, IRCCS Instituto Auxologico Italiano, Milan, Italy; [3]Department of Neurology, University of Massachusetts Medical School, Worcester, USA
*Molecular Neurodegeneration* 2019, **14(Suppl 1):**POSTER

**Correspondence:** Chun Hao Wong

### Background
The evolution of next generation sequencing has led to exponential growth in our understanding of ALS genetics. Here, we report a new candidate gene ARPP21 identified through whole-exome sequencing in a cohort of familial ALS cases. Two novel variants absent in controls are shared by several unrelated index cases for which currently known genes have yet to be accounted for. To understand the contribution of ARPP21 in ALS, we have screened the gene extensively and modelled the identified variants in cellular models.

### Methods
Direct sequencing was performed on over 2000 ALS cases and 1000 controls of UK, US and Italian origin. Disease modelling was performed in HEK239T, SH-SY5Y and primary rat cortical neurons.

### Results
Both variants linked with familial ALS (p.P563L; p.P747L) are identified in a replication sporadic ALS cohort. The same p.Proline563 is found to be substituted to glutamine in two sporadic cases of UK and Italian origin. Similar to other ALS causative genes, most of the mutations cluster in the C-terminus disordered region of low complexity. ARPP21 encodes a predominantly cytoplasmic phosphoprotein and is highly enriched in the neuronal population. Cellular modelling of the ALS-associated mutants showed RNA-dependent detergent insoluble aggregates, proteasome dysregulation, enhanced cytotoxicity and altered neuronal morphology. A proportion of these mutants showed abnormal cytoplasmic TDP43 granules, consistent with the TDP43 proteinopathy observed in most ALS post-mortem tissues.

### Discussion
Mutation frequencies of ARPP21 in current study are approximately 1.6% in familial cases and 0.3% in sporadic cases. Cellular studies of ARPP21 mutants have recapitulated pathological hallmarks of ALS.

### Conclusions
By combining the genetics and follow-up functional assays, we have identified ARPP21 as a novel ALS candidate gene that has not been described in neurodegeneration

## P1-E11 A NOVEL ANGIOTENSIN CONVERTING ENZYME 1 MUTATION IS ASSOCIATED WITH INCREASED RISK FOR ALZHEIMER'S DISEASE AND REDUCES CELL SURVIVAL

Leah K Cuddy[1], Rudolph E Tanzi[2], Robert Vassar[1]
[1]Department of Cell and Molecular Biology, Feinberg School of Medicine, Northwestern University, Chicago, IL, USA; [2]MassGeneral Institute for Neurodegenerative disease, Charlestown, MA, USA
*Molecular Neurodegeneration* 2019, **14(Suppl 1):**POSTER

**Correspondence:** Leah K Cuddy

### Background
The Cure Alzheimer's Fund Genome Project identified a novel, highly penetrant mutation in the angiotensin converting enzyme 1 (ACE1) gene that is associated with increased risk for Alzheimer's disease (AD). ACE1 is best known for its role in blood pressure control. Mutant ACE1 could cause AD pathogenesis by raising blood pressure, since midlife hypertension has been associated AD[1]. However, ACE1 is expressed in all tissues including brain and can cleave many substrates[2]. Therefore, any of the myriad of ACE1 functions in the brain or periphery could have a role in AD pathogenesis. The goal of this study is to determine how this mutation increases the risk of AD.

### Methods
The role of mutant ACE1 in AD was investigated in cultured forebrain neurons from wild-type (WT) and knock-in (KI) mice and in human SH-SY5Y cells stably expressing either WT ACE1 or mutant ACE1 and in vivo in aged cohorts of WT and KI mice. To determine the effect of ACE1 KI on amyloid pathology, rAAV1- BRI2-Aβ-42 and control rAAV1- BRI2-ΔKR were stereotaxically injected into the brains of WT or KI mice and ACE1 KI crosses with 5XFAD amyloid mice were analyzed.

### Results
Blood pressure is unchanged in KI mice, but KI mice show higher ACE1 protein levels in cortical brain regions and in cultured forebrain neurons compared to WT mice. Unexpectedly, mutant ACE1 reduces cell survival in SH-SY5Y cells, cultured mouse forebrain neurons and in mouse brains. Neurodegeneration observed in KI mice is accelerated by Aβ. Increased cell death is related to a toxic gain-of-function of mutant ACE1.

### Discussion
These studies will provide novel information about the physiological function ACE1 in the brain, and how altered ACE1 function may cause AD.

### Conclusions
Mutant ACE1 increases the risk of AD at least in part by increasing neuronal cell death.

### References
1. Kivipelto M, Laakso MP, Tuomilehto J, Nissinen A, Soininen H: Hypertension and hypercholesterolaemia as risk factors for Alzheimer's disease: potential for pharmacological intervention. CNS drugs 2002, 16:435-444.
2. Bernstein KE, Ong FS, Blackwell WL, Shah KH, Giani JF, Gonzalez-Villalobos RA, Shen XZ, Fuchs S, Touyz RM: A modern understanding of the traditional and nontraditional biological functions of angiotensin-converting enzyme. Pharmacological reviews 2013, 65:1-46.

## P1-E13 A HEXAMERIC REPEAT EXPANSION WITHIN THE SVA RETROTRANSPOSON INSERTION IN THE TAF1 GENE INFLUENCES DISEASE EXPRESSIVITY OF X-LINKED DYSTONIA- PARKINSONISM

Charles Jourdan Reyes[1], Ana Westenberger[1], Norbert Brüggemann[1,2], Valerija Dobricic[3], Aloysius Domingo[4,5], Gerard Saranza[6], Henrike Hanßen[1,2], Karen Grütz[1], Kimberly Begemann[1], Aleksandar Rakovic[1], Roland Dominic Jamora[6], Raymond Rosales[4], Katja Lohmann[1], Lars Bertram[3], Inke R. König[7], Christine Klein[1]
[1]Institute of Neurogenetics, University of Lübeck,, Lübeck, Germany; [2]Department of Neurology, University of Lübeck, Lübeck, Germany; [3]Lübeck Interdisciplinary Platform for Genome Analytics, University of Lübeck,, Lübeck, Germany; [4]Center for Genomic Medicine, Massachusetts General Hospital, Boston, MA, USA; [5]XDP Study Group, Philippine Children's Medical Center, Quezon City, Philippines; [6]Department of Neurosciences, College of Medicine - Philippine General Hospital, University of the Philippines, Manila, Manila, Philippines; [7]Institute of Medical Biometry and Statistics, University of Lübeck, Lübeck, Germany
*Molecular Neurodegeneration* 2019, **14(Suppl 1):**POSTER

**Correspondence:** Christine Klein (christine.klein@neuro.uni-luebeck.de)

### Background
X-linked dystonia-parkinsonism (XDP) is a neurodegenerative disorder showing clinical features of both dystonia and parkinsonism.

XDP is associated with the intronic insertion of a SINE-VNTR-Alu (SVA) retrotransposon in the TAF1 gene1. Recently, the length of the polymorphic (CCCTCT)n domain within this SVA retrotransposon was shown to inversely correlate with age at onset (AAO) in 140 XDP patients2. However, to what extent this repetitive sequence influences the clinical manifestation of XDP remains unknown.

**Methods**

To systematically describe the impact of the (CCCTCT)n domain on disease expressivity, we genotyped 405 SVA carriers and correlated repeat length with the following clinical parameters in patients: dystonia severity (n=19), AAO (n=223), and initial clinical manifestation (n=188). Furthermore, we genotyped post-mortem brain samples from two affected individuals.

**Results**

Repeat length ranged from 30 to 55 in our dataset and showed significant positive and inverse correlations with dystonia severity (r= 0.48, p<0.05) and AAO (r=-0.61, p<0.00001), respectively. In turn, the AAO directly determines whether dystonia (n=149) or parkinsonism (n=39) will be the initial manifestation of XDP. Moreover, we found that repeat length is unstable and exhibits somatic mosaicism in the brain.

**Discussion**

The discovery of the first genetic modifier of XDP expressivity sets up a framework that might yield an efficient and targeted (gene) therapy for this severe disease. In addition, the occurrence of somatic mosaicism in the brain offers insight as to why XDP presents with a neurological phenotype despite the broad requirement of TAF1 for transcription in all cells of the body.

**Conclusions**

Overall, our work provides comprehensive evidence that the length of the (CCCTCT)n domain within the SVA retrotransposon insertion acts as a genetic modifier of disease expressivity in XDP. Functional studies are warranted to elucidate the mechanism(s) by which this hexameric repeat expansion modifies the XDP phenotype.

## P1-F01 NAD+ supply critically modulates mitochondrial vulnerability in glaucoma

Pete A Williams, Simon WM John
HHMI, The Jackson Laboratory, 600 Main Street, Bar Harbor, ME, 04612, USA
*Molecular Neurodegeneration* 2019, **14(Suppl 1):**POSTER

**Correspondence:** Pete A Williams

**Background**

Glaucoma is a complex, multifactorial disease affecting ~80 million people worldwide. It is a leading cause of irreversible blindness. Age, genetics, and high intraocular pressure (IOP) are major risk factors. Currently only treatments to alleviate high IOP have been taken to the clinic. These therapeutics do not treat the neurodegenerative component of glaucoma. Neuroprotective strategies are of great therapeutic need.

**Methods**

RNA-sequencing was used to elucidate the very earliest mechanisms of retinal ganglion cell (RGC) degeneration in the DBA/2J mouse model of glaucoma. Using neurobiological and cell molecular techniques we assessed mitochondrial and metabolic function in RGCs from DBA/2J mice. Using these data we established treatment and gene therapy protocols to treat glaucoma in DBA/2J mice.

**Results**

Metabolism and mitochondrial health decline with age and is exacerbated by periods of high IOP. This is coupled with a marked age-related reduction in retinal NAD+ levels. Restoring NAD+ (via nicotinamide treatment, Nmnat1 gene therapy, and/or the addition of WLDS protein) improves mitochondrial health and potently protects from glaucoma [1-3]. We next identify an IOP-dependent decline in retinal pyruvate levels. Restoring pyruvate levels also prevents glaucoma in mouse and rat models.

**Discussion**

Glaucoma is the most prevalent neurodegeneration, yet there are no neuroprotective therapies. We determine that retinal NAD+ decline is an age- dependent factor that renders RGCs susceptible to the insults of high IOP. Preventing this NAD+ decline robustly protects from glaucoma and protects up to 95% of eyes from severe glaucoma. To develop an RGC-specific, long-term treatment for glaucoma we used Nmnat1 gene therapy (the terminal enzyme in NAD+ production). This is the first instance of a successful gene therapy in a complex age-related disease targeting a common mechanism.

**Conclusions**

Targeting neuronal metabolic decline and neuronal mitochondria may offer safe, neuroprotective treatments for glaucoma and other age-related neurodegenerations.

## P1-F03 RETINAL GANGLION CELL DEGENERATION IN HUMAN GLAUCOMA

James R Tribble[1], Asta Vasalauskaite[2], S Hassan[3], Michael P Fautsch[4], F Sengpiel[2], James E Morgan[1,3]
[1] School of Optometry and Vision Sciences, Cardiff University, Cardiff, UK; [2] School of Biosciences, Cardiff University, Cardiff, UK; [3] University Hospital Wales, Cardiff, UK; [4] Mayo Clinic, Rochester, Minnesota, USA
*Molecular Neurodegeneration* 2019, **14(Suppl 1):**POSTER

**Correspondence:** James R Tribble

**Background**

Glaucoma is a neurodegenerative disease that affects ~80million people worldwide and can lead to irreversible blindness. Animal models of glaucoma have demonstrated early mitochondrial abnormalities [1], synaptic loss and dendritic pruning in retinal ganglion cells [2] (RGC, the retinal output neuron) prior to axon loss and apoptosis. To date no studies have confirmed that these early changes occur in human glaucoma.

**Methods**

Donor glaucoma eyes (n=3; mean age 74.5yrs) and controls (n=3; mean age 83yrs) were used. Images of DAPI stained nuclei were acquired (2-Photon microscope) and cell counts performed (FIJI). Retinal areas corresponding to visual field test locations were prepared for Serial Block Face Scanning Electron Microscopy (Zeiss Sigma VPSEM, Gatan3View2). Volumetric data (79x79x100µm, resolution 19.2x19.2x100nm) were collected and analysed using the TrakEM2 plugin (FIJI). RGC soma, dendrites and mitochondria were segmented and reconstructed in 3D.

**Results**

Global cell density reductions were observed in glaucoma. Single RGCs from glaucomatous (n=6) areas (>20% cell loss) and equivalent areas from controls (n=4 RGCs) were reconstructed. Sholl analysis showed a 58% reduction in area under the curve for mitochondria (P=0.021) and a 71% reduction for synapses (P=0.002) in glaucomatous RGCs compared to controls. Mitochondria occupied 67% less dendritic volume in glaucoma (P=0.009) compared to controls with a marked reduction in cristae integrity. Indices of cytoplasmic vacuolation and autophagosomes were not significantly different in glaucomatous RGCs.

**Discussion**

Our data provide the first evidence that mitochondrial network abnormalities and synapse loss occurs in RGCs prior to cell death in human glaucomatous eyes. These findings concur with similar changes observed in animal models of glaucoma [1,2] and other neurodegenerations [3].

**Conclusions**

Our findings support the concept that surviving RGCs provide a neural substrate for the recovery of vision in glaucoma and raise the potential for neuro-regenerative therapies for patients.

**Funding**

Fight for Sight, UK

## P1-F04 MICRORNA-19a-PTEN AXIS IS INVOLVED IN THE DEVELOPMENTAL DECLINE OF AXON REGENERATION CAPACITY IN RETINAL GANGLION CELLS

Heather K Mak[1], Xu Cao[1], Jasmine SY Yung[1], Shuk Han Ng[2], Christopher KS Leung[1]

[1]Department of Ophthalmology and Visual Sciences, The Chinese University of Hong Kong, Hong Kong, PRC; [2]The Laboratory Animal Services Centre, The Chinese University of Hong Kong, Hong Kong, PRC

*Molecular Neurodegeneration* 2019, **14(Suppl 1):**POSTER

**Correspondence:** Heather K Mak

**Background**

While several transcription factors have been implicated in the regulation of axon regeneration in retinal ganglion cells (RGCs) [3-9], the molecular mechanisms governing the developmental decline in axon regenerative capacity are poorly understood. This study investigated developmental changes of microRNAs and their roles in the regulation of RGC axon regeneration.

**Methods**

Microarray was performed on purified RGCs from Sprague Dawley rats at E21, P6, and P30. The association between developmental decline in miR-19a and upregulation of PTEN was investigated with ISH and Western blot. The impact of miR-19a on axon regeneration was examined in vitro following AAV delivery of miR-19a in isolated rodent and human RGCs, and in vivo in C57BL/6 mice following optic nerve crush and intravitreal injection of AAV-miR-19a-eGFP.

**Results**

76 miRNAs were differentially expressed from E21 to P30 RGCs, among which miR-19a decreased by 2551-fold ($p<0.001$). miR-19a decreased and PTEN increased with age in the ganglion cell layer in rodents, and in isolated human fetal and adult RGCs. Upregulation of miR-19a suppressed PTEN in RGCs by 25.9% ($p=0.001$), increased rodent RGC axon length and growth rate by 2.62- and 2.46-fold ($p\leq0.034$), respectively, and increased human RGC axon and total neurite lengths by 26.7% and 59.8% ($p\leq0.025$), respectively. Upregulation of miR-19a increased the number of regenerated axons by 2-fold following optic nerve crush ($p\leq0.006$).

**Discussion**

Our data uncover a previously unrecognized involvement of the miR-19a-PTEN axis in regulating the developmental decline in axon regenerative capacity of RGCs, and underscore the potential therapeutic application of intravitreal injection of microRNAs to rejuvenate aged RGCs for axon regeneration in the treatment of optic neuropathies.

**Conclusions**

miR-19a is a heterochronic marker that drastically decreases in expression during the maturation of RGCs, which relieves the suppression of PTEN and contributes to the developmental decline of axon regenerative capacity.

## P1-G01 UNFOLDED PROTEIN RESPONSE ACTIVATION IN C9orf72 FTD CASES

Jeroen J.M. Hoozemans[1], Prya Gami[1], Irene van Dijken[1], Lieke H. Meeter[2], Shami Melhem[2], John C. van Swieten[2], Annemieke J.M. Rozemuller[1], Anke Dijkstra[1]

[1]Department of Pathology, VU University Medical Center, Amsterdam, The Netherlands; [2]Alzheimer Center Rotterdam and Department of Neurology, Erasmus Medical Center, Rotterdam, The Netherlands

*Molecular Neurodegeneration* 2019, **14(Suppl 1):**POSTER

**Correspondence:** Jeroen J.M. Hoozemans

**Background**

Multiple mutations are known to correspond with frontotemporal dementia (FTD), but the most common mutation is the G4C2 repeat expansion in C9orf72. Although the mechanism by which C9orf72

expansions could cause neurodegeneration is unclear, several studies have indicated the involvement of the Unfolded Protein Response (UPR). The UPR is a stress response located in the endoplasmic reticulum (ER) that protects the cell against misfolded proteins through activation of the ER-stress sensors PERK, IRE1 and ATF6. Chronic activation or aberrant signaling of the UPR results in neurodegeneration.

**Methods**

Using immunohistochemistry we assessed the presence of UPR activation markers phosphorylated PERK and phosphorylated IRE1alpha in the frontal cortex, hippocampus and cerebellum of FTD patients with the G4C2 repeat expansion in C9orf72 (n=17) and non-neurological control cases (n=7). The presence of UPR activation was compared with the occurrence of pTDP-43, P62, and dipeptides (GA, GR, GP).

**Results:**

In the frontal cortex and hippocampus no difference was observed in the occurrence of pPERK between control and C9-FTD cases. Interestingly, the occurrence of pPERK was increased in the granular layer of the cerebellum in C9-FTD cases. In contrast, pIRE1alpha was significantly increased in the frontal cortex and not in the hippocampus and cerebellum. No clear correlation between the occurrence UPR markers and pTDP-43, P62, and dipeptides was observed.

**Conclusions**

We report increased levels of UPR markers in C9- FTD, which varies between brain regions. Our data suggest that the UPR can be differentially regulated in different brain regions in one neurological disease.

## P1-G02 PROGRANULIN GENE THERAPY IMPROVES PATHOLOGY AND REVERSES SOCIAL DEFICITS IN MOUSE MODELS OF FRONTOTEMPORAL DEMENTIA AND NEURONAL CEROID LIPOFUSCINOSIS DUE TO PROGRANULIN MUTATIONS

Andrew E. Arrant, Vincent C. Onyilo, Daniel E. Unger, Erik D. Roberson
Departments of Neurology and Neurobiology, Center for Neurodegeneration and Experimental Therapeutics, University of Alabama at Birmingham School of Medicine, Birmingham, AL, USA
*Molecular Neurodegeneration* 2019, **14(Suppl 1):**POSTER

**Correspondence:** Andrew E. Arrant

**Background**

Loss-of-function mutations in progranulin (GRN) are a major autosomal dominant cause of Frontotemporal Dementia (FTD). GRN mutations exhibit a gene-dose effect, with homozygous GRN mutations causing the lysosomal storage disorder Neuronal Ceroid Lipofuscinosis (NCL). All known disease-causing GRN mutations are loss-of-function mutations, most of which cause progranulin haploinsufficiency. Therefore, boosting progranulin levels is a rational approach to treatment.

**Methods**

We generated an AAV2/1-progranulin vector (AAV- Grn) to test whether restoration of progranulin could correct NCL-like pathology in Grn–/– mice and social behavior deficits in Grn+/– mice. AAVGrn or an AAV-GFP control vector were infused into the medial prefrontal cortex (mPFC) of 10–12 month-old wild-type, Grn+/–, and Grn–/– mice. Grn–/– mice were euthanized for assessment of pathology 8–10 weeks later, and Grn+/– mice were assessed for social behavior 4–6 weeks later.

**Results**

AAV-Grn reduced lipofuscinosis and normalized cathepsin D activity in Grn–/– mice. AAV-Grn also reduced microgliosis in Grn–/– mice in several brain regions. At the AAV injection site, AAV-Grn induced an apparent non-self reaction to progranulin that was not observed in wild-type or Grn+/– mice and is unlikely to occur in FTD-GRN patients. AAV-Grn reversed social deficits and normalized markers of lysosomal dysfunction in Grn+/– mice.

**Discussion**

These data show that restoration of progranulin to progranulin-insufficient mice reduces FTD/NCLlike pathology, normalizes markers of lysosomal dysfunction, and reverses deficits in social behavior. Our AAVGrn vector expressed progranulin with a C-terminal tag that disrupted binding of progranulin to sortilin, showing that sortilin is not required for these beneficial effects of progranulin.

**Conclusions**

These data provide support for the use of progranulin-boosting therapies in GRN mutation carriers.

## P1-G03 miR-874 REGULATES EXTRACELLULAR PROGRANULIN LEVEL BY TARGETING SORTILIN IN THE BRAIN

Jaekwang Kim[1,2], Chris W. Lee[1,3], Leonard Petrucelli[1], Jungsu Kim[1]
[1]Department of Neuroscience, Mayo Clinic College of Medicine, Jacksonville, FL, 32224, USA; [2]Department of Neural Development and Disease, Korea Brain Research Institute, Daegu, Republic of Korea; [3]Biomedical Research Institute of New Jersey, Cedar Knolls, NJ USA
*Molecular Neurodegeneration* 2019, **14(Suppl 1):**POSTER

**Correspondence:** Jaekwang Kim

**Background**

Mutations in the Progranulin gene (GRN) are a major cause of frontotemporal lobar degeneration with ubiquitin positive inclusions (FTLD-U). GRN polymorphisms are also known to modify the risk for Alzheimer's disease (AD) [1]. Therefore, understanding Progranulin (PGRN)'s function and regulatory mechanisms may have broad relevance for both FTLD and AD. Recently, Sortilin (Sort1), a type-1 receptor, has been identified as a key regulator of PGRN in the brain which mediates endocytosis of extracellular PGRN [2]. Thus, identifying regulatory mechanisms of Sort1 is likely to provide novel opportunities to better understand PGRN regulation.

**Methods**

miRNAs which targets Sort1 were first identified using miRNA-target prediction algorithms and then validated by luciferase assay and western blot analysis. To express miRNA in the brain, we injected AAV8 encoding miRNA or control empty vector (Ctl) into cerebral ventricles of newborn C57BL6/J mice. Then, we analyzed the levels of Sort1 and PGRN in cortex and hippocampus at 3 months of age.

**Results**

Here, we demonstrated that microRNA-874 (miR- 874) suppresses Sort1 expression at the posttranscriptional level through directly binding to the 3'UTR of its mRNA. In contrast, inhibition of endogenous miR-874 significantly increased the levels of Sort1 in neuronal N2a cells, suggesting that Sort1 expression is actively suppressed by endogenous miR-874 under basal condition. Importantly, overexpression of miR-874 increases the levels of extracellular PGRN by suppressing Sort1 expression in neuronal cells, whereas inhibition of miR-874 decreases the levels of extracellular PGRN. Moreover, we demonstrated that cerebral expression of miR-874 using adeno-associated virus significantly increases the levels of extracellular PGRN by suppressing Sort1 expression in cortices and hippocampi of C57BL6/J mice.

**Conclusions**

Taken together, we identified miR-874 as a novel negative regulator of Sort1 expression in the brain. Our data further support a novel regulatory mechanism of extracellular PGRN by miR-874

## P1-G07 LYSOSOMAL CHANGES CHARACTERIZING FRONTOTEMPORAL DEMENTIA

M Del Campo M[1], I Rodal[2], A Rábano[2], CE Teunissen[1]
[1]Neurochemistry lab,Clinical Chemistry, Amsterdam Neuroscience, VU University Medical Center, The Netherlands; [2]Brain bank tissue (BT)-CIEN, CIEN Foundation, Madrid, Spain
*Molecular Neurodegeneration* 2019, **14(Suppl 1):**POSTER

**Correspondence:** M Del Campo M

**Background**

Frontotemporal lobar degeneration (FTLD) is the second most prevalent dementia in young patients. Despite its etiology remains largely unknown, genetic studies suggest an involvement of the autophagy/lysosome system in FTLD pathogenesis. We previously observed several lysosome- related proteins changed in the cerebrospinal fluid (CSF) of control cases and FTLD pathological subtypes (FTLD-Tau and FTLD-TDP)(1). Here we performed a pilot characterization of those lysosome-related proteins in FTLD postmortem tissue to unravel whether changes occur also in the brain and their potential as novel players in the development of the different FTLD subtypes.

**Methods**

Immunohistochemical characterization of GLA, LAMTOR2, HexA and CTSD was performed in post-mortem frontal cortex of FTLD-Tau (n=6), FTLD-TDP (n=5) and age- matched nondemented controls (n=4). CTSD was further analysed in an additional FTLD cohort (n=17) extended with cases with Alzheimer's disease (AD, n=6) and amyotrophic lateral sclerosis (ALS, n=6).

**Results**

GLA immunoreactivity was comparable among the different diagnostic groups. Intraneuronal LAMTOR2 tended to be increased in FTLD-TDP compared to controls (p=0.06). Intraneuronal HexA was significantly reduced in both FTLD- TDP and FTLD-Tau ($p<0.05$). Strikingly, a nuclear-like pattern of CTSD was observed in sporadic cases of FTD-TDP and specific cases of FTD-Tau (Pick's disease, $p<0.05$). CTSD nuclear-like staining was also present in AD ($p<0.05$) but absent in ALS or non-demented controls.

**Discussion**

We found that both LAMTOR2 and HexA were changed in FTLD cases. The nuclear-like inclusions of CTSD observed in specific subtypes of FTLD and AD suggest a possible miss-localization of this protein in sporadic cases developing dementia. Since CTSD is involved in the degradation of protein aggregates within the brain, aberrant localization could enhance protein aggregation and misfolding, thereby participating in the pathogenesis of specific types of dementia.

**Conclusion**

This data suggest that the autophagy/lysosome system is compromised in FTLD cases, revealing new potential players in FTLD.

## P1-G08 AMYOTROPHIC LATERAL SCLEROSIS-LINKED MUTATIONS IN FUS PROTEIN AFFECT U7 snRNP ACTIVITY

Ankur Gadgil, Agata Stepien, Katarzyna Dorota Raczynska
Department of Gene Expression, Institute of Molecular Biology and Biotechnology, Adam Mickiewicz University, Poznan, Poland
*Molecular Neurodegeneration* 2019, **14(Suppl 1):**POSTER

**Correspondence:** Ankur Gadgil

In the inherited form of amyotrophic lateral sclerosis (ALS) mutations in Fused in Sarcoma (FUS) gene are identified. The gene encodes a multifunctional, nuclear RNA-binding protein of the hnRNP family [1, 2]. Most of the reported FUS linked ALS mutations are missense mutations found in highly conserved C-terminus Nuclear Localization Signal [3]. Previous findings from our lab have shown that FUS protein interacts with U7 snRNP and play a role as positive regulator for efficient and correct 3' end processing of replication dependent core canonical histone transcripts [4]. This finding supports previously reported observations emphasizing the fact, how the fidelity of histone gene regulation is important for gene regulation and genome stability [5] and that FUS deficiency results in chromosomal instability [6]. FUS mutants are found to mislocalize with U1 snRNP and thus it becomes important to check if FUS can also mislocalize U7 snRNP. Microscopic experiments together with Immunoprecipitation experiment show how mutations influence FUS and U7 snRNP interaction.

## P1-G09 DISRUPTION OF FUS/TLS BY GLUTAMINE EXPANDED ATXN7

Frida Niss, Wajiha Zaidi, Elisavet Papadopoulou, Einar Hallberg, Anna-Lena Ström
Department of Biochemistry and Biophysics, Stockholm University, Sweden
*Molecular Neurodegeneration* 2019, **14(Suppl 1):**POSTER

**Correspondence:** Frida Niss

**Background**

FUS/TLS is an RNA/DNA-binding protein which regulates RNA metabolism [1]. Mutations in FUS/TLS can cause familial forms of the neurodegenerative disease amyotrophic lateral sclerosis (ALS) [2, 3]. In addition, FUS/TLS has been found to be a component of aggregates formed by the mutant proteins in polyglutamine diseases, like Huntington's disease and spinocerebellar ataxia type 7 (SCA7) [4, 5]. However, if disruption of FUS/TLS function occurs in polyglutamine diseases and if this contributes to pathology is still unclear. In this study we therefore investigated how expression of the SCA7 disease protein ATXN7 affects FUS/TLS properties and functions.

**Methods**

The expression and subcellular localization of FUS/TLS was investigated in an inducible SCA7 cell model, by cell fractionation, western blot, filter trap and microscopy. In addition, the mRNA levels of FUS/TLS regulated mRNAs were determined by semi-quantitative RT-PCR.

**Results**

We found that upon induction of mutant ATXN7 expression the total FUS/TSL level increased and co- localization of FUS/TSL and insoluble ATXN7 aggregates could be observed. Moreover, we found that the levels of several FUS/TLS regulated mRNAs were decreased in SCA7 cells.

**Discussion**

Although FUS/TLS was sequestered into mutant ATXN7 aggregates, an increase in the total FUS/TLS level could be observed in cells expressing the SCA7 disease protein. Additionally, despite the increased abundance of FUS/TLS, the RNA regulatory function was disrupted. Consistent with this, overexpression of wild-type FUS/TLS in mice has previously been shown to cause neurodegeneration and result in an ALS-like phenotype [6].

**Conclusions**

Disruption of FUS/TLS could contribute to the neuronal dysfunction in SCA7 and potentially other polyglutamine diseases.

## P2-H01 ACD855, DEVELOPMENT OF A POSITIVE MODULATOR OF NEUROTROPHIN SIGNALING FOR THE SYMPTOMATIC TREATMENT OF ALZHEIMER'S DISEASE AND PARKINSON'S DISEASE

Pontus Forsell[1,2,3], Märta Dahlström[1,4], Nather Madjid[1,2], Gunnar Nordvall[1,2,3], Johan Lundkvist[1,2,3], Magnus Halldin[1,2], Erika Vazquez-Juarez[5], Maria Lindskog[5], Maria Eriksdotter[4,6], Bengt Winblad[3,6], Johan Sandin[1,2,3]

[1]AlzeCure Foundation, Karolinska Institutet Science Park, Huddinge, Sweden; [2]AlzeCure Pharma AB, Hälsovägen 7, Huddinge, Sweden; [3]Department of Neurobiology, Care Sciences and Society, Division of Neurogeriatrics, Karolinska Institutet, Sweden; [4]Department of Neurobiology, Care Sciences and Society, Division of Clinical geriatrics, Karolinska Institutet, Sweden; [5]Department of Neurobiology, Care Sciences and Society, Division of Translational Alzheimer neurobiology, Karolinska Institutet, Sweden, [6]Theme Aging, Karolinska university hospital, Huddinge, Sweden

*Molecular Neurodegeneration* 2019, **14(Suppl 1):**POSTER

**Correspondence:** Pontus Forsell

**Objectives**

Neurotrophins are related proteins including NGF, BDNF, NT3 and NT4/5. There are numerous studies in animals demonstrating that neurotrophins are important for neuronal function. The genetic linkage between BDNF and cognitive dysfunction in AD and PD, supports the objective of this project, which is to develop positive modulators of neurotrophin signaling.

**Methods**

Modulators of neurotrophin signaling were identified by a HTS and characterized in secondary assays. ACD855 was investigated with respect to ERK phosphorylation in rat hippocampus and in in vivo models, including passive avoidance, Morris water maze and novel object recognition. ACD855 is currently being tested in preclinical GLP-toxicological and safety studies.

**Results**

ACD855 was identified as a positive modulator of neurotrophin signaling. Secondary tests in primary neurons and rat brain slices confirmed its activity as a positive modulator of BDNF signaling. ACD855 has low agonistic properties but potentiates BDNF-induced phosphorylation of ERK and facilitates the induction of LTP in the hippocampus. ACD855 can reverse scopolamine-induced memory impairment in a TrkB-dependent manner and is additive to physostigmine in the passive avoidance model. We have also tested ACD855 in Morris water maze and novel object recognition with positive outcome. Furthermore, in the forced swim test, ACD855 showed similar efficacy as Fluoxetine, a well-established anti-depressant. Results from an APP-transgenic mouse model will also be presented as well as preliminary GLP-tox and safety data.

**Conclusions**

We have identified ACD855 as a synaptic enhancer and as a potentially new cognitive enhancer. ACD855 stimulates BDNF signaling in different cellular and functional contexts in vitro as well as in multiple behavioral paradigms in vivo. The observation that ACD855 acts in an additive manner to physostigmine and is equipotent to fluoxetine, suggest a broad applicability of ACD855 for CNS disorders. ACD855 is in preclinical development and ideally suited as a symptomatic treatment for cognitive dysfunction.

## P2-H02 DEVELOPMENT OF TISSUE-SELECTIVE ABCA1 AGONISTS AS ALZHEIMER'S DISEASE THERAPEUTICS

Cutler Lewandowski[1], Manel Ben Aissa[1,2], Sue Lee[1], Bhargava Karumudi[1,2], Gregory Thatcher[1,2]
[1]Department of Medicinal Chemistry and Pharmacognosy, University of Illinois-Chicago, USA; [2]UICentre, University of Illinois-Chicago, USA
*Molecular Neurodegeneration* 2019, **14(Suppl 1):**POSTER

**Correspondence:** Cutler Lewandowski

**Background**

Apolipoprotein E (APOE) ε4 allele is the strongest risk factor for sporadic Alzheimer's disease (AD) [1]. ApoE4-containing lipoproteins have lower lipid content, which decreases stability and contributes to loss of lipoprotein function [2]. To correct these deficits, we have developed tissue-selective ABCA1 agonists (TSAAgs) that induce central nervous system expression of cholesterol transporter ABCA1, thereby increasing lipid content of apoE4- containing lipoproteins, with minimal impact on peripheral lipogenesis. TSAAgs also ameliorate additional aspects of AD, including neuroinflammation and insulin resistance [3,4].

**Methods**

High-throughput screening (HTS) utilized luciferase reporter elements expressed by CCF-STTG1 astrocytoma (primary screen) and HepG2 hepatocellular carcinoma cells (counter screen) linked to ABCA1 and SREBP1c promoters, respectively. Iterative chemical synthesis was used to develop novel analogs of HTS hits to establish TSAAg structure-activity relationships. Analogs were tested in vitro via PCR/ immunoblot for both lipid- and insulin-related genes, ELISA for inflammatory markers, and a fluorescent cholesterol efflux assay.

**Results**

Prioritized HTS hits – those demonstrating anti- inflammatory and insulin-sensitizing properties in addition to TSAAg activity – served as scaffolds to generate a library of structural analogs. In vitro evaluation of this analog library established structure-activity relationships that identified compounds with improved TSAAg activity and guided further structural modification.

**Discussion**

The results demonstrate a proof-of-concept to develop TSAAgs with multifunctional therapeutic potential for Alzheimer's disease. Future in vivo experiments in healthy mice will establish pharmacokinetic profiles, determine magnitude and mechanisms of tissue-selective ABCA1 induction, and monitor alterations in peripheral lipogenesis. Finally, treatments in the EFAD mouse model will assess TSAAg effects on cognitive and pathological deficits [5].

**Conclusions**

Our study represents a novel strategy to develop small molecule drug candidates that target multiple aspects of AD pathology. Upon conclusion of this project we hope to establish TSAAg compounds as leads for further pharmaceutical development and human clinical testing.

## P2-H03 INTERVENTION MECHANISMS OF EFFECTOR MOLECULES ON AMYLOID-BETA AND TAU PROTEIN AGGREGATES

Julia Stockmann[1], Andreas Nabers[1], Atanu Archaya[2], Léon Beyer[1], Victor Batista[2], Klaus Gerwert[1]
[1]Department of Biophysics, Ruhr University Bochum, Bochum, Germany; [2]Yale Department of Chemistry, Yale University, New Haven, Connecticut, USA
*Molecular Neurodegeneration* 2019, **14(Suppl 1):**POSTER

**Correspondence:** Julia Stockmann

**Background**

Protein aggregation plays a significant role in Alzheimer's disease (AD) progression and therapy. The aggregation of Amyloid-beta (Aß) peptides and tau proteins lead to the forming of plaques and neurofibrillary tangles in the brain. For AD treatment effector molecules have been tested, which are proposed to trigger the unfolding of those proteins.

**Methods**

An immuno-infrared-sensor [1,2] was used, which enables the immobilization of Aß peptides or tau proteins in different secondary structure isoforms, simultaneously or separately. Different effector molecules, such as berberine or methylene blue, were flushed over the immobilized proteins in a flow-through system to investigate the intervention potential on aggregated biomarkers.

**Results**

The recorded amide I band is sensitive to the conformation of the peptides displaying amide I bands. Synthetic Aß fibrils showed a characteristic amide I maximum around 1628 cm1 indicating a high content of ß-sheet structures, while monomeric and disordered helical proteins had maxima around 1648cm1. Aß fibrils and tau tangles treated with effector molecules showed a distinct shift of the amide I maxima to higher wavenumbers [3].

**Discussion**

Treatment of Aß and tau with effector molecules changed the amide I bands over time indicating a change in the secondary structure of such proteins. The extent of the amide I band gave information about the efficiency of the effector molecule. Methylene blue unfolds pathogenic tau proteins, which results in a shift of the amide I band from fibrillary to a more monomeric and unfolded helical state [3]. On the other hand, berberine seems to decelerate Aß aggregation [3].

**Conclusions**

The immuno-infrared sensor enables the analysis of the secondary structure of Aß peptides and tau proteins. Thus, it is possible to directly monitor the intervention of effector molecules on pathogenic proteins. This approach may be used to identify promising drug candidates in-vitro from a huge data base for AD treatment and clinical trials.

## P2-H04 AN IMMUNO-INFRARED-ASSAY FOR BLOOD-BASED DIAGNOSIS OF ALZHEIMER'S DISEASE AND SELECTION OF DRUG CANDIDATES

Andreas Nabers, Julia Lange, Julia Stockmann, Klaus Gerwert
Department of Biophysics, Ruhr University Bochum, Bochum, Germany
*Molecular Neurodegeneration* 2019, **14(Suppl 1):**POSTER

**Correspondence:** Andreas Nabers

**Background**

Aß and Tau aggregation plays a significant role in Alzheimer's disease (AD) pathology. Clearance and dissociation of such aggregates are promising targets in AD therapy. For a successful therapeutic intervention, early diagnosis of AD as well as the easy analysis of the drug mechanism is a prerequisite. Both can be monitored by an immuno-infrared-sensor recording the secondary structure distribution of Aß and Tau in body fluids [1-3].

**Methods**

The immuno-infrared-sensor was used to extract either the total Aß or Tau fraction from CSF or blood [1-2]. The secondary structure distribution of these biomarkers was used for early AD detection [1-2]. Moreover, the sensor was applied to study the mechanism of novel drug-candidates on human Aß and Tau aggregates in-vitro [3].

**Results**

The recorded amide I band of the total Aß and/or Tau fraction is sensitive to their respective secondary structure distribution. AD subjects showed a significant downshift of the amide I maximum in various clinical studies as compared to controls indicating a higher content of ß-sheet structures [1,2]. Therewith, AD could be detected in its earliest disease stages based on blood plasma and CSF analyses. Moreover, different effector molecules were tested for their intervention mode on ß-sheet enriched Aß and Tau species [3]. As a result, treatment of aggregated Aß and Tau with different effector molecules resulted in a distinct upshift of the amide I maximum indicating an increase in monomeric isoforms after drug intervention.

**Discussion**

The secondary structure change of Aß and Tau is an early event in AD pathology preceding clinical manifestation. Thus, the secondary structure distribution of Aß and Tau seems to be a reliable marker for AD detection in its earliest disease stages. In addition, treatment of pathogenic Aß and Tau with selected effector molecules dissociated ß-sheet structures over time.

**Conclusions**

The immuno-infrared-sensor enables the analysis of the secondary structure of Aß and Tau. Thus, AD could be detected in its early disease stages. Moreover, the assay was used to investigate the intervention effect of drug- candidates on pathogenic Aß and Tau. This approach may be used to identify and pre-select individuals for clinical trials.

## P2-H05 BRICHOS: INITIAL STUDIES TO TREAT NEUROPATHOLOGY AND MEMORY DYSFUNCTION IN ALZHEIMER KNOCK-IN MOUSE MODELS

Shaffi Manchanda, Simone Tambaro, Per Nilsson and Jan Johansson
Department of Neurobiology, Care Sciences and Society (NVS), Division of Neurogeriatrics, Karolinska Institutet, Novum 5th floor, 14157 Huddinge, Sweden
*Molecular Neurodegeneration* 2019, **14(Suppl 1):**POSTER

**Correspondence:** Shaffi Manchanda

**Background**

Alzheimer's disease (AD) is characterized by progressive deposition of extracellular amyloid plaques of amyloid-β (Aβ) peptide and intracellular neurofibrillary tangles of phosphorylated tau protein followed by synaptic dysfunction, neuronal loss and cognitive decline. BRICHOS (Bri 2, Chondromodulin, pro-SP-C) was identified as chaperone like domain derived from Bri2 protein associated with familial dementia, chondromodulin and prosurfactant protein C (pro-SP-C) [1]. It has been shown to prevent Aβ- induced neurotoxicity and reduction in ɣ-oscillations (implicated in learning and memory) in animal models [2]. In line of these findings, the current study was aimed to evaluate the passage of Bri2 BRICHOS over blood brain barrier (BBB) and its potency to ameliorate Aβ pathology, neuroinflammation and memory performance in novel APP- knock-in AD mouse models, APPNL-F harbouring the Swedish (KM670/671NL) and Beyreuther/Iberian (I716F) mutations, and APPNL-G-F mice additionally harbouring the arctic mutation.

**Methods**

APPNL-G-F mice (12-13 months old) were injected with PBS or recombinant human Bri2 BRICHOS (20mg/kg) intravenously. After 2 hours, cerebrospinal fluid (CSF) and brain were collected from each mice and analysed for the permeability across BBB by western blotting.

**Results**

Recombinant Bri2 BRICHOS was detected in the CSF and in brain of APPNL-G-F after 2 hours of its administration compared to their PBS treated control counterparts.

**Discussion**

Presence of recombinant Bri2 BRICHOS in the CSF and in brain reveals that it can cross BBB and reach brain parenchyma. This data provides us the basis to investigate the therapeutic potential of Bri2 BRICHOS in APP-knock-in mice. We will use Morris Water Maze and Novel Object Recognition tasks to test memory based functions and analyse the Aβ burden and neuroinflammation by immunohistochemistry and western blots.

**Conclusions**

These preliminary findings provide us incentives to explore BRICHOS domain as a therapeutic candidate against AD-associated neuropathology and cognitive dysfunctions.

## P2-H06 NOVEL PRECLINICAL MILD TRAUMATIC BRAIN INJURY AND AGING MOUSE MODEL: A MULTIDIMENSIONAL APPROACH TO DEVELOP DISEASE MODIFYING THERAPIES

Sue Hyun Lee[1], Manel Ben Aissa[1,5], Yue-ting Wang[1,2], Fernando Tobias[3], Stephanie Cologna[3], Olusola Ajilore[4], Gregory R.J. Thatcher[1,5]
[1]Department of Medicinal Chemistry and Pharmacognosy, University of Illinois at Chicago, IL, USA; [2]Mass Spectrometry, Metabolomics and Proteomics Facility, University of Illinois at Chicago, IL, USA; [3]Department of Chemistry, University of Illinois at Chicago, IL, USA; [4]Department of Psychiatry, University of Illinois at Chicago, IL, USA; [5]UICentre for Drug Discovery, University of Illinois at Chicago, IL, USA
*Molecular Neurodegeneration* 2019, **14(Suppl 1):**POSTER

**Correspondence:** Sue Hyun Lee

**Background**

Recent reports associate traumatic brain injury (TBI) to earlier onset dementia. However, the link between mild trauma and its role to deplete a person's "cognitive reserve" as they age is still unknown. Our primary objective is to develop a preclinical model where functional damage and future consequences induced by mTBI that contribute to increased risk of dementia can be identified and disease modifying strategies can be tested.

**Methods**

We developed an oxidative stress-induced mouse model (Aldh2-/-) in conjunction with a closed head injury model to mimic post-mTBI cognitive deficits and neuroinflammatory pathology. Brain proton magnetic resonance spectroscopy (MRS) was used to assess changes using noninvasive measures of early disease identification, and matrix-assisted laser desorption/ionization imaging (MALDI) mass spectrometry was used to elucidate molecular distributions in tissue sections, ranging from 24 hrs-1-month post-injury. Novel library of small molecules (NMZ) that reactivate CREB through NO/cGMP signaling pathways was used as potential therapeutics for TBI.

**Results**

Aldh2-/- mice exhibited accelerated cognitive deficits which were further characterized using a chemoproteomic approach to identify differentially expressed proteins linked to accelerated aging. Interestingly, when mTBI was administered, it led to exacerbation of neuroinflammation, neuronal and synaptic pathology, and post-concussive syndrome 24 hrs post-injury. In addition, MRS and MALDI data suggested that early changes can be tracked non- invasively and spatially mapped. NMZ tested in this model reversed post-concussive syndrome, decreased inflammation, and alleviated damage from other contributors of mTBI.

**Discussion**

These studies provide greater insight into the underlying mechanisms of TBI leading to early diagnosis, target identification, and treatment to alleviate higher dementia risk.

**Conclusions**

Our studies introduce full characterization of a novel mouse model of mTBI that provide valuable resource to further identify potential biomarkers for detection so that disease modifying therapies could be developed.

## P2-H07 BLOOD-BRAIN-BARRIER PERMEABILITY OF THE Bri2 AND THE proSP-C BRICHOS DOMAINS

Simone Tambaro, Lorena Galan-Acosta, Gefei Chen, Jenny Presto, Per Nilsson and Jan Johansson
Department of Neurobiology, Care Sciences and Society (NVS), Division of Neurogeriatrics, Karolinska Institutet, Novum, 141 57 Huddinge, Sweden
*Molecular Neurodegeneration* 2019, **14(Suppl 1):**POSTER

**Correspondence:** Simone Tambaro

**Background**

Preventing protein misfolding and aggregation is a therapeutic strategy for neurodegenerative disorders such as Alzheimer´s disease (AD) [1]. Recently, we reported that the chaperone BRICHOS domains from human surfactant protein C (proSP-C) and from integral membrane protein 2B (Bri2) efficiently delay Aβ42 fibril formation and its neurotoxicity in vitro [2] and in vivo [3, 4] assays. Based on these results, we investigated the potential of BRICHOS as an anti- Aβ aggregation AD drug; in this study we analyzed the serum half-life and the blood-brain barrier (BBB) permeability of proSP-C and Bri2 BRICHOS.

**Methods**

Human proSP-C and Bri2 BRICHOS domains were expressed in E. coli cells and purified by immobilized metal affinity and size exclusion chromatography. The BRICHOS domains were injected intravenously in adult wild-type mice (C57BL/6NTac). Blood samples were collected at 5, 30, 60 and 120 minutes after the injections. At 120 minutes post injection CSF samples were collected from the cisterna magna, mice were perfused and brains were removed and analyzed by immunoprecipitation, western blot, ELISA and immunohistochemistry.

**Results**

Bri2 BRICHOS was detected in the CSF and in the brain parenchyma two hours after intravenous administration in about 75% of the treated mice. Positive staining for Bri2 BRICHOS was observed in the choroid plexus and in some cases in the cortex. On the contrary, proSP-C BRICHOS was not detected in the brain even though it exhibited a higher serum half-life (75±8min) compared to Bri2 BRICHOS (29±3min).

**Discussion**

These findings support that Bri2 BRICHOS can reach the brain parenchyma after systemic injection, and indicate also that the BRICHOS domain from proSP-C and Bri2 have different pharmacokinetic properties and BBB permeability.

**Conclusions**

Taken together, these results provide the bases for a further exploration of Bri2 BRICHOS as a new therapeutic strategy for neurodegenerative disorders, in particular AD.

## P2-H08 ANTIBODY-BASED PET IMAGING DETECTS LOWERED BRAIN AMYLOID-BETA LEVELS AFTER BACE-1 INHIBITION (NB-360)

Silvio R Meier[1], Stina Syvänen[1], Greta Hultqvist[2], Xiaotian T Fang[1], Sahar Roshanbin[1], Lars Lannfelt[1,3], Ulf Neumann[4], Dag Sehlin[1]
[1]Department of Public Health and Caring Sciences, Uppsala University, Uppsala, Sweden; [2]Department of Pharmaceutical Biosciences, Uppsala University, Uppsala, Sweden; [3]BioArctic AB, Stockholm, Sweden; [4]Neuroscience Research, Novartis Institutes for BioMedical Research, Basel, Switzerland
*Molecular Neurodegeneration* 2019, **14(Suppl 1):**POSTER

**Correspondence:** Silvio R Meier

**Background**

Amyloid-beta (Aβ) positron emission tomography (PET) has become an important tool for specific diagnosis of Alzheimer's disease (AD). However, all available radioligands, such as [11C]PIB [1], reflect levels of insoluble Aβ plaques and may thus not be sensitive to treatment aimed to reduce soluble Aβ. The aim of the present study was to investigate if a novel PET radioligand, based on an antibody directed towards soluble aggregates of Aβ, could be used to detect changes in Aβ levels after treatment with a β- secretase (BACE-1) inhibitor.

**Methods**

Transgenic animals (tg-ArcSwe [2], model of Aβ pathology), were treated during 3 months with BACE-1 inhibitor NB-360 [3] and compared to an untreated control group. After treatment, animals were PET scanned with Aβ protofibril selective radioligand [124I]Rmab158-scFv8D3 [4]. A baseline group also underwent PET scanning. Brain tissue was isolated after PET and Aβ levels were measured in tissue homogenates.

**Results**

Treated animals showed significantly lower in vivo PET signal than untreated animals, and further, similar signals to the baseline group. The PET results corresponded well with decreased Aβ levels measured in post mortem brain.

**Discussion**

Antibody based PET imaging benefits from very specific binding to the target structure. With our protofibril selective radioligand we are able to image a soluble and dynamic species of Aβ which seems to be a promising marker for early diagnosis and to monitor disease progression [5] and treatment effects.

**Conclusions**

Several AD treatments [6] are currently in phase 2 and 3 clinical trials but there are limited possibilities to study their effects on a molecular level in vivo. With our previously developed protofibrils selective radioligand [124I]Rmab158-scFv8D3 we here demonstrate the ability to monitor treatment effects with PET imaging in tg-ArcSwe mice.

## P2-H09 PROFILE OF CNP520, A BACE-1 INHIBITOR FOR PREVENTION STUDIES IN ALZHEIMER'S DISEASE

Ulf Neumann[1], Derya Shimshek[1], Mike Ufer[1], Marie-Laure Rouzade-Dominguez[1], Carine Kolly[1], Gunilla Huledal[1], Nicole Pezous[1], Ana Graf[2], Cristina Lopez-Lopez[2]

[1]Novartis Institute for Biomedical Research, Basel, Switzerland, [2]Novartis Global Drug Development, Basel, Switzerland

*Molecular Neurodegeneration* 2019, **14(Suppl 1):**POSTER

**Correspondence:** Ulf Neumann

**Background**

Stopping amyloid-β (Aβ) deposition by BACE-1 inhibition appears to be a promising strategy to treat Alzheimer's disease (AD), but treatment in established dementia stages was unsuccessful. We hypothesize that BACE-1 inhibitor treatment needs to start in early stage Aβ deposition and before the onset of significant neurodegeneration. Prevention treatment puts high hurdles on the safety and tolerability, to be addressed already in the drug design and selection process.

**Methods**

CNP520 was designed and profiled in vitro, using animal pharmacological, pharmacokinetic and metabolism studies and underwent toxicological profiling with oral studies up to 39 weeks duration Clinical Phase I and Phase IIa studies in healthy elderly volunteers established its safety, tolerability, and active dose range.

**Results**

CNP520 is a potent and selective BACE-1 inhibitor in vitro. Due to its high brain penetration and plasma protein binding, free compound levels in the periphery are low. Significant Aβ reduction was observed in animals. Results of toxicology studies have not raised major safety concerns. No effects on myelin, muscle spindles, retina, pigmented organs were observed. Humans Phase I studies showed a dose- and time-dependent reduction of CSF Aβ, and a pharmacokinetic profile suitable for once-daily dosing. A 3-months study showed that CNP520 is safe and tolerated in a dosing range that result in 90% reduction of CSF Aβ.

**Discussion**

The profile of CNP520 supports its use in prevention studies of AD. Generation Study 1 and 2 have been initiated, which aim to test CNP520 at 15 or 50 mg in a population of enhanced risk to develop AD, patients are being included based on their age, APOE4 genotype and Aβ positivity.

**Conclusions**

Properties of CNP520 make it suited for the use in prevention trials of AD, the ongoing clinical studies will allow to test the concept of prevention treatment in AD

**Clinical trial registration**

EUDRACT number 2013-005576-18.

## P2-H10 DEVELOPMENT OF CRISPR/Cas AS A NOVEL TREATMENT STRATEGY FOR FAMILIAL FORMS OF ALZHEIMER'S DISEASE

E Konstantinidis[1], B György[2,3], C Lööv[4], MP Zaborowski[2,5], A Erlandsson[1], X Aguilar[1], M Essand[6], S Takeda[4], BP Kleinstiver[7,8,9,10], C Commins[4], K Kastanenka[4], D Mu[2], A Volak[2], V Giedraitis[1], L Lannfelt[1], CA Maguire[2], JK Joung [7,8,9,10], BT Hyman[4], XO Breakefield[2], M Ingelsson[1,2]

[1] Department of Public Health, Molecular Geriatrics, Rudbeck Laboratory, Uppsala University, Uppsala, Sweden; [2] Department of Neurology and Radiology, Massachusetts General Hospital and Center for NeuroDiscovery, Harvard Medical School, Boston, Massachusetts, USA; [3] Department of Neurobiology, Harvard Medical School, 220 Longwood Avenue, Boston, Massachusetts 02115, USA; [4] Department of Neurology and Radiology, Massachusetts General Hospital and Alzheimer's Disease Research Center, Harvard Medical School, Boston, Massachusetts, USA; [5] Department of Gynecology, Obstetrics and Gynecologic Oncology, Division of Gynecologic Oncology, Poznan University of Medical Sciences, 60-535 Poznan, Poland; [6] Department of Immunology, Genetics and Pathology, Uppsala University, Uppsala, Sweden; [7] Molecular Pathology Unit, Massachusetts General Hospital, Charlestown, Massachusetts, USA; [8] Center for Cancer Research, Massachusetts General Hospital, Charlestown, Massachusetts, USA; [9] Center for Computational and Integrative Biology, Massachusetts General Hospital, Charlestown, Massachusetts, USA; [10] Department of Pathology, Harvard Medical School, Boston, Massachusetts, USA

*Molecular Neurodegeneration* 2019, **14(Suppl 1):**POSTER

**Correspondence:** E Konstantinidis

**Background**

Mutations in the amyloid precursor protein (APP), presenilin 1 (PSEN1) and presenilin 2 (PSEN2) genes are known genetic causes of familial Alzheimer's disease (FAD) [1]. To date, twenty six pathogenic APP mutations have been described alongside 300 PSEN1 and 18 PSEN2 mutations. Carriers of the Swedish APP mutation develop a dominant form of early-onset AD as a consequence of increased Aβ formation while majority of the PSEN1 and PSEN2 mutations result in increased generation of the more aggregation prone Aβ form with 42 amino acids (Aβ42). Such alterations are evident not only in the brain but also in fibroblasts [2, 3].

**Methods**

Human fibroblasts from AD patients carrying the APPSwe or PSEN1 M146L mutations were transfected with a plasmid expressing guide-RNA and Cas9 protein [4]. Enzyme- linked immunosorbent assay was used to measure Aβ40 and Aβ42 levels while editing efficiency was assessed through next generation sequencing (NGS). Furthermore, adeno- associated virus (AAV) vectors were used for direct injection of the APPSwe guideRNA and Cas9 into the hippocampus of Tg2576 mice.

**Results**

We were able to show effective disruption of the mutated APPSwe allele in human AD patient fibroblasts and Tg2576 mice brains using the CRISPR/Cas9 system. There was statistically significant reduction in Aβ40 and Aβ42 levels in the edited fibroblasts while NGS showed robust indel formation in the APPSwe allele both in edited fibroblasts and hippocampus of Tg2576 mice. The same ex vivo procedure is currently ongoing for the PSEN1 M146L mutation while further in vivo experiments will be conducted on knock-in APPSwe mice.

**Discussion/Conclusions**

Effective disruption of the APPSwe allele is possible, both in vivo and in vitro, through the CRISPR/Cas9 system. We believe that the CRISPR/Cas9 system has the potential to be developed as a tool for future gene therapy against AD caused by certain APP and PSEN1 point mutations associated with increased Aβ.

## P2-H11 AdipoRon, A ADIPONECTIN RECEPTOR AGONIST, RESCUES COGNITIVE DEFICITS, REDUCED ALZHEIMER'S DISEASE PATHOLOGIES AND PREVENTED NEURODEGENERATION BY ENHANCING NEURONAL INSULIN SENSITIVITY IN 5XFAD MICE

Roy Chun-Laam NG[1], Myriam Lambert[1], Jian Min[1], Oscar Ka- Fai Ma[1], GJ. Zhou[2], Siva-Sundara Kumar Durairajan[3], KM Leung[2], Sookja Kim Chung[4], Koon-Ho Chan[1]

[1]Department of Medicine, LKS Faculty of Medicine, the University of Hong Kong, Hong Kong; [2]School of Biological Sciences, the University of Hong Kong, Hong Kong; [3]School of Chinese Medicine, Hong Kong Baptist University, Hong Kong; [4]School of Biomedical Sciences, LKS Faculty of Medicine, the University of Hong Kong, Hong Kong

*Molecular Neurodegeneration* 2019, **14(Suppl 1):**POSTER

**Correspondence:** Roy Chun-Laam NG

**Background**

Adiponectin (APN) reduces with age and has been implicated in Alzheimer's disease (AD). It is hypothesized that reduction of adiponectin impairs cerebral insulin signaling leading to β-amyloid accumulation, Tau hyperphosphorylation and glia activation that are the major events in AD [1, 2]. Enhancing insulin signaling can improve memory functions in AD patients [3]. Adiponectin possesses insulin sensitizing, anti-oxidative and anti-inflammatory effects that may treat metabolic disease. Recently, novel adiponectin agonist, AdipoRon, has shown insulin sensitizing and anti-diabetic effects in mice. Whether this molecule can cross the blood-brain-barrier (BBB) and can protect from neurodegeneration remains unknown.

**Methods**

To test this hypothesis, transgenic mice (5XFAD) that carried familial APP and PS mutations were fed with AdipoRon (50mg/kg of mice) by oral gavage for 3 months. We also generated an adiponectin deficient-AD mice (5XFAD;APN-/-) by crossing 5XFAD with APN-/- mice. Cognitive functions of these mice were evaluated by Morris-Water-Maze, Open-Field and Novel Object Recognition test. Brains and other tissues were collected for immunostaining analysis

**Results**

We found that AdipoRon penetrated BBB and reached the peak concentration 2 hour after oral administration. Oral gavage of AdipoRon improved memory functions and reduced anxiety levels in 5XFAD mice. These mice also had increased synaptic proteins levels, increased dendritic spine density and reduced number of dystrophic axons. Moreover, AdipoRon reduced Aβ accumulation, reactive microglia and dystrophic astrocytes in 5XFAD mice. AdipoRon enhanced insulin sensitivity in mouse hippocampal HT-22 cells by promoting AMPK activation. Oral gavage of AdipoRon also enhanced hippocampal insulin sensitivity in 5XFAD mice. In contrast, 5XFAD;APN-/- mice exacerbated spatial memory functions with increased Aβ accumulation in the cerebral blood vessels and had hippocampal insulin resistance upon stereotaxic injection of insulin.

**Conclusions**

Together, our results suggest that reduced APN level worsen AD pathologies and AdipoRon can enhance neuronal insulin sensitivity, inhibit inflammation and reverse AD-related pathologies and cognitive functions.

1. Ng RC-L, Cheng OY, Kwan JSC, Ho PWL, Cheng KKY, Yeung PKK, Zhou LL, Hoo RLC, Chung SK, XuA, Lam KSL, Chan KH:Chronic adiponectin deficiency leads to Alzheimer's disease-like cognitive impairments through AMPK inactivation and cerebral insulin resistance in aged mice. Mol Neurodegener2016, 11:1–16.
2. Waragai M, Adame A, Trinh I, Sekiyama K, Takamatsu Y, Une K, Masliah E, Hashimoto M:Possible Involvement of Adiponectin, the Anti-Diabetes Molecule, in the Pathogenesis of Alzheimer's Disease. J Alzheimer's Dis2016, 52:1453–1459.
3. Watson GS, Claxton A, Arbuckle M, Leverenz J, Cross D, Gerton B:Intranasal Insulin Therapy for Alzheimer Disease and Amnestic Mild Cognitive Impairment. Arch Neurol2012, 69:29–38.

## P2-H13 A NOVEL PLATFORM TECHNOLOGY FOR THE PRODUCTION OF CONFORMATION-SPECIFIC ANTIBODIES AGAINST AMYLOID PROTOFIBRIL SPECIES

W Vallen Graham[1], Alessandra Bonito-Oliva[1], Sophia Schedin-Weiss[2], Ronald J. Parchem[3], Caroline Graff[2], Vladana Vukojevic[4], Lars O. Tjernberg[2], Lars Terenius[4], Bengt Winblad[2], Thomas P. Sakmar[1, 2]

[1]Laboratory of Chemical Biology & Signal Transduction, The Rockefeller University, New York, NY 10065; [2] Department of Neurobiology, Care Sciences and Society, Center for Alzheimer Research, Division of Neurogeriatrics, Karolinska Institutet, 14157 Huddinge, Sweden; [3] Department of Neuroscience, Stem Cells and Regenerative Medicine Center, Baylor College of Medicine, Houston, TX, US; [4] Department of Clinical Neuroscience, Center for Molecular Medicine, Karolinska Institutet, 17176 Stockholm, Sweden

*Molecular Neurodegeneration* 2019, **14(Suppl 1):**POSTER

**Correspondence:** W Vallen Graham

**Background**

The precise role of amyloid in the initiation and progression of Alzheimer's disease (AD) remains controversial. Recently, there have been promising results from conformation-specific amyloid beta (Aβ)- targeting biologics in clinical trials, thereby reigniting debate about the amyloid hypothesis [1]. Discovering conformation-specific monoclonal antibodies (mAbs) that target soluble intermediates of amyloid is challenging due to the inherent transient nature of the misfolded complex. We have engineered a chaperone-like amyloid binding protein (CLABP) that enables the stabilization of protofibrils. CLABP-stabilized protofibrils can be used as immunogen to accelerate the discovery of high-affinity anti-protofibril mAbs [2].

**Methods**

We employed an engineered CLABP, NUCB1, to stabilize protofibrils for use as immunogen in mice. Through primary and secondary screenings we selected a pool of mAbs with the exceptional capability to bind specifically protofibril species, while showing minimal activity against the monomeric protein. The mAbs selected after initial screens were further characterized in several in vitro assays and in immunohistological studies of an AD mouse model as well as brain tissue from an AD patient.

**Results**

We show that an immunization campaign with NUCB1-protofibril complex produces mAbs that specifically target amyloid protofibrils, inhibiting their further aggregation. In line with conformation-specific binding, the mAbs appear to react with an intracellular antigen in diseased tissue, but only weakly with amyloid plaques. We hypothesize that the mAbs we describe here recognize a secondary or quaternary structural epitope that is common to multiple amyloid protofibrils.

**Discussion**

The amyloid protofibril stabilization method that we developed is valid for amyloid from multiple sources and can be applied to the preparation of mAbs against multiple types of amyloid protofibrils.

**Conclusions**

We report a novel method to create anti- protofibril mAbs that are conformationally-sensitive. The anti-protofibril mAbs we prepared have utility as research tools to study amyloid protofibril formation and structure, and may also have potential as diagnostic and therapeutic leads.

## P2-H15 EVALUATING CALPAIN-1 AND CATHEPSIN B AS THERAPEUTIC TARGETS FOR NEURODEGENERATION

Rachel C. Knopp, Manel Ben Aissa, Sue Lee, Ammar Jastaniah, Ragda Izar, Gregory R.J. Thatcher
Department of Medicinal Chemistry and Pharmacognosy, University of Illinois at Chicago, Chicago, IL, 60612
*Molecular Neurodegeneration* 2019, **14(Suppl 1):**POSTER

**Correspondence:** Rachel C. Knopp

**Background**

Neurodegeneration, an umbrella term for disorders with irreversible neuronal loss, is a significant global health concern. Therapeutic development has been hindered by several roadblocks, one of which is lack of in-depth exploration of potential targets. This research focuses on Calpain 1 (CAPN1), a calcium-dependent cysteine protease implicated in pathogenesis of AD, traumatic brain injury (TBI), and ischemic stroke. Prolonged CAPN1 over-activation indirectly permeabilizes lysosomes, leading to release of Cathepsin B (CTSB), a lysosomal cysteine-protease implicated in neurodegeneration. Several reports propose CAPN1 and CTSB as therapeutic targets in AD and TBI, but fail to identify efficacious strategies for inhibition. We hypothesize that dual CAPN1/CTSB inhibition affords superior neuroprotection over selective inhibition.

**Methods**

Inhibition profiles (potency, selectivity, reversibility) of small molecules were characterized through enzymatic screening assays. Subsequently, neuroprotection was characterized in SH-SY5Y cells using Oxygen Glucose Deprivation (OGD), an in vitro model simulating ischemia- reperfusion injury in stroke. Additional in vitro models induced by chemical insult were utilized to monitor CAPN1/CTSB substrates with roles in neuroplasticity/neurodegeneration via immunoblots.

**Results**

We have established inhibition and neuroprotective profiles of selective vs. dual inhibitors. All inhibitors were differentially neuroprotective against OGD-induced cell death in different treatment paradigms (pre-treatment, ischemia and reperfusion). Monitoring spectrin breakdown products (CAPN1-specific) identified different pathways of neuronal death with varying neuroinsults.

**Discussion**

After establishing the selectivity of inhibitors for CAPN1 and CTSB, monitoring of peptide substrate proteolysis confirmed inhibitory effects in neuronal cultures. CAPN1 was found to be a highly dynamic protease, with time dependent hyper-activation following neuro-insult.

**Conclusions**

Neuroprotective profiles suggest the strength of CAPN1/CTSB inhibitor strategies vary based on treatment paradigms. We have just completed testing this strategy in a murine model of mild TBI which, following behavioral and biochemical analysis, will allow us to distinguish the impact of these strategies in vivo.

## P2-H16 NEUROBEHAVIOUR EFFECT OF COCONUT OIL IN SCOPOLAMINE INDUCED AMNESIA IN RATS

Kiran Chanabasappa Nilugal[1], Janice Ng Gin Xiang[2], Santosh fattepur[1], Mohd Fadli Asmani[1], Ibrahim Abdullah[1]
[1]School of pharmacy Management and Science University Shah Alam Selangor Malaysia; [2]School of Pharmacy La Trobe University Australia
*Molecular Neurodegeneration* 2019, **14(Suppl 1):**POSTER

**Correspondence:** Kiran Chanabasappa Nilugal

**Background**

Memory loss has been the major symptom of the progressive neurodegenerative Alzheimer's disease (AD) and has affected AD patients the most. Scopolamine act as a cholinergic antagonist known to produce amnesia effect to mimic the cognitive impairment found in AD. Medicinal plants with antioxidant activities have been traditionally used to slow down the degeneration of neurons. The present study aimed to investigate the potential of extra virgin coconut oil (EVCO) to attenuate scopolamine (0.6 mg/kg, i.p.) induced amnesia and histopathological changes in rat hippocampus and cerebral cortex.

**Methods**

This study was conducted on 20 Sprague-Dawley (100-150g) female rats and the rats were divided into 5 groups (6 each). Group 1 served as negative control given normal saline (1 ml/day, p.o.) for 8 days. Group 2 served as positive control received scopolamine (0.6 mg/kg i.p.) on the 8th day. Group 3 treated with piracetam (200 mg/kg/day, p.o.), group 4 with EVCO (0.25 ml/kg/day, p.o.) and group 5 with EVCO (0.5 ml/kg/day, p.o.) for 8 consecutive days, then followed by scopolamine (0.6 mg/kg, i.p.) administration on the 8th day. Behavioral stress tests such as T-maze and Rota rod tests were carried out. At the end of the experiments, rats' brains were dissected and divided sagitally into two portions, the first was homogenized for determination of acetylcholinesterase activity and the second was used for histopathologic examination.

**Results**

This study indicated that EVCO when used for the treatment of scopolamine-induced amnesia produced increased time on the Rota rod and a reduction of duration of rats to reach food in the T-maze test. EVCO also showed a slight reduction of AChE activity and the histopathological findings showed the neurons appear more or less like normal ones but with more dark spots.

**Conclusions**

This study revealed that the treatment of amnesia-induced rats with EVCO significantly ameliorates the cognitive impairment of AD in rats.

P2-H17 TARGETED BRICHOS DOMAIN DELIVERY TO THE BRAIN USING FOCUSED ULTRASOUND-INDUCED BLOOD-BRAIN BARRIER OPENING

Lorena Galan-Acosta[1], Carlos Sierra [2], Jenny Presto[1], Per Nilsson[1], Elisa E. Konofagou[2,3], Jan Johansson [1]
[1]Department of Neurobiology, Care Sciences and Society (NVS), Division of Neurogeriatrics, Karolinska Institutet, 141 57 Huddinge, Sweden; [2]Ultrasound and Elasticity Imaging Laboratory, Department of Biomedical Engineering, Columbia University, New York, New York, USA; [3]Department of Radiology, Columbia University, New York, New York, USA
*Molecular Neurodegeneration* 2019, **14(Suppl 1):**POSTER

**Correspondence:** Lorena Galan-Acosta

**Background**

The BRICHOS domain is encoded in more than 10 human genes associated with cancer, dementia (Bri2/ITM2b) and amyloid lung disease (proSP-C) [1]. Studies have shown that overexpression of proSP-C or Bri2 BRICHOS delays fibril formation and toxicity of amyloid-β peptide (Aβ) in vitro and in vivo, which plays a central role in the development of Alzheimer´s disease (AD) [2-4]. BRICHOS domain thus has the potential for treating this disease. After peripheral administration, a limited amount of Bri2 BRICHOS was detected in the brain parenchyma of wild-type mice, suggesting passage over the blood-brain barrier (BBB) [5]. Our main objective herein is to increase the BRICHOS domain delivery rate using focused ultrasound (FUS) combined with microbubbles, which has been proven to open the BBB locally, transiently and non invasively [6].

**Methods**

A FUS transducer was used to target the left hippocampus of the mouse brain in vivo in the presence of intravenous lipid microbubbles and BRICHOS domain. Mice were kept for 2 hours after sonication to allow the BRICHOS domain to diffuse into the parenchyma, and then sacrificed for assessing the delivery by ex vivo immunohistochemistry (IHC) for proSP-C or Bri2 BRICHOS. The neuronal marker NeuN was used for assaying possible neuronal BRICHOS uptake. BBB opening was confirmed in vivo by magnetic resonance imaging. The overall brain histology was evaluated for microscopic damage.

**Results**

Successfully targeted brain BRICHOS domain delivery was achieved in 6 out of 10 cases. Notably, IHC showed selective uptake of BRICHOS by a specific subset of neurons in dentate gyrus in the FUS targeted hippocampus section. Microhemorrhages were observed only in two cases.

**Conclusions**

This study indicates that FUS is a safe methodology for targeted brain BRICHOS domain delivery, which can potentially be used in the analyses of BRICHOS treatment on AD pathology.

P2-I01 A VALIDATED ANTIBODY PANEL FOR THE CHARACTERIZATION OF TAU POST-TRANSLATIONAL MODIFICATIONS

**Correspondence:** Ebru Ercan

**Background**

Tau is a microtubule-binding protein, which is subject to various post-translational modifications (PTMs) including phosphorylation, methylation, acetylation, glycosylation, nitration, sumoylation and truncation. Aberrant PTMs such as hyperphosphorylation result in tau aggregation and the formation of neurofibrillary tangles, which are a hallmark of Alzheimer's disease (AD) [1]. In order to study the importance of PTMs on tau function, antibodies raised against specific modification sites are widely used. However, quality control of these antibodies is lacking and their specificity for particular modifications is often unclear.

**Methods**

In this study, we first designed an online tool called 'TauPTM', which enables the visualization of PTMs and their interactions on human tau. Using TauPTM, we next searched for commercially available antibodies against tau PTMs and characterized their specificity by peptide array, immunoblotting, electrochemiluminescence ELISA and immunofluorescence technologies.

**Results**

We demonstrate that commercially available antibodies can show a significant lack of specificity, and PTM-specific antibodies in particular often recognize non- modified versions of the protein. In addition, detection may be hindered by other PTMs in close vicinity, complicating the interpretation of results. Finally, we compiled a panel of specific antibodies and show that they are useful to detect PTM-modified endogenous tau in hiPSC-derived neurons and mouse brains [2].

**Discussion**

Here we show that not all tau PTM-antibodies tested detect their specific modification site, and/or detect non-modified peptide in peptide array experiments. These findings are very concerning, since they will lead to the false- positive detection of PTMs and subsequent false conclusions drawn from experiments using these tools.

**Conclusions**

This study has created a platform to reliably and robustly detect changes in localization and abundance of post-translationally modified tau in health and disease. A web-based version of TauPTM is fully available at http://www.tauptm.org [2].

## P2-I02 SORTILIN MODULATES HUMAN TAU PROPAGATION IN THE BRAINS OF TRANSGENIC MICE

Noah R. Johnson[1], Carlo Condello[1,2], Shenheng Guan[3], Abby Oehler[1], Julia Becker[1], Marta Gavidia[1], George A. Carlson[1,2], Kurt Giles[1,2], Stanley B. Prusiner[1,2,4]
[1]Institute for Neurodegenerative Diseases, Weill Institute for Neurosciences, University of California, San Francisco, CA, USA; [2]Department of Neurology, University of California, San Francisco, CA, USA; [3]Department of Pharmaceutical Chemistry and Mass Spectrometry Facility, University of California, San Francisco, CA, USA; [4]Department of Biochemistry and Biophysics, University of California, San Francisco, CA, USA
*Molecular Neurodegeneration* 2019, **14(Suppl 1):**POSTER

**Correspondence:** Noah R. Johnson

**Background**

Misfolding of tau proteins into prions and their propagation along neural circuits are thought to result in neurodegeneration causing Alzheimer's disease (AD), progressive supranuclear palsy, and other tauopathies. Little is known about the molecular processes mediating tau prion replication and spreading in different brain regions. Sortilin is a type-1 transmembrane protein that serves as a vacuolar protein sorting receptor and has pleiotropic functions in neuronal protein trafficking and viability [1]. Stimulated by a recent report that sortilin mediates lysosomal degradation of PrP [2], and its role in amyloid-β [3] and TDP-43 proteinopathies [4], we investigated its involvement with tau. **Methods**

An in vitro cell bioassay for self-templating tau species [5] was used to measure tau prion levels in the brains of transgenic mice expressing human tau with the P301S mutation [6].

**Results**

Despite similar tau protein expression in the mouse forebrain, human tau prions selectively accumulated in hindbrain regions of P301S transgenic mice [7]. The spatiotemporal appearance of tau prion species preceded tau pathology and did not correlate with tau post-translational modifications. By examining affected and spared brain regions, sortilin was found to inhibit tau prion propagation, an effect that could be blocked by sortilin antibodies. Sortilin expression was observed to be higher in the mouse forebrain compared to the hindbrain.

**Discussion**

These findings argue that mouse hindbrain neurons are more susceptible to tauopathy, which may be analogous to subcortical nuclei in the AD brain where the earliest tau pathology is observed [8]. Sortilin appears to be one protein contributing to the regional vulnerability. Therefore, sortilin-mediated lysosomal degradation may be an important mechanism underlying tau proteostasis in human tauopathies.

**Conclusions**

These findings provide evidence for selective vulnerability in mice, thus affording a model for identification of additional molecules that could mitigate the levels of tau prions in human tauopathies.

## P2-I03 OVEREXPRESSION OF LOW-DENSITY LIPOPROTEIN RECEPTOR ATTENUATES TAU-MEDIATED NEURODEGENERATION AND MARKEDLY REDUCES TAU PHOSPHORYLATION IN A TAUOPATHY MOUSE MODEL

Yang Shi[1], Christina Ising[2], Kaoru Yamada[3], Kairuo Wang[1], Floy Stewart[1], Nathan Scott[1], Javier Remolina[1], Rosa Hoyle[1], Hong Jiang[1], David M. Holtzman[1]
[1]Department of Neurology, Hope Center for Neurological Disorders, Charles F. and Joanne Knight Alzheimer's Disease Research Center, Washington University School of Medicine, St. Louis, Missouri, USA; [2]German Center for Neurodegenerative Diseases (DZNE), Bonn, Germany; [3]Department of Neuropathology, Graduate School of Medicine, The University of Tokyo, Tokyo, Japan
*Molecular Neurodegeneration* 2019, **14(Suppl 1):**POSTER

**Correspondence:** Yang Shi

**Background**

APOE is the strongest genetic risk factor for late- onset Alzheimer disease. While ApoE strongly influences amyloid-β (Aβ) deposition in the brain[1, 2], recent findings reveal an Aβ-independent role of ApoE in regulating tau- mediated neurodegeneration[3]. ApoE ablation in P301S tau transgenic mice markedly reduces tau-associated brain atrophy and neuroinflammation, and shifts tau pathology towards an early-disease pattern[3]. Hence, targeting ApoE may be an efficient therapeutic approach to ameliorate tau- mediated neurodegeneration. Low density lipoprotein receptor (LDLR) is a major receptor regulating ApoE metabolism. Modulating LDLR levels may constitute an effective approach to target ApoE.

**Methods**

To lower ApoE levels, we crossed LDLR transgenic mice overexpressing LDLR >10-fold [4] to P301S mice, and compared tau pathology and neurodegeneration between LDLR+ and LDLR- P301S mice at 9 months of age. In a parallel cohort, we generated P301S mice with two alleles, one allele, or no ApoE for comparison.

**Results**

LDLR overexpression reduced brain ApoE levels by ~90% without altering soluble or insoluble tau levels. However, overexpressed LDLR markedly reduced RAB- and RIPA-soluble phospho-tau (p-tau) levels at 9months of age and shifted tau pathology towards early-disease patterns. Notably, LDLR+P301S mice exhibit significantly reduced brain atrophy and increased brain weight compared to LDLR-P301S mice. ApoE level strongly correlates with ptau levels in all fractions, and specifically correlates with less soluble tau and higher insoluble tau. Genetic ablation of one or two alleles of ApoE in P301S mice resulted in a similar ptau reduction in RAB and RIPA fractions.

**Discussion**

ApoE appears to play an essential role in regulating ptau accumulation, which may further affect neurodegeneration. LDLR overexpression attenuates tau- mediated neurodegeneration likely by impeding ptau generation via lowering ApoE levels.

**Conclusion**

Increasing LDLR levels may represent an effective therapeutic strategy for AD/tauopathy treatment.

**Acknowledgement**
This study is funded by NS090934, AG047644, the JPB Foundation, and the Cure Alzheimer's Fund

## P2-I04 AN ADENO-ASSOCIATED VIRUS TOOLKIT TO DETERMINE MECHANISMS UNDERLYING TAU DEGRADATION

Cara L. Croft[1,2,3], Kaitlyn Rewis[1,2,3], Pedro E. Cruz[1,2,3], Marshall S. Goodwin[1,2,3], Daniel H. Ryu[1,2,3], Benoit I. Giasson[1,2,3], Todd E. Golde[1,2,3]
[1]Center for Translational Research in Neurodegenerative Disease; [2]Department of Neuroscience; [3]McKnight Brain Institute, University of Florida, Gainesville, Florida, USA
*Molecular Neurodegeneration* 2019, **14(Suppl 1):**POSTER

**Correspondence:** Cara L. Croft

**Background**

Tau inclusions comprised of aggregated, post- translationally modified tau species are one of the two pathological hallmarks of Alzheimer's disease (AD). A dysfunction of canonical protein degradation pathways has been implicated in the tauopathies such as AD. Reengaging these pathways to degrade accumulating tau could be therapeutically viable, however, the role of the autophagy- lysosome system and the ubiquitin-proteasome system and their clearance of physiological and pathological tau species still remains to be fully determined.

**Methods**

We have developed several recombinant adeno-associated viral (rAAV) vectors that enable the activation and inhibition of lysosomal and proteosomal pathways. Using HEK293T cell models and organotypic mouse brain slice cultures (BSCs) we explore the effects of enhancing and reducing lysosomal or proteosomal activity on the accumulation of wild-type or mutated MAPT.

**Results**

By using rAAVs to overexpress the herpes simplex virus type 1 (HSV-1)-encoded neurovirulence protein ICP34.5 to inhibit autophagy and the essential autophagy protein Beclin-1 to enhance autophagy we are able to determine the effects of the autophagy-lysosome system on the clearance of tau in our cell and BSC models of tauopathy. In addition, we have developed methods to drive intracellular expression of antibody fragments known as intrabodies to target tau and have fused these intrabodies to functional domains which target tau for increased proteasomal or lysosomal degradation.

**Discussion**

By overexpressing biological inhibitors and activators of key protein degradation pathways we can dissect mechanisms determining the clearance of protein aggregates which can lead to neurodegenerative proteinopathies such as the accumulation of tau in tauopathies. This rAAV toolkit provides a robust and reliable system to explore tau degradation in biological systems.

**Conclusions**

Our rAAV toolkit in combination with our novel cell and slice culture models of tauopathy can enable us to understand the mechanisms underlying autophagy-lysosome and ubiquitin-proteasome dysfunction in the tauopathies.

## P2-I05 IN VITRO DISEASE MODELING OF TAU R406W MUTATION USING PATIENT-DERIVED iPSCs

Mari Nakamura[1,2], Hirotaka Watanabe[1], Seiji Shiozawa[1], Sumihiro Maeda[1], Sho Yoshimatsu[1], Shinichi Hisanaga[3], Naruhiko Sahara[4], Taeko Kimura[4], Tomohiro Miyasaka[5], Akihiko Takashima[6], Takeshi Ikeuchi[7], Hideyuki Okano[1]
[1]Department of Physiology, Keio University School of Medicine, Tokyo, Japan; [2]Department of Biomedical Chemistry, University of Tokyo Graduate School of Medicine, Tokyo, Japan; [3]Department of Biological Science, Tokyo Metropolitan University School of Science and Engineering, Tokyo, Japan; [4] National Institute of Radiological Sciences, Chiba, Japan; [5]Department of Neuropathology, Doshisha University Faculty of Medical and Life Sciences, Kyoto, Japan; [6]Department of Neurobiology, Gakushuin University Graduate School of Science, Tokyo, Japan; [7]Brain Research Institute, Niigata University, Niigata, Japan
*Molecular Neurodegeneration* 2019, **14(Suppl 1):**POSTER

**Correspondence:** Mari Nakamura

**Background**

Frontotemporal dementia and Parkinsonism linked to chromosome 17 (FTDP-17) is a neurodegenerative disease caused by mutations in the microtubule-associated protein tau (MAPT) gene, which encodes the tau protein. Among the MAPT mutations, the R406W mutation is a unique missense mutation whose patients have been reported to exhibit AD-like phenotypes, rather than the more typical FTDP symptoms. To date, there is no treatment known to be effective for FTDP-17, including the R406W mutation. The objective of this study is to establish a suitable model for studying the abnormalities induced by R406W tau, as a basis for drug screening.

**Methods**

Induced pluripotent stem cells (iPSCs) were generated from patients harboring the MAPT R406W mutation and manipulated with a genome-editing technique to create isogenic lines. Using iPSC-derived neurons, phosphorylation and proteolysis of tau was investigated with Phos-Tag SDS-PAGE or western blotting. Furthermore, localization of tau and neurite morphology were examined using a high-content imaging microscope.

**Results**

iPSC lines from patients heterozygous for the MAPT R406W mutation were established. These lines were gene- edited to establish isogenic lines with the mutation corrected, or homozygous for the mutation. iPSCs were then induced into forebrain cortical neurons, with more than 85% neuronal purity. In these neurons, R406W tau exhibited abnormal phosphorylation and increased fragmentation. Furthermore, increased percentage of R406W tau was mislocalized to the dendrites, which correlated with increased axonal dystrophy.

**Discussion**

Abnormal phosphorylation and fragmentation of R406W tau could be triggers of further neurodegeneration, with tau mislocalization and axonal dystrophy being some of the early pathological features. The next step is to identify specific phosphorylation sites and proteases responsible for the increased fragmentation, and to elucidate the molecular mechanisms leading to axonal degeneration.

**Conclusions**

An iPSC-derived neuronal model successfully recapitulated what are thought to be early pathological hallmarks caused by R406W tau.

P2-I09 SPPL2b: A NOVEL PROTEIN RELATED TO TAU PATHOLOGY IN ALZHEIMER'S DISEASE?

M Del Campo[1], L Boonkamp[1], VI Wiersma[2], A Nolle[2], W Scheper[2], AB Smit[3], RE Van Kesteren[3], R Fluhrer[4,5], B Schröder[6], J Hoozemans[7], CE Teunissen[1]
[1]Department of Clinical Chemistry, VU University Medical Center, the Netherlands; [2]Department of Clinical genetics and Alzheimer Center, VU University Medical Center and department of Functional Genomics, Molecular Neurodegeneration, Center for Neurogenomics and Cognitive Research (CNCR), VU University, The Netherlands; [3]Department of Molecular and Cellular Neurobiology, Center for Neurogenomics and Cognitive Research (CNCR), VU University, The Netherlands; [4]DZNE – German Center for Neurodegenerative Diseases, Munich, Germany; [5] Institute for Metabolic Biochemistry, Ludwig-Maximilians University Munich, Munich, Germany; [6] Institute of biochemistry, CAU Kiel University, Germany; [7]Department of pathology, VU University Medical Center, the Netherlands
*Molecular Neurodegeneration* 2019, **14(Suppl 1):**POSTER

**Correspondence:** M Del Campo

**Background**

Alzheimer's disease (AD) is characterized by the presence of amyloid beta (Aβ) plaques and neurofibrillary tangles (NFT). SPPL2b is a transmembrane protease involved in the processing of ITM2B (BRI2) and TNFα, substrates involved in Aβ plaque and NFT formation. Previously, we have shown that levels of SPPL2b were 10-fold increased in early stages of Alzheimer's disease (Braak III-IV)[1], which was to a lower extent reflected in the cerebrospinal fluid (CSF) of AD cases. We also observed co-localization of SPPL2b with phosphorylated tau. Here, we aimed to investigate the functional relation between SPPL2b and tau in cell and animal models.

**Materials and Methods**

SPPL2b was quantified in hippocampus tissue of mice models driven by Aβ pathology (APP-PS1, n=5) and tau pathology (P301S, n=5) as well as in HEK293 cells overexpressing mutated Tau (P301S-Tau; n = 4). Corresponding wild-type mice and control cells were also included (n = 4-5/group).

**Results**

SPPL2b was 10–fold increased in the hippocampus of the mice overexpressing tau P301S ($p < 0.0001$) compared to wild-type. In contrast, no changes were observed in the hippocampus of APP-PS1 mice. Human cell lines overexpressing the mutated tau form also showed a strong increase in SPPL2b levels ($p < 0.0001$) compared to controls.

**Discussion**

Our results from mice and cell models suggest that the strong SPPL2b changes previously observed in AD post-mortem tissue are likely driven by tau pathology and not by Aβ aggregates.

**Conclusions**

Considering that SPPL2b is a novel Tau binding protein with unknown physiological function, it will be important to unravel whether the strong SPPL2b changes observed in early stages of AD aid to prevent tau pathology (i.e. via lysosomal degradation) or contribute to the development of AD (i.e. neurotoxicity, tau spreading).

P2-J01 STRUCTURAL INSIGHTS INTO APOLIPOPROTEIN E ISOFORMS: UNDERSTANDING THE MAJOR RISK FACTOR FOR LATE ONSET ALZHEIMER'S DISEASE

**Correspondence:** Ana-Caroline Raulin

**Background**

Alzheimer's disease (AD) is the leading cause of dementia in the elderly, with age as a primary risk factor. Environmental and genetic factors also modulate the onset of sporadic, late-onset cases of the disease (LOAD). With respect to genetics, the APOE genotype, and more particularly the ε4 variant of the gene, has been identified as a major risk factor [1]. Very little is known about the ε2 variant of APOE, which has been shown to be protective against the onset of the disease. Understanding why a factor is protective is as important as understanding how a factor may present a risk as this information may lead to the discovery of new therapeutic targets.

**Methods**

The differences between human recombinant ApolipoproteinE (ApoE) isoforms were investigated at the structural level using a range of biophysical techniques (including analytical ultracentrifugation, circular dichroism spectroscopy, fluorescence spectroscopy and transmission electron microscopy (TEM)) to characterise their conformation and stability as well as their self-assembly properties.

**Results**

The three isoforms adopted a tetrameric conformation in physiological buffer and displayed a similar, high α-helix content in solution. Unfolding studies did not reveal significant differences between ApoE3 and E4. However, the three isoforms differed in terms of self- assembly properties, with ApoE4 having a higher propensity to form fibrillary aggregates than ApoE2 and ApoE3 as shown both by thioflavin-T fluorescence and TEM.

**Discussion**

One of the main hypotheses behind the onset of AD revolves around protein misfolding and aggregation, hence the observation that ApoE4, one of the major risk factor for LOAD, is prone to fibril formation in vitro is of great interest.

**Conclusions**

Studying the structure of ApoE has highlighted major differences between the three isoforms, mainly in terms of aggregation propensity which is interesting in the context of protein misfolding and aggregation in AD [2].

## P2-J02 CELL AUTONOMOUS EFFECTS OF APOE ε4/ε4 ON HUMAN iPSC-DERIVED ASTROCYTES AND MICROGLIA

Julia TCW[1], Sarah E. Bertelsen[1], Shuang A. Liang[2], Lu Qian[1], Cristhian Fimbres[2], Raja Jain[2], Elizabeth J. Sikora[1], Wayne W. Poon[2], Alison M. Goate[1]
[1]Department of Neuroscience, Ronald M. Loeb Center for Alzheimer's Disease, Icahn School of Medicine at Mount Sinai, New York, NY, USA; [2]Institute for Memory Impairments and Neurological Disorders, University of California Irvine, Irvine, CA, USA
*Molecular Neurodegeneration* 2019, **14(Suppl 1):**POSTER

**Correspondence:** Julia TCW

**Background**
Apolipoprotein E (APOE) is the most significant risk gene for late-onset Alzheimer's disease (AD). APOE ε4/ε4 homozygosity increases AD risk by >14-fold [1]. Although an association between the APOE ε4 allele and increased AD risk is well-established, the mechanisms underlying this genetic risk on particular brain cell types is elusive. We hypothesized that the APOE ε4/ε4 genotype contributes to disease risk through cell autonomous mechanisms in glia.

**Methods**
We have differentiated astrocytes [2], microglia [3], cortical neurons [4] and brain microvascular endothelial cells [5] from human induced pluripotent stem cells (iPSC) derived from non-isogenic and isogenic cohort of cells selected based on APOE genotype. RNAseq was performed, and differentially expressed genes of APOE ε4/ε4 compared to ε3/ε3 were analyzed in each cell type. We executed Gene Set Enrichment Analysis (GSEA) to identify the top significantly enriched genes, followed by Ingenuity Pathway Analysis (IPA).

**Results**
When APOE ε4/ε4 transcriptomes were compared to ε3/ε3 by GSEA the most significantly enriched pathways are cholesterol biosynthesis (positive enrichment) in astrocytes and lysosomal pathways (negative) in microglia. Overlapping pathway analysis (FDR<0.05) of both cell types showed positive enrichment of cholesterol biosynthesis. Functional network analysis by IPA incorporated with GSEA showed that APOE ε4/ε4 cells significantly upregulate cholesterol biosynthesis and lipid metabolism regulatory networks. Consistently, lysosomal pathways of microglia enriched in GSEA are associated with phagosome maturation and autophagic function, defects of which leads to increased lipid accumulation and decreased lipid catabolism.

**Discussion**
In addition to effects on Aβ APOE genotype appears to alter glial handling of lipids including those engulfed as a result of neurodegeneration.

**Conclusions**
Human CNS cell type based iPSC models allowed us to elucidate APOE ε4/ε4 cell autonomous effects; astrocytes and microglia of APOE ε4/ε4 compared to ε3/ε3 have deficits in lipid metabolism, leading to an increased cholesterol accumulation.

**References**
1. Genin, E., et al., APOE and Alzheimer disease: a major gene with semi-dominant inheritance. Mol Psychiatry, 2011. 16: 903-907.
2. TCW, J., et al., An Efficient Platform for Astrocyte Differentiation from Human Induced Pluripotent Stem Cells. Stem Cell Reports, 2017. 9: 600-614.
3. Abud, E.M., et al., iPSC-Derived Human Microglia-like Cells to Study Neurological Diseases. Neuron, 2017. 94: 278-293.
4. Bardy, C., et al., Neuronal medium that supports basic synaptic functions and activity of human neurons in vitro. Proc Natl Acad Sci U S A, 2015. 112: E2725-2734.

## P2-K01 TELOMERASE ACTIVATING COMPOUNDS IN ALZHEIMER'S DISEASE MODEL

Natalie Baruch-Eliyahu[1], Vladislav Rud[1], Alex Braiman[1], Dan Mishmar[2], Esther Priel[1]
[1]Department of Microbiology, Immunology, and Genetics Ben-Gurion University of the Negev, Be'er Sheva, Isarel; [2]Department of Life Science Ben-Gurion University of the Negev, Be'er Sheva, Isarel
*Molecular Neurodegeneration* 2019, **14(Suppl 1):**POSTER

**Correspondence:** Vladislav Rud

In a previous study our lab demonstrated the protective effect of telomerase in the neurodegenerative disease ALS. Telomerase is a reverse transcriptase protein that is best known for telomere maintenance functions in dividing cells. 1It can also shuttle from the nucleus to the mitochondria upon oxidative stress, where it decreases levels of ROS, DNA damage, apoptosis, and complex I activity. 2 3 We showed that TERT protein is expressed in old mouse brain and that increasing its expression by pharmaceutical compounds, such as novel tri-aryl compounds designated AGS, which were developed in our lab, may protect brain cells from death. 4 It was found that AGS compounds increase telomerase expression in a time and dose-dependent manner and that they protect cells from oxidative stress in vivo and in vitro. 5 Previous studies have shown that the catalytic subunit of telomerase protects neurons from amyloid-beta toxicity, and its apoptosis inducing effects. 6 We hypothesize that the aforementioned protective effect can also be applied in other neurodegenerative diseases such as Alzheimer's disease (AD).

**Results**
Although the exact mechanism remains unknown, using both an immunofluorescent staining and a Real-Time PCR based assay it appears that AGS increased the expression of telomerase in primary neuronal cultures, and conferred a significant protective effect from Aβ-induced damages of neurons in primary cultures. In further Real-Time based analyses have shown an increase in expression of neuronal genes, such as Synaptophysin, GAP43, and NeuN which correlates with the increase in TERT expression. These results were also shown in mice. Further analysis is required to establish cause and effect. Preliminary results with immunofluorescent staining show that the presence of AB affects the size and number of apoptotic cells in the culture, while the addition of AGS mitigates the peptides negative effects. Although not-conclusive, these results, along with previous studies may imply a connection between TERT and prevention of neurodegenerative disorders, and the potential therapeutic effect of AGS in neurodegenerative diseases.

**References**
1. Giardini, M. A., Segatto, M., da Silva, M. S., Nunes, V. S. S. & Cano, M. I. Telomere and telomerase biology. Prog Mol Biol Transl Sci 125, 1–40 (2014).
2. Chiodi, I. & Mondello, C. Telomere-independent functions of telomerase in nuclei, cytoplasm, and mitochondria. Front Oncol 2, 133 (2012).
3. Zhou, J., Ding, D., Wang, M. & Cong, Y.-S. S. Telomerase reverse transcriptase in the regulation of gene expression. BMB Rep 47, 8–14 (2014).
4. Eitan, E., Tichon, A., Daniel, G. & Priel, E. Telomerase expression in adult and old mouse Purkinje neurons. Rejuvenation Res 15, 206–9 (2012).
5. Eitan, E. et al. Novel telomerase-increasing compound in mouse brain delays the onset of amyotrophic lateral sclerosis. EMBO Mol Med 4, 313–29 (2012).

6. Zhu, H., Fu, W. & Mattson, M. P. The catalytic subunit of telomerase protects neurons against amyloid beta-peptide- induced apoptosis. J. Neurochem. 75, 117–24 (2000).

## P2-K02 ACRYLAMIDE AFFECTS PROLIFERATION AND DIFFERENTIATION OF THE NEURONAL PROGENITOR CELL LINE C17.2 AND THE NEUROBLASTOMA CELL LINE SH-SY5Y

Kristina Attoff[1], Dimitra Kertika[1], Jessica Lundqvist[1], Stina Oredsson[2], Anna Forsby[1,3]
[1]Department of Neurochemistry, Stockholm University, Stockholm, Sweden, 2Department of Biology, Lund University, Lund, Sweden, 3Swedish Toxicology Science Research Center (Swetox), Karolinska Institute, Södertälje, Sweden
*Molecular Neurodegeneration* 2019, **14(Suppl 1):**POSTER

**Correspondence:** Kristina Attoff

**Background**

Acrylamide is a well-known neurotoxic compound and exposure occurs through food consumption and environmental pollutants. There is a risk for developmental neurotoxicity since acrylamide crosses the placenta. Apart from this, many neurodegenerative diseases are in fact believed to originate from neurodevelopmental disorders and share a common pathophysiological cascade involving oxidative stress and impaired hippocampal neurogenesis in mice [1].

**Methods**

The neural progenitor cell line C17.2 and the neuroblastoma cell line SH-SY5Y were used to study proliferation and differentiation in vitro as alerting indicators for developmental neurotoxicity.

**Results**

Acrylamide (10 fM and higher) attenuated the differentiation process in SH-SY5Y cells and neurite outgrowth was reduced at concentrations ≥10 pM. In the C17.2 cell line, 1 μM acrylamide significantly reduced the number of neurons and altered the ratio between the cell phenotypes. Ten micromolar of acrylamide also reduced the expression of the neuronal and astrocyte biomarkers.

**Discussion**

Concomitantly with earlier studies [2], we showed that neurotoxic effects of acrylamide are concentration- dependent and accumulate over time. It is not the accumulation of acrylamide, but rather the accumulation of damages over prolonged exposure time that leads to neurotoxicity [3]. Hence, it raises the question about the developmental consequences of prenatal acrylamide exposure and what the tolerated daily intake of acrylamide should be during pregnancy. In the SH-SY5Ycell line, acrylamide induced significant effects on differentiation starting at 10 fM, which is seven orders of magnitude lower than the estimated plasma concentration of free acrylamide in the fetus.

**Conclusions**

Although the neurotoxic concentrations in the femtomolar range seem to be specific for the SH-SY5Y cell line, the fact that micromolar concentrations of acrylamide seem to attenuate the differentiation process in both cell lines raises the interest to further investigations on the possible developmental neurotoxicity of acrylamide.

## P2-K03 CEREBROSPINAL FLUID FROM ALZHEIMER PATIENTS AFFECTS CELL-MEDIATED NERVE GROWTH FACTOR PRODUCTION AND CELL SURVIVAL IN VITRO

Homira Behbahani[1], Manuel Navarro Oviedo[1], Sumonto Mitra[1], Bengt Linderoth[3], Åke Seiger[4], Lars O. Tjernberg[1], Lars Wahlberg[5] and Maria Eriksdotter[2, 3]
Karolinska Institutet, [1]Departments of Neurobiology, Care Sciences and Society, Division of Neurogeriatrics; [2]Division of Clinical Geriatrics Huddinge, Sweden; [3]Clinical Neuroscience, Karolinska University Hospital, Stockholm Sweden, Theme Aging; [4]Stiftelsen Stockholms Sjukhem, Stockholm, Sweden; [5]NsGene Inc, Providence, RI USA
*Molecular Neurodegeneration* 2019, **14(Suppl 1):**POSTER

**Correspondence:** Homira Behbahani

**Background**

Alzheimer's disease (AD) is characterized by early degeneration of cholinergic neurons and has been associated with decreased levels of nerve growth factor (NGF) [1]. Thus, increasing the NGF levels is a potential treatment strategy [2]. Encapsulated cell bio-delivery (ECB) of NGF is an emerging technique for direct drug delivery of molecules. We previously tested ECB for delivery of NGF to the basal forebrain cholinergic neurons in ten AD patients in a first-in-human study [3, 4]. The results from these studies were promising [5] however; there was an inter-capsule difference of cell survival and NGF-release among the devices which needs to be investigated before further clinical trials. **Objective**

The aim was to identify factors that might affect the survival of encapsulated cells (NGC-0295 cell) and alterations in their NGF production, respectively.

**Methods**

We studied the effect of Abeta-peptides and IL- 1beta on a cell line (NGC-0295), overproducing NGF using apoptosis assay, flow cytometry, western blot, and liquid chromatography mass spectroscopy. Further, NGC-0295 cells were exposed to AD CSF, subjective cognitive impairment (SCI) and Lewy body dementia (LBD) patients CSF and the effect of AD CSF on NGF release was investigated and compared to SCI and LDB CSF.

**Results**

In vitro studies revealed that neither Abeta40 nor Abeta42 had any major impact on the cell viability or NGF- production at physiological concentrations. In contrast, there was a dose-dependent response of IL-1beta on NGF- production over time. The exposure of NGF-producing cells to CSF from AD patients showed significantly reduced NGF- release as compared to SCI and LBD patients CSF. Preliminary, we identified 3 differentially expressed proteins in AD CSF compared to SCI and LDB, which are involved in inflammatory pathways by mass spectrometer.

**Conclusion**

Cell survival and NGF-release are not affected by Abeta-peptides while the NGF-release is affected by IL-1beta with implications for the role of inflammation in this therapeutic platform.

## P2-K04 UNRAVELING THE SYNAPTIC VULNERABILITY IN ALZHEIMER'S DISEASE: THE PROTEOME OF THE OUTER MOLECULAR LAYER OF THE DENTATE GYRUS

Hazal Haytural[1], Saket Milind Nigam[2,3], Bengt Winblad[1], Lars O. Tjernberg[1], Susanne Frykman[1]
[1]Department of Neurobiology, Care Sciences and Society, Center for Alzheimer Research, Karolinska Institutet, Huddinge, Sweden; [2]Department of Neuroscience, Karolinska Institutet, Stockholm, Sweden, [3]Laboratory of Neurosciences, National Institute on Aging Intramural Research Program, National Institutes of Health, Maryland, USA
*Molecular Neurodegeneration* 2019, **14(Suppl 1):**POSTER

**Correspondence:** Hazal Haytural

**Background**
Synaptic dysfunction occurs early in Alzheimer disease (AD) pathogenesis and strongly correlates with cognitive decline [1]. Increasing evidence suggests that some synapses are more vulnerable than others, however, little is known about what might be causing this selective vulnerability. Therefore, in this project, we studied the proteome of the outer molecular layer (OML) of the dentate gyrus, which contains the synapses of the perforant pathway that are affected early in AD pathogenesis[2].

**Methods**
The OML of the dentate gyrus was cut out from 5 AD cases and 5 nondemented controls using laser microdissection (LMD). The microdissected tissues were dissolved, and digested by trypsin. Peptides from each sample were labeled with different isobaric tags, pooled together, and pre-fractionated into 72 fractions using high resolution isoelectric focusing (HiRIEF) [3]. Each fraction was then analyzed by liquid chromatography-mass spectrometry (LC-MS).

**Results**
In total, we calculated the relative expression levels of 7460 proteins in AD cases compared to controls. We are now in the process of analyzing the data to identify proteins with significantly altered levels in AD and controls, and to further subject them to Ingenuity Pathway Analysis.

**Discussion**
Pre-fractionation reduces sample complexity and increases the number of low-abundant proteins that can be identified and quantified, increasing the possibility to reveal proteins and pathways of importance for disease pathogenesis. This study shows that LMD combined with MS is a powerful tool to assess region-specific changes.

**Conclusions**
To our knowledge, the proteome of the OML of the dentate gyrus has not been studied before and can provide invaluable insights into the mechanisms behind the dysfunction of synapses of the perforant pathway.

## P2-K05 PERICYTE DEGENERATION IMPAIRS CAPILLARY REFLOW IN RADIATION INDUCED BRAIN INJURY

Jinping Cheng[1], Jingru Jiang[1], Baixuan He[1], Zhongshan Shi[1],Jinhua Cai[1], HaoChen[1], Yamei Tang[1,2,3]
[1]Department of Neurology, Sun Yat-Sen Memorial Hospital, Sun Yat-Sen University, Guangzhou, China; [2]Guangdong Provincial Key Laboratory of Malignant Tumor Epigenetics and Gene Regulation, Sun Yat-Sen Memorial Hospital, Sun Yat-Sen University, Guangzhou, China, [3]Guangdong Province Key Laboratory of Brain Function and Disease, Zhongshan School of Medicine, Sun Yat-Sen University, 74 Zhongshan 2nd Road, Guangzhou, China
*Molecular Neurodegeneration* 2019, **14(Suppl 1):**POSTER

**Correspondence:** Jinping Cheng

**Background**
Radiation therapy enables long term control or cure for patients with primary or metastatic head and neck tumors[1]. However, around 30% of these patients suffer from deteriorated and progressive neurological complications [2]. The underlying mechanism of radiation induced brain injury (RBI) remains unclear. Our preliminary data displayed radiation induced decrease of cerebral blood perfusion in mice and human. Pericytes are essential for regulation of vascular integrity, angiogenesis and cerebral blood flow (CBF) [3-6]. In this study we investigate how pericytes get involved in neurovascular uncoupling and lead to RBI.

**Methods**
Mice exposed to 30 Gy whole brain radiation were assessed by 7T-MRI scan and behavior test[7]. The cerebral hemodynamics change[8], blood brain barrier (BBB) permeability[9], structure and activity of neurons[10] were measured using two-photon microscope. Pathophysiological change of the brain was measured by immunofluorescence and transmission electron microscope. Protein expressions of marker of pericytes were measured by western blot.

**Results**
In mice, after whole brain single radiation, the CBF decreased and erythrocyte trapped. Both indexes were negatively correlated with the expression of PDGFRβ, pericytes density and coverage rate.

Besides, dysfunction of pericytes evoked capillary constriction, followed by BBB disruption. Increase of spine elimination and decrease of spine formation were also seen in mice with hypoperfusion and vascular leakage after radiation.

**Discussion**
PDGFRβ is essential for the survival, migration, proliferation of pericytes. Radiation caused loss of pericytes by disrupting the PDGFBB-PDGFRβ pathway[11]. Beyond that, the residual pericytes showed abnormal contract properties [12]. This causes reduction of blood oxygen supply to the neighboring cells, causing damage to BBB and neuronal spine, eventually resulted in cognition and learning dysfunction[13]. Thus, targeting pericytes might be the potential therapeutic strategy and shed light on the treatment of RBI.

**Conclusions**
Degeneration of Pericytes resulted in neurovascular uncoupling and contributed to development of RBI.

## P2-K08 AUTOPHAGOLYSOSOMAL MYELIN CLEARANCE BY SCHWANN CELLS IN WALLERIAN DEGENERATION AND SEGMENTALLY DEMYELINATING PERIPHERAL NERVES

Hwan Tae Park[1], Young-Rae Jo[1], Byeol-A Yoon[1], So-Young Jang[1], Hyun-Seok Park[2]
[1]Department of Physiology and [2]Neurosurgery, Peripheral Neuropathy Research Center (PNRC), College of Medicine, Dong-A University, Busan, South Korea
*Molecular Neurodegeneration* 2019, **14(Suppl 1):**POSTER

**Correspondence:** Hwan Tae Park

**Background**

The myelin sheath is an essential plasma membrane compaction made by Schwann cell (SC), the only glial cell in the peripheral nerves, for rapid conduction of electrical impulse through the axon. SCs have the unique ability to dedifferentiate and to destroy the myelin sheath under various demyelination condition. [1]

**Methods**

In the present study, we investigated ultrastructural morphology of demyelinating SC in Wallerian degeneration and chronic inflammatory demyelinating polyneuropathy (CIDP) using SBFscanning electron microscopy and immunoelectron microscopy. In addition, biochemical assays and fluorescence analysis on LC3-GFP-RFP mice were employed for the analysis of autophagic flux in Wallerian degeneration.

**Results**

We observed many electron microscopic findings showing diverse modes autophagy-mediated myelin clearance in demyelinating SC in Wallerian degeneration and inflammatory segmental demyelination. LC3-GFP-RFP mice showed the activation of autophagic flux in non-compact myelin regions of SC in Wallerian degeneration. Inhibition of autophagy in SC via SC-specific deletion of atg7 gene delayed demyelination in Wallerian degeneration. Finally, inhibition of lysosome resulted in a significant delay in SC demyelination in Wallerian degeneration and a CIDP mouse model.

**Discussion**

Demyelination of the peripheral nerves appears to require active phenotype changes of SC for efficient myelin clearance in lesioned nerves.

**Conclusions**

Our findings suggest that the autophagic flux in SC is required for demyelination in Wallerian degeneration and inflammatory segmental demyelination.

## P2-K09 CELL TYPE-SPECIFIC FUNCTION FOR TBK1, A TANK BINDING KINASE-1 IN SYNAPTIC GROWTH AND ORGANIZATION

Minyeop Nahm
Min-Young Noh, Su Min Lim, Seung Hyun Kim Cell Therapy Center and Department of Neurology, College of Medicine, Hanyang University, Seoul, Korea
*Molecular Neurodegeneration* 2019, **14(Suppl 1):**POSTER

**Background**

TBK1 is a multifunctional serine/threonine protein kinase that modulates various cellular functions including inflammation, autophagy, and cellular homeostasis. Recently, our group and other research groups worldwide found mutations in the TBK1 gene to be associated with amyotrophic lateral sclerosis (ALS) with or without frontotemporal dementia (FTD). However, little is known about its potential role in the regulation of synaptic structure and function.

**Methods**

To analyze synaptic morphology in the TBK1 mutants, third instar larval NMJs were visualized using the neuronal membrane marker anti-HRP antibody. The morphological phenotypes were quantified by two parameters; total bouton number and NMJ length normalized with muscle area at third instar larval NMJs onto muscle 6 and 7 of abdominal segment 2.

**Results**

To address synaptic roles of TBK1, we analyzed synaptic morphology of Drosophila TBK1 mutant allele Loss of TBK1 causes synaptic overgrowth with increase of total boutons number and NMJ length. These phenotypes were highly penetrant in all type I synapse examined. To determine whether TBK1 is required pre- or postsynaptically, we knocked down TBK1 in a tissue-specific fashion using a transgenic RNA interference (RNAi) approach. When UAS- TBK1RNAi was driven by a pan-neuronal C155-GAL4 driver, we observed presynaptic overgrowth phenotypes similar to those observed in TBK1P mutant. In contrast, expression of UAS-TBK1RNAi with the muscle-specific BG57-GAL4 or glia- specific Repo-GAL4 driver had no significant effect on overall bouton number. These data suggest that TBK1 function is required presynaptically for normal synaptic growth at the NMJ. In addition to presynaptic morphological defects, TBK1 mutant NMJs also displays increase of synaptic levels of GluRIIA subunits. A similar phenotype is induced by glia- specific knockdown of TBK1 expression.

## Conclusions

Taken together, our results provide evidence for cell-type specific roles of TBK1 in the modulation of synaptic growth and organization

P2-K10 HYPERCHOLESTEROLEMIA AND 27-HYDROXYCHOLESTEROL ENHANCE S100A8 AND RAGE EXPRESSION IN BRAIN: A LINK TOWARDS AMYLOID-BETA ACCUMULATION

Raúl Loera-Valencia[1], Muhammad-Al-Mustafa Ismail[1], Maria Lodeiro[1], Patricia Rodriguez-Rodriguez[1], Silvia Maioli[1], Laura Mateos[1], Paula Merino-Serrais[1], Ingemar Björkhem[2], Elena Puerta[1,3], Angel Cedazo-Minguez[1]

[1] Karolinska Institutet, Center for Alzheimer Research, Department of Neurobiology Care Sciences and Society, Division of Neurogeriatrics, Stockholm, Sweden; [2] Department of Laboratory Medicine, Division of Clinical Chemistry, Karolinska University Hospital, Huddinge, Sweden; [3] Department of Cellular and Molecular Neuropharmacology, Division of Neurosciences, Center for Applied Medical Research (CIMA), University of Navarra, Pamplona, Spain

*Molecular Neurodegeneration* 2019, **14(Suppl 1):**POSTER

**Correspondence:** Raúl Loera-Valencia, Muhammad-Al-Mustafa Ismail, Angel Cedazo-Minguez

## Background

Growing evidence suggest that alterations in cholesterol metabolism and in inflammatory responses have an important role in some neurodegenerative disorders including Alzheimer´s disease (AD) [1]. In the present study, to give insight on how high plasma cholesterol levels contribute to neurodegeneration and AD, we investigated the effects of high cholesterol diet and high 27- hydroxycholesterol (27-OH) concentration on the levels of S100A8 and RAGE in the brain.

## Methods

For this purpose we have used hippocampal samples of WT mice fed on a high fat diet. In addition, rat primary neurons and glial cultures treated with 27- hydroxycholesterol were used together with siRNA of retinoid receptor X gamma (RxRg) to find the mechanism influencing amyloidogenic pathways in vivo.

## Results

In this study, we report that high fat diet and excess 27-hydroxycholesterol (27-OH), a cholesterol metabolite passing from the circulation into the brain, induce the upregulation of the glial inflammatory mediator S100A8 as well as its receptor RAGE both in vivo and in vitro. S100A8 is observed as extracellular aggregates, similar to those previously reported in the hippocampi of amyloid-β (Aβ) overproducing mouse models. We found that RxRg mediates the effects of 27-OH, in a mechanism involving NF-κB activation. Moreover, 27-OH treatment enhances ADAM10, APP and reduces sAPP-alpha as a result of the same pathological cascade.

## Discussion and Conclusions

These results, together with our recent finding that S100A8 escalates the production of Aβ [2], may indicate that the S100A8/RAGE increase in the brain, is one of the mechanisms behind the association of high peripheral cholesterol and excessive 27-OH in the pathogenesis of AD.

P2-K12 NEUROPROTECTIVE EFFECTS OF A PALMITOYLATED PROLACTIN-RELEASING PEPTIDE ANALOG IN THE APP/PS1 TRANSGENIC MOUSE MODEL OF ALZHEIMER'S DISEASE

Martina Holubová[1], Michal Bencze[1,2], Blanka Železná[1], Andrea Popelová[1], Simon Gengler[3], Christian Hölscher[3], Lenka Maletínská[1]

[1]Institute of Organic Chemistry and Biochemistry of the CAS, Prague, Czech Republic; [2]Institute of Physiology of the CAS, Prague, Czech Republic; [3]Biomedical and Life Science, Faculty of Health and Medicine, Lancaster University, Lancaster, UK

*Molecular Neurodegeneration* 2019, **14(Suppl 1):**POSTER

**Correspondence:** Martina Holubová

## Background

Alzheimer´s disease (AD) is one of the most common types of dementia characterized by progressive memory loss, cognitive decline and aberrant behavior. Currently, there is no effective treatment of AD. Type 2 diabetes mellitus was reported to be a risk factor for AD, and anorexigenic and anti-diabetic insulin and glucagon-like peptide 1 agonists were found to prevent AD features in animal models of AD [1]. In this study, we examined the role of novel anorexigenic lipidized analog of prolactin-releasing peptide (PrRP) in the development of neurodegenerative changes.

## Methods

6-8 months-old APPSWE/PS1dE9 (APP/PS1) transgenic mice (n=9-10 per group) were once-daily subcutaneously injected with saline, 0.2 mg/kg liraglutide or 5 mg/kg [N-palm-γGlu-Lys11] prolactinreleasing peptide 31 (palm11-PrRP31). After 2 months of treatment, the mice were transcardially perfused with saline. One hemisphere of each brain was post-fixed in 4% paraformaldehyde and used for immunohistochemistry analysis of β-amyloid (Aβ) plaque load and neuroinflammation. The other hemisphere was used for western blot analysis.

## Results

Both liraglutide and palm11-PrRP31 were shown to significantly reduce both number and size of the β-amyloid deposits in the hippocampus and cortex of the APP/PS1 mice. Both anorexigenic lipopeptides also reduced inflammatory response associated with the Aβ plaques, namely microglia and astrocytes activation.

## Discussion

The results demonstrate clear neuroprotective effects of palm11-PrRP31 which were comparable to the previously published effects of liraglutide [2].

## Conclusions

Treatment with palmitoylated PrRP analog seems to be a promising tool for therapy of AD.

**Acknowledgements**

This work was supported by grants 16- 00918S (GA CR), RVO:61388963 (CAS) and The Alzheimer Foundation Czech Republic – AVAST Foundation.

## P2-K13 NERVE GROWTH FACTOR MUTANTS MODIFY TrkA SIGNALING CASCADE AND NEURITE OUTGROWTH CAPACITY IN HUMAN FETAL DORSAL ROOT GANGLION NEURONS

Märta Dahlström[1,3], Gunnar Nordvall[2,3,4], Erik Sundström[2,6], Elisabet Åkesson[2,6], Maria Eriksdotter[1,5], Pontus Forsell[2,3,4]
[1]Division of Clinical Geriatrics, Department of Neurobiology, Care Sciences and Society, Karolinska Institutet, Huddinge, Sweden; [2]Division of Neurogeriatrics, Department of Neurobiology, Care Sciences and Society, Karolinska Institutet, Huddinge, Sweden; [3]AlzeCure Foundation, Karolinska Institutet Science Park, Huddinge, Sweden; [4]AlzeCure Pharma AB, Hälsovägen 7, Huddinge, Sweden; [5]Department of Geriatric Medicine, Karolinska University Hospital, Huddinge, Sweden; [6]R&D unit, Stockholms Sjukhem, Stockholm, Sweden
*Molecular Neurodegeneration* 2019, **14(Suppl 1):**POSTER

**Correspondence:** Märta Dahlström

**Background**

Shortage of nerve growth factor (NGF) in the basal forebrain in Alzheimer's disease causes degeneration of cholinergic neurons [1]. NGF is a potential treatment for Alzheimer's disease, but peripheral nociceptive signaling prevents therapeutic doses of NGF [2, 3]. In the Hereditary Sensory and Autonomic Neuropathy type V (HSAN V) the mutant NGF-R100W displays conserved neurotrophic properties and decreased pain signaling [4, 5]. A variant of the HSAN V mutant, NGF-R100E [6], was examined along with NGF mutants NGF-W99A [7] and NGF-K95A/Q96A, to determine TrkA signaling and neurite outgrowth abilities.

**Methods**

Recombinant U2OS cells were used to study activation of TrkA. PC12 cells and human fetal dorsal root ganglion (DRG) neurons were used to study cell survival and neurite outgrowth.

**Results**

NGF-R100E displayed potent effects on studied functions, generally more potent than wild-type NGF. The double mutant NGF-K95A/Q96A displayed increased signaling via the TrkA receptor but had no effect on ERK1/2 phosphorylation, cell survival or neurite outgrowth. NGF- W99A activated TrkA but was unable to increase cell survival and neurite outgrowth to the same extent as NGF.

**Discussion**

NGF-R100E improved TrkA activation as well as signaling and functional effects in human DRG neurons compared with NGF. Neither TrkA-SHC1 nor TrkA-PLCγ1 signaling or phosphorylation of ERK1/2 was affected in a way that can explain the effects of the NGF-R100 mutant in HSAN V. Alterations in loop 4 of NGF were studied using the NGF- K95A/Q96A mutant. Interestingly, the TrkA signaling of NGF- K95A/Q96A is conserved and improved at high concentrations, while the neurite outgrowth abilities are completely abolished.

**Conclusions**

Differentiated activation of the TrkA signaling cascade and the neurite outgrowth capacity by NGF mutants suggest that there are possibilities to obtain selective NGF/TrkA modulation.

## P2-K14 MODEL ORGANISM DEVELOPMENT AND EVALUATION OF LATE-ONSET ALZHEIMER'S DISEASE – MODEL

Harriet Williams[1] on behalf of the MODEL-AD consortium[1,2,3,4]
[1]The Jackson Laboratory, Bar Harbor, Maine, USA; [2]Stark Neuroscience Research Institute, Indianapolis, Indiana, USA; [3]Sage Bionetworks Seattle, Washington, USA; [4]University of California, Irvine, California, USA
*Molecular Neurodegeneration* 2019, **14(Suppl 1):**POSTER

**Correspondence:** Harriet Williams

**Background**

Alzheimer's disease (AD) is the most common form of dementia, impacting at least 5 million Americans. It is estimated that by 2050, 16 million Americans will be affected by the disease. Currently there is no effective treatment or preventative strategy. One of the major obstacles facing the development of these therapeutics, is a lack of predictive animal models. One reason for this may be that the existing models are based on familial mutations, which account for only 2-5% of the AD population. The Model Organism Development and Evaluation of Late-onset Alzheimer's Disease (MODEL-AD) center has been established as a consortium, consisting of Indiana University, The Jackson Laboratory, Sage Bionetworks and University of California Irvine. Our goal is to develop panel of at least 50 mouse models for late onset AD (LOAD) that more faithfully recapitulate the human condition.

**Methods**

There are three cores: the Bioinformatics and Data Management Core (BDMC), the Disease Modeling Project (DMP) and the Preclinical Testing Core (PTC). Each core has specific aims to developing new testing pipelines and models for LOAD.

**Results**

The BDMC is charged with prioritizing novel variants, developing analytical pipelines for human-mouse phenotype comparisons, and analyzing phenotypic data. Biomarkers and disease endophenotypes will be compared to patient data wherever possible. The DMP will create new models for LOAD, based on variants identified by the BDMC, and will be phenotypically characterized through standardized pipelines. The PTC has established a pipeline for tertiary screening. This pipeline includes predetermined go/no-go criteria to evaluate the efficacy of novel compounds in newly developed models, that show an AD-like phenotype.

**Discussion**

All models, protocols, and data are to be made widely available through the Sage-Synapse portal.

**Conclusions**

We aim to seek input and collaborations from the AD community. For more information see http://www.model-ad.org

## P2-K15 RAB39B REGULATES AUTOPHAGY AND SYNAPTIC PLASTICITY

Mengxi Niu[1], Xianghua Luo[1], Lei Shi[1], Yanyan Wang[1], Manqing Liu[1], Guojun Bu[1,2], Huaxi Xu[1,3], Yun-wu Zhang[1]
[1]Fujian Provincial Key Laboratory of Neurodegenerative Disease and Aging Research, Institute of Neuroscience, Medical College of Xiamen University, Xiamen, Fujian 361102, China; [2]Department of Neuroscience, Mayo Clinic, Jacksonville, FL 32224, USA; [3]Neuroscience Initiative, Sanford-Burnham-Prebys Medical Discovery Institute, La Jolla, CA 92037, USA
*Molecular Neurodegeneration* 2019, **14(Suppl 1):**POSTER

**Correspondence:** Yun-wu Zhang

**Background**

Genetic analyses have identified that loss-of- function mutations in the RAB39B gene are responsible for X- linked mental retardation (XLMR) associated with autism, epilepsy and macrocephaly, and more recently, for early- onset parkinsonism. But the physio/pathological functions of RAB39B remain unknown.

**Methods**

We generated Rab39b knockout mice using the TALEN technique and characterized them by using electrophysiological analyses, behavioral tests and biochemical assays.

**Results**

We found that Rab39b-/Y males were viable and fertile, and born in an expected Mendelian pattern of inheritance. Rab39b-/- females were also viable but there seemed to be a trend of reduction in their born rate. There were no obvious morphological differences between Rab39b KO mice and littermate controls. Behavioral tests revealed that Rab39b-/Y males had deficiency in learning and memory, and in balancing and gait status. In addition, synaptic plasticity was markedly impaired in Rab39b-/Y mice. Moreover, loss of Rab39b resulted in impaired autophagy.

**Discussion**

RAB39B is neuronal-specific RAB GTPase protein with undetermined function. Here we show that loss of RAB39B leads to impaired autophagy and synaptic plasticity. Since autophagy deficiency has been found to compromise synaptic functions, these results suggest that autophagy deficits may be an important molecular mechanism underlying loss of function RAB39B mutation-caused XLMR and parkinsonism.

**Conclusions**

Our study reveals a novel role of RAB39B in regulating autophagy and thus synaptic plasticity.

P2-K16 AUTOPHAGY-MEDIATED Aβ METABOLISM AND NEURODEGENERATION IN ALZHEIMER'S DISEASE

Richeng Jiang[1], Simone Tambaro[1], Misaki Sekiguchi[2], Takashi Saito[2], Takaomi C Saido[2], Bengt Winblad[1], Martin Hallbeck[3], Per Nilsson[1]
[1]Department of Neurobiology, Care Sciences and Society, Division for Neurogeriatrics, Karolinska Institutet, Huddinge, Sweden; [2]Laboratory for Proteolytic Neuroscience, RIKEN Brain Science Institute, Wako, Saitama, Japan; [3]Department of Clinical and Experimental Medicine, Division of Neuro and Inflammation Science, Linköping University, Linköping, Sweden
*Molecular Neurodegeneration* 2019, **14(Suppl 1):**POSTER

**Correspondence:** Richeng Jiang

**Background**

Autophagy is dysregulated in Alzheimer's disease (AD). We have previously shown that autophagy, in addition to its degradative function, mediates the secretion of Aβ and hence directly influences Aβ plaque formation [1]. This was shown using transgenic APP overexpressing mice. Immunoelectron microscopy data indicated the involvement of multi-vesicular bodies (MVB) in this secretion [2]. Since the APP overexpression induces a number of artifacts that may influence the results we have here investigated the role of autophagy in Aβ metabolism using a novel APP knock-in mouse model of AD, APPNL-F, recently generated [3].

**Methods**

Autophagy-deficient APP knock-in mice were generated by conditional genetic deletion of autophagy- related gene 7 (Atg7) in a novel APP knock-in mouse model of AD (APPNL-F). Exosome isolation is ongoing to measure the Aβ content. The intracellular Aβ is identified by immunoelectromicroscopy. Knock down of autophagy genes is performed in SHSY-5Y cells.

**Results**

Autophagy-deficient APPNL-F mice exhibit drastically lowered Aβ plaque load as well as pronounced intracellular Aβ accumulation. Manipulation of MVB biogenesis in SHSY- 5Y cells altered the secretion of Aβ.

**Discussion**

The use of non-overexpressing mouse models facilitates interpretation of in vivo data. The lowered Aβ plaque load and the increased intracellular Aβ accumulation of the autophagy-deficient APPNL-F mice confirm our previous data and establish that autophagy plays a key role in Aβ secretion even under physiological APP levels.

**Conclusions**

Autophagy influences secretion/transfer of Aβ in neuronal cells and causes neurodegeneration in APP knock-in mouse model of AD.

P2-K18 BRAIN REGION-SPECIFIC ENHANCEMENT OF REMYELINATION AND PREVENTION OF DEMYELINATION BY THE CSF1R KINASE INHIBITOR BLZ945

**Correspondence:** Derya R. Shimshek

**Background**

Multiple sclerosis (MS) is a chronic inflammatory disease affecting the central nervous system (CNS). While multiple effective immunomodulatory therapies for MS exist today, they lack the scope of promoting CNS repair, in particular remyelination. Microglia play a pivotal role in regulating myelination processes, and the colony-stimulating factor 1 (CSF-1) pathway is a key regulator for microglia differentiation and survival.
Methods: We investigated the effects of the CSF-1 receptor kinase inhibitor, BLZ945, on central myelination processes in the 5-week murine cuprizone model by non-invasive and longitudinal magnetic resonance imaging (MRI) and histology.

**Results**

Therapeutic 2-week BLZ945 treatment caused a brain region-specific enhancement of remyelination in the striatum/cortex, which was absent in the corpus callosum/external capsule. This beneficial effect correlated positively with microglia reduction, increased oligodendrocytes and astrogliosis. Prophylactic BLZ945 treatment prevented excessive demyelination in the corpus callosum by reducing microglia and increasing oligondendrocytes. In the external capsule oligodendrocytes were depleted but not microglia and a buildup of myelin debris and axonal damage was observed.

**Discussion**

BLZ945 treatment seems to induce prompt differentiation of oligodendrocyte precursor cells (OPCs) as NG2-positive OPCs were reduced but mature oligodendrocytes (ODs) increased. Furthermore, discrepancy between MRI signal and MTR might indicate poor myelin debris removal in a given area, pointing to the importance of assessing both parameters. Prophylactic BLZ945 treatment and TREM2 knock-out animals in the cuprizone model displayed similar results indicating that the CSF1R and TREM2 pathway may converge and even interact to exert similar downstream events.

**Conclusions**

Our data suggest that a short-term therapeutic inhibition of the CSF-1 receptor pathway enhances central remyelination by modulating neuroinflammation. Hence, BLZ945 might be considered clinically for promoting myelination in combination with standard-of-care treatments in MS patients.

P2-K19 CELLULAR PROCESSES LINKING TRAUMATIC BRAIN INJURY AND ALZHEIMER'S DISEASE

Sweden

**Background**

It has been demonstrated that there is an epidemiological association between traumatic brain injury (TBI) and the development of

Alzheimer's disease (AD) later in life, but the cellular mechanism behind this link remains elusive [1].

**Methods**

In this study we aimed to investigate the cellular connection between TBI and AD by using an in vitro TBI model. Primary neurons, astrocytes and oligodendrocytes, derived from E14 wild-type mouse cortices, were exposed to synthetic Aβ42 protofibrils for 24h and then mechanically injured by scalpel cuts. The cellular response to the injury was investigated by immunocytochemistry and Western blot analysis.

**Results and discussion**

Previously, we have demonstrated that the astrocytes in the coculture engulf dead cells following the scratch injury [2]. Moreover, astrocytes readily engulf Aβ42 protofibrils, but are unable to degrade the ingested material effectively [3]. Hence, TBI and Aβ42 protofibril exposure result in high loads of engulfed material in astrocytes, which led us to focus this study on autophagy. Our results show that the number of puncta/cell and the total expression of LC3B was increased following Aβ42 protofibril exposure and TBI, indicating that the autophagy pathway was affected. However, the LC3BII/LC3BI ratio was unchanged. Importantly, there was no effect on the number of apoptotic cells following injury, Aβ42 protofibril exposure or a combination of both treatments, indicating that there is no widespread Aβ-induced cell death in direct response to the injury. To further clarify the effect on the autophagy pathway we will perform LC3B tandem sensor assays to monitor autophagic flux and also examine p62 levels. Moreover, we will investigate the long-term effect of Aβ42 protofibril exposure and TBI on cell survival and autophagy.

**Conclusion**

In order to prevent the onset of AD, a better understanding of the cellular and molecular mechanisms triggering the first steps of the disease is highly desirable. Our data suggest that phagocytic astrocytes and the autophagic processes may be interesting to study in this context.

## P2-K20 GLUTATHIONE TRANSFERASE IN CNS: GUARDIANS AGAINST NEURODEGENERATION

Helena Lindström, Birgitta Sjödin, Bengt Mannervik
Department of Biochemistry and Biophysics, Stockholm University, Sweden
*Molecular Neurodegeneration* 2019, **14(Suppl 1):**POSTER

**Background**

Glutathione transferases (GSTs) comprise a superfamily of enzymes prominently involved in detoxication by making toxic electrophiles more polar and therefore more easily excretable. Ubiquitously expressed throughout the organism, GSTs counteract pathology in all organs and tissues, including the CNS [1]. Furthermore, some GSTs have developed alternative functions, augmenting the protective effect. Thus GSTs are involved in the biosynthesis of steroid hormones including progesterone, a neurohormone the CNS levels of which are altered in pathologies including Parkinson, Alzheimer, Huntington disease, multiple sclerosis, and other neurodegenerative disorders. Estradiol triggered formation of progesterone in neurons has been found to provide important protection against oxidative stress [2].

**Methods**

To complement the characterization of the horse enzyme EcaGST A3-3 [3] with inhibition studies, 1096 FDA- approved compounds were screened for inhibitory effect on EcaGST A3-3 activity. The in vitro measurements of enzyme activity were followed spectrophotometrically.

**Results**

Our experiments have revealed that a member of the Alpha class GSTs in human, horse and pig is catalyzing the obligatory double-bond isomerization of Δ5-androstene- 3,17-dione to Δ4-androstene-3,17-dione and of Δ5- pregnene-3,20-dione to Δ4-pregnene-3,20-dione on the biosynthetic pathways to testosterone and progesterone. Five FDA-approved compounds had IC50 values in the nanomolar range and two in the micromolar range.

**Discussion and Conclusion**

The equine GST A3-3, as well as the human enzyme, is the most efficient steroid double-bond isomerase known so far in mammals, suggesting a physiologically significant role in the synthesis of steroid hormones. This notion is supported by significant suppression of progesterone synthesis in a cell line by the inhibition of GST A3-3 activity by drugs as well as by RNAi [4]. The inhibition pattern of EcaGST A3-3 is similar to that of HsaGST A3-3.

## P2-K22 CONSECUTIVE ORDER OF CASPACE ACTIVATION DETECTED BY DUAL ANCHORED FLUORESCENT SENSOR PROBES

E.V. Ivanova, E Hallberg
Department of biochemistry and biophysics, Arrhenius laboratory, Stockholm University, SE-106 91, Stockholm, Sweden
*Molecular Neurodegeneration* 2019, **14(Suppl 1):**POSTER

**Correspondence:** E.V. Ivanova

**Background**

Efficient functioning of signal transduction networks within living cells is provided, among other processes, by its dynamic regulation in space and time. This factor is particularly prominent in morphologically and functionally polarized cells, such as neurons. For instance, spatially and temporally limited activation of apoptotic proteases of caspase family has been implicated in neuronal differentiation and degeneration. Among the most popular tools for assessing protein activation in live cells are genetically encoded probes based on fluorescent proteins.

**Methods**

Being highly efficient at detecting signaling events in general, conventional fluorescent reporters typically cannot provide information on the spatiotemporal dynamics of these events. Addressing this issue, we have constructed and evaluated Tau-anchored FRET-based caspase sensors (TAFSs)[1].

**Results**

In human neuroblastoma cell lines, TAFSs are concentrated at cytoskeleton and are enriched in the processes of neuronally differentiated SH-SY5Y cells. Thereby, as compared to conventional reporters, TAFSs provide increased total signal as well as information on subcellular variations in caspase activation [1]. Furthermore, we have created caspase activity reporters based on dimerization-dependent red fluorescent proteins (ddRFP) with the anchoring tags for microtubular or synaptic localization.

**Discussion**

Coexpressing TAFSs and ddRFP-based sensors targeting different compartments or different caspases within the same cell, we expect to characterize spatiotemporal dynamics of caspase signalling in cell models of neurodegeneration.

**Conclusion**

Tau-anchored FRET-based caspase sensors (TAFSs) give spatiotemporal information and when coexpressed with ddRFP-based sensors is expected to increase our understanding of signal transduction networks within living cells.

## P2-K23 REVEALING MOLECULAR BASIS OF NEURODEGENERATION IN CORTICOSPINAL MOTOR NEURONS

P. Hande Ozdinler

Department of Neurology, Northwestern University Feinberg School of Medicine, Chicago, IL, USA

*Molecular Neurodegeneration* 2019, **14(Suppl 1):**POSTER

**Background**

Corticospinal motor neurons (CSMN) are large excitatory neurons located in layer V of the motor cortex, with unique abilities to collect, integrate and translate cerebral cortex's input into a signal that is conveyed to targets in the spinal cord, initiating and modulating voluntary movement. CSMN degeneration is the characteristic of many neurodegenerative diseases, which manifests itself with paralysis and early death. Therefore, understanding the molecular and cellular basis of CSMN degeneration is critically important for building effective treatment strategies.

**Methods**

We first generated a reporter line for CSMN, in which CSMN are genetically labeled with eGFP expression that is stable and long-lasting. We then crossed this reporter line with motor neuron disease models, which closely recapitulate human pathology and display progressive CSMN degeneration (e.g. TDP43A314T, hSOD1G93A, AlsinKO mice). Using FACS-mediated approaches CSMN are isolated as pure neuron populations and their gene expression profile is investigated at different stages of disease initiation and progression by microarray and RNASeq.

**Results**

Our results reveal that in CSMN that degenerate due to different underlying genetic causes, different cellular events are activated at distinct stages of disease initiation and progression. There are signature of events that are specific either to one type of diseased CSMN or events that occur at distinct stages of the disease. For example, cellular events that support survival and maintenance of homeostasis is upregulated at earlier time points, whereas apoptosis is initiated just prior to symptom onset.

**Discussion**

Neuronal degeneration is an active event with the involvement of numerous cellular pathways and events. Some of these pathways are common, but some are unique to the neuron and to the disease.

**Conclusions**

It is important to decipher the common and unique aspects of neurodegeneration at a cellular level, so that effective treatment strategies can be developed.

## P2-K24 POST-TRANSLATIONAL-MEDIATED RYANODINE RECEPTORS CALCIUM LEAK LEADS TO ALZHEIMER'S DISEASE-LIKE PATHOLOGIES, AND COGNITIVE DEFICITS

Renaud Bussiere[1], Alain Lacampagne[2], Xiaoping Liu[3], Steven Reiken[3], Valérie Scheuerman[2], Ran Zalk[3], Andrew F. Teich[4] , Ottavio Arancio[4], Albano C. Meli[2], Inger Lauritzen[1], Nathalie Saint[2], Charlotte Bauer[1], Fabrice Duprat[1], Cécile Martin[1], Clark A. Briggs[5], Shreaya Chakroborty[5], Grace E. Stutzman[5], Michael L. Shelanski[4], Frédéric Checler[1], Andrew R. Marks[3], Mounia Chami[1]

[1]Université Côte d'Azur, CNRS, IPMC, France, "Labex Distalz," 660 route des Lucioles, 06560 Sophia-Antipolis, Valbonne, France; [2] INSERM U1046, CNRS UMR9214, CNRS LIA1185, Université de Montpellier, CHRU Montpellier, 34295 Montpellier, France; [3]Department of Physiology and Cellular Biophysics, Clyde and Helen Wu Center for Molecular Cardiology, Columbia University College of Physicians and Surgeons, New York, New York 10032; [4]Department of Pathology and Cell Biology, Taub Institute for Research on Alzheimer's Disease and the Aging Brain and Department of Pathology and Cell Biology, Columbia University College of Physicians and Surgeons, New York, NY 10032, USA; [5]Department of Neuroscience, Rosalind Franklin University/Chicago Medical School, North Chicago, IL 60064, USA

*Molecular Neurodegeneration* 2019, **14(Suppl 1):**POSTER

**Correspondence:** Renaud Bussiere

**Background**

Data are now converging to suggest an important contribution of Endoplasmic Reticulum (ER) Ca2+ homeostasis deregulation in Alzheimer's disease (AD) pathological process. This combines pathological ER Ca2+ release via the Inositol 1,4,5-trisphosphate and the Ryanodine (RyR) Receptors. Importantly, the molecular mechanisms underlying RyR2-mediated ER Ca2+ leak in AD were not fully understood.

**Methods**

We studied post-translational remodeling of RyR2 in brains of AD patients, in two murine models of AD (3×Tg- AD and APP +/- /PS1 +/-), and in in vitro AD model (SH-SY5Y neuroblastoma cells expressing the harboring the familial APP with the double Swedish mutations (APPswe)). RyR2- mediated ER Ca2+ leak was investigated by imagery and single channel analyses. We used pharmacologic (stabilization of calstabin2 on RyR2 complex) and genetic approaches (RyR2 phosphorylated and unphosphorylated mutants) to modulate RyR2 macromolecular complex remodeling.

**Results**

We showed that post-translational modifications (phosphorylation, oxidation, and nitrosylation) occur on RyR2 in human brains and in AD models [1, 2]. We identified the molecular cascade in which Amyloid β (Aβ) activates β2- Adrenergic receptors leading to neuronal RyR2 channels post- translational remodeling thereby enhancing the ER Ca2+ leak and activating Ca2+ dependent signaling pathways which contribute to AD pathogenesis [1, 2]. We also showed that pharmacological or genetic rescue of RyR2-mediated ER Ca2+ leak reduced Aβ load, normalized behavioral and cognitive functions and improved synaptic plasticity. [2]

**Discussion**

Our data support the hypothesis that intracellular Ca2+ leak can be an early factor in the development of AD.

**Conclusions**

Data from the present study and from others raise the possibility of a vicious circle in which leaky RyR2 channels promote Aβ production and Aβ enhances RyR2 leak. This study also provides a mechanism underlying leaky RyR2 channels, which could be considered as a potential therapeutic target for AD.

1. Bussiere R., et al. Amyloid β production is regulated by β2- adrenergic signaling-mediated post-translational modifications of the ryanodine receptor. J Biol Chem. 2017 Jun 16;292(24):10153-10168. doi: 10.1074/jbc.M116.743070

## P2-K25 MYELIN BREAKDOWN LEADES TO BIN1 ACCUMULATION IN AMYLOID DEPOSITS

Pierre De Rossi[1], Robert J. Andrew[1], Tim Musial[2], Sofia V. Krause[1], Virginie Buggia-Prevot[1], Richard C. Rice[1], Peter Pytel[3], Dan Nicholson[2], Gopal Thinakaran[4]
[1]Department of Neurobiology, The University of Chicago, The University of Chicago, JFK R212, 924 East 57th Street, Chicago, IL 60637, USA; [2] Department of neurological sciences, Rush University, 600 S. Paulina St., Chicago, Illinois 60612; [3]Department of Pathology, The University of Chicago, The University of Chicago, Chicago, IL 60637, USA; [4]Departments of Neurobiology, Neurology, and Pathology, The University of Chicago, The University of Chicago, Chicago, IL 60637, USA
*Molecular Neurodegeneration* 2019, **14(Suppl 1):**POSTER

**Correspondence:** Pierre De Rossi

**Background**

The deposition of amyloid-β (Aβ) peptide in extracellular senile plaques is a hallmark of Alzheimer's disease (AD). Aβ is derived from the cleavage of amyloid precursor protein (APP) by the β- and γ-secretases. It has been proposed as the key trigger in the complex cascade of events which lead to AD.
Genome-wide association studies have recently identified BIN1 as a major susceptibility locus for late-onset AD (LOAD) We previously reported a predominant expression of BIN1 in mature oligodendrocytes and the white matter tracts. Interestingly, recent in vitro studies described a role for BIN1 in APP processing by BACE1 and Aβ production. However, the role of BIN1 in Aβ generation in vivo remains to be reported.

**Methods**

In this study, we explored BIN1 localization within the amyloid deposits in different model for Alzheimer disease. We explored the change of solubility properties of BIN1 using sequential detergent extraction. We investigated BIN1 localization within the deposits using confocal and STED microscopy. We confirmed our observations by immunoEM.

**Results**

Here, we report an increase in the levels of insoluble BIN1 in the brains of 5XFAD transgenic mice. We also observe a striking accrual of BIN1 within the amyloid deposits in multiple transgenic models of AD and in the human brain. This aberrant BIN1 localization was distinct from dystrophic neurite accumulation of APP and BACE1. Our immunoEM results suggest that the accumulation of insoluble BIN1 appears along the amyloid processes. We hypothesize that BIN1 insolubility and accumulation is a consequence of myelin destruction and the extracellular release of BIN1.

**Discussion**

Altogether, our results suggest a reorganization of myelin proteins surrounding the amyloid deposit and the change of BIN1 biophysical properties in relation to Aβ accumulation in the brain.

**Conclusions**

Our results bring new evidence of myelin protein reorganization associated with amyloid deposition. This work opens new avenues related to myelin pathology observed in AD.

## P2-K26 DELETION OF A SINGLE BIN1 ALLELE DOES NOT ALTER AMYLOID PATHOLOGY IN A MOUSE MODEL OF ALZHEIMER'S DISEASE

Robert J. Andrew, Pierre De Rossi, Thomas Guerbette, Sofia V. Krause, Elle M. Rathbun, R. Casey Rice, Gopal Thinakaran
Departments of Neurobiology, Neurology, and Pathology, The University of Chicago, Chicago, Illinois, USA
*Molecular Neurodegeneration* 2019, **14(Suppl 1):**POSTER

**Correspondence:** Robert J. Andrew

**Background**

BIN1 was identified as the second most prevalent risk factor for late-onset Alzheimer's disease (AD) in genome-wide association studies. A reduction in neuronal BIN1 isoform has been reported in patients with AD. Though little is known about BIN1 function in the brain, the loss of BIN1 expression has been proposed to influence BACE1 trafficking and Aβ production in vitro.

**Methods**

Mice with germline deletion of a single Bin1 allele were analysed for BACE1 and APP distribution by immunofluorescence microscopy. Following crossing to the 5XFAD model of amyloidosis, the amyloid burden was assessed at 4 months of age using mAb-3D6 (anti-Aβ) and ThioflavinS staining. Endogenous Aβ and soluble and insoluble Aβ were measured using MesoScale Multi-plex assays.

**Results**

Deletion of a single Bin1 allele in mice, resulted in 50% reduction in BIN1 protein levels in the brain. However, the partial loss of BIN1 expression did not grossly affect BACE1 distribution in mouse brain, alter endogenous Aβ levels or cause significant cognitive or motor deficits in a range of behavioural paradigms.
The loss of a single Bin1 allele in 5XFAD mice resulted in no significant difference in amyloid burden, as observed by immunofluorescence microscopy or ELISA, compared to littermate controls with two Bin1 alleles. The partial reduction in BIN1 expression did not alter behavioural deficits in 5XFAD mice associated with amyloid deposition in vivo.

**Discussion**

Despite prior reports of reduced neuronal BIN1 levels associated with AD and a role in BACE1 trafficking and Aβ generation in vitro, reduction of BIN1 protein within the brain of non-transgenic and 5XFAD mice resulted in no significant change in amyloid production or deposition.

**Conclusions**

Our results indicate that the mechanism(s) through which risk is conferred by polymorphisms in the BIN1 gene is not through a role in amyloid generation or deposition.

## P2-K29 A NOVEL SEX-DEPENDENT AND ESTROGEN-SENSITIVE EFFECT OF THE HUMAN GENE RPS23RG1 ON SYNAPTIC FUNCTION AND PLASTICITY

Yu Sun, Spencer Gibson, Juan C. Piña-Crespo, Huaxi Xu
Sanford Burnham Prebys Medical Discovery Institute, La Jolla, CA, USA
*Molecular Neurodegeneration* 2019, **14(Suppl 1):**POSTER

**Correspondence:** Yu Sun

**Background**

The ability of estrogens to improve hippocampus-dependent cognitive function, including learning and memory, is not well understood. Here we report a novel sex-dependent and estrogen-sensitive effect of the novel retroposed human gene RPS23RG1 [1,2] on synaptic function and plasticity in mouse hippocampus.

**Materials and Methods**

We used slice electrophysiology to study late-LTP (L- LTP) in hippocampal slices from young (21-28-days-old) and adult (2-3 months old) male and female mice transgenically expressing human RPS23RG1.

**Results**

We observed no significant difference in L-LTP between young WT and TG female (WT vs TG: 147.7 ± 2.8 vs 162 ± 4.8, p= 0.558) and male mice (WT vs TG: 159.9 ± 3.4 vs 157.5 ± 2.5, p= 0.9587). However, there was a large and significant difference in L-LTP between adult WT and TG female mice (147.0 ± 6.51 vs 205.8±3.13, p=0.0085), but not between adult WT and TG male mice (WT vs TG: 162.0 ± 2.6 vs 153.6 ± 31.62, p= 0.6380). In addition, we observed that the effects of 17-β estradiol on L- LTP was dependent on RPS23RG1 expression, even in male mice.

**Discussion**

This preliminary data and our previous observation that RPS23RG1 expression, like estrogen function, also declines during normal brain aging [3], strongly support a role for RPS23RG1 on the pro-cognitive effect of estrogen in hippocampus.

**Conclusions**

Here we describe a plasticity promoting effect of RPS23RG1 that is sensitive to estrogens and limited to the sexually mature female brain.

## P2-K32 ABNORMAL HIPPOCAMPAL AXONAL ORGANIZATION IN ADULT CONDITIONAL BACE1 KNOCKOUT MICE

Ming-Hsuan Ou-Yang[1], Jonathan E. Kurz[2], Toshihiro Nomura[3], Jelena Popovic[1], Tharinda W. Rajapaksha[1], Hongxin Dong[5], Anis Contractor[3,4], Dane M. Chetkovich[3,6], Warren G. Tourtellotte[2,7,8], Robert Vassar[1]

[1]Department of Cell and Molecular Biology, Feinberg School of Medicine, Northwestern University, Chicago, IL, USA; [2]Department of Pediatrics, Feinberg School of Medicine, Northwestern University, Chicago, IL, USA; [3]Department of Physiology, Feinberg School of Medicine, Northwestern University, Chicago, IL, USA; [4]Department of Neurobiology, Weinberg College of Arts and Sciences, Northwestern University, Evanston, IL, USA; [5]Department of Psychiatry and Behavioral Sciences, Feinberg School of Medicine, Northwestern University, Chicago, IL, USA; [6]Department of Neurology and Clinical Neurosciences, Feinberg School of Medicine, Northwestern University, Chicago, IL, USA; [7]Department of Pathology, Division of Neuropathology, Feinberg School of Medicine, Northwestern University, Chicago, IL, USA; [8]Department of Pathology and Laboratory Medicine, Department of Neurology, Neurological Surgery, and Board of Governors Regenerative Medicine Institute, Cedar-Sinai Medical Center, Los Angeles, CA, USA

*Molecular Neurodegeneration* 2019, **14(Suppl 1):**POSTER

**Correspondence:** Ming-Hsuan Ou-Yang

**Background**

The neurodegenerative Alzheimer's disease (AD) is characterized by cerebral deposition of amyloid β which, suggested by a large body of evidence, plays an important role in disease progression. β-site APP cleaving enzyme (BACE1) is the initiating enzyme in the production of Aβ and thus a prime therapeutic target of AD. However, BACE1 deletion is known to cause neurological phenotypes in germ-line BACE1 knockout mice due to insufficient cleavage of its vast variety of substrates. While BACE1 inhibitors have advanced into clinical trials, little is known about function of BACE1 in adults. The phenotypes observed in BACE1 knockout mice could result from the role of BACE1 in developmental stages. On the other hand, developmental compensation could also mask phenotypes that would be otherwise detected when BACE1 is inhibited in a later age.

**Methods**

In order to dissect the roles of BACE1 in the brain and in adult life, we generated BACE1fl/fl mice in which BACE1 gene exon 2 was flanked by loxP sites and crossed them to either CamKIIα-iCre mice to generate fore-brain specific BACE1 knockout or R26CreERT2 mice to generate tamoxifen-inducible BACE1fl/fl, R26CreERT2 mice.

**Results**

We evaluated BACE1 conditional knockout mice for phenotypes previously reported in germ-line BACE1 knockout mice and both conditional knockout mice appeared to be largely normal, with one notable exception. Surprisingly, despite having normal memory and LTP, both conditional knockout mice have abnormalities in hippocampal mossy fiber organization as reported in germ-line BACE1 knockout mice. Importantly, this abnormality is correlated with the levels of CHL1, a BACE1 substrate involved in axon guidance. Discussion Future studies will be required to understand potential functional effects of BACE1-associated mossy fiber abnormalities.

**Conclusion**

Our results agree with early clinical trials that BACE1 inhibition in adults may be well-tolerated, yet caution is warranted for long-term side-effects.

## P2-K33 AMYLOID β-CYCLOPHILIN D-OSCP MAKES THE DEVIL'S TRIANGLE OF MITOCHONDRIAL F1FO ATP SYNTHASE DYSFUNCTION IN ALZHEIMER'S DISEASE

Esha Gauba, Lan Guo, Heng Du

Department of Biological Sciences, University of Texas at Dallas, USA

*Molecular Neurodegeneration* 2019, **14(Suppl 1):**POSTER

**Correspondence:** Esha Gauba

**Background**

Synaptic mitochondrial dysfunction in Alzheimer's disease (AD) is strongly associated with F1FO ATP synthase (F1FO ATPS) deregulation, which results in inefficient OXPHOS and collapsed mitochondrial membrane potential, as well as excess mitochondrial Permeability Transition pore (mPTP) formation. Recent studies from our lab have determined the physical interaction between a F1FO ATPS subunit Oligomycin Sensitivity Conferring Protein (OSCP) and Amyloid β (Aβ), as well as the interaction between Aβ and the key mPTP regulator Cyclophilin D (CypD) in AD brains (1, 2). Given the known interplay of OSCP with CypD raises an intriguing question that whether Aβ is a factor promoting the formation of OSCP/CypD complex in AD- related pathological settings, thus inducing excess mPTP formation and aggravating synaptic mitochondrial dysfunction in AD.

**Methods**

CypD deficient mice were crossed with 5xFAD mice (an AD mouse model). The mice at different ages were used to isolate synaptic mitochondria brain to perform mitochondrial studies & brain cryosections for immunostaining and interaction studies.

**Results**

We found that the interaction between OSCP/CypD elevates in Aβ-rich conditions in AD mouse brains along with reduced synaptic mitochondrial function and F1FO ATPS dysfunction. Moreover, OSCP/Aβ interaction decreases with CypD depletion in a dose dependent manner in AD conditions. Lastly, dose-dependent CypD depletion restored the decreased OSCP levels in AD mice brain together with attenuated mitochondrial OXPHOS function, restored ATP levels, and mitigated mitochondrial calcium handling capacity as well as alleviated F1FO ATPS enzymatic activity in AD mouse brains.

**Discussion**

We have determined the binding interplay of the proteins- OSCP, CypD and Aβ and revealed the deleterious impact of such interactions on mitochondrial function in AD conditions.

## P2-K35 AMYLIN PATHOLOGY IN FAMILIAL ALZHEIMER'S DISEASE – REDUCING SYSTEMIC AMYLIN DYSHOMEOSTASIS AMELIORATES BEHAVIOR CHANGES IN ALZHEIMER'S DISEASE RATS

Han Ly[1,2], Nirmal Verma[1,2], Fengen Wu[1,2], John Hardy[3], Tammaryn Lashley[4], Claire Troakes[5], Florin Despa[1,2]
[1]Department of Pharmacology and Nutritional Sciences; [2]Department of Neurology, University of Kentucky, Lexington, USA; [3]Department of Molecular Neuroscience, Institute of Neurology, UCL, London, UK; [4]Queen Square Brain Bank for Neurological Studies, Institute of Neurology, UCL, London, UK; [5]London Neurodegenerative Diseases Brain Bank, Institute of Psychiatry, London, UK
*Molecular Neurodegeneration* 2019, **14(Suppl 1):**POSTER

**Correspondence:** Han Ly

### Background
Recent studies showed that amylin, a pancreatic amyloidogenic hormone co-secreted with insulin, forms mixed amylin-Aβ plaques in the brains of patients with sporadic Alzheimer's disease (AD) [1-3]. Here, we tested the hypotheses that amylin dyshomeostasis affects the brains of individuals with familial AD (fAD) and that reducing amylin dyshomeostasis ameliorates brain dysfunction in a rat model of amylin-Aβ interaction.

### Methods
Brains from fAD patients (n=27) and controls (n=5) were investigated for amylin deposition by immunohistochemistry and immunoconfocal microscopy. Rats that overexpress human amylin in the pancreas (HIP) rats were crossed with AD rats to generate AD-HIP rats. Littermate AD rats expressing the non-amyloidogenic rat amylin served as negative controls for brain amylin deposition. Pharmacological upregulation of epoxyeicosatrienoic acids, which are metabolites formed by endothelial cells and have anti-aggregation properties, was used to reduce amylin deposition in the brain in AD-HIP rats (16 months old; n=6/group). Behavior changes were assessed by rotarod, inclined plane and hind limb clasping tests.

### Results
fAD brains showed amylin plaques in the white matter and mixed amylin-Aβ deposits in the gray matter and in blood vessel walls. Elevated aggregated human amylin in the periphery greatly accelerated behavior changes in the AD- HIP rats compared to AD and WT littermate rats. Brain dysfunction in AD-HIP rats correlated with the formation of mixed amylin-Aβ deposits in brain parenchyma, mirroring our findings in fAD patients. Treatment improved motor coordination ($P = 0.02$), ability to maintain the balance ($P=0.04$) and lowered the hind limb clasping score ($P=0.03$).

### Discussion
fAD brains contain amylin plaques in the white matter and mixed amylin-Aβ deposits in gray matter and blood vessels. Reducing amylin dyshomeostasis ameliorates behavior changes in a rat model of amylin-Aβ interaction.

## P2-K36 FUS AFFECTS THE EXPRESSION OF CORE CANONICAL HISTONE GENES IN UNDIFFERENTIATED SH-SY5Y NEUROBLASTOMA CELLS AND IN DIFFERENTIATED NEURON-LIKE CELLS

Pawel Czubak, Katarzyna Dorota Raczynska
Department of Gene Expression, Institute of Molecular Biology and Biotechnology, Adam Mickiewicz University, Poznan, Poland
*Molecular Neurodegeneration* 2019, **14(Suppl 1):**POSTER

**Correspondence:** Pawel Czubak

### Background
FUS (fused in sarcoma) is a multifunctional nuclear RNA-binding protein, expressed in neuronal and non- neuronal tissues [1; 2]. Mutations in the FUS gene cause familial amyotrophic lateral sclerosis (FALS) type 6 [3; 4]. This leads to almost abolished or significantly reduced nuclear import of FUS and due to repeated stress to cytoplasmic accumulation of FUS aggregates in neurons and glial cells of ALS patients. Mislocalized FUS mutants sequester RNA- binding proteins, resulted in the extensive pre-mRNA processing defects in cells [5; 6; 7]. One of the FUS function is to activate core canonical histone genes expression during the DNA replication phase in complex with U7 snRNP [8]. Therefore, we hypothesize that one of a consequences of the loss of the nuclear function of ALS-linked FUS mutants is U7 snRNP mislocalization and de-regulation of histone gene expression.

### Methods
SH-SY5Y neuroblastoma cells (wild type (WT) and FUS KO cell lines) were grown in Dulbecco's modified Eagle medium (DMEM). Cells were differentiated per ten days with the use of retinoic acid (RA) at the concentration of 75 μmol/mL RA to obtain neuron-like cells. After that, using a quantitative reverse transcription PCR (RT-qPCR) technique, we investigated the effects of FUS on the 3'end processing efficiency of the histone gene transcripts in differentiated cells in comparison to undifferentiated cells.

### Results
FUS influences the expression of histone genes in neuroblastoma cells by affecting the 3'end processing of their transcript. The effect was different in undifferentiated SH- SY5Y neuroblastoma cells and differentiated neuron-like cells.

### Discussion
FUS influences the replication dependent histone gene expression both in proliferating neuroblastoma cells and in terminally differentiated neuron-like cells, that might correspond to glial cells and motor neurons, respectively. We suggest, that ALS-linked FUS mutations result in de-regulation of histone gene expression and lead to altered cell homeostasis in ALS patients.

## P2-L01 HIGH-RESOLUTION 3D IMMUNO-IMAGING OF HYPERPHOSPHORYLATED TAU AND BETA-AMYLOID IN THE HUMAN LOCUS COERULEUS REGION AND NEOCORTEX BY LIGHT SHEET MICROCSOPY

Csaba Adori[1], Gabor G. Kovacs[2], Linnea Kyrö[1], Zsofia Magloczky[3], Mathias Uhlen[1,4], Tomas Hökfelt[1]
[1]Department of Neuroscience, Karolinska Institutet, Stockholm, Sweden, [2]Medical University of Vienna, Institute of Neurology, Vienna, Austria; [3]Institute of Experimental Medicine, Hungarian Academy of Sciences, Budapest, Hungary, [4]KTH-Royal Institute of Technology, Science for Life Laboratory, Stockholm, Sweden
*Molecular Neurodegeneration* 2019, **14(Suppl 1):**POSTER

**Correspondence:** Csaba Adori

**Background**

iDISCO volume imaging (immunolabelling- enabled three-dimensional imaging of solvent-cleared organs), combined with light sheet microscopy, is a powerful method enabling three-dimensional (volume) imaging of cleared and immunolabelled structures in complex tissues (e.g. entire human locus coeruleus (LC), or pieces of neocortex in the 1-10 mm range, including all layers). Its short clearing time makes iDISCO especially suitable for immunostaining [1]. A recent hypothesis suggests that the LC region represent an early starting point for Alzheimer's disease pathology, even preceding the occurrence of cortical lesions [2]. Our earlier studies emphasized the role of the somatostatin receptor 2 in the maintenance of LC neurons [3].

**Methods**

Here we show preliminary results with iDISCO+ volume immunostaining of hyperphosphorylated tau in neocortex and LC as well as beta-amyloid 3D staining in the neocortex from short post-mortem time subjects with neuropathologically verified late-stage Alzheimer's disease. We worked out a special way of tissue fixation that allows a highly efficient epitope-preservation.

**Results**

Amyloid plaques of various sizes were distributed throughout all layers of the Brodmann 4 cortical area with no apparent layer preference, and a subset of layer III pyramidal neurons exhibited intracellular beta-amyloid accumulation. Particularly high density of tau-AT8-immunoreactive tangles and dystrophic neurites were found in the upper cortical layers of the same Brodmann area. We also observed a subset of neurons containing intracellular tau-AT8 accumulation in the LC, dorsal raphe and in the medial parabrachial nucleus.

**Discussion**

The method here described allows a deeper analysis of the 3D pattern of these (and other) pathological protein inclusions, including their spatial distribution compared to various cell types (pyramidal neurons/interneurons), innervations or vasculature.

**Conclusion**

Taken together, studies of this type, involving subjects also with earlier stages of neurodegenerations, may provide novel information concerning generation and spreading of hyperphosphorylated tau and beta-amyloid in the human brain.

## P2-L03 CHARACTERIZATION OF THE POSTSYNAPTIC PROTEIN NEUROGRANIN IN PATIENTS WITH FAMILIAL ALZHEIMER'S DISEASE, SPORADIC ALZHEIMER'S DISEASE, PATHOLOGICAL AGING AND HEALTHY CONTROLS

Hlin Kvartsberg[1,2], Tammaryn Lashley[3], Christina E Murray[3], Henrik Zetterberg[1,2,4,5], Kaj Blennow[1,2], Erik Portelius[1,2]
[1]Department of Psychiatry and Neurochemistry, Institute of Neuroscience and Physiology, The Sahlgrenska Academy at the University of Gothenburg, Mölndal, Sweden; [2] Clinical Neurochemistry Laboratory, Sahlgrenska University Hospital, Mölndal, Sweden; [3] Queen Square Brain Bank for Neurological Disorders, Department of Molecular Neuroscience, UCL Institute of Neurology, London, UK; [4] Department of Molecular Neuroscience, UCL Institute of Neurology, Queen Square, London, UK; [5] UK Dementia Research Institute at UCL, London, UK
*Molecular Neurodegeneration* 2019, **14(Suppl 1):**POSTER

**Correspondence:** Hlin Kvartsberg

**Background**

Synaptic degeneration and neuronal loss are believed to be early events in Alzheimer's disease (AD), probably long before symptom onset. Thus, synaptic proteins are highly relevant for enabling early diagnosis. The postsynaptic protein neurogranin (Ng) has recently emerged as a promising cerebrospinal fluid biomarker for AD, even during the prodromal phase. [1] Here we aimed to quantify endogenous peptides and full-length (FL)-Ng in AD, familial AD (FAD), pathological aging (PA, brains with Aβ and tau deposition but no cognitive impairment) and healthy controls in two different brain regions.

**Methods**

Brain tissue from parietal lobe included AD (n=10) and age matched healthy controls (Co) (n=10) while samples from temporal cortex included AD (n=9), FAD (n=10, mutations in PSN1 or APP), PA (n=5) and Co (n=4). Samples were homogenized in TBS and analyzed by WB, ELISA and immunoprecipitation combined with mass spectrometric analysis. All antibodies were in-house-generated monoclonal antibodies.

**Results**

In AD parietal lobe, there was an inverse relationship between Ng fragments and FL-Ng; 10 endogenous Ng peptides were increased whilst FL-Ng was decreased. In temporal cortex, AD and FAD showed similar trends. PA, however, had significantly increased FL-Ng according to immunoprecipitation and WB, but peptide concentrations were similar to Co.

**Discussion**

As both CSF and brain Ng peptides seem to increase in AD, and possibly FAD, combined with a decrease of FL-Ng, this might reflect both ongoing synaptic loss and perhaps an increased enzymatic activity that digests Ng. PA, however, showed fewer peptides and higher amounts of FL- Ng. Considering that PA patients have a degree of AD pathology without cognitive impairments, the increased amount of FL-Ng could be compensating for the spreading neurodegenerative disease process.

**Conclusions**

The inverse relationship between endogenous Ng peptides and FL-Ng in AD brains suggests that Ng degradation may play a role in AD-related synaptic degeneration.

## P2-L04 WIDESPREAD DISTRIBUTION OF TAUOPATHY IN PRECLINICAL ALZHEIMER'S DISEASE

Stephanie A. Schultz[1,2], Brian A. Gordon[2,3,4], Shruti Mishra[2], Yi Su[2], Nigel J. Cairns[3,5], John C. Morris[3,5], Beau M. Ances[3,5†], Tammie L.S. Benzinger[2,3,6†]
[1]Division of Biology and Biomedical Sciences, Washington University School of Medicine, St. Louis, MO; [2]Department of Radiology, Washington University School of Medicine, St. Louis, MO; [3] Knight Alzheimer's Disease Research Center, Washington University School of Medicine ,St. Louis, MO; [4]Department of Psychological & Brain Sciences, Washington University, St. Louis, MO; [5]Department of Neurology, Washington University School of Medicine, St. Louis, MO; [6]Department of Neurological Surgery, Washington University School of Medicine, St. Louis, MO
*Molecular Neurodegeneration* 2019, **14(Suppl 1):**POSTER

**Correspondence:** Stephanie A. Schultz
[†] Contributed equally

**Background**

The Braak and Braak staging of neurofibrillary tangle (NFT) pathology in Alzheimer disease (AD) proposes that tauopathy in the form of NFTs develops early in the medial temporal lobes in cognitively normal (CN) adults. However, in vivo quantification remains unexamined in individuals with preclinical AD. Understanding both the spatial pattern and severity of tauopathy in brain positron emission tomography (PET) scans in this at-risk group is of great importance in the context of designing clinical trials and pharmacological interventions. Therefore the aim of this study was to Leverage the recent development of tau-PET to examine the distribution and severity of tauopathy in CN adults with preclinical AD as determined by positive beta-amyloid biomarkers.

**Methods**

One hundred and ten CN older adults underwent 18F-AV-1451-tau-PET and florbetapir-beta-amyloid-PET imaging. Tau-PET data were processed with 34 cortical and 9 subcortical FreeSurfer regions and averaged across both hemispheres. Individuals were classified as being beta amyloid-positive (preclinical AD) or negative (control) based on a beta-amyloid-PET value. We compared the tau-PET binding in the two groups using linear regressions, adjusting for age and sex.

**Results**

After adjustment for multiple comparison correction, the preclinical AD cohort had higher tau- PET binding within 8 regions: precuneus (B=0.115, p=0.003), amygdala (B=0.133, p=0.004), banks of the superior temporal sulcus (B=0.107, p=0.002), entorhinal cortex (B=0.173, p=0.003), fusiform gyrus (B=0.096, p=.001), inferior parietal cortex (B=0.114, p=0.002), inferior temporal cortex (B=0.111, p=.002), and middle temporal cortex (B=0.108, p=0.001).

**Discussion**

Our study reports elevated patterns of tauopathy in preclinical AD in the medial temporal lobe and parietal lobe and association regions, suggestive of more widespread tauopathy early in the disease process.

**Conclusions**

These results suggest that therapies targeting tauopathy could be considered earlier in the disease course in order to prevent or ameliorate cognitive decline.

## P2-L05 STORE-OPERATED CALCIUM ENTRY AND DENDRITIC SPINES PATHOLOGY IN ALZHEIMER'S DISEASE

Ekaterina Pchitskaya[1], Elena Popugaeva[1], Nina Kraskovskaya[1], Daria Chernyuk[1], Huz Zhang[2], Ilya Bezprozvanny[1,2]
[1]Laboratory of Molecular Neurodegeneration, Department of Medical Physics, Peter the Great St. Petersburg Polytechnic University, St. Petersburg, Russian Federation; [2]Department of Physiology, UT Southwestern Medical Center at Dallas, Dallas, TX, 75390, USA
*Molecular Neurodegeneration* 2019, **14(Suppl 1):**POSTER

**Correspondence:** Ekaterina Pchitskaya

**Background**

Alzheimer's disease (AD) is the most common reason for elderly dementia, and its prevalence will continue to increase with aging population of the world. Previously we observed that reduced postsynaptic neuronal store-operated calcium entry (nSOC) is responsible for mushroom spine loss in knock-in AD mice model expressing mutant human presenilin 1 (PS1-M146V-KI)[1].

**Results**

In previous studies we observed downregulation of mushroom dendritic spines in hippocampal neurons in conditions of low amyloid toxicity [2] and in newly generated APP-KI mice model of AD [3, 4]. We reasoned that the loss of mushroom spines occurs due to impaired dendritic spine nSOC, which is mediated in hippocampal neurons by STIM2 endoplasmic reticulum resident protein. SOC is relatively well studied in non-excitable cells, but still little is known about its regulation in neuronal cells. In our recent study we found a new STIM2 binding partner – tubulin associated protein EB3[5]. EB3 protein transiently enters dendritic spines at the end of growing microtubule (MT) [6]. We shown that STIM2- EB3 association is necessary for normal dendritic spines morphology and nSOC, therefore new potential nSOC regulating pathways in neurons and link between neuronal cytoskeleton and calcium signaling were identified. We further demonstrated that EB3 overexpression rescues mushroom dendritic spine deficiency in PS1-M146V-KI neurons [5]. Therefore, influencing dynamic microtubules and dendritic spine nSOC through overexpression of tubulin plus-end binding partner EB3 showed promising result in AD mouse model.

**Conclusions**

We propose that loss of mushroom dendritic spines and dysregulation of calcium homeostasis is a common driving mechanism of AD pathogenesis in both APP and presenilin-based models [7]. Finding ways which will helps to restore detected calcium signaling abnormalities is under urgent need. Observed by us link between MT, dendritic spines morphology and calcium signaling indicate that development of MT-targeting drugs is potential strategy for treatment of AD and other neurodegenerative diseases. Targeting of neuron-specific MTbased signaling pathways may help to overcome toxicity of such compounds.

**Funding**

This work is supported by the Russian Science Foundation Grant 14–25–00024–П (IB). EP is a holder of Russian Presidential Stipend СП–1929.2018.4

**Publisher's Note**

**Springer Nature remains neutral with regard to jurisdictional claims in published maps and institutional affiliations.**

Publisher’s Note

Springer Nature remains neutral with regard to jurisdictional claims in published maps and institutional affiliations.

